# Decarboxylative Hydroxylation of Benzoic Acids

**DOI:** 10.1002/anie.202108971

**Published:** 2021-10-04

**Authors:** Wanqi Su, Peng Xu, Tobias Ritter

**Affiliations:** ^1^ Max-Planck-Institut für Kohlenforschung Kaiser-Wilhelm Platz 1 45470 Mülheim an der Ruhr Germany; ^2^ Institute of Organic Chemistry RWTH Aachen University Landoltweg 1 52074 Aachen Germany

**Keywords:** decarboxylation, ligand to metal charge transfer, phenol synthesis

## Abstract

Herein, we report the first decarboxylative hydroxylation to synthesize phenols from benzoic acids at 35 °C via photoinduced ligand‐to‐metal charge transfer (LMCT)‐enabled radical decarboxylative carbometalation. The aromatic decarboxylative hydroxylation is synthetically promising due to its mild conditions, broad substrate scope, and late‐stage applications.

## Graphical abstract

Herein, the first decarboxylative hydroxylation reaction to synthesize phenols from benzoic acids is reported. The method overcomes the challenges associated with conventional decarboxylation of benzoic acids and can be applied even for the late‐stage functionalization.

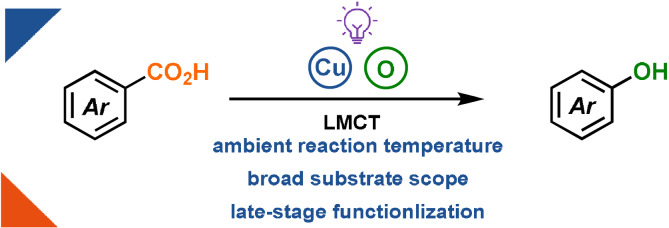

Phenols are valuable building blocks in natural products, pharmaceuticals, and functional materials and can be synthesized from a variety of different aryl precursors, yet, not directly from benzoic acids. Conventional polar or radical decarboxylation have failed to provide a solution to phenol synthesis. The high activation barrier for polar decarboxylation often requires reaction temperatures of 140 °C or more and *ortho*‐substituents on the substrates.^[1]^ The slow rate of conventional radical aromatic decarboxylation (about three orders of magnitude slower than the rate of aliphatic counterparts[^2^, ^3^]) results in undesirable side reactions that outcompete productive decarboxylation. Consequently, there is currently no general method available to access phenols directly from benzoic acids.^[4]^ Here, we report the first general protocol for decarboxylative hydroxylation of benzoic acids. The phenol synthesis is enabled by a radical decarboxylation through ligand to metal charge transfer (LMCT) in copper carboxylates, which produces aryl radicals for subsequent capture by copper followed by C‐O reductive elimination from arylcopper(III). The decarboxylative hydroxylation follows our recently introduced concept of radical decarboxylative carbometalation via copper carboxylates.^[5]^ Independent of the mechanism, the method overcomes the challenges associated with conventional decarboxylation of benzoic acids,^[6]^ enables a hitherto unknown transformation, and can be applied for the late‐stage functionalization.^[7]^

More than 99 % of phenol production is based on the cumene process, the radical oxidative cleavage of isopropylbenzene with dioxygen to yield phenol and acetone.^[8]^ More complex phenols can be prepared by transition‐metal catalyzed cross‐coupling and C−H activation reactions. Common starting materials for such state‐of‐the‐art methods include aryl diazonium salts,^[9]^ aryl (pseudo)halides,^[10]^ aryl sulfonium salts,^[11]^ aryl boronic acids,^[12]^ aryl silanes,^[13]^ and arenes themselves.^[14]^ While benzoic acids are abundant, stable, and available in large structural diversity from commercial sources, direct access to phenols by cleavage of the C−C bond via decarboxylation and formation of the C−O bond has been elusive (Figure 1 A), because conventional decarboxylation strategies lack the opportunity to combine both steps due to their intrinsic reactivity profile. Decarboxylative C−C and C‐heteroatom bond formation of benzoic acids have mostly been achieved by transition‐metal‐mediated or ‐catalyzed thermal decarboxylative carbometalation, to generate arylmetal intermediates for reductive elimination with versatile coupling partners.^[1]^ However, the activation barriers (24–30 kcal mol^−1^)^[15]^ require forcing reaction conditions, as well as activating *ortho*‐substitutents that can decreased the barriers by 3–5 kcal mol^−1^ due to their destabilization effect.^[15]^ For example, decarboxylative etherification of simple benzoic acids with activating ortho‐substituents was achieved with a Ag/Cu bimetallic catalyst combination at 145 °C with ortho silicates as oxygen donors.^[16]^ We are not aware of any other general decarboxylative C−O bond forming reaction of benzoic acids. Radical decarboxylation can proceed much faster at activation barriers of about 8–9 kcal mol^−1^ [^2^, ^17^] to afford synthetically useful aryl radicals. Aliphatic acids activated through this pathway have been used successfully for radical addition reactions,^[18]^ carbometalation,^[19]^ and radical crossover.^[20]^ However, even with the low barrier for radical aromatic decarboxylation, other reactions such as hydrogen atom abstraction (HAT) and back electron transfer (BET) can be even faster^[2]^ and result in undesired reactivity. Radical decarboxylation of benzoic acids has been successfully used to react with reactive radical acceptors such as (hetero)arenes,^[21]^ acrylates, or diboron species,^[22]^ but not to make C−O bonds, even with prior activation to activated esters.^[23]^

**Figure 1:**
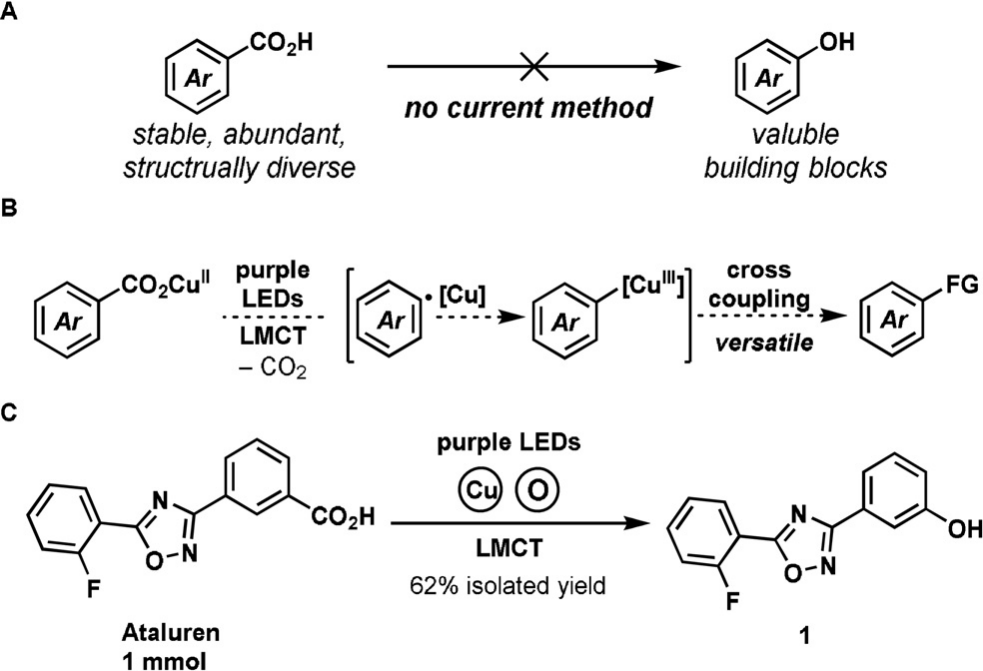
A) No synthetic method available from benzoic acids to phenols. B) A general decarboxylative cross‐coupling strategy enabled by radical decarboxylative carbometalation. C) Example of late‐stage decarboxylative hydroxylation of ataluren on 1 mmol scale.

Because conventional decarboxylation strategies so far have not been successful to address decarboxylative hydroxylation of benzoic acids, we attempted to approach the problem with a new concept using copper ligand‐to‐metal charge transfer (LMCT). Our group reported a conceptually different decarboxylative cross‐coupling strategy of benzoic acids, which combined a low‐barrier radical decarboxylation process enabled by photoinduced copper ligand‐to‐metal charge transfer (LMCT) with subsequent carbometalation to afford putative arylcopper (III) complexes (Figure 1 B).[^5^, ^24^] The light‐mediated LMCT from carboxylate to copper enables radical formation, and the copper mitigates undesired side reactions, and presumably captures the aryl radical to form arylcopper(III) for fast reductive elimination. Based on our findings in fluorination via copper LMCT,^[5]^ we report here the first practical synthesis of phenols from benzoic acids, through irradiation of in situ formed copper carboxylates and subsequent hydrolysis of the resulting aryl esters, as exemplified by decarboxylative hydroxylation of ataluren, a drug for the treatment of Duchenne muscular dystrophy, in 62 % isolated yield on a 1 mmol scale (Figure 1 C).

During the development of our decarboxylative fluorination of benzoic acids,^[5]^ we discovered a remarkably efficient decarboxylative C−O bond formation reaction when fluoride was omitted. The aryl carboxylate functioned both as substrate and as nucleophile to yield homo‐coupled benzoic ester in near‐quantitative yield (Figure 2 A). Yet, the theoretical yield of such transformation is limited to 50 % based on the limiting reagent, the benzoic acid. To prevent the sacrificial use of half of the substrate, we sought to identify an exogenous oxygen‐based nucleophile that is suitable for our strategy. Hydroxide, phenoxide, and alkoxides shut down productive decarboxylation, possibly due to outcompeting carboxylate for coordination to copper, which precludes productive carboxylate to copper LMCT.^[25]^ Aliphatic carboxylates gave low oxydecarboxylation yields, presumably because they undergo decarboxylation much faster than benzoates. Aryl carboxylates performed superior in the C−O bond forming event to all other oxygen‐based nucleophiles analyzed. Assuming that both substrate and nucleophile aryl carboxylates coordinate to copper, both would also undergo LMCT to generate carboxyl radicals. A desirable scenario to overcome this conundrum would be that the nucleophile carboxyl radical would undergo decarboxylation at a rate slower than the substrate carboxyl radical. Instead, the nucleophile carboxyl radical should undergo fast back electron transfer (BET) or hydrogen atom abstraction (HAT) to reform the acid that can act as nucleophile (Figure 2 B).

**Figure 2:**
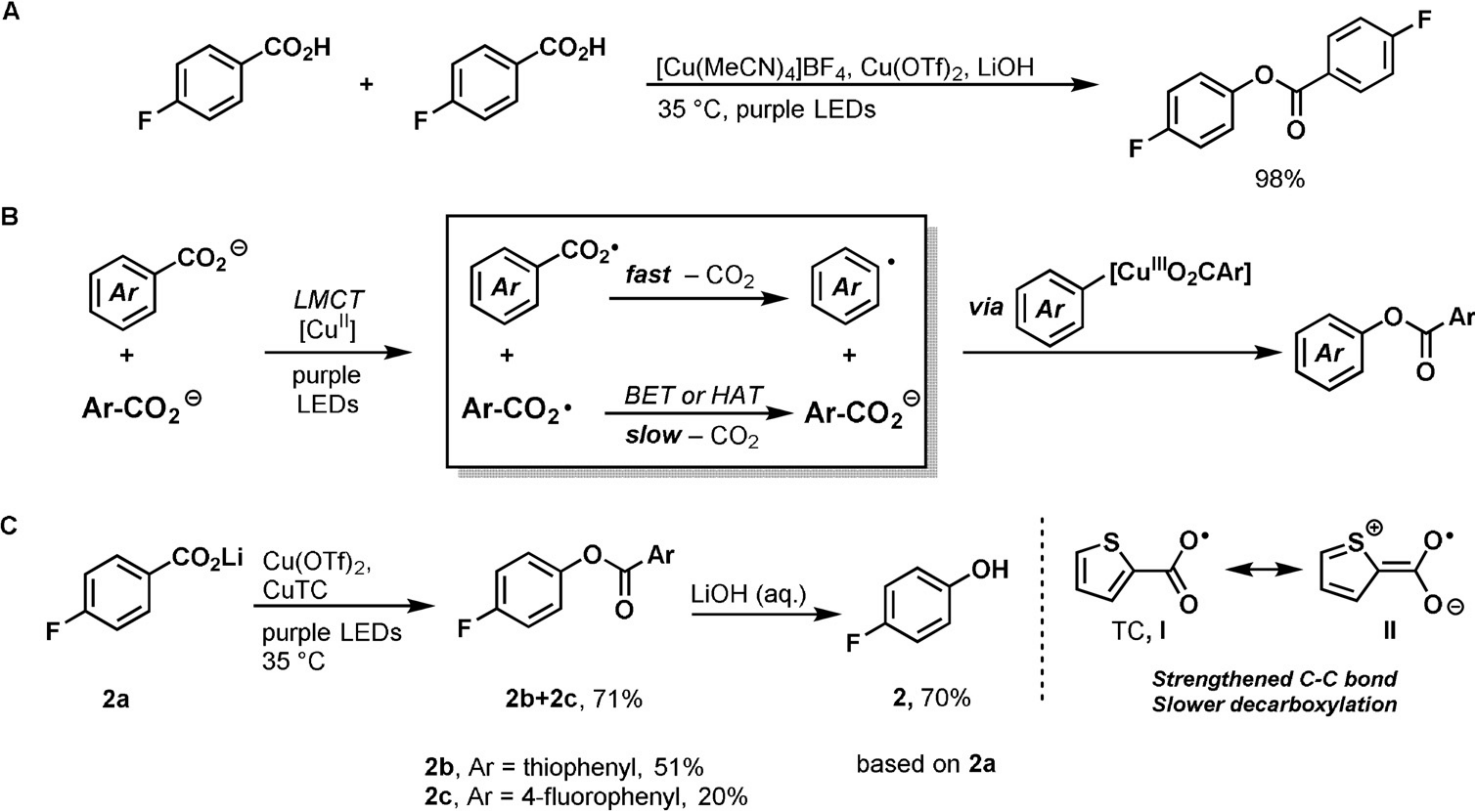
A) Decarboxylative C−O bond formation of 4‐fluorobenzoic acid. Reaction conditions: 4‐fluorobenzoic acid (0.05 mmol, 1.0 equiv), LiOH (4 equiv), [Cu(MeCN)_4_]BF_4_ (2.5 equiv), Cu(OTf)_2_ (2.5 equiv), MeCN (*c*=25 mM), 16 h purple LEDs irradiation, 35 °C. B) Reaction design. C) Left: use of copper(I) thiophene‐2‐carboxylate (CuTC) as nucleophile. Right: electron delocalization strengthens the C‐COO^.^ bond in the TC radical.

Decarboxylation of the electron‐rich 4‐methoxybenzoyloxyl radical proceeds slower by about an order of magnitude than for the electron‐neutral benzoyloxyl radical, presumably due to strengthening of the Ar‐COO^.^ bond caused by the conjugation of an appropriately positioned π donor on the arene.^[2]^ Consistent with this hypothesis, we identified thiophene‐2‐carboxylate (TC) as the most promising coupling partner (Figure 2 C). π Donation by the sulfur atom should strengthen the C‐COO^.^ bond, which would provide a sufficient rate difference in decarboxylation of the two acids to achieve synthetically useful yields based on the substrate. Purple LEDs irradiation of a mixture of **2 a**, CuTC, and Cu(OTf)_2_ in MeCN resulted in clean conversion to ester **2 b** in 51 % yield, and an additional 20 % of ester **2 c**, in which the substrate functioned as both radical donor and nucleophile, corresponding to a total of 91 % mass conversion of **2 a** that is accounted for. Hydrolysis was performed without isolation of the esters and afforded 70 % overall yield of phenol **2**, together with 20 % starting material. No oxydecarboxylation product of TC was detected and less than 10 % of TC protodecarboxylation was observed, consistent with our design. While other sources of TC also provided product, copper(I) thiophene‐2‐carboxylate (CuTC) provided the best result as protodecarboxylation was suppressed and conversion to ester **2 b** was increased. We speculate that the advantage of CuTC can be explained by capture of the aryl radical by CuTC species with subsequent oxidatiton by Cu^II^ to generate arylcopper(III)TC for C‐O reductive elimination. The process (deprotonation, decarboxylative oxygenation, and hydrolysis) can be carried out in the same pot but initial deprotonation of the benzoic acids to form their lithium salts before addition of copper generally afforded higher yields, consistent with the formation of copper carboxylates for efficient LMCT. A low concentration (25 mM) was necessary to promote light transmission, due to the heterogeneity of the reaction mixture.

Both electron‐poor and electron‐rich substrates, such as 4‐cyanobenzoate (**3**) and 3,5‐dimethylbenzoate (**13**) can outcompete TC to afford the corresponding phenols (Table 1). Electron neutral benzoates, which are often problematic for thermal decarboxylation due to the lack of electronic bias,^[16]^ or electron deficient benzoates, which are often problematic for oxidative radical decarboxylation due to their high oxidation potential,[^21b^, ^22^] performed well. Heteroaryl carboxylates, such as isonicotinic carboxylates (**6**, **14**) and quinoxaline‐2‐carboxylate (**15**), are also compatible. Functional groups including aryl halides (**2**, **25**, **27**, **31**), oxidation‐sensitive aldehydes (**10**), enolizable ketones (**18**, **28**), heterocycles (**1**, **6**, **7**, **14**, **24**), sulfonamides (**16**, **17**, **19**, **24**), amides (**22**), ether (**12**, **31**) and nitriles (**3**, **8**, **29**) are well tolerated. α‐Heteroatom (**9**, **12**, **16**, **17**, **22**), benzylic (**7**, **11**, **13**, **16**, **23**, **28**, **31**) and tertiary (**9**, **23**) C−H bonds that are sensitive to HAT processes also did not pose a problem. Alkyl esters (**9**) are tolerated due to the more facile cleavage of aryl esters.^[26]^ The synthetic utility was further demonstrated by decarboxylative hydroxylation of several complex small molecules (**1**, **9**, **17**, **24**, **31**) at a late stage. In summary, substrates such as electron‐deficient to electron‐rich benzoic acids with versatile functional groups, (hetero)aryl carboxylic acids and several complex small molecules were included. However, substrates such as benzoic acids bearing large *ortho*‐substituents and some heteroaryl carboxylates, gave low yields. Strong coordinating or oxidizable functional groups, such as phenols and amines are not tolerated. The remaining mass balance consists mostly of the starting material, for example, the benzoic acid that either did not decarboxylate or served as oxygen nucleophile.

**Table 1:**
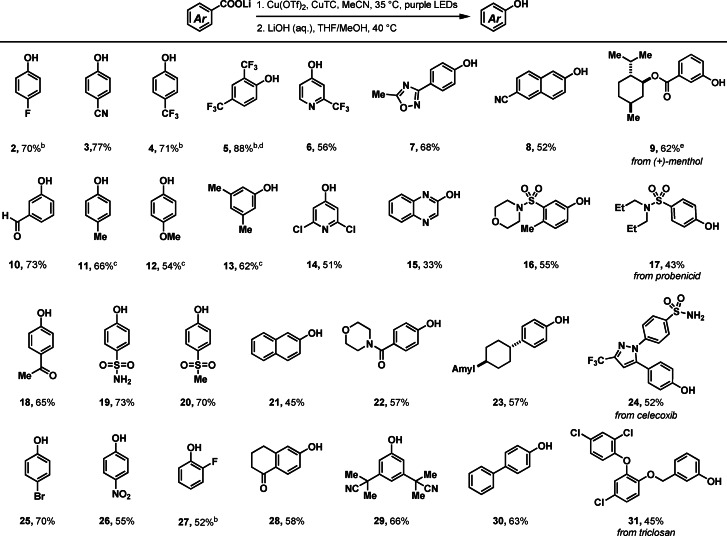
Decarboxylative hydroxylation of benzoic acids.^[a]^

[a] Standard reaction conditions: 1. lithium carboxylate (0.20 mmol, 1.0 equiv), CuTC (1.5 equiv), Cu(OTf)_2_ (2.5 equiv), MeCN (*c*=25 mM), 16 h purple LEDs irradiation, 35 °C; 2. 1 M LiOH (aq.), THF/MeOH (1:1, v/v, 55 mM), 40 °C. [b] Yields based on ^19^F NMR integration with internal standard 2‐fluorotoluene (0.20 mmol, 1.0 equiv). [c] Yields based on ^1^H NMR integration with an internal standard dibromomethane (0.20 mmol, 1.0 equiv). [d] Reaction conditions: carboxylic acid (0.20 mmol, 1.0 equiv), LiOH (4 equiv), [Cu(MeCN)_4_]BF_4_ (2.5 equiv), Cu(OTf)_2_ (2.5 equiv), CD_3_CN (*c*=50 mM), 16 h purple LEDs irradiation, 35 °C. [e] 2. Aminolyisis: nBuNH_2_ (2.0 mmol, 10 equiv), benzene (0.10 M), 25 °C.

We propose that irradiation of the copper(II) carboxylate results in carboxylate to Cu^II^ charge transfer (LMCT) (Figure 3 A).^[5]^ Subsequent homolysis of the O‐Cu^II^ bond produces an aroyloxyl radical which decarboxylates to afford aryl radical for immediate capture by copper. While LMCT from Cu^II^TC proceeds accordingly, TC is regenerated from TC radical by BET or HAT. Both LMCT steps of the copper(II) carboxylates are supported by the observation that a mixture of lithium 4‐fluorobenzoate (**2 a**) and Cu(OTf)_2_, and a mixture of CuTC and Cu(OTf)_2_ both show a significant absorbance at 370–470 nm in their UV/Vis absorption spectra, which is ascribed to the LMCT band of copper(II) carboxylates (Figure 3 B).^[27]^ The LMCT band overlaps with the purple LED emission spectrum, consistent with excitation of the copper(II) carboxylates under the reaction conditions.^[5]^ Generation of the aroyloxyl radical of the substrate via LMCT is supported by formation of lactone **33** via 6‐endo‐trig intramolecular radical cyclisation^[21b]^ (Figure 3 C) and formation of 4‐methoxybenzoate (**36**) via radical trapping with benzene^[5]^ (Figure 3 D). Decarboxylation of the aroyloxyl radical to aryl radical is supported by isolation of 4‐methoxy‐1,1′‐biphenyl (**35**) from the same radical trapping experiment (Figure 3 D).^[5]^ The generated aryl radical is trapped by a Cu^II^TC complex, or by Cu^I^TC with subsequent oxidation by Cu^II^ to afford an arylcopper(III)TC in both cases for C−O bond reductive elimination. Copper assisted aryl radical capture^[28]^ with subsequent C‐O reductive elimination from arylcopper(III) complex^[29]^ to yield aryl TC ester^[15]^ is a known process. Reduction of Cu^II^ to Cu^I^ as the reaction progresses is supported by the continuous decrease of the Cu^II^‐based d‐d transition band (550 nm–900 nm)^[27]^ in the UV/Vis spectrum of the reaction mixture upon irradiation (Figure 3 E).[^5^, ^24^]

**Figure 3:**
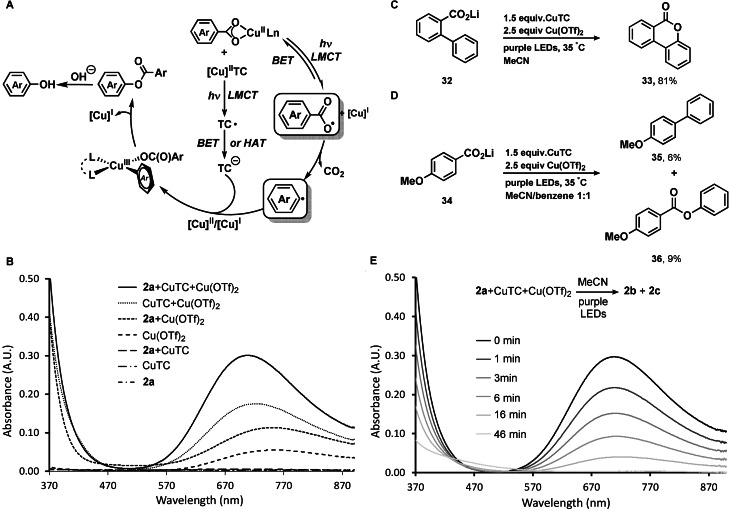
A) Proposed reaction mechanism. B) UV/Vis absorption spectra of reaction components C) Radical cyclisation experiment. D) Radical trapping experiment. E) UV/Vis spectral changes observed upon photolysis of a mixture of **2 a** (1 mM), Cu(OTf)_2_ (2.5 mM) and CuTC (1.5 mM) in MeCN under purple LEDs irradiation (0–46 min).

Radical decarboxylative carbometalation enabled by LMCT in copper benzoates provides the first decarboxylative hydroxylation of benzoic acids at 35 °C, a temperature about 100 °C below conventional decarboxylation of aryl carboxylic acids. Expansion of LMCT‐based decarboxylative carbometalation to enable C−O bond formation beyond the initially discovered C‐F bond formation establishes the utility and power of the new concept for previously inaccessible decarboxylative functionalizations.

## Conflict of interest

The authors declare no conflict of interest.

## Supporting information

As a service to our authors and readers, this journal provides supporting information supplied by the authors. Such materials are peer reviewed and may be re‐organized for online delivery, but are not copy‐edited or typeset. Technical support issues arising from supporting information (other than missing files) should be addressed to the authors.

Supporting Information
