# Supplementary material for: Decarboxylative Hydroxylation of Benzoic Acids
Source: Angew Chem Int Ed Engl. 2021 Oct 4;60(45):24012–7. doi: 10.1002/anie.202108971 (PMC8596882; doi:10.1002/anie.202108971)
Supplement: Supplementary file 1 — Supporting Information [file ANIE-60-24012-s001.pdf]

Angewandte Chemie
Eine Zeitschrift der Gesellschaft Deutscher Chemiker

Supporting Information

Decarboxylative Hydroxylation of Benzoic Acids

*Wanqi Su, Peng Xu, and Tobias Ritter**

anie_202108971_sm_miscellaneous_information.pdf

TABLE OF CONTENTS

TABLE OF CONTENTS ... 1

MATERIALS AND METHODS ... 6

EXPERIMENTAL DATA ... 7

General procedure of preparing lithium aryl carboxylate ... 7

General procedure of aromatic decarboxylative hydroxylation ... 7

Reaction condition optimization ... 8

Table S1 O-nucleophiles ... 8

Table S2 Cu(II) sources ... 11

Table S3 Counter ions ... 11

Table S4 Control experiments ... 12

Identification of the decarboxylation products ... 13

CuTC as coupling partner ... 13

4-Methoxybenzoate as coupling partner ... 15

Lithium thiophene-2-carboxylate as substrate ... 17

Decarboxylative hydroxylation of benzoic acids to phenols ... 19

Ataluren derivative **1** ... 19

1 mmol Scale synthesis of Ataluren derivative **1** ... 20

4-Fluorophenol (**2**) ... 21

One-pot synthesis of 4-fluorophenol (**2**) ... 22

4-Cyanophenol (**3**) ... 23

4-(Trifluoromethyl)phenol (**4**) ... 24

2,4-Bis(trifluoromethyl)phenol (**5**) ... 25

2-(Trifluoromethyl)pyridin-4-ol (**6**) ... 27

4-(5-Methyl-1,2,4-oxadiazol-3-yl)phenol (**7**) ... 28

6-Hydroxy-2-naphthonitrile (**8**) ... 29

(+)-Menthol derivative **9** ... 30

3-Hydroxybenzaldehyde (**10**) ... 31

4-Methylphenol (**11**) ... 32

4-Methoxyphenol (**12**) ... 33

3,5-Dimethylphenol (**13**) ... 35

2,6-Dichloropyridin-4-ol (**14**) ... 37

2-Hydroxyquinoxaline (**15**) ... 38

4-Methyl-3-(morpholinosulfonyl)phenol (**16**) ... 39

Probenicid derivative **17** ... 40

4-Acetylphenol (**18**) ... 41
4-Sulfonamidephenol (**19**) ... 42
4-(Methylsulfonyl)phenol (**20**) ... 43
2-Naphthol (**21**) ... 44
(4-Hydroxyphenyl)(morpholino)methanone (**22**) ... 45
4-(*trans*-4-Pentylcyclohexyl)phenol (**23**) ... 46
Celecoxib derivative **24** ... 47
4-Bromophenol (**25**) ... 48
4-Nitrophenol (**26**) ... 49
2-Fluorophenol (**27**) ... 50
6-Hydroxy-1-tetralone (**28**) ... 51
2,2'-(5-Hydroxy-1,3-phenylene)bis(2-methylpropanenitrile) (**29**) ... 52
4-Phenylphenol (**30**) ... 53
Triclosan derivative **31** ... 54
Reaction performed outside of a glovebox by using Schlenk techniques ... 55
Failed substrates ... 56

Preparation of starting material ... 56

Triclosan-derived benzoic acid **S31** ... 56

Mechanistic Studies ... 57

UV-vis Absorption Spectrum ... 57
Photolysis of the mixture of **2a**, $Cu(OTf)_2$, and CuTC ... 58
Radical cyclisation experiment ... 59
Radical trapping experiments ... 60

SPECTROSCOPIC DATA ... 62

^{1}H NMR of ataluren derivative **1** ... 62

^{13}C NMR of ataluren derivative **1** ... 63

^{19}F NMR of ataluren derivative **1** ... 64

^{1}H NMR of 4-cyanophenol (**3**) ... 65

^{13}C NMR of 4-cyanophenol (**3**) ... 66

^{1}H NMR of 2-(trifluoromethyl)pyridin-4-ol (**6**) ... 67

^{13}C NMR of 2-(trifluoromethyl)pyridin-4-ol (**6**) ... 68

^{19}F NMR of 2-(trifluoromethyl)pyridin-4-ol (**6**) ... 69

^{1}H NMR of 4-(5-methyl-1,2,4-oxadiazol-3-yl)phenol (**7**) ... 70

^{13}C NMR of 4-(5-methyl-1,2,4-oxadiazol-3-yl)phenol (**7**)....71

^{1}H NMR of 6-hydroxy-2-naphthonitrile (**8**)....72

^{13}C NMR of 6-hydroxy-2-naphthonitrile (**8**)....73

^{1}H NMR of (+)-menthol derivative **9**....74

^{13}C NMR of (+)-menthol derivative **9**....75

^{1}H NMR of 3-hydroxybenzaldehyde (**10**)....76

^{13}C NMR of 3-hydroxybenzaldehyde (**10**)....77

^{1}H NMR of 2,6-dichloropyridin-4-ol (**14**)....78

^{13}C NMR of 2,6-dichloropyridin-4-ol (**14**)....79

^{1}H NMR of 2-hydroxyquinoxaline (**15**)....80

^{13}C NMR of 2-hydroxyquinoxaline (**15**)....81

^{1}H NMR of 4-methyl-3-(morpholinosulfonyl)phenol (**16**)....82

^{13}C NMR of 4-methyl-3-(morpholinosulfonyl)phenol (**16**)....83

^{1}H NMR of probenicid derivative **17**....84

^{13}C NMR of probenicid derivative **17**....85

^{1}H NMR of 4-acetylphenol (**18**)....86

^{13}C NMR of 4-acetylphenol (**18**)....87

^{1}H NMR of 4-sulfonamidephenol (**19**)....88

^{13}C NMR of 4-sulfonamidephenol (**19**)....89

^{1}H NMR of 4-(methylsulfonyl)phenol (**20**)....90

^{13}C NMR of 4-(methylsulfonyl)phenol (**20**)....91

^{1}H NMR of 2-naphthol (**21**)....92

^{13}C NMR of 2-naphthol (**21**)....93

^{1}H NMR of (4-hydroxyphenyl)(morpholino)methanone (**22**)....94

^{13}C NMR of 4-(morpholinocarbonyl)phenone (**22**)....95

^{1}H NMR of 4-(*trans*-4-pentylcyclohexyl)phenol (**23**)....96

^{13}C NMR of 4-(*trans*-4-pentylcyclohexyl)phenol (**23**)....97

^{1}H NMR of celecoxib derivative **24**....98

^{13}C NMR of celecoxib derivative **24**....99

^{19}F NMR of celecoxib derivative **24** 100

^{1}H NMR of 4-bromophenol (**25**) 101

^{13}C NMR of 4-bromophenol (**25**) 102

^{1}H NMR of 4-nitrophenol (**26**) 103

^{13}C NMR of 4-nitrophenol (**26**) 104

^{1}H NMR of 6-hydroxy-1-tetralone (**28**) 105

^{13}C NMR of 6-hydroxy-1-tetralone (**28**) 106

^{1}H NMR of 2,2'-(5-Hydroxy-1,3-phenylene)bis(2-methylpropanenitrile) (**29**) 107

^{13}C NMR of 2,2'-(5-Hydroxy-1,3-phenylene)bis(2-methylpropanenitrile) (**29**) 108

^{1}H NMR of 4-phenylphenol (**30**) 109

^{13}C NMR of 4-phenylphenol (**30**) 110

^{1}H NMR of triclosan derivative **31** 111

^{13}C NMR of triclosan derivative **31** 112

^{1}H NMR of triclosan-derived benzoic acid **S31** 113

^{13}C NMR of triclosan-derived benzoic acid **S31** 114

^{1}H NMR of 6H-benzo[c]chromen-6-one (**33**) 115

^{1}H NMR of 4-methoxy-1,1'-biphenyl (**35**) 117

^{13}C NMR of 4-methoxy-1,1'-biphenyl (**35**) 118

^{1}H NMR of phenyl 4-methoxybenzoate (**36**) 119

^{13}C NMR of phenyl 4-methoxybenzoate (**36**) 120

^{1}H NMR of 4-fluorophenyl thiophene-2-carboxylate (**2b**) 121

^{13}C NMR of 4-fluorophenyl thiophene-2-carboxylate (**2b**) 122

^{19}F NMR of 4-fluorophenyl thiophene-2-carboxylate (**2b**) 123

^{1}H NMR of 4-fluorophenyl 4-fluorobenzoate (**2c**) 124

^{13}C NMR of 4-fluorophenyl 4-fluorobenzoate (**2c**) 125

^{19}F NMR of 4-fluorophenyl 4-fluorobenzoate (**2c**) 126

^{1}H NMR of 4-fluorophenyl 4-methoxybenzoate (**2e**) 127

^{13}C NMR of 4-fluorophenyl 4-methoxybenzoate (**2e**) 128

^{19}F NMR of 4-fluorophenyl 4-methoxybenzoate (**2e**) 129

^{1}H NMR of 4-methoxyphenyl 4-fluorobenzoate (**2f**) 130

^{13}C NMR of4-methoxyphenyl 4-fluorobenzoate (**2f**) 131

^{19}F NMR of 4-methoxyphenyl 4-fluorobenzoate (**2f**) 132

^{1}H NMR of 4-methoxyphenyl 4-methoxybenzoate (**2g**) 133

^{13}C NMR of 4-methoxyphenyl 4-methoxybenzoate (**2g**) 134

REFERENCES 135

MATERIALS AND METHODS

All air- and moisture-insensitive reactions were carried out under an ambient atmosphere and monitored by thin-layer chromatography (TLC). All air- and moisture-sensitive manipulations were performed using standard Schlenk and glove-box techniques under an atmosphere of nitrogen. Concentration under reduced pressure was performed by rotary evaporation at 25–40 °C at an appropriate pressure, unless otherwise stated. Purified compounds were further dried under high vacuum (0.008–0.5 Torr). Yields refer to purified and spectroscopically pure compounds, unless otherwise stated.

Solvents

Anhydrous acetonitrile, DCM, THF, and toluene were obtained from Phoenix Solvent Drying Systems. All deuterated solvents were purchased from Euriso-Top.

Chromatography

Thin layer chromatography (TLC) was performed using EMD TLC silica gel 60 F254 plates pre-coated with 250 μm thickness silica gel and visualized by fluorescence quenching under UV light or phosphomolybdic acid stain. Preparative HPLC separation was executed on Shimadzu Prominence Preparative HPLC system. Preparative TLC was performed using pre-coated TLC plates SIL G-100 UV_{254} (Layer: 1.00 mm silica gel 60 with fluorescent indicator UV_{254}).

Spectroscopy and Instruments

NMR spectra were recorded on a Bruker Ascend™ 500 spectrometer operating at 500 MHz, 471 MHz, and 126 MHz, for ^{1}H, ^{19}F, and ^{13}C acquisitions, respectively; or a Bruker UltraShield™ 300 spectrometer operating at 300 MHz, 282 MHz, and 75 MHz, for ^{1}H, ^{19}F, and ^{13}C acquisitions, respectively; or a Bruker AV600 spectrometer operating at 600 MHz and150 MHz for ^{1}H and ^{13}C acquisitions; respectively. Chemical shifts are reported in ppm with the solvent residual peak as the internal standard. For ^{1}H NMR: $CDCl_3$, δ 7.26; CD_3CN, δ 1.94; CD_2Cl_2, δ 5.32, THF-d_8, δ 1.72, 3.58; CD_3OD-d_4, δ 3.31, $(CD_3)_2SO$, δ 2.50. For ^{13}C NMR: $CDCl_3$, δ 77.16; CD_3CN, δ 1.32; CD_2Cl_2, δ 53.84, THF-d_8, δ 25.31, 67.21, CD_3OD-d_4, δ 49.00.[1] Data is reported as follows: s = singlet, d = doublet, t = triplet, q = quartet, m = multiplet, brs = broad singlet; coupling constants in Hz; integration.

Starting materials

All substrates and materials were used as received from commercial suppliers, unless otherwise stated. $Cu(OTf)_2$ purchased from Fluorochem was stored in a glovebox and used as received. Copper(I) thiophene-2-carboxylate, abbreviated as CuTC, purchased from Chempur Feinchemikalien was stored on the bench and used as received. (+)Menthol-derived benzoic acid (**S9**) and carboxycelecoxib (**S24**) were synthesized according to the literature.[2,3]

EXPERIMENTAL DATA

General procedure of preparing lithium aryl carboxylate

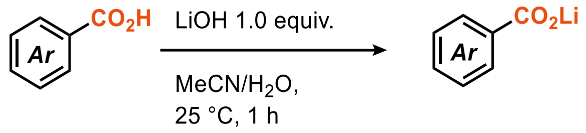

Under an ambient atmosphere, a 20 mL borosilicate vial equipped with a magnetic stir bar was charged with aryl carboxylic acid (0.30 mmol, 1.0 equiv.), lithium hydroxide (7.2 mg, 0.30 mmol, 1.0 equiv), and MeCN/H_2O (3.0 mL, v/v = 1:1, c = 0.10 M), which resulted in a solution. The reaction mixture was stirred at 25 °C for 1 h. The solvent was evaporated under reduced pressure to afford the lithium aryl carboxylate as a solid. The lithium aryl carboxylate was dried under high vacuum (0.008–0.5 Torr) at 45 °C. Dried lithium aryl carboxylate can be used directly without purification in the subsequent transformation, or stored on the bench for at least six months for later use.

Note: For some substrates, the v/v ratio and the final volume of MeCN/H_2O were adjusted by adding additional MeCN or H_2O according to the solubility of the aryl carboxylic acid and the resulting lithium salt to generate a solution.

General procedure of aromatic decarboxylative hydroxylation

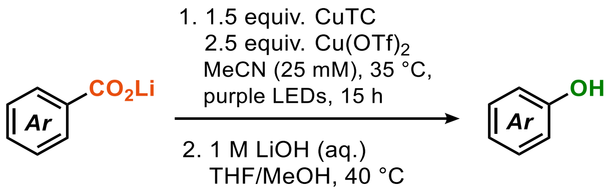

Under an ambient atmosphere, a 10 mL borosilicate vial equipped with a magnetic stir bar was charged with lithium aryl carboxylate (0.200 mmol, 1.00 equiv.) and copper (I) thiophene-2-carboxylate (0.300 mmol, 1.50 equiv.). The vial was transferred into an anhydrous, N_2-filled glovebox. Under nitrogen atmosphere, $Cu(OTf)_2$ (0.500 mmol, 2.50 equiv.), and anhydrous MeCN (8.0 mL, c = 25 mM) were then added into the vial, resulting in a green suspension. The vial was sealed with a Teflon cap and placed 5 cm away from two purple LEDs (Kessil PR160-390 nm LEDs). The reaction mixture was stirred at a speed of 1200 rpm (Note: Fast stirring is essential due to the heterogeneity of the reaction mixture.) and irradiated for 16 h while maintaining the temperature at approximately 35 °C through cooling with a fan. Subsequently, the reaction mixture was transferred into a 50 mL round-bottom flask and evaporated to dryness. The residue was dissolved in EtOAc (40 mL) and filtered through a short pad of silica using EtOAc (50 mL) as the eluent to remove the majority of the copper salts. The filtrate was collected and concentrated under reduced pressure into a 20 mL borosilicate vial. To the 20 mL vial containing phenolic esters, a magnetic stir bar, THF (1.5 mL), MeOH (1.5 mL) and aqueous LiOH solution (1.0 M, 0.60 mL) were added. The reaction mixture was stirred at 40 °C and monitored by TLC until the phenolic esters were fully consumed (Note: The hydrolysis normally took roughly 2 hours). The

reaction mixture was acidified by addition of 1.0 M aqueous HCl to pH = 3–5 (roughly 0.70 mL). The solution is concentrated to about 10% or less of its volume under reduced pressure. The remaining suspension was dissolved in EtOAc (20 mL) and transferred into a separatory funnel containing 5 mL saturated aqueous $NaHCO_3$ and 5 mL H_2O. The organic layer was collected, and the aqueous layer was further extracted with EtOAc (3 × ca. 20 mL). The combined organic layers were dried over Na_2SO_4, filtered, and the filtrate was concentrated under reduced pressure to dryness. The residue was purified by chromatography on silica gel to afford the corresponding phenol.

Note: The reactions can be performed outside of a glovebox by using Schlenk techniques, which gives the same yield (see **Figure S21**). Acid deprotonation by lithium carbonate to form copper carboxylates, decarboxylative oxygenation, and subsequent hydrolysis of the aryl ester can be carried out in the same pot to afford comparable yield (see **Figure S7**).

Reaction condition optimization

Table S1 O-nucleophiles

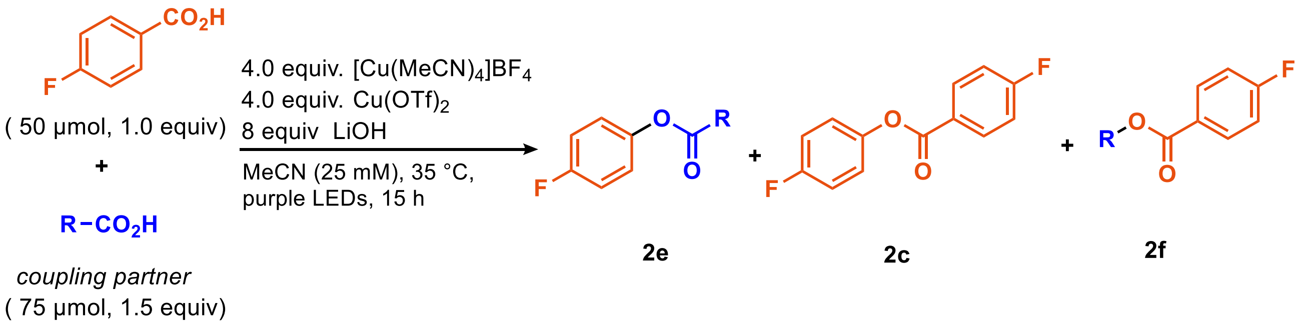

Coupling partner	Yield (**2e**/**2c**/**2f**, %)[a]	Ratio [**2e**/(**2c*0.5**)	Oxydecarboxylation yield (**2e**+**2c*0.5**, %)[a]
**1.5 equiv. CuTC[b]**	**51/40/0**	**2.5/1**	**71**
2.0 equiv. CuTC[b]	52/34/0	2.5/1	69
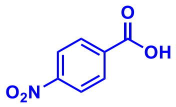	21/44/12	1/1	43
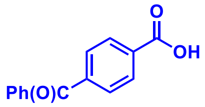	31/30/26	2/1	46

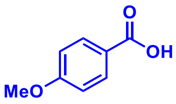	46/34/19	2.7/1	63
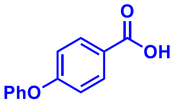	29/50/10	1.2/1	54
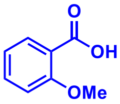	37/56/17	1.3/1	65
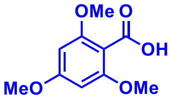	0/0/0	/	0
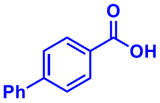	27/60/17	0.9/1	57
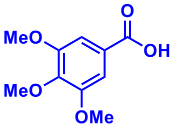	21/72/0	0.6/1	57
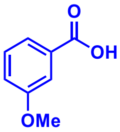	25/38/23	1.3/1	44
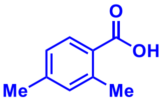	17/50/15	0.7/1	42
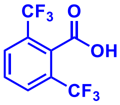	0/0/0	/	0
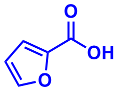	45/48/0	1.9/1	69

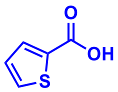	30/26/0	2.3/1	43
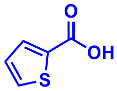 *c*	34/26/0	2.6/1	47
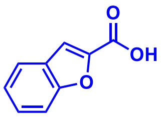	10/28/0	0.7/1	24
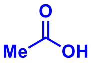	8/80/0	0.2/1	44
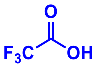	8/56/0	0.3/1	36
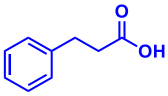	14/72/0	0.4/1	50
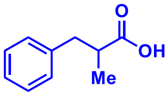	12/31/0	0.8/1	27 (56% SM left)
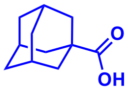	5/63/0	0.2/1	36 (24% SM left)

[a]Yield was determined by ^{19}F NMR using 2-fluorotoluene (1.0 equiv.) as an internal standard.

[b]lithium 2-fluorobenzoate (50 μmol, 1.0 equiv) as starting material. reaction condition: 2.5 equiv $Cu(OTf)_2$, MeCN (25 mM), 35 °C, purple LEDs, 15 h.

[c]lithium 2-fluorobenzoate (50 μmol, 1.0 equiv) as starting material.

Table S2 Cu(II) sources

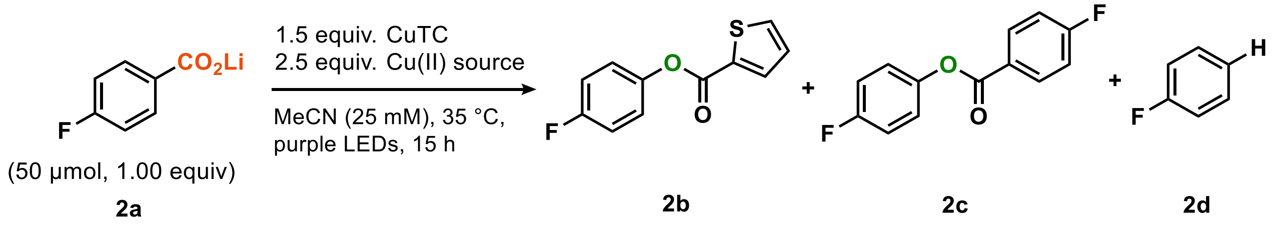

Cu(II) source	Yield (**2b**/**2c**/**2d**, %)[a]	Oxydecarboxylation yield (**2b**+**2c*0.5**, %)[a]
**2.5 equiv $Cu(OTf)_2$**	**51/40/5**	**71**
2.0 equiv $Cu(OTf)_2$	25/42/5	46
1.5 equiv $Cu(OTf)_2$	9/26/4	22
1.1 equiv $Cu(OTf)_2$	0/0/0	0
$Cu(NO_3)_2{\cdot}nH_2O$	0/0/0	0
$Cu(ClO)_4{\cdot}6H_2O$	18/30/2	33
$Cu(BF_4)_2{\cdot}nH_2O$	22/34/3	39

[a] ^{19}F NMR yield with 2-fluorotoluene (1.0 equiv.) as an internal standard.

Table S3 Counter ions

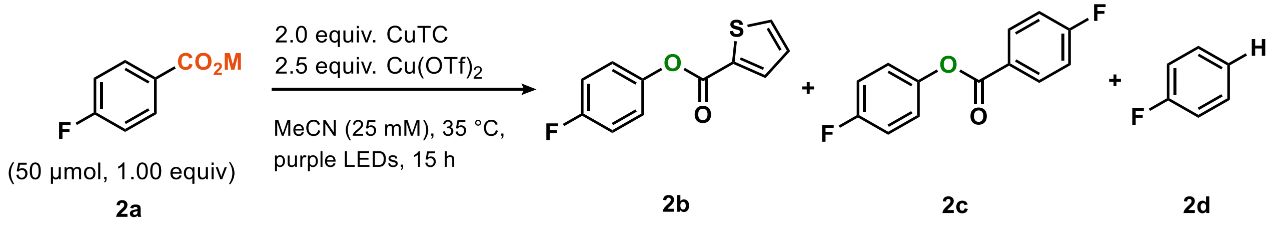

Counterion M^+	Yield (**2b**/**2c**/**2d**, %)[a]	Oxydecarboxylation yield (**2b**+**2c*0.5**, %)[a]
**Li^+**	**52/34/7**	**69**

K^+	43/32/4	59
Na^+	46/34/3	63
Cs^+	48/44/5	70
TBA^+	37/24/6	49

[a] ^{19}F NMR yield with 2-fluorotoluene (1.0 equiv.) as an internal standard.

Table S4 Control experiments

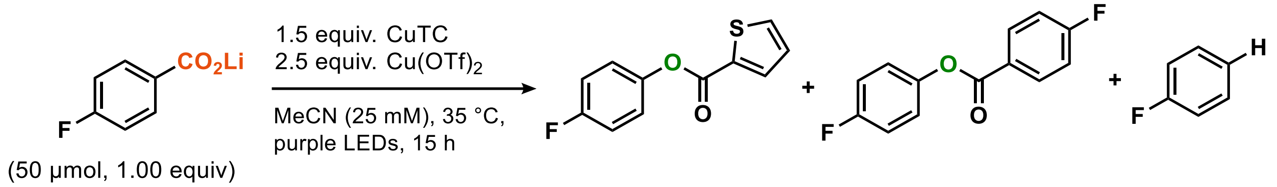

2a 2b 2c 2d

Deviation	Yield (**2b**/**2c**/**2d**, %)[a]	Oxydecarboxylation yield (**2b**+**2c*0.5**, %)[a]
no deviation	**51/40/5**	**71**
0.20 mmol scale	54/40/6	74
no irradiation	0/0/0	0
blue LEDs instead of purple LEDs	14/34/4	31
white CFL instead of purple LEDs	6/16/2	14
100 °C heating instead of irradination	0/0/0	0
lithium benzoate generated *in situ* by adding 4-fluorobenzoic acid and 2.0 equiv. Li_2CO_3	54/34/5	71
lithium benzoate generated *in situ* by adding 4-fluorobenzoic acid and 2.0 equiv. Li_2CO_3 (0.20 mmol scale)	42/46/6	64
no CuTC	0/60/13	30
no $Cu(OTf)_2$	0/0/0	0

1.5 equiv. LiTC instead of CuTC	35/30/12	50
5.0 equiv. H_2O as additive	37/44/5	59
2.0 equiv. TEMPO as additive	36/56/6	64
air atmosphere instead of nitrogen atmosphere	0/0/0	0

[a] ^{19}F NMR yield with 2-fluorotoluene (1.0 equiv.) as an internal standard.

Identification of the decarboxylation products

CuTC as coupling partner

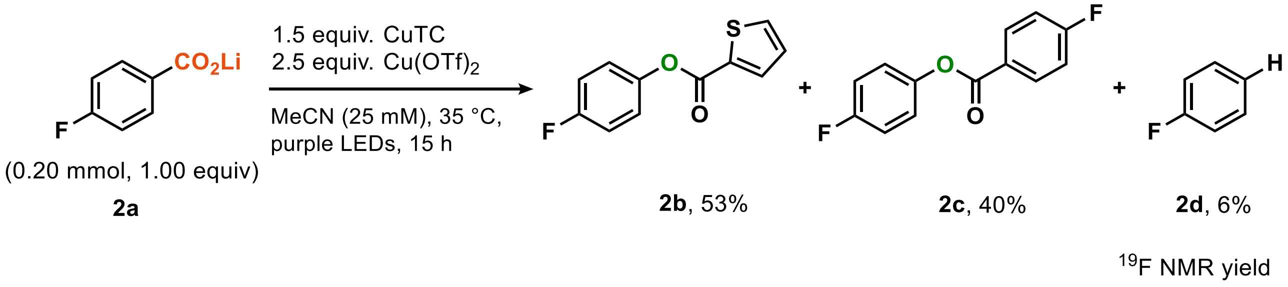

Scheme S1. CuTC as coupling partner

Under an ambient atmosphere, a 10 mL borosilicate vial equipped with a magnetic stir bar was charged with lithium 4-fluorobenzoate (29.2 mg, 0.200 mmol, 1.00 equiv.) and copper (I) thiophene-2-carboxylate (57.2 mg, 0.300 mmol, 1.50 equiv.). The vial was transferred into an anhydrous, N_2-filled glovebox. Under nitrogen atmosphere, $Cu(OTf)_2$ (181 mg, 0.500 mmol, 2.50 equiv.), and anhydrous MeCN (8.0 mL, c = 25 mM) were then added into the vial, resulting in a green suspension. The vial was sealed with a Teflon cap and placed 5 cm away from two purple LEDs (Kessil PR160-390 nm LEDs). The reaction mixture was stirred at a speed of 1200 rpm (Note: Fast stirring is essential due to the heterogeneity of the reaction mixture.) and irradiated for 16 h while maintaining the temperature at approximately 35 °C through cooling with a fan. 2-fluorotoluene (22.0 mg, 22.0 µL, 0.200 mmol, 1.00 equiv.) was added as an internal standard. The reaction mixture was diluted with CD_3CN, and the ^{19}F NMR spectrum was measured. The yields of 4-fluorophenyl thiophene-2-carboxylate (**2b**, 53%, δ –118.42 (m) ppm), 4-fluorophenyl 4-fluorobenzoate (**2c**, 40%, δ –118.60, 106.41 (m) ppm) and 4-fluorobenzene (**2d**, 6%, δ –114.82 (m) ppm) were determined by ^{19}F NMR integration relative to the internal standard (δ –119.37 (m) ppm), respectively. The NMR sample was recycled and the reaction mixture was transferred into a 50 mL round-bottom flask and evaporated to dryness. The residue was purified by chromatography on silica gel (pentane/EtOAc 4:1, v/v) to afford 4-fluorophenyl thiophene-2-carboxylate (**2b**) (9.0 mg, 40 µmol) as a colorless solid and 4-fluorophenyl 4-fluorobenzoate (**2c**) (6.0 mg, 25 µmol) also as a colorless solid

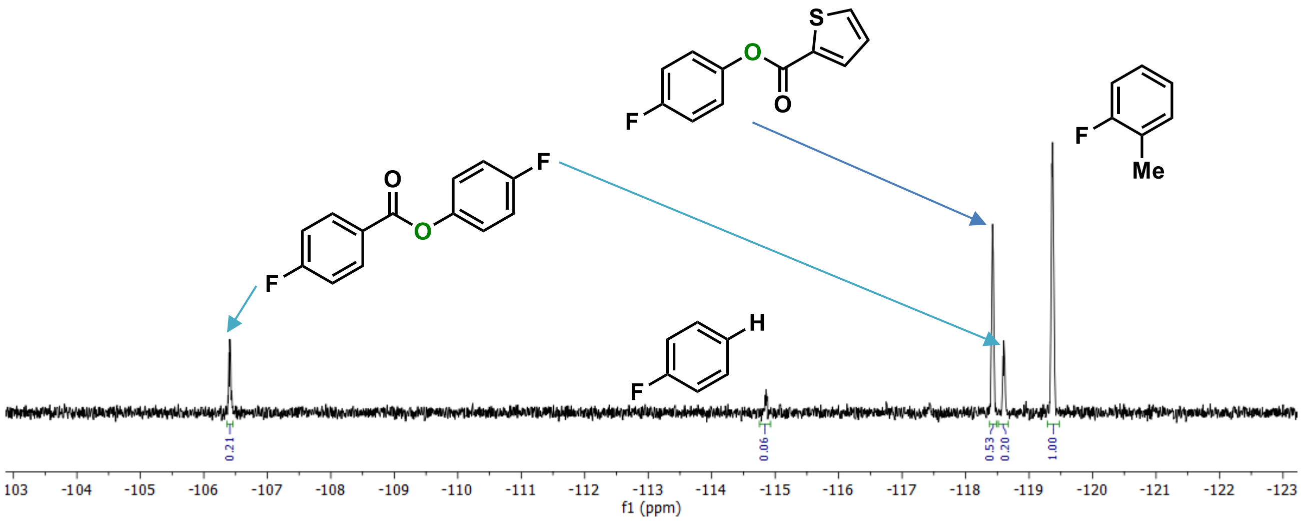

Figure S1.^{19}F NMR of resulting mixture of **2b**, **2c** and **2d**.

4-fluorophenyl thiophene-2-carboxylate (**2b**)

R*f* = 0.20 (pentane/EtOAc = 4/1, v/v).

NMR Spectroscopy:

^{1}H NMR (500 MHz, $CDCl_3$, 298 K, δ): 7.91 (dd, *J* = 3.8, 1.3 Hz, 1H), 7.60 (dd, *J* = 5.0, 1.3 Hz, 1H), 7.16–7.07 (m, 3H), 7.07–6.99 (m, 2H) ppm.

^{13}C NMR (126 MHz, $CDCl_3$, 298 K, δ): 160.6, 160.4 (d, *J* = 244.8 Hz) 146.4 (d, *J* = 3.0 Hz), 134.8, 133.7, 132.6, 128.1, 123.1 (d, *J* = 8.4 Hz), 116.1 (d, *J* = 23.3 Hz) ppm.

^{19}F NMR (471 MHz, $CDCl_3$, 298 K, δ): –116.74 ppm.

HRMS-EI (m/z) calculated for $C_{11}H_7O_2SF^+$ $[M]^+$, 222.0145; found, 222.0144; deviation: 0.5 ppm.

4-fluorophenyl 4-fluorobenzoate (**2c**)

R*f* = 0.25 (Pentane/EtOAc = 4/1, v/v).

NMR Spectroscopy:

^{1}H NMR (500 MHz, $CDCl_3$, 298 K, δ): 8.21 (dd, *J* = 8.9, 5.3 Hz, 2H), 7.25–7.15 (m, 4H), 7.11 (dd, *J* = 9.2, 8.0 Hz, 2H) ppm.

^{13}C NMR (126 MHz, $CDCl_3$, 298 K, δ): 166.2 (d, *J* = 255.6 Hz), 164.2, 160.4 (d, *J* = 244.4 Hz), 146.6 (d, *J* = 3.0 Hz), 132.8 (d, *J* = 9.5 Hz), 125.6 (d, *J* = 3.0 Hz), 123.1 (d, *J* = 8.8 Hz), 116.2 (d, *J* = 23.3 Hz), 115.9 (d, *J* = 22.1 Hz) ppm.

^{19}F NMR (471 MHz, $CDCl_3$, 298 K, δ): –104.13, –116.78 ppm.

HRMS-ESI (m/z) calculated for $C_{13}H_8O_2NaF^+$ $[M+Na]^+$, 257.0384; found, 257.0386; deviation: –0.6 ppm.

4-Methoxybenzoate as coupling partner

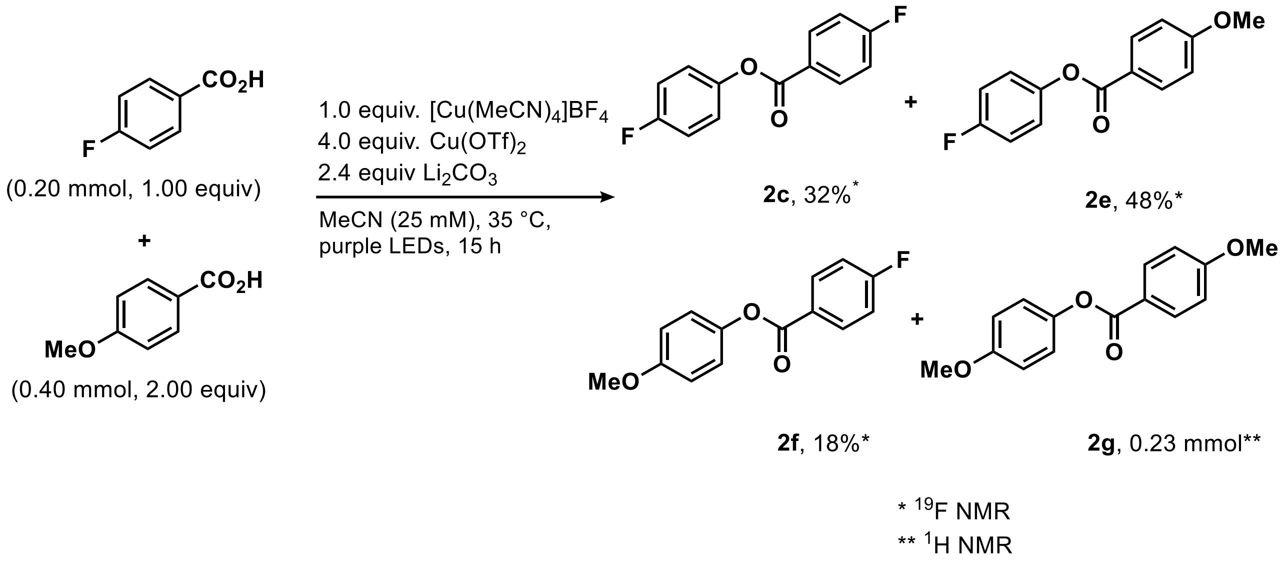

Scheme S2. 4-methoxybenzoate as coupling partner

Under an ambient atmosphere, a 10 mL borosilicate vial equipped with a magnetic stir bar was charged with 4-fluorobenzoic acid (28.0 mg, 0.200 mmol, 1.00 equiv.), 4-methoxybenzoic acid (60.8 mg, 0.400 mmol, 2.00 equiv.) and lithium carbonate (35.4 mg, 0.480 mmol, 2.40 equiv.). The vial was transferred into an anhydrous, N_2-filled glovebox. Under nitrogen atmosphere, $Cu(OTf)_2$ (289 mg, 0.800 mmol, 4.00 equiv.), $[Cu(MeCN)_4]BF_4$ (62.9 mg, 0.200 mmol, 1.00 equiv.) and anhydrous MeCN (8.0 mL, *c* = 25 mM) were then added into the vial, resulting in a green suspension. The vial was sealed with a Teflon cap and placed 5 cm away from two purple LEDs (Kessil PR160-390 nm LEDs). The reaction mixture was stirred at a speed of 1200 rpm (Note: Fast stirring is essential due to the heterogeneity of the reaction mixture.) and irradiated for 16 h while maintaining the temperature at approximately 35 °C through cooling with a fan. Subsequently, 2-fluorotoluene (22.0 mg, 22.0 µL, 0.200 mmol, 1.00 equiv.) and dibromomethane (69.5 mg, 28.1 µL, 0.400 mmol, 2.00 equiv.) were added as internal standards for ^{19}F and ^{1}H NMR measurements respectively. The reaction mixture was diluted with CD_3CN, and the ^{19}F NMR spectrum and ^{1}H NMR spectrum were measured. The yields of 4-fluorophenyl 4-fluorobenzoate (**2c**, 32%, δ –118.75, 106.54 (m) ppm), 4-fluorophenyl 4-methoxybenzoate (**2e**, 48%, δ –119.10 (m) ppm) and 4-methoxyphenyl 4-fluorobenzoate (**2f**, 18%, δ –106.88 (m) ppm) were determined by ^{19}F NMR integration relative to the internal standard (δ –119.53 (m) ppm), respectively. The amount of 4-methoxyphenyl 4-methoxybenzoate (**2g,** 0.230 mmol, 1.16 equiv. δ 3.87 (s), 3.79 (s) ppm) was determined by ^{1}H integration relative to the internal standard (δ 5.08 (s) ppm)The NMR sample was recycled and the reaction mixture was transferred into a 50 mL round-bottom flask and evaporated to dryness. The residue was purified by chromatography on silica gel (pentane/DCM 1:1, v/v) to afford 4-fluorophenyl 4-methoxybenzoate (**2e**) (8.4 mg, 34 µmol) as a colorless solid and 4-methoxyphenyl 4-methoxybenzoate (**2g**) (17 mg, 68 µmol) as a colorless solid. Fractions containing **2e** and **2f** were collected and concentrated. The residue was further purified by preparation TLC (pentane/DCM 1:1 v/v) to afford 4-methoxyphenyl 4-fluorobenzoate (**2f**) (2.3 mg, 9.3 µmol) as a pale yellow liquid.

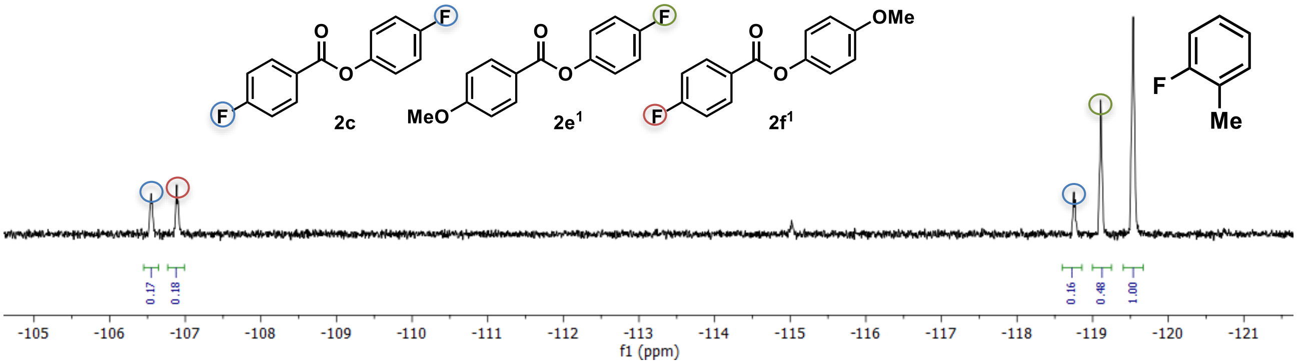

Figure S2.^{19}F NMR of resulting mixture of **2c**, **2e[1]** and **2f[1]**.

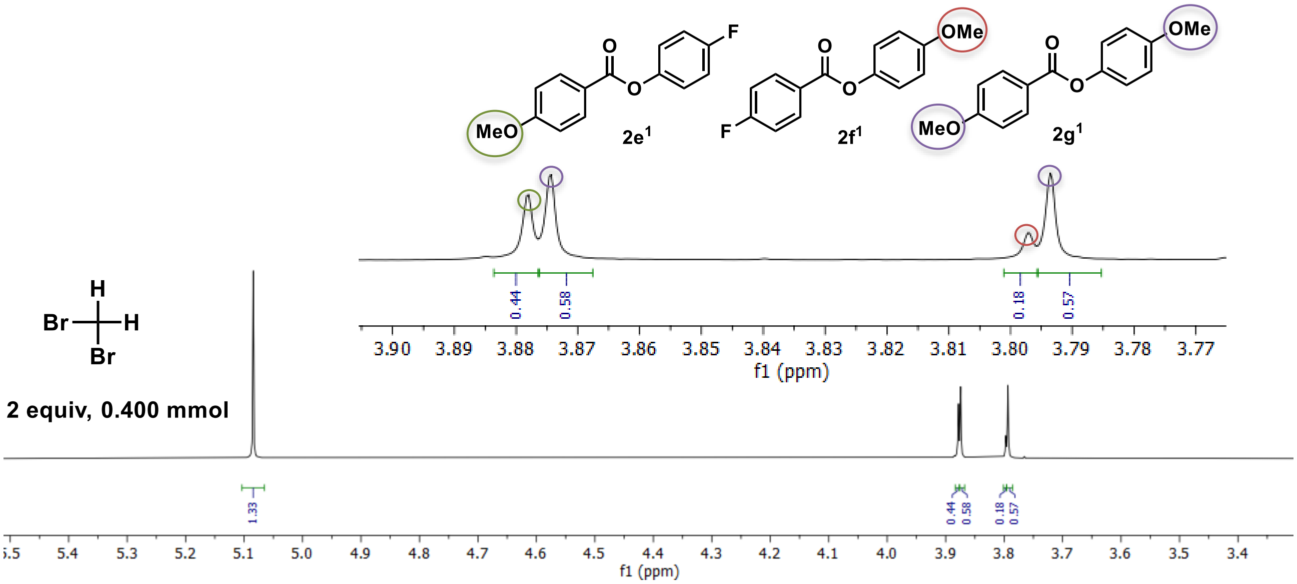

Figure S3.^{1}H NMR of resulting mixture of **2e**, **2f**.and **2g**

4-fluorophenyl 4-methoxybenzoate (**2e**)

R*f* = 0.40 (Pentane/DCM = 1/1, v/v).

NMR Spectroscopy:

^{1}H NMR (500 MHz, $CDCl_3$, 298 K, δ): 8.07 (d, *J* = 8.9 Hz, 2H), 7.13–7.06 (m, 2H), 7.03 (dd, *J* = 9.1, 8.1 Hz, 2H), 6.91 (d, *J* = 8.9 Hz, 2H), 3.83 (s, 3H) ppm.

^{13}C NMR (126 MHz, $CDCl_3$, 298 K, δ): 164.9, 164.0, 160.2 (d, *J* = 244.2 Hz), 146.9 (d, *J* = 2.9 Hz), 132.3, 123.2 (d, *J* = 8.3 Hz), 121.6, 116.1 (d, *J* = 23.7 Hz), 113.9, 55.5 ppm.

^{19}F NMR (471 MHz, $CDCl_3$, 298 K, δ): –117.28 ppm.

HRMS-ESI (m/z) calculated for $C_{14}H_{11}O_3NaF^+$ $[M+Na]^+$, 269.0584; found, 269.0585; deviation: –0.2 ppm.

4-methoxyphenyl 4-fluorobenzoate (**2f**)

R*f* = 0.35 (Pentane/DCM = 1/1, v/v).

NMR Spectroscopy:

^{1}H NMR (500 MHz, $CDCl_3$, 298 K, δ): 8.22 (dd, *J* = 8.9, 5.4 Hz, 2H), 7.18 (t, *J* = 8.7 Hz, 2H), 7.12 (d, *J* = 9.1 Hz, 2H), 6.94 (d, *J* = 9.0 Hz, 2H), 3.83 (s, 3H).

^{13}C NMR (126 MHz, $CDCl_3$, 298 K, δ): 166.3 (d, *J* = 254.9 Hz), 164.7, 157.5, 144.4, 132.9 (d, *J* = 9.3 Hz), 126.0 (d, *J* = 3.1 Hz), 122.5, 115.9 (d, *J* = 22.1 Hz), 114.7, 55.8 ppm.

^{19}F NMR (471 MHz, $CDCl_3$, 298 K, δ): –104.64.

HRMS-ESI (m/z) calculated for $C_{14}H_{11}O_3NaF^+$ $[M+Na]^+$, 269.0584; found, 269.0586; deviation: –0.7 ppm.

4-methoxyphenyl 4-methoxybenzoate (**2g**)

R*f* = 0.10 (Pentane/DCM = 1/1, v/v).

NMR Spectroscopy:

^{1}H NMR (600 MHz, $CDCl_3$, 298 K, δ): 8.15 (d, *J* = 9.1 Hz, 2H), 7.12 (d, *J* = 9.1 Hz, 2H), 6.98 (d, *J* = 9.0 Hz, 2H), 6.93 (d, *J* = 9.1 Hz, 2H), 3.89 (s, 3H), 3.82 (s, 3H) ppm.

^{13}C NMR (151 MHz, $CDCl_3$, 298 K, δ): 165.4, 164.0, 157.3, 144.7, 132.4, 122.7, 122.1, 114.6, 113.9, 55.7, 55.6 ppm.

HRMS-ESI (m/z) calculated for $C_{15}H_{14}O_4Na^+$ $[M+Na]^+$, 281.0784; found, 281.0785; deviation: –0.4 ppm.

Lithium thiophene-2-carboxylate as substrate

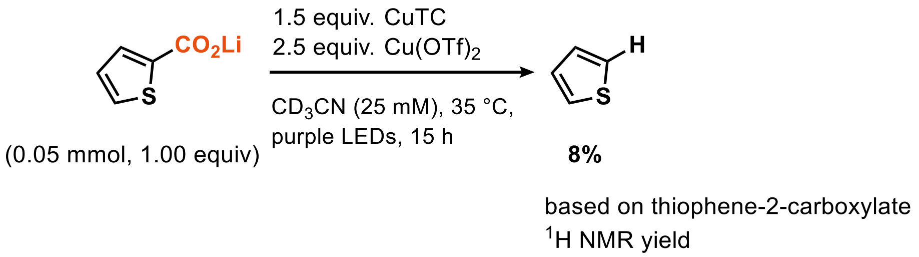

Scheme S3. lithium thiophene-2-carboxylate as substrate

Under an ambient atmosphere, a 4 mL borosilicate vial equipped with a magnetic stir bar was charged with 4-lithium thiophene-2-carboxylate (6.7 mg, 50 μmol , 1.0 equiv.) and copper (I) thiophene-2-carboxylate (14.3 mg, 75.0 μmol, 1.50 equiv.). The vial was transferred into an anhydrous, N_2-filled glovebox. Under nitrogen atmosphere, $Cu(OTf)_2$ (45.2 mg, 125 μmol, 2.50 equiv.), CD_3CN (2.0 mL, *c* = 25 mM) were then added into the vial, resulting in a green suspension. The vial was sealed with a Teflon cap and placed 5 cm away from two purple LEDs (Kessil PR160-390 nm LEDs). The reaction mixture was stirred at a speed of 1200 rpm (Note: Fast stirring is essential due to the heterogeneity of the reaction mixture.) and irradiated for 16 h while maintaining the temperature at approximately 35 °C through cooling with a fan. Subsequently, dibromomethane (15 mg, 7.0 μL, 0.10 mmol, 0.80 equiv. to thiophene carboxylate) was added as an internal standard, and the yield of thiophene (8%, δ 7.57 (dd, *J* = 4.9, 1.0 Hz, 2H), 7.28 (m, *J* = 4.9, 1.0 Hz, 2H ppm)). was determined by ^{1}H NMR integration relative to the internal standard (δ 5.23 (s) ppm).

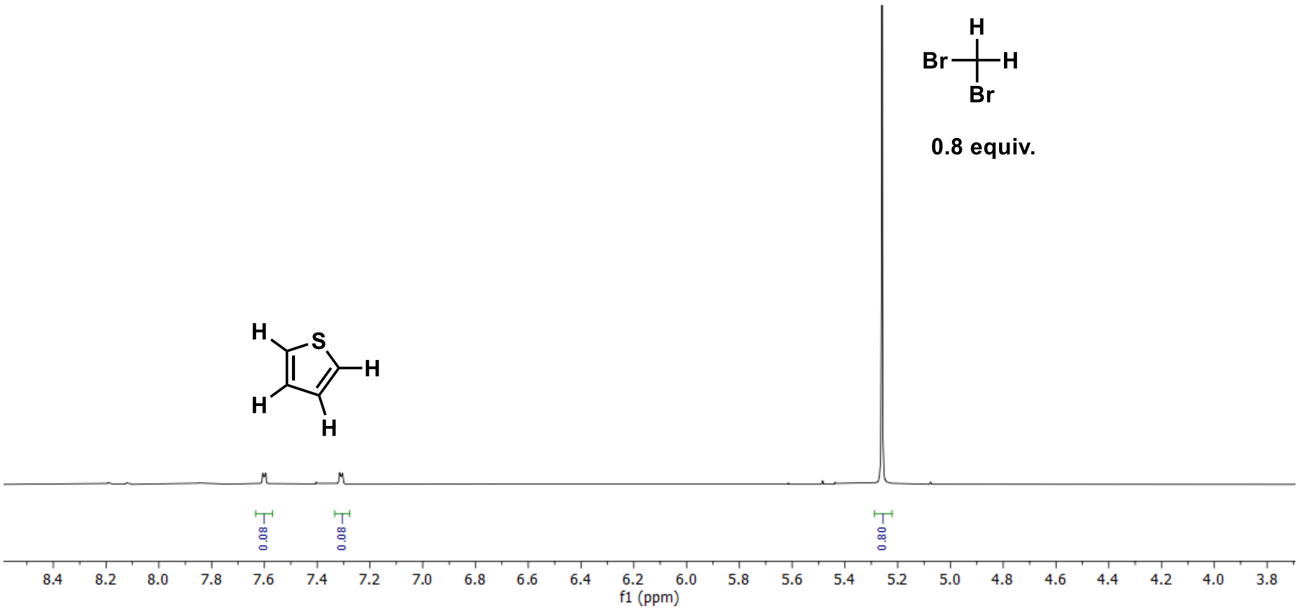

Figure S4. ^{1}H NMR of the reaction mixture

Decarboxylative hydroxylation of benzoic acids to phenols

Ataluren derivative 1

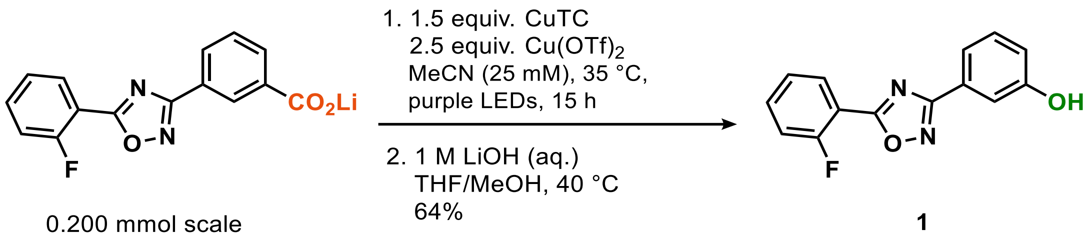

0.200 mmol scale **1**

Under an ambient atmosphere, a 10 mL borosilicate vial equipped with a magnetic stir bar was charged with the lithium salt of ataluren (58.0 mg, 0.200 mmol, 1.00 equiv.) and copper (I) thiophene-2-carboxylate (57.2 mg, 0.300 mmol, 1.50 equiv.). The vial was transferred into an anhydrous, N_2-filled glovebox. Under nitrogen atmosphere, $Cu(OTf)_2$ (181 mg, 0.500 mmol, 2.50 equiv.), and anhydrous MeCN (8.0 mL, *c* = 25 mM) were then added into the vial, resulting in a green suspension. The vial was sealed with a Teflon cap and placed 5 cm away from two purple LEDs (Kessil PR160-390 nm LEDs). The reaction mixture was stirred at a speed of 1200 rpm (Note: Fast stirring is essential due to the heterogeneity of the reaction mixture.) and irradiated for 16 h while maintaining the temperature at approximately 35 °C through cooling with a fan. Subsequently, the reaction mixture was transferred into a 50 mL round-bottom flask and evaporated to dryness. The residue was dissolved in EtOAc (40 mL) and filtered through a short pad of silica using EtOAc (50 mL) as the eluent to remove the majority of the copper salts. The filtrate was collected and concentrated under reduced pressure into a 20 mL borosilicate vial. To the 20 mL vial containing phenolic esters, a magnetic stir bar, THF (1.5 mL), MeOH (1.5 mL) and aqueous LiOH solution (1.0 M, 0.60 mL) were added. The reaction mixture was stirred at 40 °C and monitored by TLC until the phenolic esters were fully consumed. The reaction mixture was acidified by addition of 1 M aqueous HCl to pH = 3–5 (roughly 0.70 mL). The solution is concentrated to about 10% or less of its volume under reduced pressure. The remaining suspension was dissolved in EtOAc (20 mL) and transferred into a separatory funnel containing 5 mL saturated aqueous $NaHCO_3$ and 5 mL H_2O. The organic layer was collected, and the aqueous layer was further extracted with EtOAc (3 × ca. 20 mL). The combined organic layers were dried over Na_2SO_4, filtered, and the filtrate was concentrated under reduced pressure to dryness. The residue was purified by chromatography on silica gel (pentane/EtOAc = 4/1, v/v) to afford the ataluren derivative **1** (32.6 mg, 127 µmol, 64%) as an off-white solid.

R*f* =0.20 (pentane/EA = 4/1, v/v).

NMR Spectroscopy:

^{1}H NMR (600 MHz, CD_3CN, 298 K, δ): 8.20 (td, *J* = 7.5, 1.8 Hz, 1H), 7.70 (dddd, *J* = 8.4, 7.1, 5.1, 1.8 Hz, 1H), 7.63 (dt, *J* = 7.7, 1.3 Hz, 1H), 7.55 (dd, *J* = 2.6, 1.5 Hz, 1H), 7.45–7.38 (m, 2H), 7.39–7.35 (m, 2H), 7.02 (ddd, *J* = 8.2, 2.6, 1.0 Hz, 1H) ppm.

^{13}C NMR (151 MHz, CD_3CN, 298 K, δ): 173.8 (d, *J* = 4.3 Hz), 169.4, 161.6 (d, *J* = 257.8 Hz), 158.4, 136.2 (d, *J* = 8.9 Hz), 131.8 (d, *J* = 1.2 Hz), 131.5, 128.9, 126.1 (d, *J* = 3.7 Hz), 119.8, 119.5, 118.1 (d, *J* = 21.0

Hz), 114.7, 113.5 (d, J = 11.5 Hz) ppm.

^{19}F NMR (565 MHz, CD_3CN, 298 K, δ): –110.73 ppm.

HRMS-ESI (m/z) calculated for $C_{14}H_9N_2O_2FNa^+$ $[M+Na]^+$, 279.0540; found, 279.0540; deviation: 0 ppm.

1 mmol Scale synthesis of Ataluren derivative 1

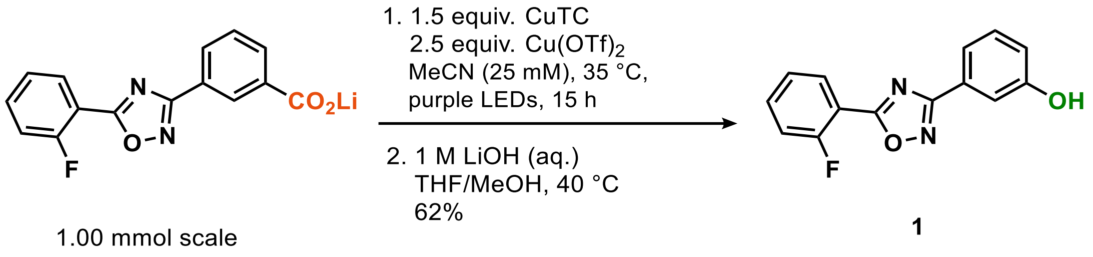

Under an ambient atmosphere, a 100 mL Schlenk tube equipped with a rubber stopper and magnetic stir bar was charged with the lithium salt of ataluren (290 mg, 1.00 mmol, 1.00 equiv.) and copper (I) thiophene-2-carboxylate (286 mg, 1.50 mmol, 1.50 equiv.). The vial was transferred into an anhydrous, N_2-filled glovebox. Under nitrogen atmosphere, $Cu(OTf)_2$ (904 mg, 0.500 mmol, 2.50 equiv.), and anhydrous MeCN (40 mL, c = 25 mM) were then added into the vial, resulting in a green suspension. The Schlenk tube was sealed with the rubber stopper and placed 5 cm away from two purple LEDs (Kessil PR160-390 nm LEDs). The reaction mixture was stirred at a speed of 1200 rpm (Note: Fast stirring is essential due to the heterogeneity of the reaction mixture.) and irradiated for 16 h while maintaining the temperature at approximately 35 °C through cooling with a fan. Subsequently, the reaction mixture was transferred into a 250 mL round-bottom flask and evaporated to dryness. The residue was dissolved in EtOAc (100 mL) and filtered through a short pad of silica using EtOAc (400 mL) as the eluent to remove the majority of the copper salts. The filtrate was collected and concentrated under reduced pressure into a 100 mL round-bottom flask. To the 100 mL flask containing phenolic esters, a magnetic stir bar, THF (7.5 mL), MeOH (7.5 mL) and aqueous LiOH solution (1.0 M, 4.0 mL) were added. The reaction mixture was stirred at 40 °C and monitored by TLC until the phenolic esters were fully consumed (roughly 5 hours). The reaction mixture was acidified by addition of 1 M aqueous HCl to pH = 3–5 (roughly 5 mL). The solution is concentrated to about 10% or less of its volume under reduced pressure. The remaining suspension was dissolved in EtOAc (100 mL) and transferred into a separatory funnel containing 25 mL saturated aqueous $NaHCO_3$ and 25 mL H_2O. The organic layer was collected, and the aqueous layer was further extracted with EtOAc (3 × ca. 100 mL). The combined organic layers were dried over Na_2SO_4, filtered, and the filtrate was concentrated under reduced pressure to dryness. The residue was purified by chromatography on silica gel (pentane/EtOAc = 4/1, v/v) to afford the ataluren derivative **1** (158 mg, 617 μmol, 62%) as an off-white solid.

4-Fluorophenol (2)

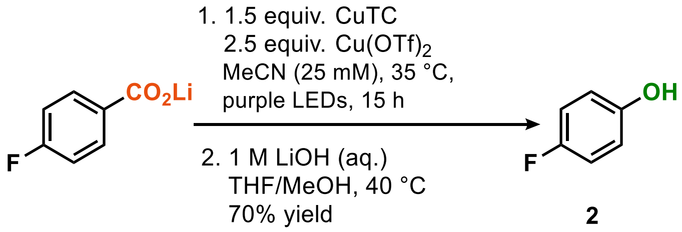

Under an ambient atmosphere, a 10 mL borosilicate vial equipped with a magnetic stir bar was charged with lithium 4-fluorobenzoate (29.2 mg, 0.200 mmol, 1.00 equiv.) and copper (I) thiophene-2-carboxylate (57.2 mg, 0.300 mmol, 1.50 equiv.). The vial was transferred into an anhydrous, N_2-filled glovebox. Under nitrogen atmosphere, $Cu(OTf)_2$ (181 mg, 0.500 mmol, 2.50 equiv.), and anhydrous MeCN (8.0 mL, *c* = 25 mM) were then added into the vial, resulting in a green suspension. The vial was sealed with a Teflon cap and placed 5 cm away from two purple LEDs (Kessil PR160-390 nm LEDs). The reaction mixture was stirred at a speed of 1200 rpm (Note: Fast stirring is essential due to the heterogeneity of the reaction mixture.) and irradiated for 16 h while maintaining the temperature at approximately 35 °C through cooling with a fan. Subsequently, the reaction mixture was transferred into a 50 mL round-bottom flask and evaporated to dryness. The residue was dissolved in EtOAc (40 mL) and filtered through a short pad of silica using EtOAc (50 mL) as the eluent to remove the majority of the copper salts. The filtrate was collected and concentrated under reduced pressure into a 20 mL borosilicate vial. To the 20 mL vial containing phenolic esters, a magnetic stir bar, THF (1.5 mL), MeOH (1.5 mL) and aqueous LiOH solution (1.0 M, 0.60 mL) were added. The reaction mixture was stirred at 40 °C and monitored by TLC until the phenolic esters were fully consumed. The reaction mixture was acidified by 1 M aqueous HCl to pH = 3–5. 4-fluoroanisole (25.2 mg, 22.5 μL, 0.200 mmol, 1.00 equiv.) was added as an internal standard, and the yield of 4-fluorophenol (**2**) was determined by ^{19}F NMR integration relative to the internal standard (72% yield, standard: δ –125.39 (m) ppm, and **2**: δ –126.95 (m) ppm, Figure **S5**). The identity of the product was further confirmed by GCMS (Figure **S6**) and HRMS-EI analysis

The yield of **2** reported in the manuscript (70%) represents an average of two runs [72% (above) and 69%].

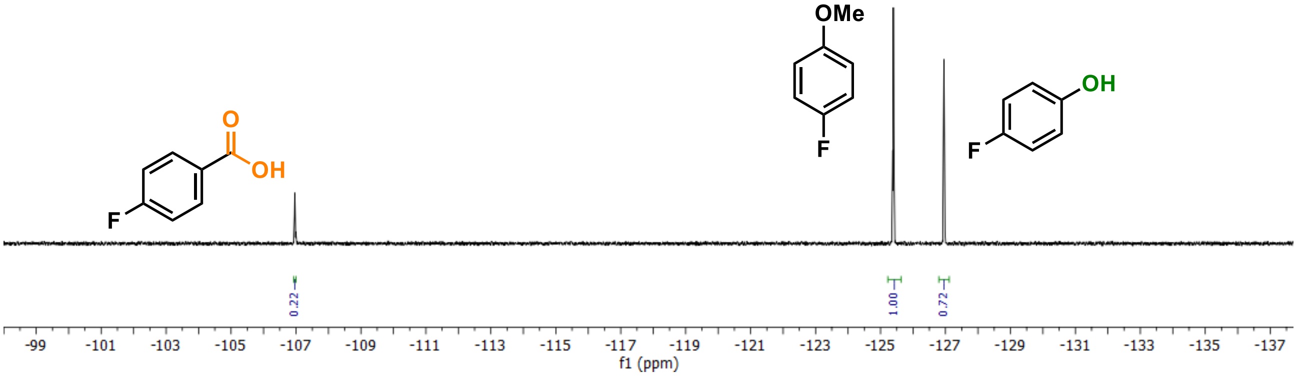

Figure S5. ^{19}F NMR of the resulting mixtures for product **2**

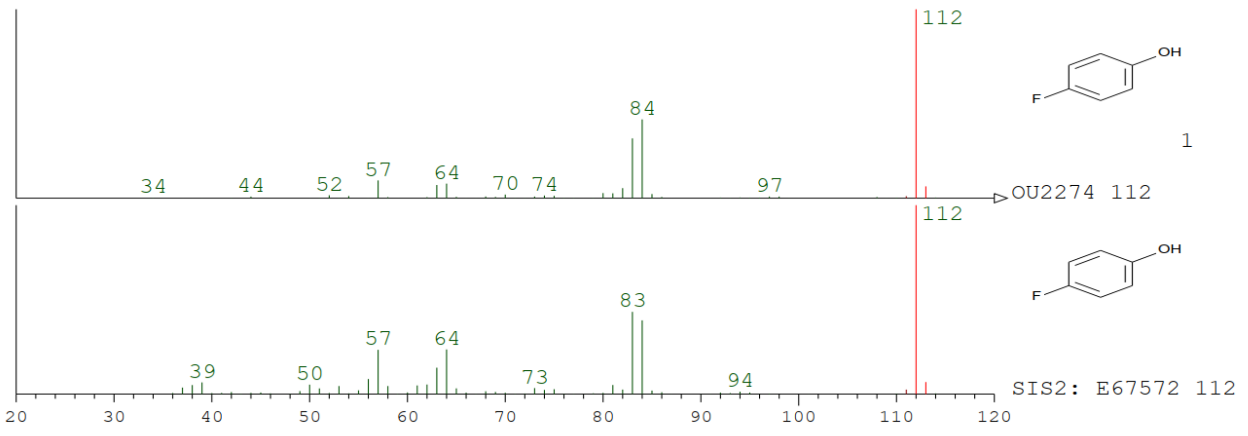

Figure S6. Mass spectrum of product **2** peak from GCMS

HRMS-EI (m/z) calculated for $C_6H_5OF^+$ $[M]^+$, 112.0319; found, 112.0321; deviation: –2 ppm.

One-pot synthesis of 4-fluorophenol (2)

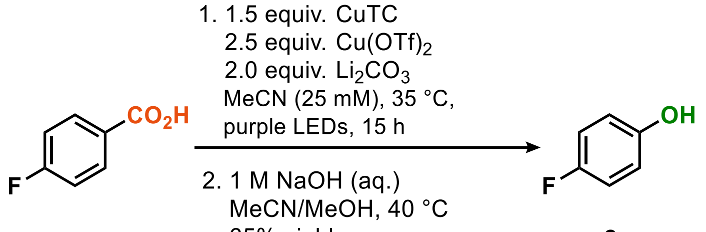

Under an ambient atmosphere, a 20 mL borosilicate vial equipped with a magnetic stir bar was charged with lithium 4-fluorobenzoic acid (28.0 mg, 0.200 mmol, 1.00 equiv.) and copper (I) thiophene-2-carboxylate (57.2 mg, 0.300 mmol, 1.50 equiv.). The vial was transferred into an anhydrous, N_2-filled glovebox. Under nitrogen atmosphere, $Cu(OTf)_2$ (181 mg, 0.500 mmol, 2.50 equiv.), and anhydrous MeCN (8.0 mL, *c* = 25 mM) were then added into the vial, resulting in a green suspension. The vial was sealed with a Teflon cap and placed 5 cm away from two purple LEDs (Kessil PR160-390 nm LEDs). The reaction mixture was stirred at a speed of 1200 rpm (Note: Fast stirring is essential due to the heterogeneity of the reaction mixture.) and irradiated for 16 h while maintaining the temperature at approximately 35 °C through cooling with a fan. Subsequently, MeOH (4.0 mL) and aqueous NaOH solution (1.0 M, 4.0 mL) were added into the same vial. The reaction mixture was stirred at 40 °C and monitored by TLC until the phenolic esters were fully consumed (about 1 h). The reaction mixture was acidified by 2.0 M aqueous HCl to pH = 3–5. 4-Fluoroanisole (25.2 mg, 22.5 μL, 0.200 mmol, 1.00 equiv.) was added as an internal standard, and the yield of 4-fluorophenol (**2**) was determined by ^{19}F NMR integration relative to the internal standard (65% yield, standard: δ –125.31 (m) ppm, and **2**: δ –126.62 (m) ppm, Figure **S7**)

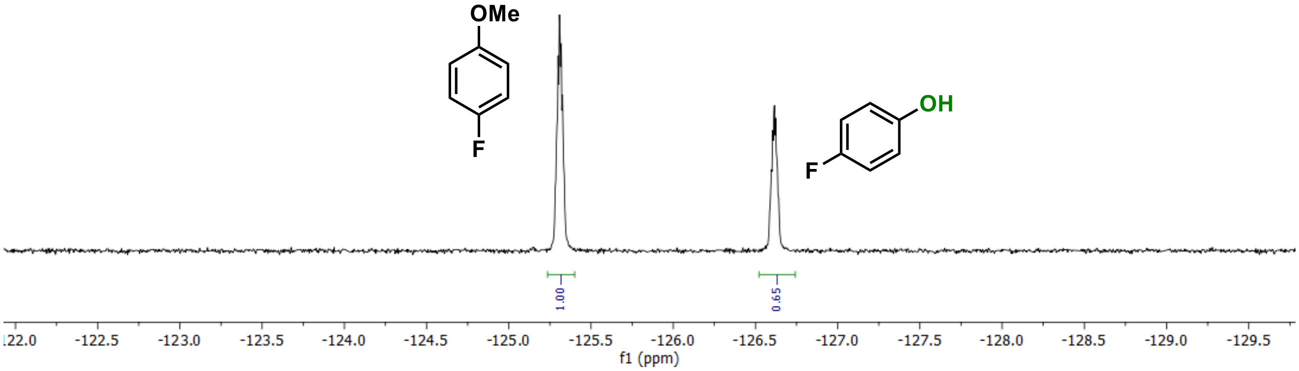

Figure **S7.** ^{19}F NMR of the resulting mixtures for product **2** from one-pot synthesis

4-Cyanophenol (3)

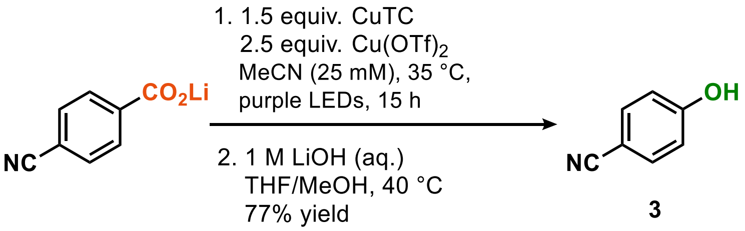

3

Under an ambient atmosphere, a 10 mL borosilicate vial equipped with a magnetic stir bar was charged with lithium 4-cyanobenzoate (30.6 mg, 0.200 mmol, 1.00 equiv.) and copper (I) thiophene-2-carboxylate (57.2 mg, 0.300 mmol, 1.50 equiv.). The vial was transferred into an anhydrous, N_2-filled glovebox. Under nitrogen atmosphere, $Cu(OTf)_2$ (181 mg, 0.500 mmol, 2.50 equiv.), and anhydrous MeCN (8.0 mL, c = 25 mM) were then added into the vial, resulting in a green suspension. The vial was sealed with a Teflon cap and placed 5 cm away from two purple LEDs (Kessil PR160-390 nm LEDs). The reaction mixture was stirred at a speed of 1200 rpm (Note: Fast stirring is essential due to the heterogeneity of the reaction mixture.) and irradiated for 16 h while maintaining the temperature at approximately 35 °C through cooling with a fan. Subsequently, the reaction mixture was transferred into a 50 mL round-bottom flask and evaporated to dryness. The residue was dissolved in EtOAc (40 mL) and filtered through a short pad of silica using EtOAc (50 mL) as the eluent to remove the majority of the copper salts. The filtrate was collected and concentrated under reduced pressure into a 20 mL borosilicate vial. To the 20 mL vial containing phenolic esters, a magnetic stir bar, THF (1.5 mL), MeOH (1.5 mL) and aqueous LiOH solution (1.0 M, 0.60 mL) were added. The reaction mixture was stirred at 40 °C and monitored by TLC until the phenolic esters were fully consumed. The reaction mixture was acidified by 1 M aqueous HCl to pH = 3–5 (roughly 0.70 mL). The solution is concentrated to about 10% or less of its volume under reduced pressure. The remaining suspension was dissolved in DCM (20 mL) and transferred into a separatory funnel containing 5 mL saturated aqueous $NaHCO_3$ and 5 mL H_2O. The organic layer was collected, and the aqueous layer was further extracted with DCM (3 × ca. 20 mL). The combined organic layers were dried over Na_2SO_4, filtered, and the filtrate was concentrated under reduced pressure to dryness. The residue was purified by chromatography on silica gel (DCM/EtOAc = 16/1, v/v) to afford the 4-cyanophenol (**3**) (18.3 mg, 154 µmol, 77%) as an off-white solid.

R*f* =0.30 (DCM/EA = 16/1, v/v).

NMR Spectroscopy:

^{1}H NMR (500 MHz, $CDCl_3$, 298 K, δ): 7.55 (d, *J* = 8.7 Hz, 2H), 6.93 (d, *J* = 8.8 Hz, 2H), 6.55 (brs, 1H). ppm.

^{13}C NMR (126 MHz, $CDCl_3$, 298 K, δ): 160.2, 134.5, 119.4, 116.6, 103.3.ppm.

HRMS-ESI (m/z) calculated for $C_7H_4NOF^-$ [M-H]$^-$, 118.0298; found, 118.0300; deviation: –1 ppm.

4-(Trifluoromethyl)phenol (4)

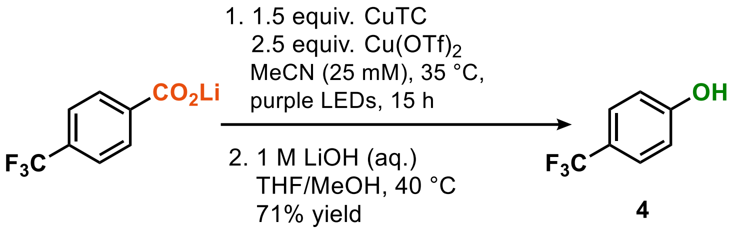

4

Under an ambient atmosphere, a 10 mL borosilicate vial equipped with a magnetic stir bar was charged with lithium 4-trifluoromethylbenzoate (39.2 mg, 0.200 mmol, 1.00 equiv.) and copper (I) thiophene-2-carboxylate (57.2 mg, 0.300 mmol, 1.50 equiv.). The vial was transferred into an anhydrous, N_2-filled glovebox. Under nitrogen atmosphere, $Cu(OTf)_2$ (181 mg, 0.500 mmol, 2.50 equiv.), and anhydrous MeCN (8.0 mL, *c* = 25 mM) were then added into the vial, resulting in a green suspension. The vial was sealed with a Teflon cap and placed 5 cm away from two purple LEDs (Kessil PR160-390 nm LEDs). The reaction mixture was stirred at a speed of 1200 rpm (Note: Fast stirring is essential due to the heterogeneity of the reaction mixture.) and irradiated for 16 h while maintaining the temperature at approximately 35 °C through cooling with a fan. Subsequently, the reaction mixture was transferred into a 50 mL round-bottom flask and evaporated to dryness. The residue was dissolved in EtOAc (40 mL) and filtered through a short pad of silica using EtOAc (50 mL) as the eluent to remove the majority of the copper salts. The filtrate was collected and concentrated under reduced pressure into a 20 mL borosilicate vial. To the 20 mL vial containing phenolic esters, a magnetic stir bar, THF (1.5 mL), MeOH (1.5 mL) and aqueous LiOH solution (1.0 M, 0.60 mL) were added. The reaction mixture was stirred at 40 °C and monitored by TLC until the phenolic esters were fully consumed. The reaction mixture was acidified by 1 M aqueous HCl to pH = 3–5. 4-Fluorobenzotrifluoride (32.8 mg, 25.4 μL, 0.200 mmol, 1.00 equiv.) was added as an internal standard, and the yield of 4-(trifluoromethyl)phenol (**4**) was determined by ^{19}F NMR integration relative to the internal standard (71% yield, standard: δ –63.16 ppm, and **4**: δ –62.45 ppm, Figure **S8**). The identity of the product was further confirmed by GCMS (Figure **S9**) and HRMS-EI analysis

The yield of **4** reported in the manuscript (71%) represents an average of two runs [71% (above) and 70%].

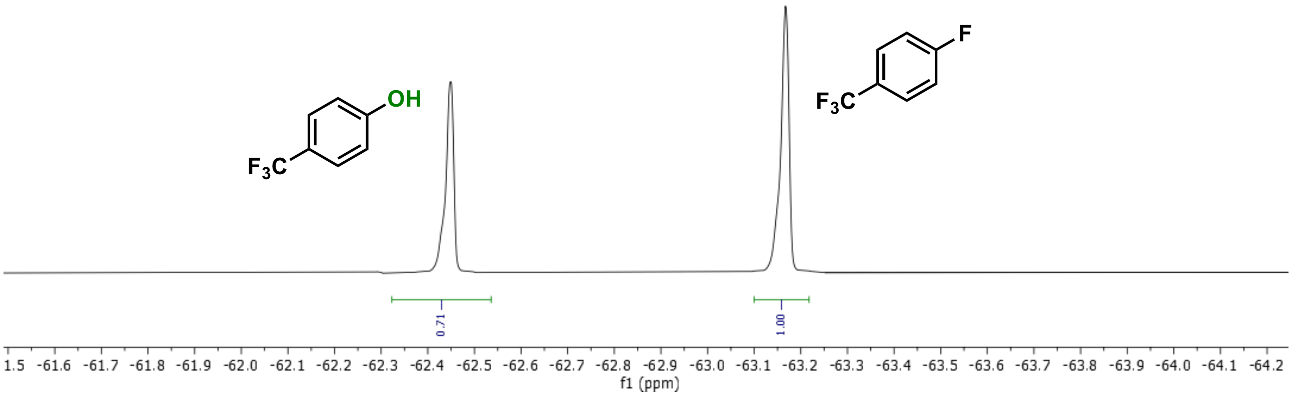

Figure S8. ^{19}F NMR of the resulting mixtures for product **4**

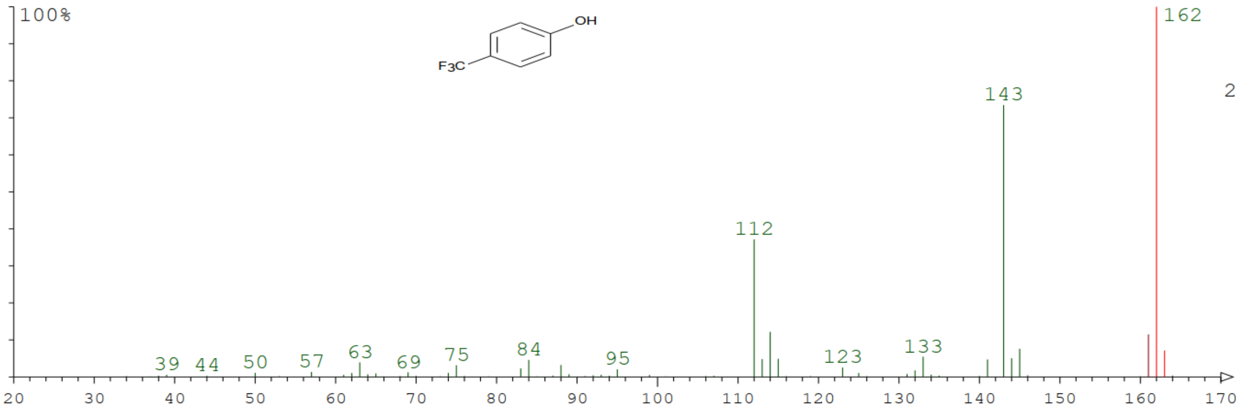

Figure S9. Mass spectrum of product **4** peak from GCMS

HRMS-EI (m/z) calculated for $C_7H_5OF_3^+$ [M]$^+$,162.0287; found,162.0289; deviation: –1 ppm.

2,4-Bis(trifluoromethyl)phenol (5)

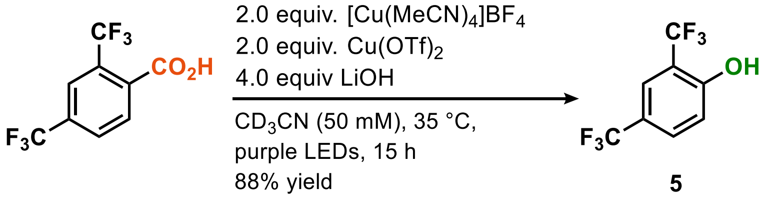

Under an ambient atmosphere, a 10 mL borosilicate vial equipped with a magnetic stir bar was charged with 2,4-bis(trifluoromethyl)benzoic acid (51.6 mg, 0.200 mmol, 1.00 equiv.) and LiOH (19.1 mg, 0.800 mmol, 4.00 equiv.). The vial was transferred into an anhydrous, N_2-filled glovebox. Under nitrogen atmosphere, [Cu(MeCN)$_4$BF$_4$] (125 mg, 0.400 mmol, 2.00 equiv.), Cu(OTf)$_2$ (145 mg, 0.400 mmol, 2.00 equiv.) and CD_3CN (4.0 mL, *c* = 50 mM) were then added into the vial, resulting in a blue suspension. The vial was sealed with a Teflon cap and placed 5 cm away from two purple LEDs (Kessil PR160-390 nm LEDs). The reaction mixture was stirred at a speed of 1200 rpm (Note: Fast stirring is essential due to the heterogeneity of the reaction mixture.) and irradiated for 16 h while maintaining the temperature at approximately 35 °C through cooling with

a fan. Subsequently, trifuoromethylbenzene (29.2 mg, 24.6 μL, 0.200 mmol, 1.00 equiv.) was added as an internal standard, and the yield of 2,4-Bis(trifluoromethyl)phenol (**5**) was determined by ^{19}F NMR integration relative to the internal standard (88% yield, standard: δ –63.19 (m) ppm, and **5**: δ –63.50 (m) ppm, Figure **S10**). The identity of the product was further confirmed by ^{1}H NMR (Figure **S11**), GCMS (Figure **S12**) and HRMS-EI analysis

The yield of **5** reported in the manuscript (88%) represents an average of two runs [88% (above) and 89%].

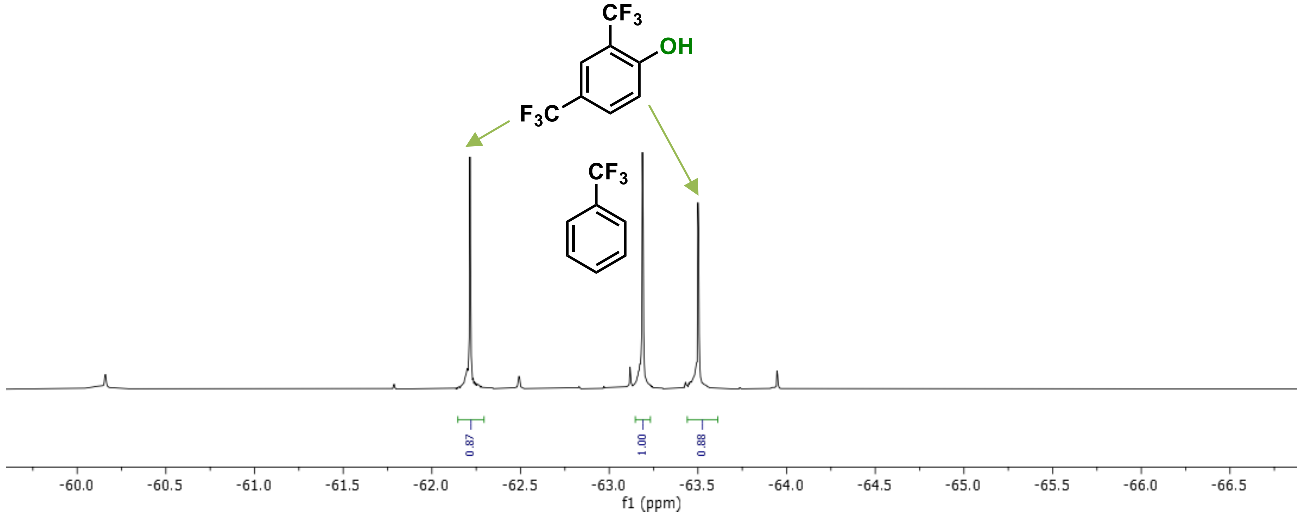

Figure S10. ^{19}F NMR of the resulting mixtures for product **5**

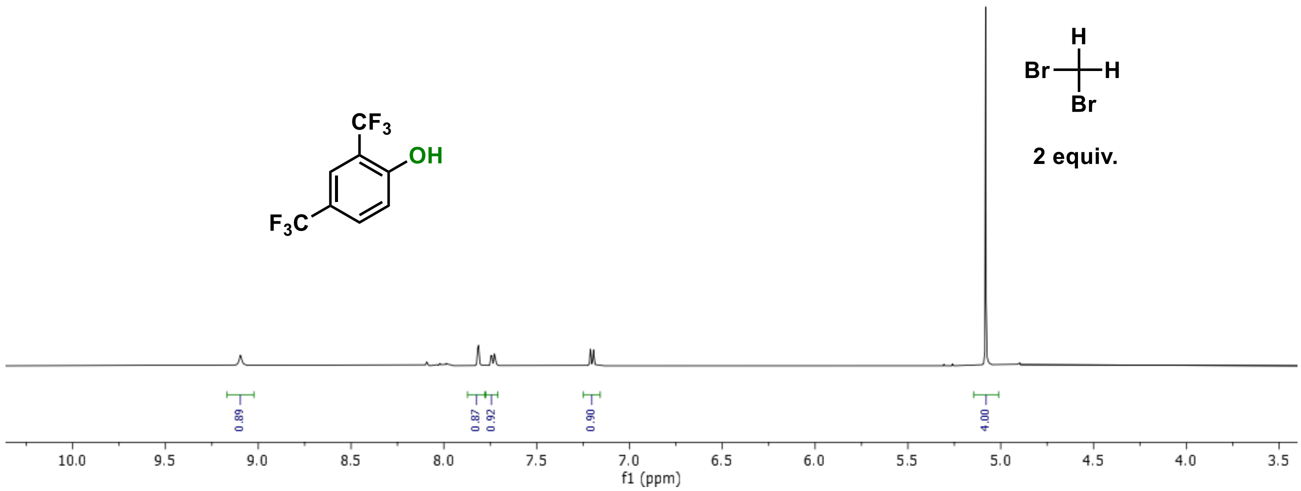

Figure S11. ^{1}H NMR of the resulting mixtures for product **5** (based on 0.05 mmol scale)

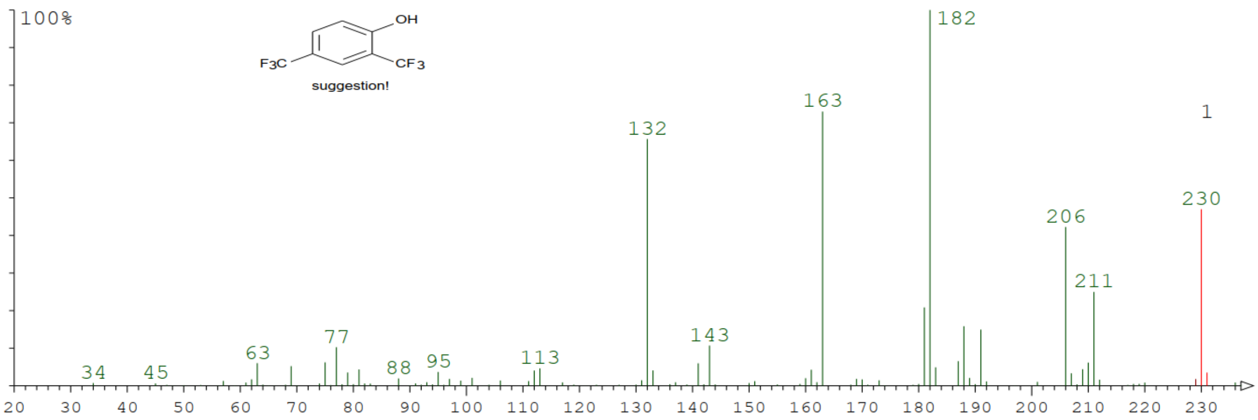

Figure S12. Mass spectrum of product **5** peak from GCMS

HRMS-EI (m/z) calculated for $C_8H_4OF_6^+$ [M]$^+$, 230.0161; found,230.0159; deviation: 0.7 ppm.

Note: This is the only example in which we directly obtained the phenol product after irradiation without an additional hydrolysis step. We proposed the phenol was produced from *in situ* hydrolysis of generated homo-ester, which released the unreacted carboxylates and eliminated the need of the coupling partner, CuTC, to afford more than 50% oxydecarboxylation yield. The possible reason for the unique reactivity could be the generated homo-ester, 2,4-bis(trifluoromethyl)phenyl 2,4-bis(trifluoromethyl)benzoate, can be hydrolyzed very easily compared to phenyl benzoate. Electron-withdrawing groups at the 2 and 4 positions significantly increase the electrophilicity of the carbonyl and the corresponding phenol is a good leaving group.

2-(Trifluoromethyl)pyridin-4-ol (6)

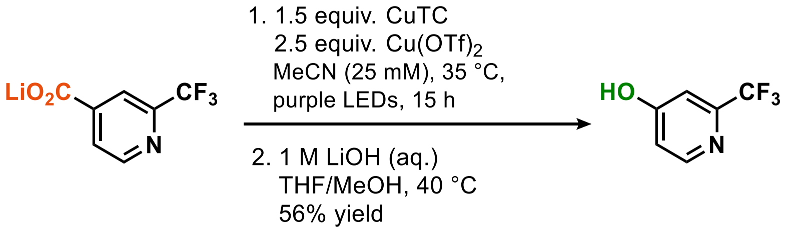

6

Under an ambient atmosphere, a 10 mL borosilicate vial equipped with a magnetic stir bar was charged with lithium 2-(trifluoromethyl)isonicotinate (39.4 mg, 0.200 mmol, 1.00 equiv.) and copper (I) thiophene-2-carboxylate (57.2 mg, 0.300 mmol, 1.50 equiv.). The vial was transferred into an anhydrous, N_2-filled glovebox. Under nitrogen atmosphere, $Cu(OTf)_2$ (181 mg, 0.500 mmol, 2.50 equiv.), and anhydrous MeCN (8.0 mL, c = 25 mM) were then added into the vial, resulting in a green suspension. The vial was sealed with a Teflon cap and placed 5 cm away from two purple LEDs (Kessil PR160-390 nm LEDs). The reaction mixture was stirred at a speed of 1200 rpm (Note: Fast stirring is essential due to the heterogeneity of the reaction mixture.) and irradiated for 16 h while maintaining the temperature at approximately 35 °C through cooling with a fan. Subsequently, the reaction mixture was transferred into a 50 mL round-bottom flask and evaporated to dryness. The residue was dissolved in EtOAc (40 mL) and filtered through a short pad of silica using EtOAc (50 mL) as the eluent to remove the majority of the copper salts. The filtrate was collected and concentrated under reduced

pressure into a 20 mL borosilicate vial. To the 20 mL vial containing phenolic esters, a magnetic stir bar, THF (1.5 mL), MeOH (1.5 mL) and aqueous LiOH solution (1.0 M, 0.60 mL) were added. The reaction mixture was stirred at 40 °C and monitored by TLC until the phenolic esters were fully consumed. The reaction mixture was acidified by 1 M aqueous HCl to pH = 3–5 (roughly 0.70 mL). The solution is concentrated to about 10% or less of its volume under reduced pressure. The remaining suspension was dissolved in EtOAc (20 mL) and transferred into a separatory funnel containing 5 mL saturated aqueous $NaHCO_3$ and 5 mL H_2O. The organic layer was collected, and the aqueous layer was further extracted with EtOAc (3 × ca. 20 mL). The combined organic layers were dried over Na_2SO_4, filtered, and the filtrate was concentrated under reduced pressure to dryness. The residue was purified by chromatography on silica gel (DCM/EtOAc = 5/1, v/v) to afford the 2-(trifluoromethyl)pyridin-4-ol (**6**) (18.3 mg, 112 µmol, 56%) as a colorless solid.

R*f* =0.20 (DCM/EA = 5/1, v/v).

NMR Spectroscopy:

^{1}H NMR (500 MHz, $CDCl_3$, 298 K, δ): 9.25 (brs, 1H), 8.22 (d, *J* = 6.1 Hz, 1H), 7.19 (d, *J* = 2.4 Hz, 1H), 6.96 (dd, *J* = 6.1, 2.5 Hz, 1H) ppm.

^{13}C NMR (126 MHz, $CDCl_3$, 298 K, δ): 169.8, 147.9, 146.9 (d, *J* = 34.8 Hz), 121.1 (d, *J* = 275.4 Hz), 115.6, 111.4 ppm

^{19}F NMR (471 MHz, $CDCl_3$, 298 K, δ): –67.66ppm.

HRMS-EI (m/z) calculated for $C_6H_4NOF_3^+$ $[M]^+$, 163.0239; found, 163.0241; deviation: –1 ppm.

4-(5-Methyl-1,2,4-oxadiazol-3-yl)phenol (7)

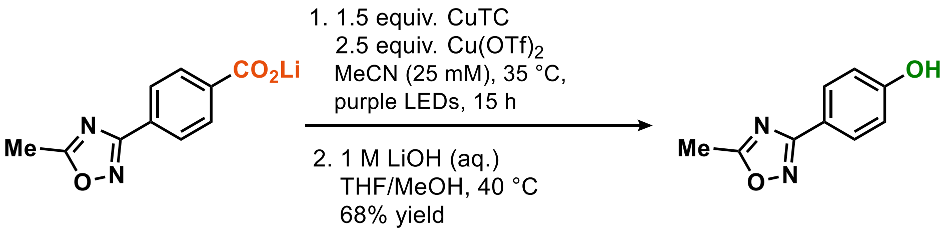

7

Under an ambient atmosphere, a 10 mL borosilicate vial equipped with a magnetic stir bar was charged with lithium 4-(5-methyl-1,2,4-oxadiazol-3-yl)benzoate (42.0 mg, 0.200 mmol, 1.00 equiv.) and copper (I) thiophene-2-carboxylate (57.2 mg, 0.300 mmol, 1.50 equiv.). The vial was transferred into an anhydrous, N_2-filled glovebox. Under nitrogen atmosphere, $Cu(OTf)_2$ (181 mg, 0.500 mmol, 2.50 equiv.), and anhydrous MeCN (8.0 mL, *c* = 25 mM) were then added into the vial, resulting in a green suspension. The vial was sealed with a Teflon cap and placed 5 cm away from two purple LEDs (Kessil PR160-390 nm LEDs). The reaction mixture was stirred at a speed of 1200 rpm (Note: Fast stirring is essential due to the heterogeneity of the reaction mixture.) and irradiated for 16 h while maintaining the temperature at approximately 35 °C through cooling with a fan. Subsequently, the reaction mixture was transferred into a 50 mL round-bottom flask and evaporated to dryness. The residue was dissolved in EtOAc (40 mL) and filtered through a short pad of silica using EtOAc (50 mL) as

the eluent to remove the majority of the copper salts. The filtrate was collected and concentrated under reduced pressure into a 20 mL borosilicate vial. To the 20 mL vial containing phenolic esters, a magnetic stir bar, THF (1.5 mL), MeOH (1.5 mL) and aqueous LiOH solution (1.0 M, 0.60 mL) were added. The reaction mixture was stirred at 40 °C and monitored by TLC until the phenolic esters were fully consumed. The reaction mixture was acidified by addition of 1 M aqueous HCl to pH = 3–5 (roughly 0.70 mL). The solution is concentrated to about 10% or less of its volume under reduced pressure. The remaining suspension was dissolved in EtOAc (20 mL) and transferred into a separatory funnel containing 5 mL saturated aqueous $NaHCO_3$ and 5 mL H_2O. The organic layer was collected, and the aqueous layer was further extracted with EtOAc (3 × ca. 20 mL). The combined organic layers were dried over Na_2SO_4, filtered, and the filtrate was concentrated under reduced pressure to dryness. The residue was purified by chromatography on silica gel (pentane/EtOAc = 4/1, v/v) to afford 4-(5-methyl-1,2,4-oxadiazol-3-yl)phenol (**7**) (24.0 mg, 136 µmol, 68%) as a colorless solid.

R*f* =0.15 (pentane/EA = 4/1, v/v).

NMR Spectroscopy:

^{1}**H NMR** (500 MHz, CD_3CN, 298 K, δ): 7.88 (d, *J* = 8.9 Hz, 1H), 6.92 (d, *J* = 9.0 Hz, 1H), 2.57 (s, 3H) ppm.

^{13}**C NMR** (126 MHz, CD_3CN, 298 K, δ): 177.9, 169.0, 160.6, 129.9, 119.6, 116.7, 12.6 ppm.

HRMS-ESI (m/z) calculated for $C_9H_7N_2O_2^-$ $[M-H]^-$, 175.0513; found, 175.0513; deviation: –0.3 ppm.

6-Hydroxy-2-naphthonitrile (8)

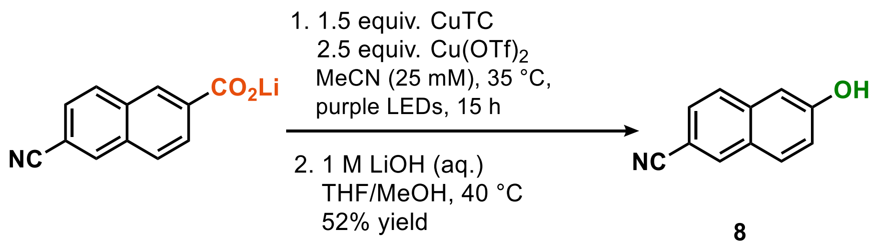

8

Under an ambient atmosphere, a 10 mL borosilicate vial equipped with a magnetic stir bar was charged with lithium 6-cyano-2-naphthoate (40.6 mg, 0.200 mmol, 1.00 equiv.) and copper (I) thiophene-2-carboxylate (57.2 mg, 0.300 mmol, 1.50 equiv.). The vial was transferred into an anhydrous, N_2-filled glovebox. Under nitrogen atmosphere, $Cu(OTf)_2$ (181 mg, 0.500 mmol, 2.50 equiv.), and anhydrous MeCN (8.0 mL, *c* = 25 mM) were then added into the vial, resulting in a green suspension. The vial was sealed with a Teflon cap and placed 5 cm away from two purple LEDs (Kessil PR160-390 nm LEDs). The reaction mixture was stirred at a speed of 1200 rpm (Note: Fast stirring is essential due to the heterogeneity of the reaction mixture.) and irradiated for 16 h while maintaining the temperature at approximately 35 °C through cooling with a fan. Subsequently, the reaction mixture was transferred into a 50 mL round-bottom flask and evaporated to dryness. The residue was dissolved in EtOAc (40 mL) and filtered through a short pad of silica using EtOAc (50 mL) as the eluent to remove the majority of the copper salts. The filtrate was collected and concentrated under reduced pressure into a 20 mL borosilicate vial. To the 20 mL vial containing phenolic esters, a magnetic stir bar, THF (1.5 mL), MeOH (1.5 mL) and aqueous LiOH solution (1.0 M, 0.60 mL) were added. The reaction mixture was stirred at

40 °C and monitored by TLC until the phenolic esters were fully consumed. The reaction mixture was acidified by addition of 1 M aqueous HCl to pH = 3–5 (roughly 0.70 mL). The solution is concentrated to about 10% or less of its volume under reduced pressure. The remaining suspension was dissolved in EtOAc (20 mL) and transferred into a separatory funnel containing 5 mL saturated aqueous $NaHCO_3$ and 5 mL H_2O. The organic layer was collected, and the aqueous layer was further extracted with EtOAc (3 × ca. 20 mL). The combined organic layers were dried over Na_2SO_4, filtered, and the filtrate was concentrated under reduced pressure to dryness. The residue was purified by chromatography on silica gel (pentane/EtOAc = 4/1, v/v) to afford 6-hydroxy-2-naphthonitrile (**8**) (17.4 mg, 102 μmol, 52%) as a colorless solid.

R*f* =0.15 (pentane/EA = 4/1, v/v).

NMR Spectroscopy:

^{1}H NMR (600 MHz, CD_3CN, 298 K, δ): 8.31–8.22 (m, 1H), 7.87 (d, *J* = 9.8 Hz, 1H), 7.82 (d, *J* = 8.6 Hz, 1H), 7.75 (brs, 1H,), 7.59 (dd, *J* = 8.6, 1.7 Hz, 1H), 7.28–7.23 (m, 2H) ppm.

^{13}C NMR (151 MHz, CD_3CN, 298 K, δ): 158.6, 137.6, 135.1, 131.4, 128.4, 128.2, 127.8, 121.0, 120.5, 110.2, 106.9 ppm.

HRMS-ESI (m/z) calculated for $C_{11}H_6NO^-$ $[M\text{-}H]^-$, 168.0455; found, 168.0456; deviation: –0.7 ppm.

(+)-Menthol derivative 9

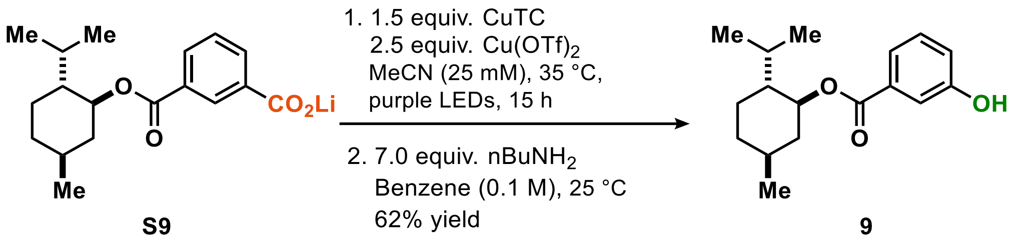

Under an ambient atmosphere, a 10 mL borosilicate vial equipped with a magnetic stir bar was charged with lithium (+)-menthol derived benzoate (62.1 mg, 0.200 mmol, 1.00 equiv.) and copper (I) thiophene-2-carboxylate (57.2 mg, 0.300 mmol, 1.50 equiv.). The vial was transferred into an anhydrous, N_2-filled glovebox. Under nitrogen atmosphere, $Cu(OTf)_2$ (181 mg, 0.500 mmol, 2.50 equiv.), and anhydrous MeCN (8.0 mL, *c* = 25 mM) were then added into the vial, resulting in a green suspension. The vial was sealed with a Teflon cap and placed 5 cm away from two purple LEDs (Kessil PR160-390 nm LEDs). The reaction mixture was stirred at a speed of 1200 rpm (Note: Fast stirring is essential due to the heterogeneity of the reaction mixture.) and irradiated for 16 h while maintaining the temperature at approximately 35 °C through cooling with a fan. Subsequently, the reaction mixture was transferred into a 50 mL round-bottom flask and evaporated to dryness. The residue was dissolved in EtOAc (40 mL) and filtered through a short pad of silica using EtOAc (50 mL) as the eluent to remove the majority of the copper salts. The filtrate was collected and concentrated under reduced pressure into a 20 mL borosilicate vial. To the 20 mL vial containing phenolic esters, a magnetic stir bar, benzene (2.0 mL) and $nBuNH_2$ (102 mg, 138 μL, 1.40 mmol, 7.00 equiv.) were added.[4] The reaction mixture was stirred at 25 °C and monitored by TLC until the phenolic esters were fully consumed. The reaction mixture

was concentrated under reduced pressure to dryness. The residue was purified by chromatography on silica gel (pentane/EtOAc = 20/1, v/v) to afford the (+)-menthol derivative **9** (34.2 mg, 124 μmol, 62%) as a colorless solid.

R*f* =0.10 (pentane/EA = 20/1, v/v).

NMR Spectroscopy:

^{1}H NMR (500 MHz, CD_2Cl_2, 298 K, δ): 7.59 (dt, *J* = 7.6, 1.2 Hz, 1H), 7.56 (dd, *J* = 2.7, 1.5 Hz, 1H), 7.32 (t, *J* = 7.9 Hz, 1H), 7.06 (ddd, *J* = 8.1, 2.7, 1.0 Hz, 1H), 5.95 (brs, 1H), 4.91 (td, *J* = 10.9, 4.4 Hz, 1H), 2.09 (dtd, *J* = 12.0, 4.0, 1.8 Hz, 1H), 1.95 (pd, *J* = 7.0, 2.8 Hz, 1H), 1.83–1.65 (m, 2H), 1.58–1.52 (m, 2H), 1.20–1.05 (m, 2H), 0.95–0.93 (m, 1H), 0.93–0.91 (m, 6H), 0.78 (d, *J* = 6.9 Hz, 3H) ppm.

^{13}C NMR (101 MHz, CD_2Cl_2, 298 K, δ): 166.5, 156.4, 132.7, 130.0, 122.1, 120.3, 116.6, 75.6, 47.6, 41.3, 34.7, 31.8, 26.9, 24.0, 22.2, 20.9, 16.6 ppm.

HRMS-CI (m/z) calculated for $C_{17}H_{23}O_3^-$ [M-H]$^-$, 275.1642; found, 275.1639; deviation: 0.8 ppm.

3-Hydroxybenzaldehyde (10)

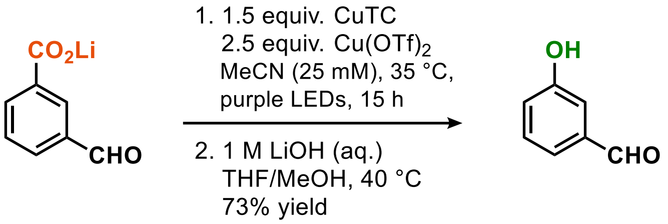

10

Under an ambient atmosphere, a 10 mL borosilicate vial equipped with a magnetic stir bar was charged with lithium 3-formylbenzoate (31.2 mg, 0.200 mmol, 1.00 equiv.) and copper (I) thiophene-2-carboxylate (57.2 mg, 0.300 mmol, 1.50 equiv.). The vial was transferred into an anhydrous, N_2-filled glovebox. Under nitrogen atmosphere, $Cu(OTf)_2$ (181 mg, 0.500 mmol, 2.50 equiv.), and anhydrous MeCN (8.0 mL, *c* = 25 mM) were then added into the vial, resulting in a green suspension. The vial was sealed with a Teflon cap and placed 5 cm away from two purple LEDs (Kessil PR160-390 nm LEDs). The reaction mixture was stirred at a speed of 1200 rpm (Note: Fast stirring is essential due to the heterogeneity of the reaction mixture.) and irradiated for 16 h while maintaining the temperature at approximately 35 °C through cooling with a fan. Subsequently, the reaction mixture was transferred into a 50 mL round-bottom flask and evaporated to dryness. The residue was dissolved in EtOAc (40 mL) and filtered through a short pad of silica using EtOAc (50 mL) as the eluent to remove the majority of the copper salts. The filtrate was collected and concentrated under reduced pressure into a 20 mL borosilicate vial. To the 20 mL vial containing phenolic esters, a magnetic stir bar, THF (1.5 mL), MeOH (1.5 mL) and aqueous LiOH solution (1.0 M, 0.60 mL) were added. The reaction mixture was stirred at 40 °C and monitored by TLC until the phenolic esters were fully consumed. The reaction mixture was acidified by addition of 1 M aqueous HCl to pH = 3–5 (roughly 0.70 mL). The solution is concentrated to about 10% or less of its volume under reduced pressure. The remaining suspension was dissolved in EtOAc (20 mL) and transferred into a separatory funnel containing 5 mL saturated aqueous $NaHCO_3$ and 5 mL H_2O. The organic

layer was collected, and the aqueous layer was further extracted with EtOAc (3 × ca. 20 mL). The combined organic layers were dried over Na_2SO_4, filtered, and the filtrate was concentrated under reduced pressure to dryness. The residue was purified by chromatography on silica gel (pentane/EtOAc = 4/1, v/v) to afford 3-hydroxybenzaldehyde (**10**) (17.8 mg, 146 μmol, 73%) as a colorless solid.

R*f* =0.25 (pentane/EA = 4/1, v/v).

NMR Spectroscopy:

^{1}**H NMR** (500 MHz, CD_3CN, 298 K, δ): 9.92 (s, 1H), 7.43 (m, 1H), 7.41 (dd, *J* = 2.1, 1.3 Hz, 1H), 7.39 (s, 1H), 7.28 (ddd, *J* = 2.6, 1.3, 0.6 Hz, 1H), 7.16–7.08 (m, 1H) ppm.

^{13}**C NMR** (126 MHz, CD_3CN, 298 K, δ): 193.5, 158.5, 139.2, 131.4, 122.8, 122.6, 115.6 ppm.

HRMS-EI (m/z) calculated for $C_7H_6O_2^+$ [M]$^+$, 122.0362; found, 122.0363; deviation: –0.3 ppm.

4-Methylphenol (11)

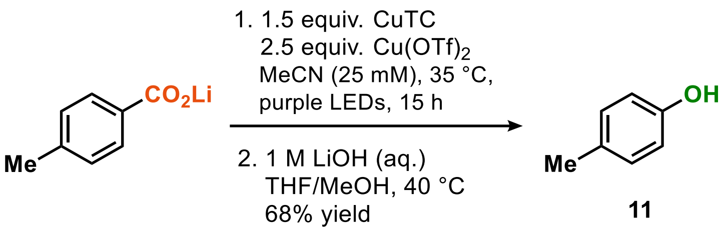

11

Under an ambient atmosphere, a 10 mL borosilicate vial equipped with a magnetic stir bar was charged with lithium 4-methylbenzoate (28.4 mg, 0.200 mmol, 1.00 equiv.) and copper (I) thiophene-2-carboxylate (57.2 mg, 0.300 mmol, 1.50 equiv.). The vial was transferred into an anhydrous, N_2-filled glovebox. Under nitrogen atmosphere, $Cu(OTf)_2$ (181 mg, 0.500 mmol, 2.50 equiv.), and anhydrous MeCN (8.0 mL, *c* = 25 mM) were then added into the vial, resulting in a green suspension. The vial was sealed with a Teflon cap and placed 5 cm away from two purple LEDs (Kessil PR160-390 nm LEDs). The reaction mixture was stirred at a speed of 1200 rpm (Note: Fast stirring is essential due to the heterogeneity of the reaction mixture.) and irradiated for 16 h while maintaining the temperature at approximately 35 °C through cooling with a fan. Subsequently, the reaction mixture was transferred into a 50 mL round-bottom flask and evaporated to dryness. The residue was dissolved in EtOAc (40 mL) and filtered through a short pad of silica using EtOAc (50 mL) as the eluent to remove the majority of the copper salts. The filtrate was collected and concentrated under reduced pressure into a 20 mL borosilicate vial. To the 20 mL vial containing phenolic esters, a magnetic stir bar, THF (1.5 mL), MeOH (1.5 mL) and aqueous LiOH solution (1.0 M, 0.60 mL) were added. The reaction mixture was stirred at 40 °C and monitored by TLC until the phenolic esters were fully consumed. The reaction mixture was acidified by 1 M aqueous HCl to pH = 3–5. The solution is concentrated to about 10% of its volume under reduced pressure. The remaining suspension was dissolved in EtOAc (20 mL) and transferred into a separatory funnel containing 10 mL H_2O. The organic layer was collected, and the aqueous layer was further extracted with EtOAc (3 × ca. 10 mL). The combined organic layers were dried over Na_2SO_4, filtered, and the filtrate was concentrated under reduced pressure to roughly 0.1 mL then diluted with 0.50 mL $CDCl_3$. Dibromomethane

(34.8 mg, 14.0 μL, 0.200 mmol, 1.00 equiv.) was added as an internal standard, and the yield of 4-methylphenol (**11**) was determined by ^{1}H NMR integration relative to the internal standard (66% yield, standard: δ 4.91 (s) ppm, and **11**: δ 6.99 (d, *J* = 8.5 Hz) ppm, Figure **S13**). The identity of the product was further confirmed by GCMS (Figure **S14**) and HRMS-EI analysis.

The yield of **11** reported in the manuscript (68%) represents an average of two runs [66% (above) and 70%].

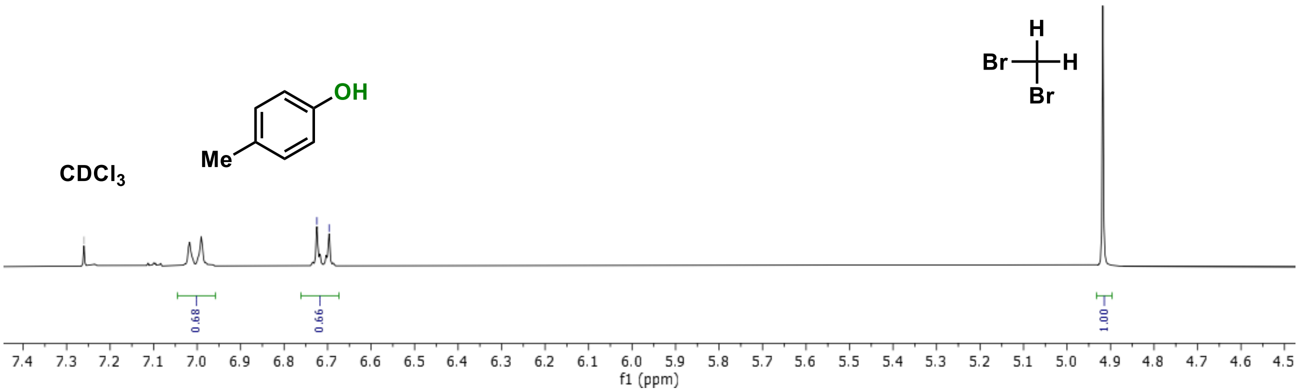

Figure S13. ^{1}H NMR of the resulting mixtures for product **11**

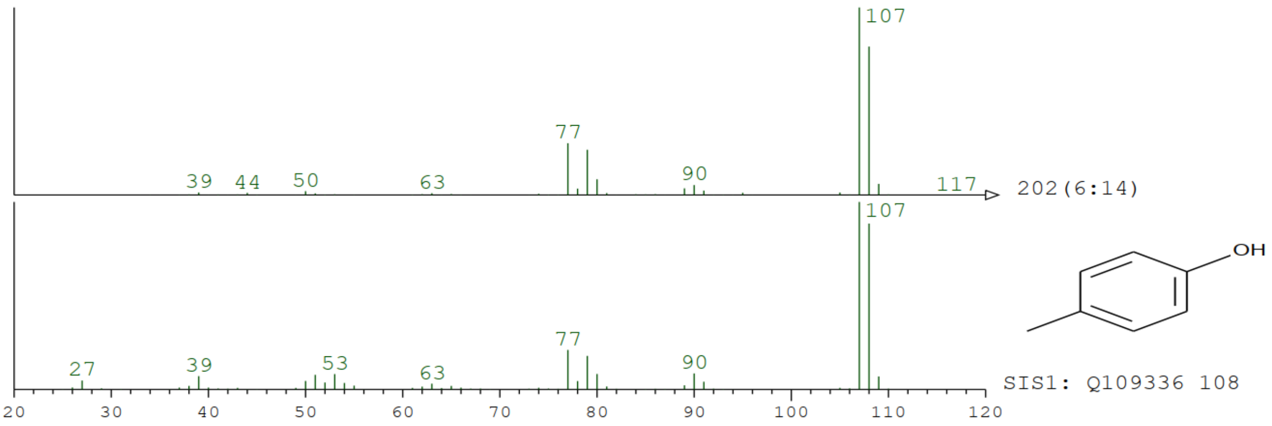

Figure S14. Mass spectrum of product **11** peak from GCMS

HRMS-EI (m/z) calculated for $C_7H_8O^+$ $[M]^+$,108.0569; found,108:0570; deviation: –0.6 ppm.

4-Methoxyphenol (12)

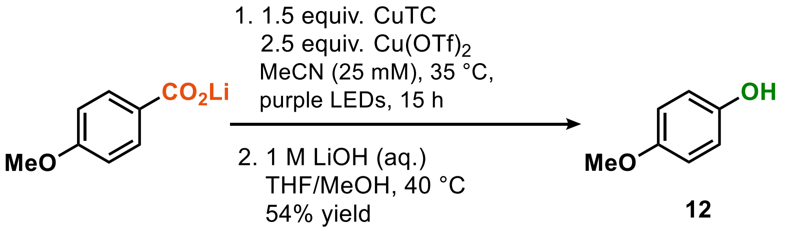

12

Under an ambient atmosphere, a 10 mL borosilicate vial equipped with a magnetic stir bar was charged with lithium 4-methoxybenzoate (31.6 mg, 0.200 mmol, 1.00 equiv.) and copper (I) thiophene-2-carboxylate (57.2

mg, 0.300 mmol, 1.50 equiv.). The vial was transferred into an anhydrous, N_2-filled glovebox. Under nitrogen atmosphere, $Cu(OTf)_2$ (181 mg, 0.500 mmol, 2.50 equiv.), and anhydrous MeCN (8.0 mL, *c* = 25 mM) were then added into the vial, resulting in a green suspension. The vial was sealed with a Teflon cap and placed 5 cm away from two purple LEDs (Kessil PR160-390 nm LEDs). The reaction mixture was stirred at a speed of 1200 rpm (Note: Fast stirring is essential due to the heterogeneity of the reaction mixture.) and irradiated for 16 h while maintaining the temperature at approximately 35 °C through cooling with a fan. Subsequently, the reaction mixture was transferred into a 50 mL round-bottom flask and evaporated to dryness. The residue was dissolved in EtOAc (40 mL) and filtered through a short pad of silica using EtOAc (50 mL) as the eluent to remove the majority of the copper salts. The filtrate was collected and concentrated under reduced pressure into a 20 mL borosilicate vial. To the 20 mL vial containing phenolic esters, a magnetic stir bar, THF (1.5 mL), MeOH (1.5 mL) and aqueous LiOH solution (1.0 M, 0.60 mL) were added. The reaction mixture was stirred at 40 °C and monitored by TLC until the phenolic esters were fully consumed. The reaction mixture was acidified by 1 M aqueous HCl to pH = 3–5. The solution is concentrated to about 10% of its volume under reduced pressure. The remaining suspension was dissolved in EtOAc (20 mL) and transferred into a separatory funnel containing 10 mL H_2O. The organic layer was collected, and the aqueous layer was further extracted with EtOAc (3 × ca. 10 mL). The combined organic layers were dried over Na_2SO_4, filtered, and the filtrate was concentrated under reduced pressure to roughly 0.1 mL then diluted with 0.50 mL CD_3CN. Dibromomethane (34.8 mg, 14.0 µL, 0.200 mmol, 1.00 equiv.) was added as an internal standard, and the yield of 4-methoxyphenol (**12**) was determined by ^1H NMR integration relative to the internal standard (54% yield, standard: δ 5.08 (s) ppm, and **12**: δ 6.74 (d, *J* = 6.1 Hz) ppm, Figure **S15**). The identity of the product was further confirmed by GCMS (Figure **S16** and HRMS-ESI analysis.

The yield of **12** reported in the manuscript (54%) represents an average of two runs [54% (above) and 55%].

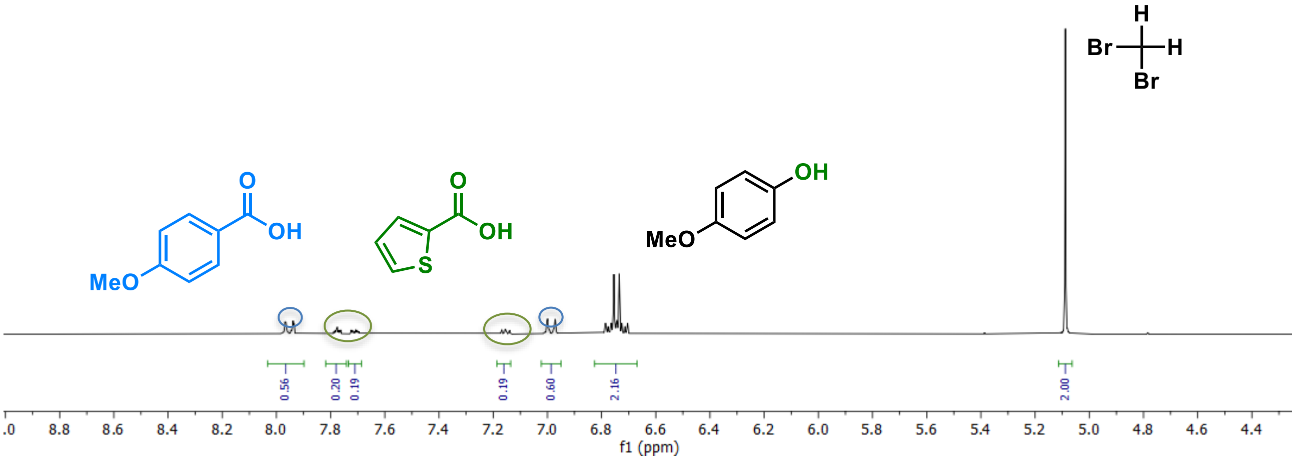

Figure S15. ^1H NMR of the resulting mixtures for product **12**

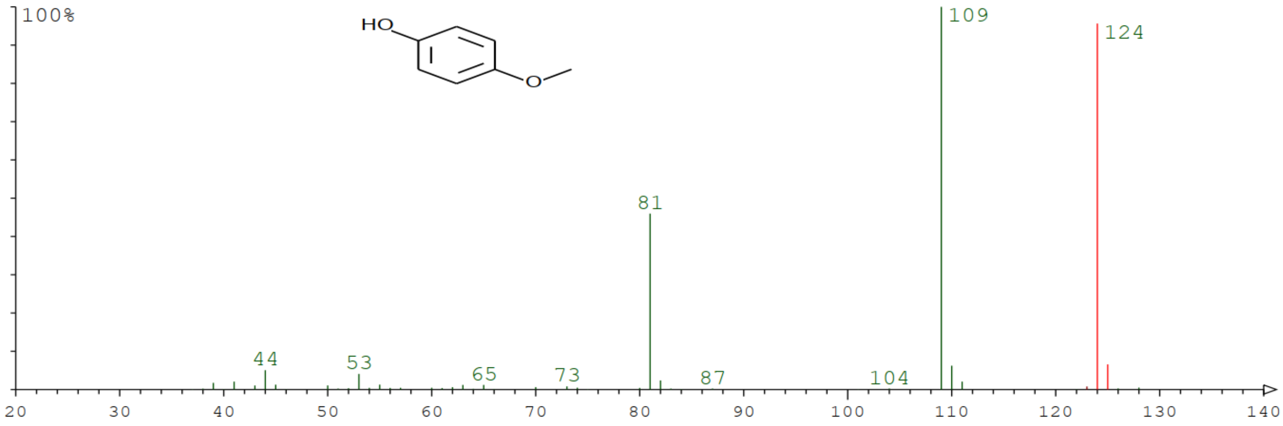

Figure S16. Mass spectrum of product **12** peak from GCMS

HRMS-ESI (m/z) calculated for $C_7H_8O_2^+$ $[M]^+$,124:0519; found,124:0521; deviation: –2 ppm.

3,5-Dimethylphenol (13)

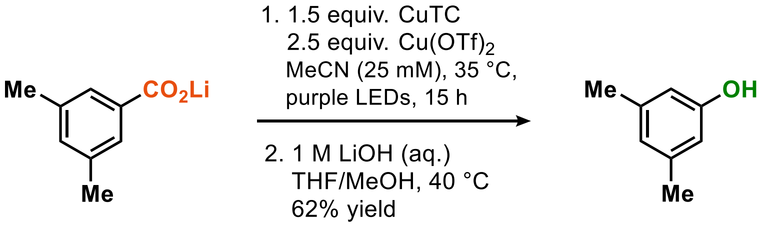

13

Under an ambient atmosphere, a 10 mL borosilicate vial equipped with a magnetic stir bar was charged with lithium 3,5-dimethylbenzoate (31.2 mg, 0.200 mmol, 1.00 equiv.) and copper (I) thiophene-2-carboxylate (57.2 mg, 0.300 mmol, 1.50 equiv.). The vial was transferred into an anhydrous, N_2-filled glovebox. Under nitrogen atmosphere, $Cu(OTf)_2$ (181 mg, 0.500 mmol, 2.50 equiv.), and anhydrous MeCN (8.0 mL, c = 25 mM) were then added into the vial, resulting in a green suspension. The vial was sealed with a Teflon cap and placed 5 cm away from two purple LEDs (Kessil PR160-390 nm LEDs). The reaction mixture was stirred at a speed of 1200 rpm (Note: Fast stirring is essential due to the heterogeneity of the reaction mixture.) and irradiated for 16 h while maintaining the temperature at approximately 35 °C through cooling with a fan. Subsequently, the reaction mixture was transferred into a 50 mL round-bottom flask and evaporated to dryness. The residue was dissolved in EtOAc (40 mL) and filtered through a short pad of silica using EtOAc (50 mL) as the eluent to remove the majority of the copper salts. The filtrate was collected and concentrated under reduced pressure into a 20 mL borosilicate vial. To the 20 mL vial containing phenolic esters, a magnetic stir bar, THF (1.5 mL), MeOH (1.5 mL) and aqueous LiOH solution (1.0 M, 0.60 mL) were added. The reaction mixture was stirred at 40 °C and monitored by TLC until the phenolic esters were fully consumed. The reaction mixture was acidified by 1 M aqueous HCl to pH = 3–5. The solution is concentrated to about 10% of its volume under reduced pressure. The remaining suspension was dissolved in DCM (20 mL) and transferred into a separatory funnel containing 10 mL H_2O. The organic layer was collected, and the aqueous layer was further extracted with DCM (3 × ca. 10 mL). The combined organic layers were dried over Na_2SO_4, filtered, and the filtrate was concentrated

under reduced pressure to roughly 0.1 mL then diluted with 0.50 mL CD_3CN. Dibromomethane (34.8 mg, 14.0 µL, 0.200 mmol, 1.00 equiv.) was added as an internal standard, and the yield of 3,5-dimethylphenol (**13**) was determined by ^1H NMR integration relative to the internal standard (62% yield, standard: δ 4.99 (s) ppm, and **13**: δ 6.61 (s) ppm, Figure **S17**). The identity of the product was further confirmed by GCMS (Figure **S18**) and HRMS-EI analysis.

The yield of **13** reported in the manuscript (62%) represents an average of two runs [62% (above) and 63%].

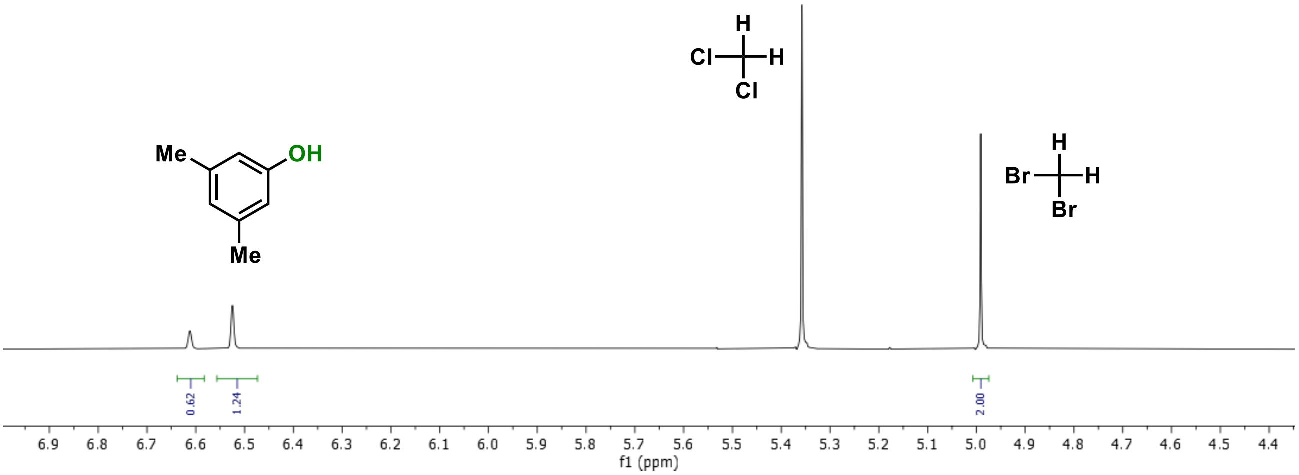

Figure S17. ^1H NMR of the resulting mixtures for product **13**

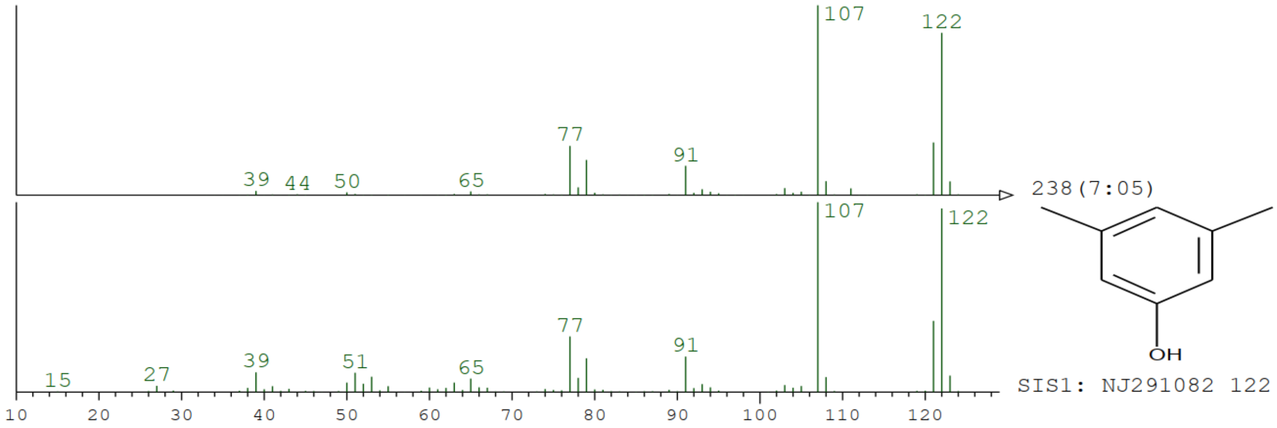

Figure S18. Mass spectrum of product **13** peak from GCMS

HRMS-EI (m/z) calculated for $C_8H_{10}O^+$ $[M]^+$,122.0726; found,122.0728; deviation: –2 ppm.

2,6-Dichloropyridin-4-ol (14)

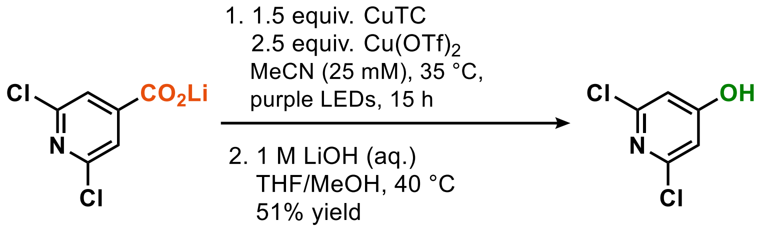

Under an ambient atmosphere, a 10 mL borosilicate vial equipped with a magnetic stir bar was charged with lithium 2,6-dichloroisonicotinateate (39.6 mg, 0.200 mmol, 1.00 equiv.) and copper (I) thiophene-2-carboxylate (57.2 mg, 0.300 mmol, 1.50 equiv.). The vial was transferred into an anhydrous, N_2-filled glovebox. Under nitrogen atmosphere, $Cu(OTf)_2$ (181 mg, 0.500 mmol, 2.50 equiv.), and anhydrous MeCN (8.0 mL, *c* = 25 mM) were then added into the vial, resulting in a green suspension. The vial was sealed with a Teflon cap and placed 5 cm away from two purple LEDs (Kessil PR160-390 nm LEDs). The reaction mixture was stirred at a speed of 1200 rpm (Note: Fast stirring is essential due to the heterogeneity of the reaction mixture.) and irradiated for 16 h while maintaining the temperature at approximately 35 °C through cooling with a fan. Subsequently, the reaction mixture was transferred into a 50 mL round-bottom flask and evaporated to dryness. The residue was dissolved in EtOAc (40 mL) and filtered through a short pad of silica using EtOAc (50 mL) as the eluent to remove the majority of the copper salts. The filtrate was collected and concentrated under reduced pressure into a 20 mL borosilicate vial. To the 20 mL vial containing phenolic esters, a magnetic stir bar, THF (1.5 mL), MeOH (1.5 mL) and aqueous LiOH solution (1.0 M, 0.60 mL) were added. The reaction mixture was stirred at 40 °C and monitored by TLC until the phenolic esters were fully consumed. The reaction mixture was acidified by addition of 1 M aqueous HCl to pH = 3–5 (roughly 0.70 mL). The solution is concentrated to about 10% or less of its volume under reduced pressure. The remaining suspension was dissolved in EtOAc (20 mL) and transferred into a separatory funnel containing 5 mL saturated aqueous $NaHCO_3$ and 5 mL H_2O. The organic layer was collected, and the aqueous layer was further extracted with EtOAc (3 × ca. 20 mL). The combined organic layers were dried over Na_2SO_4, filtered, and the filtrate was concentrated under reduced pressure to dryness. The residue was purified by chromatography on silica gel (pentane/EtOAc = 4/1, v/v) to afford 2,6-dichloropyridin-4-ol (**14**) (16.7 mg, 102 μmol, 51%) as a colorless solid.

R*f* =0.30 (pentane/EA = 4/1, v/v).

NMR Spectroscopy:

^{1}**H NMR** (500 MHz, CD_3CN, 298 K, δ): 6.83 (s, 2H).ppm.

^{13}**C NMR** (151 MHz, CD_3CN, 298 K, δ): 168.0, 151.6, 111.7 ppm.

HRMS-ESI (m/z) calculated for $C_5H_2Cl_2NO^-$ $[M-H]^-$, 161.9519; found, 161.9517; deviation: 1 ppm.

2-Hydroxyquinoxaline (15)

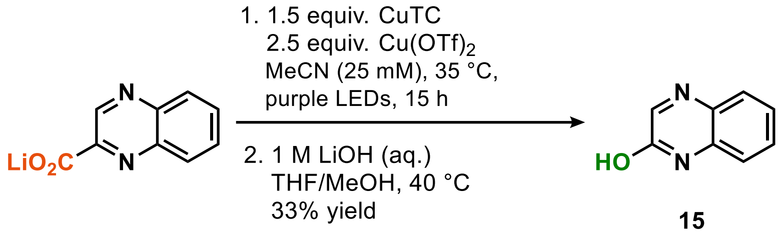

Under an ambient atmosphere, a 10 mL borosilicate vial equipped with a magnetic stir bar was charged with lithium quinoxaline-2-carboxylate (36.0 mg, 0.200 mmol, 1.00 equiv.) and copper (I) thiophene-2-carboxylate (57.2 mg, 0.300 mmol, 1.50 equiv.). The vial was transferred into an anhydrous, N_2-filled glovebox. Under nitrogen atmosphere, $Cu(OTf)_2$ (181 mg, 0.500 mmol, 2.50 equiv.), and anhydrous MeCN (8.0 mL, c = 25 mM) were then added into the vial, resulting in a green suspension. The vial was sealed with a Teflon cap and placed 5 cm away from two purple LEDs (Kessil PR160-390 nm LEDs). The reaction mixture was stirred at a speed of 1200 rpm (Note: Fast stirring is essential due to the heterogeneity of the reaction mixture.) and irradiated for 16 h while maintaining the temperature at approximately 35 °C through cooling with a fan. Subsequently, the reaction mixture was transferred into a 50 mL round-bottom flask and evaporated to dryness. The residue was dissolved in EtOAc (40 mL) and filtered through a short pad of silica using EtOAc (50 mL) as the eluent to remove the majority of the copper salts. The filtrate was collected and concentrated under reduced pressure into a 20 mL borosilicate vial. To the 20 mL vial containing phenolic esters, a magnetic stir bar, THF (1.5 mL), MeOH (1.5 mL) and aqueous LiOH solution (1.0 M, 0.60 mL) were added. The reaction mixture was stirred at 40 °C and monitored by TLC until the phenolic esters were fully consumed. The reaction mixture was acidified by addition of 1 M aqueous HCl to pH = 3–5 (roughly 0.70 mL). The solution is concentrated to about 10% or less of its volume under reduced pressure. The remaining suspension was dissolved in EtOAc (20 mL) and transferred into a separatory funnel containing 5 mL saturated aqueous $NaHCO_3$ and 5 mL H_2O. The organic layer was collected, and the aqueous layer was further extracted with EtOAc (3 × ca. 20 mL). The combined organic layers were dried over Na_2SO_4, filtered, and the filtrate was concentrated under reduced pressure to dryness. The residue was purified by chromatography on silica gel (pentane/EtOAc = 1/1, v/v) to afford 2-hydroxyquinoxaline (**15**) (9.6 mg, 66 μmol, 33%) as a yellow solid. (The compound is poorly dissolved in CD_3OD)

R*f* =0.20 (pentane/EA = 1/1, v/v).

NMR Spectroscopy:

^{1}H NMR (500 MHz, $(CD_3)_2SO$, 298 K, δ): 8.16 (s, 1H), 7.77 (d, J = 7.9 Hz, 1H), 7.54 (t, J = 7.7 Hz, 1H), 7.33–7.30 (m, 1H), 7.30–7.28 (m, 1H) ppm.

^{13}C NMR (126 MHz, $(CD_3)_2SO$, 298 K, δ): 155.0, 151.6, 132.0, 132.0, 130.7, 128.8, 123.2, 115.8 ppm.

HRMS-ESI (m/z) calculated for $C_8H_5N_2O^-$ [M-H]$^-$, 145.0407; found, 145.0408; deviation: –0.8 ppm.

4-Methyl-3-(morpholinosulfonyl)phenol (16)

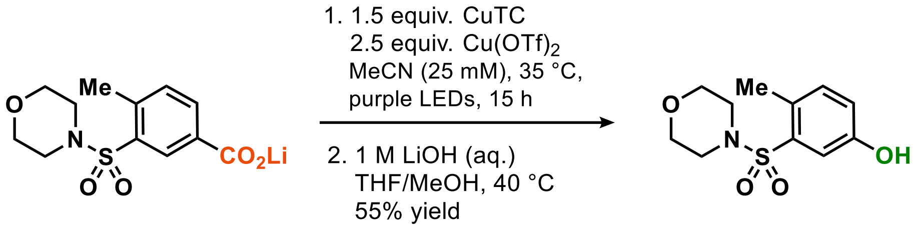

16

Under an ambient atmosphere, a 10 mL borosilicate vial equipped with a magnetic stir bar was charged with lithium 4-methyl-3-(morpholinosulfonyl)benzoate (58.2 mg, 0.200 mmol, 1.00 equiv.) and copper (I) thiophene-2-carboxylate (57.2 mg, 0.300 mmol, 1.50 equiv.). The vial was transferred into an anhydrous, N_2-filled glovebox. Under nitrogen atmosphere, $Cu(OTf)_2$ (181 mg, 0.500 mmol, 2.50 equiv.), and anhydrous MeCN (8.0 mL, *c* = 25 mM) were then added into the vial, resulting in a green suspension. The vial was sealed with a Teflon cap and placed 5 cm away from two purple LEDs (Kessil PR160-390 nm LEDs). The reaction mixture was stirred at a speed of 1200 rpm (Note: Fast stirring is essential due to the heterogeneity of the reaction mixture.) and irradiated for 16 h while maintaining the temperature at approximately 35 °C through cooling with a fan. Subsequently, the reaction mixture was transferred into a 50 mL round-bottom flask and evaporated to dryness. The residue was dissolved in EtOAc (40 mL) and filtered through a short pad of silica using EtOAc (50 mL) as the eluent to remove the majority of the copper salts. The filtrate was collected and concentrated under reduced pressure into a 20 mL borosilicate vial. To the 20 mL vial containing phenolic esters, a magnetic stir bar, THF (1.5 mL), MeOH (1.5 mL) and aqueous LiOH solution (1.0 M, 0.60 mL) were added. The reaction mixture was stirred at 40 °C and monitored by TLC until the phenolic esters were fully consumed. The reaction mixture was acidified by addition of 1 M aqueous HCl to pH = 3–5 (roughly 0.70 mL). The solution is concentrated to about 10% or less of its volume under reduced pressure. The remaining suspension was dissolved in EtOAc (20 mL) and transferred into a separatory funnel containing 5 mL saturated aqueous $NaHCO_3$ and 5 mL H_2O. The organic layer was collected, and the aqueous layer was further extracted with EtOAc (3 × ca. 20 mL). The combined organic layers were dried over Na_2SO_4, filtered, and the filtrate was concentrated under reduced pressure to dryness. The residue was purified by chromatography on silica gel (pentane/EtOAc = 5/1, v/v) to afford 4-methyl-3-(morpholinosulfonyl)phenol (**16**) (28.3 mg, 110 μmol, 55%) as a colorless solid.

R*f* =0.10 (pentane/EA = 5/1, v/v).

NMR Spectroscopy:

> **^{1}H NMR** (500 MHz, $CDCl_3$, 298 K, δ): 7.43 (d, *J* = 2.7 Hz, 1H), 7.19 (d, *J* = 8.2 Hz, 1H), 6.98 (dd, *J* = 8.3, 2.7 Hz, 1H), 6.34 (brs, 1H), 3.76–3.69 (m, 4H), 3.18–3.12 (m, 4H), 2.53 (s, 3H) ppm.
>
> **^{13}C NMR** (126 MHz, $CDCl_3$, 298 K, δ): 154.3, 134.4, 129.6, 120.7, 117.1, 66.4, 45.5, 19.9 ppm.

HRMS-EI (m/z) calculated for $C_{11}H_{15}NO_4S^+$ $[M]^+$, 257.0716; found, 257.0717; deviation: –0.2 ppm.

Probenicid derivative 17

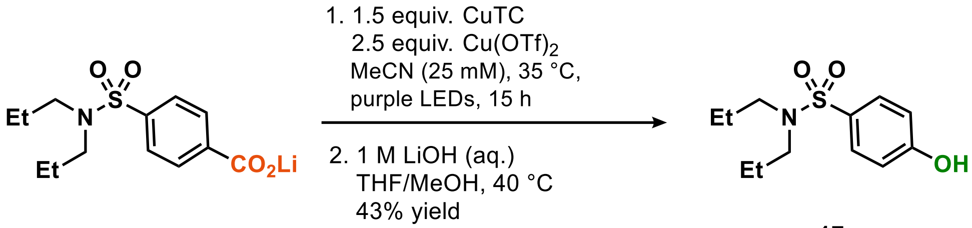

Under an ambient atmosphere, a 10 mL borosilicate vial equipped with a magnetic stir bar was charged with lithium probenecid salt (58.2 mg, 0.200 mmol, 1.00 equiv.) and copper (I) thiophene-2-carboxylate (57.2 mg, 0.300 mmol, 1.50 equiv.). The vial was transferred into an anhydrous, N_2-filled glovebox. Under nitrogen atmosphere, $Cu(OTf)_2$ (181 mg, 0.500 mmol, 2.50 equiv.), and anhydrous MeCN (8.0 mL, *c* = 25 mM) were then added into the vial, resulting in a green suspension. The vial was sealed with a Teflon cap and placed 5 cm away from two purple LEDs (Kessil PR160-390 nm LEDs). The reaction mixture was stirred at a speed of 1200 rpm (Note: Fast stirring is essential due to the heterogeneity of the reaction mixture.) and irradiated for 16 h while maintaining the temperature at approximately 35 °C through cooling with a fan. Subsequently, the reaction mixture was transferred into a 50 mL round-bottom flask and evaporated to dryness. The residue was dissolved in EtOAc (40 mL) and filtered through a short pad of silica using EtOAc (50 mL) as the eluent to remove the majority of the copper salts. The filtrate was collected and concentrated under reduced pressure into a 20 mL borosilicate vial. To the 20 mL vial containing phenolic esters, a magnetic stir bar, THF (1.5 mL), MeOH (1.5 mL) and aqueous LiOH solution (1.0 M, 0.60 mL) were added. The reaction mixture was stirred at 40 °C and monitored by TLC until the phenolic esters were fully consumed. The reaction mixture was acidified by addition of 1 M aqueous HCl to pH = 3–5 (roughly 0.70 mL). The solution is concentrated to about 10% or less of its volume under reduced pressure. The remaining suspension was dissolved in EtOAc (20 mL) and transferred into a separatory funnel containing 5 mL saturated aqueous $NaHCO_3$ and 5 mL H_2O. The organic layer was collected, and the aqueous layer was further extracted with EtOAc (3 × ca. 20 mL). The combined organic layers were dried over Na_2SO_4, filtered, and the filtrate was concentrated under reduced pressure to dryness. The residue was purified by chromatography on silica gel (pentane/EtOAc = 4/1, v/v) to afford the probenicid derivative **17** (22 mg, 86 μmol, 43%) as a colorless solid.

R*f* =0.10 (pentane/EA = 4/1, v/v).

NMR Spectroscopy:

^{1}H NMR (500 MHz, $CDCl_3$, 298 K, δ): 7.65 (d, *J* = 8.8 Hz, 2H), 6.92 (d, *J* = 8.7 Hz, 2H), 6.65 (brs, 1H), 3.07–3.03 (m, 4H), 1.62–1.46 (m, 4H), 0.86 (t, *J* = 7.4 Hz, 6H) ppm.

^{13}C NMR (126 MHz, $CDCl_3$, 298 K, δ): 159.9, 131.2, 129.4, 116.0, 50.1, 22.1, 11.3 ppm.

HRMS-ESI (m/z) calculated for $C_{12}H_{18}NO_3S^-$ [M-H]$^-$, 256.1013; found, 256.1015; deviation: –0.9 ppm.

4-Acetylphenol (18)

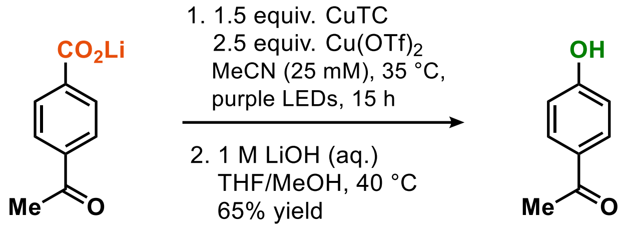

18

Under an ambient atmosphere, a 10 mL borosilicate vial equipped with a magnetic stir bar was charged with lithium 4-acetylbenzoate (34.0 mg, 0.200 mmol, 1.00 equiv.) and copper (I) thiophene-2-carboxylate (57.2 mg, 0.300 mmol, 1.50 equiv.). The vial was transferred into an anhydrous, N_2-filled glovebox. Under nitrogen atmosphere, $Cu(OTf)_2$ (181 mg, 0.500 mmol, 2.50 equiv.), and anhydrous MeCN (8.0 mL, *c* = 25 mM) were then added into the vial, resulting in a green suspension. The vial was sealed with a Teflon cap and placed 5 cm away from two purple LEDs (Kessil PR160-390 nm LEDs). The reaction mixture was stirred at a speed of 1200 rpm (Note: Fast stirring is essential due to the heterogeneity of the reaction mixture.) and irradiated for 16 h while maintaining the temperature at approximately 35 °C through cooling with a fan. Subsequently, the reaction mixture was transferred into a 50 mL round-bottom flask and evaporated to dryness. The residue was dissolved in EtOAc (40 mL) and filtered through a short pad of silica using EtOAc (50 mL) as the eluent to remove the majority of the copper salts. The filtrate was collected and concentrated under reduced pressure into a 20 mL borosilicate vial. To the 20 mL vial containing phenolic esters, a magnetic stir bar, THF (1.5 mL), MeOH (1.5 mL) and aqueous LiOH solution (1.0 M, 0.60 mL) were added. The reaction mixture was stirred at 40 °C and monitored by TLC until the phenolic esters were fully consumed. The reaction mixture was acidified by addition of 1 M aqueous HCl to pH = 3–5 (roughly 0.70 mL). The solution is concentrated to about 10% or less of its volume under reduced pressure. The remaining suspension was dissolved in EtOAc (20 mL) and transferred into a separatory funnel containing 5 mL saturated aqueous $NaHCO_3$ and 5 mL H_2O. The organic layer was collected, and the aqueous layer was further extracted with EtOAc (3 × ca. 20 mL). The combined organic layers were dried over Na_2SO_4, filtered, and the filtrate was concentrated under reduced pressure to dryness. The residue was purified by chromatography on silica gel (pentane/EtOAc = 4/1, v/v) to afford 4-acetylphenol (**18**) (17.7 mg, 130 μmol, 65%) as a colorless solid.

R*f* =0.30 (pentane/EA = 4/1, v/v).

NMR Spectroscopy:

^{1}H NMR (600 MHz, $CDCl_3$, 298 K, δ): 7.92 (d, *J* = 8.9 Hz, 2H), 7.50 (brs, 1H), 6.94 (d, *J* = 8.9 Hz, 2H), 2.59 (s, 3H) ppm.

^{13}C NMR (151 MHz, $CDCl_3$, 298 K, δ): 198.6, 161.3, 131.3, 129.8, 115.7, 26.5 ppm.

HRMS-EI (m/z) calculated for $C_8H_8O_2^+$ $[M]^+$, 136.0519; found, 136.0522; deviation: –2 ppm.

4-Sulfonamidephenol (19)

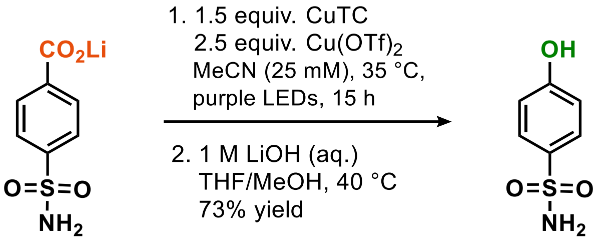

19

Under an ambient atmosphere, a 10 mL borosilicate vial equipped with a magnetic stir bar was charged with lithium 4-sulfamoylbenzoate (41.4 mg, 0.200 mmol, 1.00 equiv.) and copper (I) thiophene-2-carboxylate (57.2 mg, 0.300 mmol, 1.50 equiv.). The vial was transferred into an anhydrous, N_2-filled glovebox. Under nitrogen atmosphere, $Cu(OTf)_2$ (181 mg, 0.500 mmol, 2.50 equiv.), and anhydrous MeCN (8.0 mL, *c* = 25 mM) were then added into the vial, resulting in a green suspension. The vial was sealed with a Teflon cap and placed 5 cm away from two purple LEDs (Kessil PR160-390 nm LEDs). The reaction mixture was stirred at a speed of 1200 rpm (Note: Fast stirring is essential due to the heterogeneity of the reaction mixture.) and irradiated for 16 h while maintaining the temperature at approximately 35 °C through cooling with a fan. Subsequently, the reaction mixture was transferred into a 50 mL round-bottom flask and evaporated to dryness. The residue was dissolved in EtOAc (40 mL) and filtered through a short pad of silica using EtOAc (50 mL) as the eluent to remove the majority of the copper salts. The filtrate was collected and concentrated under reduced pressure into a 20 mL borosilicate vial. To the 20 mL vial containing phenolic esters, a magnetic stir bar, THF (1.5 mL), MeOH (1.5 mL) and aqueous LiOH solution (1.0 M, 0.60 mL) were added. The reaction mixture was stirred at 40 °C and monitored by TLC until the phenolic esters were fully consumed. The reaction mixture was acidified by addition of 1 M aqueous HCl to pH = 3–5 (roughly 0.70 mL). The solution is concentrated to about 10% or less of its volume under reduced pressure. The remaining suspension was dissolved in EtOAc (20 mL) and transferred into a separatory funnel containing 5 mL saturated aqueous $NaHCO_3$ and 5 mL H_2O. The organic layer was collected, and the aqueous layer was further extracted with EtOAc (3 × ca. 20 mL). The combined organic layers were dried over Na_2SO_4, filtered, and the filtrate was concentrated under reduced pressure to dryness. The residue was purified by chromatography on silica gel (pentane/EtOAc = 1/1, v/v) to afford 4-sulfonamidephenol (**19**) (25.2 mg, 146 μmol, 73%) as a colorless solid.

R*f* =0.30 (pentane/EA = 1/1, v/v).

NMR Spectroscopy:

^{1}H NMR (500 MHz, CD_3CN, 298 K, δ): 7.71 (d, *J* = 8.8 Hz, 2H), 7.69 (brs, 1H), 6.93 (d, *J* = 8.8 Hz, 2H), 5.50 (brs, 2H) ppm.

^{13}C NMR (126 MHz, CD_3CN, 298 K, δ): 161.3, 135.5, 129.3, 116.4 ppm.

HRMS-ESI (m/z) calculated for $C_6H_6NO_3S^-$ [M-H]$^-$, 172.0074; found, 172.0075; deviation: –0.7 ppm.

4-(Methylsulfonyl)phenol (20)

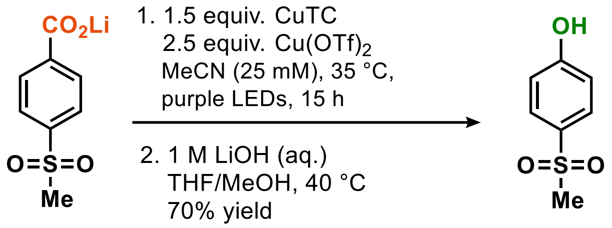

20

Under an ambient atmosphere, a 10 mL borosilicate vial equipped with a magnetic stir bar was charged with lithium 4-(methylsulfonyl)benzoate (41.2 mg, 0.200 mmol, 1.00 equiv.) and copper (I) thiophene-2-carboxylate (57.2 mg, 0.300 mmol, 1.50 equiv.). The vial was transferred into an anhydrous, N_2-filled glovebox. Under nitrogen atmosphere, $Cu(OTf)_2$ (181 mg, 0.500 mmol, 2.50 equiv.), and anhydrous MeCN (8.0 mL, c = 25 mM) were then added into the vial, resulting in a green suspension. The vial was sealed with a Teflon cap and placed 5 cm away from two purple LEDs (Kessil PR160-390 nm LEDs). The reaction mixture was stirred at a speed of 1200 rpm (Note: Fast stirring is essential due to the heterogeneity of the reaction mixture.) and irradiated for 16 h while maintaining the temperature at approximately 35 °C through cooling with a fan. Subsequently, the reaction mixture was transferred into a 50 mL round-bottom flask and evaporated to dryness. The residue was dissolved in EtOAc (40 mL) and filtered through a short pad of silica using EtOAc (50 mL) as the eluent to remove the majority of the copper salts. The filtrate was collected and concentrated under reduced pressure into a 20 mL borosilicate vial. To the 20 mL vial containing phenolic esters, a magnetic stir bar, THF (1.5 mL), MeOH (1.5 mL) and aqueous LiOH solution (1.0 M, 0.60 mL) were added. The reaction mixture was stirred at 40 °C and monitored by TLC until the phenolic esters were fully consumed. The reaction mixture was acidified by addition of 1 M aqueous HCl to pH = 3–5 (roughly 0.70 mL). The solution is concentrated to about 10% or less of its volume under reduced pressure. The remaining suspension was dissolved in EtOAc (20 mL) and transferred into a separatory funnel containing 5 mL saturated aqueous $NaHCO_3$ and 5 mL H_2O. The organic layer was collected, and the aqueous layer was further extracted with EtOAc (3 × ca. 20 mL). The combined organic layers were dried over Na_2SO_4, filtered, and the filtrate was concentrated under reduced pressure to dryness. The residue was purified by chromatography on silica gel (pentane/EtOAc = 2/1, v/v) to afford 4-(methylsulfonyl)phenol (**20**) (24.0 mg, 140 μmol, 70%) as a colorless solid.

R*f* =0.20 (pentane/EA = 2/1, v/v).

NMR Spectroscopy:

^{1}H NMR (300 MHz, $CDCl_3$, 298 K, δ): 7.77 (d, J = 8.8 Hz, 2H), 6.97 (d, J = 8.8 Hz, 2H), 3.06 (s, 3H) ppm.

^{13}C NMR (75 MHz, $CDCl_3$, 298 K, δ): 161.2, 131.5, 129.8, 116.4, 45.1 ppm.

HRMS-EI (m/z) calculated for $C_7H_8O_3S^+$ $[M]^+$, 172.0189; found, 172.0190; deviation: –1 ppm.

2-Naphthol (21)

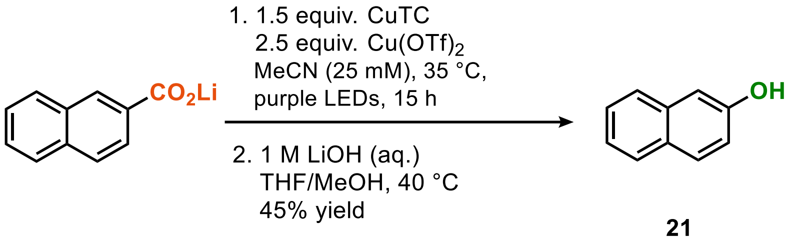

21

Under an ambient atmosphere, a 10 mL borosilicate vial equipped with a magnetic stir bar was charged with lithium 2-naphthoate (35.6 mg, 0.200 mmol, 1.00 equiv.) and copper (I) thiophene-2-carboxylate (57.2 mg, 0.300 mmol, 1.50 equiv.). The vial was transferred into an anhydrous, N_2-filled glovebox. Under nitrogen atmosphere, $Cu(OTf)_2$ (181 mg, 0.500 mmol, 2.50 equiv.), and anhydrous MeCN (8.0 mL, *c* = 25 mM) were then added into the vial, resulting in a green suspension. The vial was sealed with a Teflon cap and placed 5 cm away from two purple LEDs (Kessil PR160-390 nm LEDs). The reaction mixture was stirred at a speed of 1200 rpm (Note: Fast stirring is essential due to the heterogeneity of the reaction mixture.) and irradiated for 16 h while maintaining the temperature at approximately 35 °C through cooling with a fan. Subsequently, the reaction mixture was transferred into a 50 mL round-bottom flask and evaporated to dryness. The residue was dissolved in EtOAc (40 mL) and filtered through a short pad of silica using EtOAc (50 mL) as the eluent to remove the majority of the copper salts. The filtrate was collected and concentrated under reduced pressure into a 20 mL borosilicate vial. To the 20 mL vial containing phenolic esters, a magnetic stir bar, THF (1.5 mL), MeOH (1.5 mL) and aqueous LiOH solution (1.0 M, 0.60 mL) were added. The reaction mixture was stirred at 40 °C and monitored by TLC until the phenolic esters were fully consumed. The reaction mixture was acidified by addition of 1 M aqueous HCl to pH = 3–5 (roughly 0.70 mL). The solution is concentrated to about 10% or less of its volume under reduced pressure. The remaining suspension was dissolved in EtOAc (20 mL) and transferred into a separatory funnel containing 5 mL saturated aqueous $NaHCO_3$ and 5 mL H_2O. The organic layer was collected, and the aqueous layer was further extracted with EtOAc (3 × ca. 20 mL). The combined organic layers were dried over Na_2SO_4, filtered, and the filtrate was concentrated under reduced pressure to dryness. The residue was purified by chromatography on silica gel (pentane/EtOAc = 7/1, v/v) to afford 2-naphthol (**21**) (13 mg, 90 µmol, 45%) as a colorless solid.

R*f* =0.20 (pentane/EA = 7/1, v/v)

NMR Spectroscopy:

^{1}H NMR (600 MHz, $CDCl_3$, 298 K, δ): 7.81–7.74 (m, 2H), 7.71–7.67 (m, 1H), 7.44 (ddd, *J* = 8.2, 6.8, 1.3 Hz, 1H), 7.34 (ddd, *J* = 8.1, 6.8, 1.2 Hz, 1H), 7.18–7.14 (m, 1H), 7.11 (dd, *J* = 8.8, 2.6 Hz, 1H), 5.04 (s, 1H) ppm.

^{13}C NMR (151 MHz, $CDCl_3$, 298 K, δ): 153.4, 134.7, 130.0, 129.1, 127.9, 126.7, 126.5, 123.8, 117.9, 109.6 ppm.

HRMS-ESI (m/z) calculated for $C_{10}H_7O^-$ $[M-H]^-$, 143.0502; found, 143.0501; deviation: 0.7 ppm.

(4-Hydroxyphenyl)(morpholino)methanone (22)

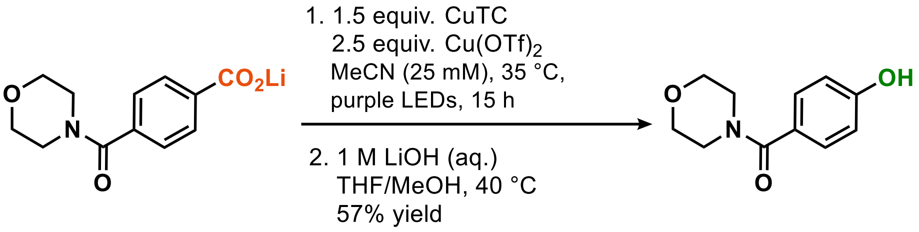

22

Under an ambient atmosphere, a 10 mL borosilicate vial equipped with a magnetic stir bar was charged with lithium 4-(4-morpholinylcarbonyl) benzoate (48.2 mg, 0.200 mmol, 1.00 equiv.) and copper (I) thiophene-2-carboxylate (57.2 mg, 0.300 mmol, 1.50 equiv.). The vial was transferred into an anhydrous, N_2-filled glovebox. Under nitrogen atmosphere, $Cu(OTf)_2$ (181 mg, 0.500 mmol, 2.50 equiv.), and anhydrous MeCN (8.0 mL, *c* = 25 mM) were then added into the vial, resulting in a green suspension. The vial was sealed with a Teflon cap and placed 5 cm away from two purple LEDs (Kessil PR160-390 nm LEDs). The reaction mixture was stirred at a speed of 1200 rpm (Note: Fast stirring is essential due to the heterogeneity of the reaction mixture.) and irradiated for 16 h while maintaining the temperature at approximately 35 °C through cooling with a fan. Subsequently, the reaction mixture was transferred into a 50 mL round-bottom flask and evaporated to dryness. The residue was dissolved in EtOAc (40 mL) and filtered through a short pad of silica using EtOAc (50 mL) as the eluent to remove the majority of the copper salts. The filtrate was collected and concentrated under reduced pressure into a 20 mL borosilicate vial. To the 20 mL vial containing phenolic esters, a magnetic stir bar, THF (1.5 mL), MeOH (1.5 mL) and aqueous LiOH solution (1.0 M, 0.60 mL) were added. The reaction mixture was stirred at 40 °C and monitored by TLC until the phenolic esters were fully consumed. The reaction mixture was acidified by addition of 1 M aqueous HCl to pH = 3–5 (roughly 0.70 mL). The solution is concentrated to about 10% or less of its volume under reduced pressure. The remaining suspension was dissolved in EtOAc (20 mL) and transferred into a separatory funnel containing 5 mL saturated aqueous $NaHCO_3$ and 5 mL H_2O. The organic layer was collected, and the aqueous layer was further extracted with EtOAc (3 × ca. 20 mL). The combined organic layers were dried over Na_2SO_4, filtered, and the filtrate was concentrated under reduced pressure to dryness. The residue was purified by chromatography on silica gel (pentane/EtOAc = 2/1, v/v) to afford (4-hydroxyphenyl)(morpholino)methanone (**22**) (23.6 mg, 114 μmol, 57%) as a colorless solid.

R*f* =0.10 (pentane/EA = 2/1, v/v).

NMR Spectroscopy:

^{1}**H NMR** (500 MHz, $CDCl_3$, 298 K, δ): 8.09 (brs, 1H), 7.23 (d, *J* = 8.6 Hz, 2H), 6.73 (d, *J* = 8.6 Hz, 2H),3.89–3.41 (m, 8H) ppm.

^{13}**C NMR** (126 MHz, $CDCl_3$, 298 K, δ): 171.5, 158.8, 129.3, 125.8, 115.7, 67.0. ppm (one carbon resonance was not identified).

HRMS-ESI (m/z) calculated for $C_{11}H_{12}NO_3^-$ [M-H]$^-$, 206.0823; found, 206.0823; deviation: –0.2 ppm.

4-(*trans*-4-Pentylcyclohexyl)phenol (23)

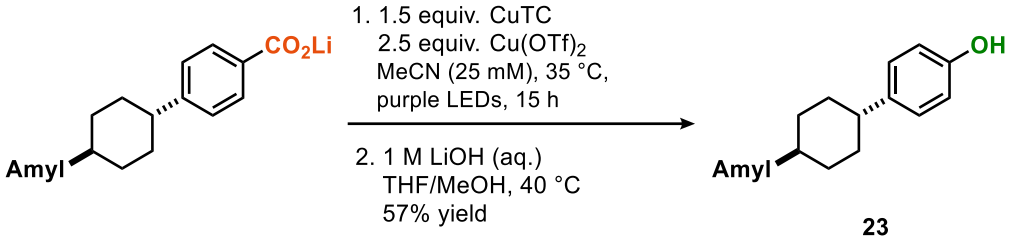

23

Under an ambient atmosphere, a 10 mL borosilicate vial equipped with a magnetic stir bar was charged with lithium 4-(4-pentylcyclohexyl)benzoate (56.4 mg, 0.200 mmol, 1.00 equiv.) and copper (I) thiophene-2-carboxylate (57.2 mg, 0.300 mmol, 1.50 equiv.). The vial was transferred into an anhydrous, N_2-filled glovebox. Under nitrogen atmosphere, $Cu(OTf)_2$ (181 mg, 0.500 mmol, 2.50 equiv.), and anhydrous MeCN (8.0 mL, *c* = 25 mM) were then added into the vial, resulting in a green suspension. The vial was sealed with a Teflon cap and placed 5 cm away from two purple LEDs (Kessil PR160-390 nm LEDs). The reaction mixture was stirred at a speed of 1200 rpm (Note: Fast stirring is essential due to the heterogeneity of the reaction mixture.) and irradiated for 16 h while maintaining the temperature at approximately 35 °C through cooling with a fan. Subsequently, the reaction mixture was transferred into a 50 mL round-bottom flask and evaporated to dryness. The residue was dissolved in EtOAc (40 mL) and filtered through a short pad of silica using EtOAc (50 mL) as the eluent to remove the majority of the copper salts. The filtrate was collected and concentrated under reduced pressure into a 20 mL borosilicate vial. To the 20 mL vial containing phenolic esters, a magnetic stir bar, THF (1.5 mL), MeOH (1.5 mL) and aqueous LiOH solution (1.0 M, 0.60 mL) were added. The reaction mixture was stirred at 40 °C and monitored by TLC until the phenolic esters were fully consumed. The reaction mixture was acidified by addition of 1 M aqueous HCl to pH = 3–5 (roughly 0.70 mL). The solution is concentrated to about 10% or less of its volume under reduced pressure. The remaining suspension was dissolved in EtOAc (20 mL) and transferred into a separatory funnel containing 5 mL saturated aqueous $NaHCO_3$ and 5 mL H_2O. The organic layer was collected, and the aqueous layer was further extracted with EtOAc (3 × ca. 20 mL). The combined organic layers were dried over Na_2SO_4, filtered, and the filtrate was concentrated under reduced pressure to dryness. The residue was purified by chromatography on silica gel (pentane/EtOAc = 10/1, v/v) to afford 4-(*trans*-4-pentylcyclohexyl)phenol (**23**) (28.0 mg, 114 µmol, 57%) as a colorless solid.

R*f* =0.20 (pentane/EA = 10/1, v/v).

NMR Spectroscopy:

^{1}H NMR (500 MHz, $CDCl_3$, 298 K, δ): 7.08 (d, *J* = 8.5 Hz, 2H), 6.76 (d, *J* = 8.5 Hz, 2H), 4.61 (brs, 1H), 2.40 (tt, *J* = 12.2, 3.2 Hz, 1H), 1.98–1.73 (m, 4H), 1.40 (qd, *J* = 13.6, 13.0, 3.6 Hz, 2H), 1.35–1.18 (m, 9H), 1.09–0.98 (m, 2H), 0.90 (t, *J* = 7.0 Hz, 3H) ppm.

^{13}C NMR (101 MHz, $CDCl_3$, 298 K, δ): 153.6, 140.5, 128.0, 115.2, 43.9, 37.5, 37.5, 34.7, 33.8, 32.4, 26.8, 22.9, 14.3 ppm.

HRMS-EI (m/z) calculated for $C_{17}H_{26}O^+$ [M]$^+$, 246.1978; found, 246.1979; deviation: –0.4 ppm.

Celecoxib derivative 24

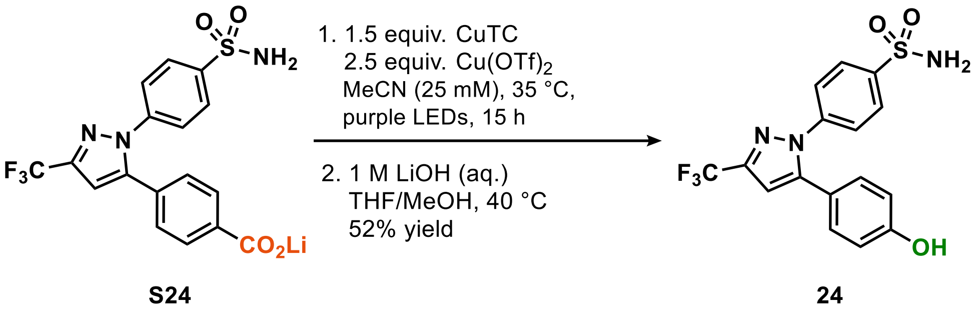

Under an ambient atmosphere, a 10 mL borosilicate vial equipped with a magnetic stir bar was charged with lithium celecoxib-derived benzoate (83.4 mg, 0.200 mmol, 1.00 equiv.) and copper (I) thiophene-2-carboxylate (57.2 mg, 0.300 mmol, 1.50 equiv.). The vial was transferred into an anhydrous, N_2-filled glovebox. Under nitrogen atmosphere, $Cu(OTf)_2$ (181 mg, 0.500 mmol, 2.50 equiv.), and anhydrous MeCN (8.0 mL, c = 25 mM) were then added into the vial, resulting in a green suspension. The vial was sealed with a Teflon cap and placed 5 cm away from two purple LEDs (Kessil PR160-390 nm LEDs). The reaction mixture was stirred at a speed of 1200 rpm (Note: Fast stirring is essential due to the heterogeneity of the reaction mixture.) and irradiated for 16 h while maintaining the temperature at approximately 35 °C through cooling with a fan. Subsequently, the reaction mixture was transferred into a 50 mL round-bottom flask and evaporated to dryness. The residue was dissolved in EtOAc (40 mL) and filtered through a short pad of silica using EtOAc (50 mL) as the eluent to remove the majority of the copper salts. The filtrate was collected and concentrated under reduced pressure into a 20 mL borosilicate vial. To the 20 mL vial containing phenolic esters, a magnetic stir bar, THF (1.5 mL), MeOH (1.5 mL) and aqueous LiOH solution (1.0 M, 0.60 mL) were added. The reaction mixture was stirred at 40 °C and monitored by TLC until the phenolic esters were fully consumed. The reaction mixture was acidified by addition of 1 M aqueous HCl to pH = 3–5 (roughly 0.70 mL). The solution is concentrated to about 10% or less of its volume under reduced pressure. The remaining suspension was dissolved in EtOAc (20 mL) and transferred into a separatory funnel containing 5 mL saturated aqueous $NaHCO_3$ and 5 mL H_2O. The organic layer was collected, and the aqueous layer was further extracted with EtOAc (3 × ca. 20 mL). The combined organic layers were dried over Na_2SO_4, filtered, and the filtrate was concentrated under reduced pressure to dryness. The residue was purified by chromatography on silica gel (pentane/EtOAc = 2/1, v/v) to afford the celecoxib derivative **24** (39.8 mg, 104 μmol, 52%) as a pale yellow solid.

R*f* =0.10 (pentane/EA = 2/1, v/v).

NMR Spectroscopy:

^{1}H NMR (500 MHz, CD_3CN, 298 K, δ): 7.88 (d, J = 8.7 Hz, 2H), 7.48 (d, J = 8.7 Hz, 2H), 7.38 (brs, 1H), 7.13 (d, J = 8.6 Hz, 2H), 6.86 (s, 1H), 6.81 (d, J = 8.6 Hz, 2H), 5.76 (s, 2H) ppm.

^{13}C NMR (75 MHz, CD_3CN, 298 K, δ): 159.0, 146.6, 143.9 (q, J = 37.9 Hz), 143.9, 143.2, 131.6, 128.1, 122.6 (q. 126.8, J = 266.3 Hz) 121.2, 116.5, 106.6 (q, J = 2.1 Hz) ppm.

^{19}F NMR (282 MHz, CD_3CN, 298 K, δ): –62.88 ppm.

HRMS-ESI (m/z) calculated for $C_{16}H_{11}F_3N_3O_3S^-$ [M-H]$^-$, 382.0478; found, 382.0481; deviation: –0.8 ppm.

4-Bromophenol (25)

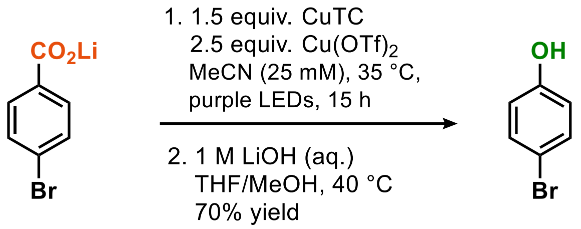

25

Under an ambient atmosphere, a 10 mL borosilicate vial equipped with a magnetic stir bar was charged with lithium 4-bromobenzoate (41.9 mg, 0.200 mmol, 1.00 equiv.) and copper (I) thiophene-2-carboxylate (57.2 mg, 0.300 mmol, 1.50 equiv.). The vial was transferred into an anhydrous, N_2-filled glovebox. Under nitrogen atmosphere, $Cu(OTf)_2$ (181 mg, 0.500 mmol, 2.50 equiv.), and anhydrous MeCN (8.0 mL, *c* = 25 mM) were then added into the vial, resulting in a green suspension. The vial was sealed with a Teflon cap and placed 5 cm away from two purple LEDs (Kessil PR160-390 nm LEDs). The reaction mixture was stirred at a speed of 1200 rpm (Note: Fast stirring is essential due to the heterogeneity of the reaction mixture.) and irradiated for 16 h while maintaining the temperature at approximately 35 °C through cooling with a fan. Subsequently, the reaction mixture was transferred into a 50 mL round-bottom flask and evaporated to dryness. The residue was dissolved in EtOAc (40 mL) and filtered through a short pad of silica using EtOAc (50 mL) as the eluent to remove the majority of the copper salts. The filtrate was collected and concentrated under reduced pressure into a 20 mL borosilicate vial. To the 20 mL vial containing phenolic esters, a magnetic stir bar, THF (1.5 mL), MeOH (1.5 mL) and aqueous LiOH solution (1.0 M, 0.60 mL) were added. The reaction mixture was stirred at 40 °C and monitored by TLC until the phenolic esters were fully consumed. The reaction mixture was acidified by addition of 1 M aqueous HCl to pH = 3–5 (roughly 0.70 mL). The solution is concentrated to about 10% or less of its volume under reduced pressure. The remaining suspension was dissolved in DCM (20 mL) and transferred into a separatory funnel containing 5 mL saturated aqueous $NaHCO_3$ and 5 mL H_2O. The organic layer was collected, and the aqueous layer was further extracted with DCM (3 × ca. 20 mL). The combined organic layers were dried over Na_2SO_4, filtered, and the filtrate was concentrated under reduced pressure to dryness. The residue was purified by chromatography on silica gel (pentane/DCM = 2/1, v/v) to afford 4-bromophenol (**25**) (24.0 mg, 173 μmol, 70%) as a colorless solid.

R*f* =0.10 (pentane/DCM = 2/1, v/v).

NMR Spectroscopy:

^{1}**H NMR** (500 MHz, CD_2Cl_2, 298 K, δ): 7.34 (d, *J* = 8.9 Hz, 2H), 6.74 (d, *J* = 8.9 Hz, 2H), 5.12 (s, 1H) ppm.

^{13}**C NMR** (151 MHz, CD_3CN, 298 K, δ): 155.3, 132.8, 117.6, 113.0 ppm.

HRMS-EI (m/z) calculated for $C_6H_5OBr^+$ [M]$^+$, 171.9518; found, 171.9519; deviation: –0.4 ppm.

4-Nitrophenol (26)

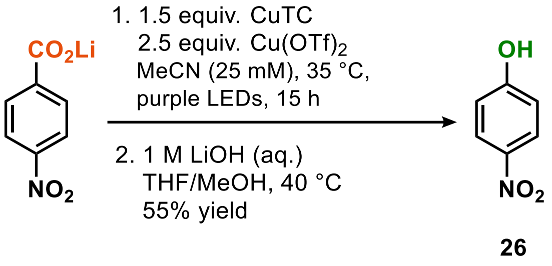

Under an ambient atmosphere, a 10 mL borosilicate vial equipped with a magnetic stir bar was charged with lithium 4-nitrobenzoate (34.6 mg, 0.200 mmol, 1.00 equiv.) and copper (I) thiophene-2-carboxylate (57.2 mg, 0.300 mmol, 1.50 equiv.). The vial was transferred into an anhydrous, N_2-filled glovebox. Under nitrogen atmosphere, $Cu(OTf)_2$ (181 mg, 0.500 mmol, 2.50 equiv.), and anhydrous MeCN (8.0 mL, *c* = 25 mM) were then added into the vial, resulting in a green suspension. The vial was sealed with a Teflon cap and placed 5 cm away from two purple LEDs (Kessil PR160-390 nm LEDs). The reaction mixture was stirred at a speed of 1200 rpm (Note: Fast stirring is essential due to the heterogeneity of the reaction mixture.) and irradiated for 16 h while maintaining the temperature at approximately 35 °C through cooling with a fan. Subsequently, the reaction mixture was transferred into a 50 mL round-bottom flask and evaporated to dryness. The residue was dissolved in EtOAc (40 mL) and filtered through a short pad of silica using EtOAc (50 mL) as the eluent to remove the majority of the copper salts. The filtrate was collected and concentrated under reduced pressure into a 20 mL borosilicate vial. To the 20 mL vial containing phenolic esters, a magnetic stir bar, THF (1.5 mL), MeOH (1.5 mL) and aqueous LiOH solution (1.0 M, 0.60 mL) were added. The reaction mixture was stirred at 40 °C and monitored by TLC until the phenolic esters were fully consumed. The reaction mixture was acidified by addition of 1 M aqueous HCl to pH = 3–5 (roughly 0.70 mL). The solution is concentrated to about 10% or less of its volume under reduced pressure. The remaining suspension was dissolved in EtOAc (20 mL) and transferred into a separatory funnel containing 5 mL saturated aqueous $NaHCO_3$ and 5 mL H_2O. The organic layer was collected, and the aqueous layer was further extracted with EtOAc (3 × ca. 20 mL). The combined organic layers were dried over Na_2SO_4, filtered, and the filtrate was concentrated under reduced pressure to dryness. The residue was purified by chromatography on silica gel (pentane/EtOAc = 4/1, v/v) to afford 4-nitrophenol (**26**) (15.3 mg, 110 μmol, 55%) as a colorless solid.

R*f* =0.3 (pentane/EA = 4/1, v/v).

NMR Spectroscopy:

^{1}H NMR (300 MHz, CD_3CN, 298 K, δ): 8.12 (d, *J* = 9.2 Hz, 1H), 6.93 (d, *J* = 9.2 Hz, 1H), 2.26 (s, 3H).ppm.

^{13}C NMR (75 MHz, CD_3CN, 298 K, δ): 164.0, 127.1, 116.6 ppm. (one carbon missing)

HRMS-EI (m/z) calculated for $C_6H_5NO_3^+$ $[M]^+$, 139.0264; found, 139.0266; deviation: –2 ppm.

2-Fluorophenol (27)

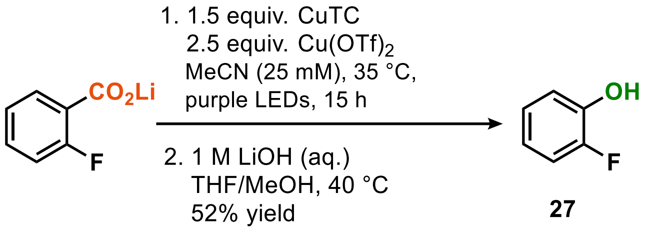

27

Under an ambient atmosphere, a 10 mL borosilicate vial equipped with a magnetic stir bar was charged with lithium 2-fluorobenzoate (29.2 mg, 0.200 mmol, 1.00 equiv.) and copper (I) thiophene-2-carboxylate (57.2 mg, 0.300 mmol, 1.50 equiv.). The vial was transferred into an anhydrous, N_2-filled glovebox. Under nitrogen atmosphere, $Cu(OTf)_2$ (181 mg, 0.500 mmol, 2.50 equiv.), and anhydrous MeCN (8.0 mL, c = 25 mM) were then added into the vial, resulting in a green suspension. The vial was sealed with a Teflon cap and placed 5 cm away from two purple LEDs (Kessil PR160-390 nm LEDs). The reaction mixture was stirred at a speed of 1200 rpm (Note: Fast stirring is essential due to the heterogeneity of the reaction mixture.) and irradiated for 16 h while maintaining the temperature at approximately 35 °C through cooling with a fan. Subsequently, the reaction mixture was transferred into a 50 mL round-bottom flask and evaporated to dryness. The residue was dissolved in EtOAc (40 mL) and filtered through a short pad of silica using EtOAc (50 mL) as the eluent to remove the majority of the copper salts. The filtrate was collected and concentrated under reduced pressure into a 20 mL borosilicate vial. To the 20 mL vial containing phenolic esters, a magnetic stir bar, THF (1.5 mL), MeOH (1.5 mL) and aqueous LiOH solution (1.0 M, 0.60 mL) were added. The reaction mixture was stirred at 40 °C and monitored by TLC until the phenolic esters were fully consumed. The reaction mixture was acidified by 1 M aqueous HCl to pH = 3–5. 4-fluoroanisole (25.2 mg, 22.5 µL, 0.200 mmol, 1.00 equiv.) was added as an internal standard, and the yield of 2-fluorophenol (**27**) was determined by ^{19}F NMR integration relative to the internal standard (50% yield, standard: δ –126:29 (m) ppm, and **27**: δ –139:29 (m) ppm, Figure **S19**). The identity of the product was further confirmed by GCMS (Figure **S20**) and HRMS-EI analysis

The yield of **25** reported in the manuscript (52%) represents an average of two runs [50% (above) and 54%].

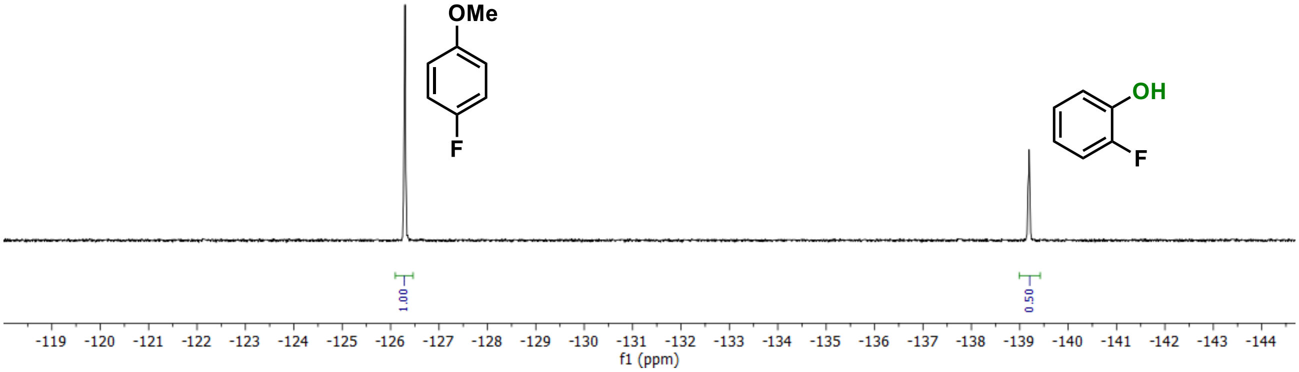

Figure S19. ^{19}F NMR of the resulting mixtures for product **27**

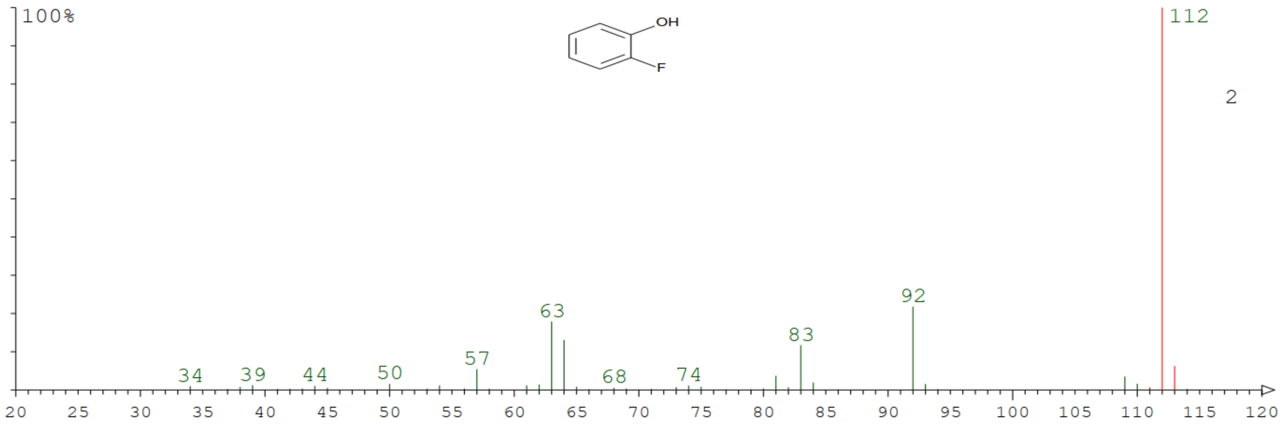

Figure S20. Mass spectrum of product **27** peak from GCMS

HRMS-EI (m/z) calculated for $C_6H_5OF^+$ $[M]^+$, 112.0319; found,112.0320; deviation: –2 ppm.

6-Hydroxy-1-tetralone (28)

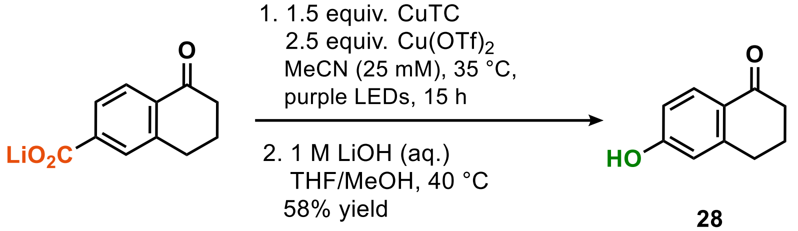

28

Under an ambient atmosphere, a 10 mL borosilicate vial equipped with a magnetic stir bar was charged with lithium 5-oxo-5,6,7,8-tetrahydronaphthalene-2-carboxylate (39.3 mg, 0.200 mmol, 1.00 equiv.) and copper (I) thiophene-2-carboxylate (57.2 mg, 0.300 mmol, 1.50 equiv.). The vial was transferred into an anhydrous, N_2-filled glovebox. Under nitrogen atmosphere, $Cu(OTf)_2$ (181 mg, 0.500 mmol, 2.50 equiv.), and anhydrous MeCN (8.0 mL, *c* = 25 mM) were then added into the vial, resulting in a green suspension. The vial was sealed with a Teflon cap and placed 5 cm away from two purple LEDs (Kessil PR160-390 nm LEDs). The reaction mixture was stirred at a speed of 1200 rpm (Note: Fast stirring is essential due to the heterogeneity of the reaction mixture.) and irradiated for 16 h while maintaining the temperature at approximately 35 °C through cooling with a fan. Subsequently, the reaction mixture was transferred into a 50 mL round-bottom flask and evaporated to dryness. The residue was dissolved in EtOAc (40 mL) and filtered through a short pad of silica using EtOAc (50 mL) as the eluent to remove the majority of the copper salts. The filtrate was collected and concentrated under reduced pressure into a 20 mL borosilicate vial. To the 20 mL vial containing phenolic esters, a magnetic stir bar, THF (1.5 mL), MeOH (1.5 mL) and aqueous LiOH solution (1.0 M, 0.60 mL) were added. The reaction mixture was stirred at 40 °C and monitored by TLC until the phenolic esters were fully consumed. The reaction mixture was acidified by addition of 1 M aqueous HCl to pH = 3–5 (roughly 0.70 mL). The solution is concentrated to about 10% or less of its volume under reduced pressure. The remaining suspension was dissolved in EtOAc (20 mL) and transferred into a separatory funnel containing 5 mL saturated aqueous $NaHCO_3$ and 5 mL H_2O. The organic layer was collected, and the aqueous layer was further extracted with EtOAc (3 × ca. 20 mL). The combined organic layers were dried over Na_2SO_4, filtered, and the filtrate was

concentrated under reduced pressure to dryness. The residue was purified by chromatography on silica gel (pentane/EtOAc = 4/1, v/v) to afford 6-hydroxy-1-tetralone (**28**) (18.7 mg, 116 μmol, 58%) as a colorless solid.

R*f* =0.30 (pentane/EA = 4/1, v/v).

NMR Spectroscopy:

^{1}**H NMR** (500 MHz, $CDCl_3$, 298 K, δ): 7.98 (d, *J* = 8.7 Hz, 1H), 7.55 (brs, 1H), 6.82 (dd, *J* = 8.6, 2.5 Hz, 1H), 6.73 (d, *J* = 2.5 Hz, 1H), 2.90 (t, *J* = 6.1 Hz, 2H), 2.63 (dd, *J* = 7.2, 5.8 Hz, 2H), 2.11 (p, *J* = 6.4 Hz, 2H) ppm.

^{13}**C NMR** (151 MHz, $CDCl_3$, 298 K, δ): 198.4, 161.1, 147.8, 130.3, 126.1, 114.7, 114.7, 39.0, 30.1, 23.4 ppm.

HRMS-ESI (m/z) calculated for $C_{10}H_9O_2^-$ $[M-H]^-$, 161.0608; found, 161.0609; deviation: –0.7 ppm.

2,2'-(5-Hydroxy-1,3-phenylene)bis(2-methylpropanenitrile) (29)

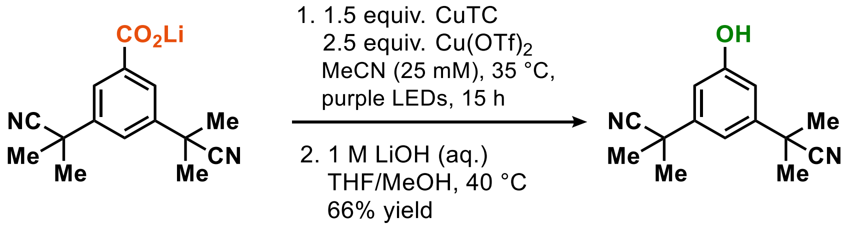

29

Under an ambient atmosphere, a 10 mL borosilicate vial equipped with a magnetic stir bar was charged with lithium 3,5-bis(2-cyanopropan-2-yl)benzoate (52.4 mg, 0.200 mmol, 1.00 equiv.) and copper (I) thiophene-2-carboxylate (57.2 mg, 0.300 mmol, 1.50 equiv.). The vial was transferred into an anhydrous, N_2-filled glovebox. Under nitrogen atmosphere, $Cu(OTf)_2$ (181 mg, 0.500 mmol, 2.50 equiv.), and anhydrous MeCN (8.0 mL, *c* = 25 mM) were then added into the vial, resulting in a green suspension. The vial was sealed with a Teflon cap and placed 5 cm away from two purple LEDs (Kessil PR160-390 nm LEDs). The reaction mixture was stirred at a speed of 1200 rpm (Note: Fast stirring is essential due to the heterogeneity of the reaction mixture.) and irradiated for 16 h while maintaining the temperature at approximately 35 °C through cooling with a fan. Subsequently, the reaction mixture was transferred into a 50 mL round-bottom flask and evaporated to dryness. The residue was dissolved in EtOAc (40 mL) and filtered through a short pad of silica using EtOAc (50 mL) as the eluent to remove the majority of the copper salts. The filtrate was collected and concentrated under reduced pressure into a 20 mL borosilicate vial. To the 20 mL vial containing phenolic esters, a magnetic stir bar, THF (1.5 mL), MeOH (1.5 mL) and aqueous LiOH solution (1.0 M, 0.60 mL) were added. The reaction mixture was stirred at 40 °C and monitored by TLC until the phenolic esters were fully consumed. The reaction mixture was acidified by addition of 1 M aqueous HCl to pH = 3–5 (roughly 0.70 mL). The solution is concentrated to dryness. The residue was purified by chromatography on silica gel (DCM/EtOAc = 16/1, v/v) to afford 2,2'-(5-Hydroxy-1,3-phenylene)bis(2-methylpropanenitrile) (**29**) (30.0 mg, 131 μmol, 66%) as a colorless solid.

R*f* =0.4 (DCM/EA = 16/1, v/v).

NMR Spectroscopy:

^{1}H NMR (500 MHz, $CDCl_3$, 298 K, δ): 7.03 (t, *J* = 1.7 Hz, 1H), 6.96 (d, *J* = 1.6 Hz, 2H), 6.36 (s, 1H), 1.73 (s, 12H). ppm.

^{13}C NMR (126 MHz, $CDCl_3$, 298 K, δ): 157.2, 144.0, 124.2, 113.1, 112.3, 37.5, 29.2. ppm.

HRMS-EI (m/z) calculated for $C_{14}H_{16}N_2O^+$ $[M]^+$, 228.1257; found, 228.1260; deviation: –1 ppm.

4-Phenylphenol (30)

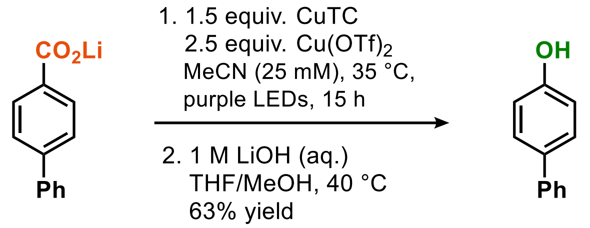

30

Under an ambient atmosphere, a 10 mL borosilicate vial equipped with a magnetic stir bar was charged with 4-biphenylcarboxylic acid lithium salt (40.8 mg, 0.200 mmol, 1.00 equiv.) and copper (I) thiophene-2-carboxylate (57.2 mg, 0.300 mmol, 1.50 equiv.). The vial was transferred into an anhydrous, N_2-filled glovebox. Under nitrogen atmosphere, $Cu(OTf)_2$ (181 mg, 0.500 mmol, 2.50 equiv.), and anhydrous MeCN (8.0 mL, *c* = 25 mM) were then added into the vial, resulting in a green suspension. The vial was sealed with a Teflon cap and placed 5 cm away from two purple LEDs (Kessil PR160-390 nm LEDs). The reaction mixture was stirred at a speed of 1200 rpm (Note: Fast stirring is essential due to the heterogeneity of the reaction mixture.) and irradiated for 16 h while maintaining the temperature at approximately 35 °C through cooling with a fan. Subsequently, the reaction mixture was transferred into a 50 mL round-bottom flask and evaporated to dryness. The residue was dissolved in EtOAc (40 mL) and filtered through a short pad of silica using EtOAc (50 mL) as the eluent to remove the majority of the copper salts. The filtrate was collected and concentrated under reduced pressure into a 20 mL borosilicate vial. To the 20 mL vial containing phenolic esters, a magnetic stir bar, THF (1.5 mL), MeOH (1.5 mL) and aqueous LiOH solution (1.0 M, 0.60 mL) were added. The reaction mixture was stirred at 40 °C and monitored by TLC until the phenolic esters were fully consumed. The reaction mixture was acidified by addition of 1 M aqueous HCl to pH = 3–5 (roughly 0.70 mL). The solution is concentrated to about 10% or less of its volume under reduced pressure. The remaining suspension was dissolved in Et_2O (20 mL) and transferred into a separatory funnel containing 5 mL saturated aqueous $NaHCO_3$ and 5 mL H_2O. The organic layer was collected, and the aqueous layer was further extracted with Et_2O (3 × ca. 20 mL). The combined organic layers were dried over Na_2SO_4, filtered, and the filtrate was concentrated under reduced pressure to dryness. The residue was purified by chromatography on silica gel (pentane/Et_2O = 4/1, v/v) to afford 4-phenylphenol (**30**) (21.4 mg, 126 μmol, 63%) as a colorless solid.

R*f* =0.30 (pentane/Et_2O = 4/1, v/v)

NMR Spectroscopy:

^{1}H NMR (500 MHz, CD_3CN, 298 K, δ): 7.57 (dd, *J* = 8.4, 1.2 Hz, 2H), 7.49 (d, *J* = 8.7 Hz, 2H), 7.41 (t, *J* = 7.7 Hz, 2H), 7.34–7.27 (m, 1H), 7.03 (brs, 1H), 6.89 (d, *J* = 8.7 Hz, 2H) ppm.

^{13}C NMR (126 MHz, CD_3CN, 298 K, δ): 157.6, 141.7, 133.6, 129.8, 129.1, 127.6, 127.3, 116.6 ppm.

HRMS-EI (m/z) calculated for $C_{12}H_{10}O^+$ $[M]^+$, 170.0726; found, 170.0728; deviation: –0.9 ppm.

Triclosan derivative 31

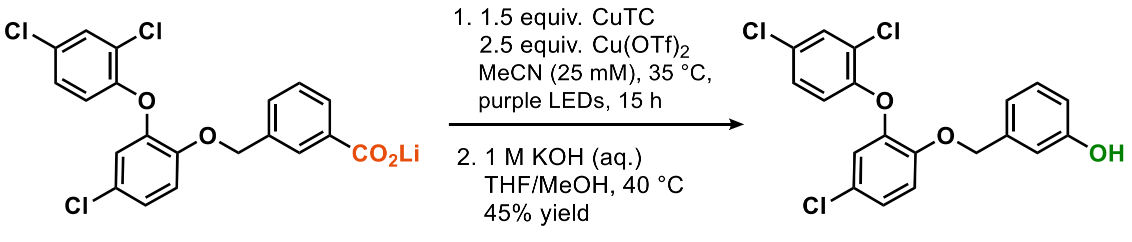

Under an ambient atmosphere, a 10 mL borosilicate vial equipped with a magnetic stir bar was charged with lithium triclosan-derived benzoate (85.9 mg, 0.200 mmol, 1.00 equiv.) and copper (I) thiophene-2-carboxylate (57.2 mg, 0.300 mmol, 1.50 equiv.). The vial was transferred into an anhydrous, N_2-filled glovebox. Under nitrogen atmosphere, $Cu(OTf)_2$ (181 mg, 0.500 mmol, 2.50 equiv.), and anhydrous MeCN (8.0 mL, *c* = 25 mM) were then added into the vial, resulting in a green suspension. The vial was sealed with a Teflon cap and placed 5 cm away from two purple LEDs (Kessil PR160-390 nm LEDs). The reaction mixture was stirred at a speed of 1200 rpm (Note: Fast stirring is essential due to the heterogeneity of the reaction mixture.) and irradiated for 16 h while maintaining the temperature at approximately 35 °C through cooling with a fan. Subsequently, the reaction mixture was transferred into a 50 mL round-bottom flask and evaporated to dryness. The residue was dissolved in EtOAc (40 mL) and filtered through a short pad of silica using EtOAc (50 mL) as the eluent to remove the majority of the copper salts. The filtrate was collected and concentrated under reduced pressure into a 20 mL borosilicate vial. To the 20 mL vial containing phenolic esters, a magnetic stir bar, THF (1.5 mL), MeOH (1.5 mL) and aqueous KOH solution (1.0 M, 0.60 mL) were added. The reaction mixture was stirred at 40 °C and monitored by TLC until the phenolic esters were fully consumed. The reaction mixture was acidified by addition of 1 M aqueous HCl to pH = 3–5 (roughly 0.70 mL). The solution is concentrated to dryness. The residue was purified by chromatography on silica gel (pentane/EtOAc = 7/1, v/v) to afford triclosan derivative **31** (35.6 mg, 110 µmol, 55%) as a colorless solid.

R*f* =0.3 (pentane/EA = 7/1, v/v).

NMR Spectroscopy:

^{1}H NMR (500 MHz, CD_3CN, 298 K, δ): 7.50 (d, *J* = 2.6 Hz, 1H), 7.22–7.16 (m, 2H), 7.13 (t, *J* = 8.1 Hz, 1H), 7.03 (d, *J* = 8.5 Hz, 1H), 7.00 (dd, *J* = 8.6, 2.3 Hz, 1H), 6.97 (brs, 1H), 6.74–6.72 (m, 2H), 6.67–6.66 (m, 2H), 4.99 (s, 2H). ppm.

^{13}C NMR (126 MHz, CD_3CN, 298 K, δ): 158.0, 153.4, 151.6, 144.0, 138.8, 131.2, 130.9, 130.6, 129.0, 128.5, 124.8, 123.3, 122.3, 119.7, 119.1, 116.4, 115.9, 115.1, 71.5. ppm.

HRMS-ESI (m/z) calculated for $C_{19}H_{12}Cl_3O_3^-$ [M-H]$^-$, 392.9857; found, 392.9859; deviation: –0.4 ppm.

Reaction performed outside of a glovebox by using Schlenk techniques

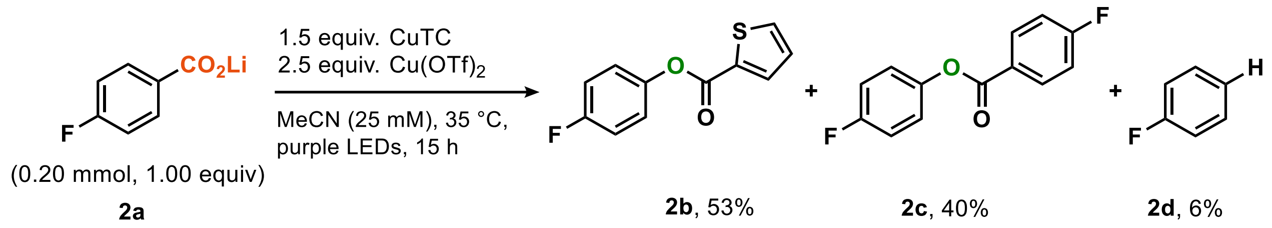

Under argon atmosphere, an oven-dried 20 mL Schlenk tube equipped with a rubber stopper and a magnetic stir bar was charged with lithium 4-fluorobenzoate (29.2 mg, 0.200 mmol, 1.00 equiv.), copper (I) thiophene-2-carboxylate (57.2 mg, 0.300 mmol, 1.50 equiv.) and $Cu(OTf)_2$ (181 mg, 0.500 mmol, 2.50 equiv.). The Schlenk tube was evacuated under low pressure (ca. 3 × 10^{-1} mbar) with oil pump then backfilled with Ar for 3 times. Anhydrous MeCN (8.0 mL, *c* = 25 mM) was then added via a syringe. The tube was sealed with a rubber stopper and placed 5 cm away from two purple LEDs (Kessil PR160-390 nm LEDs). The reaction mixture was stirred at a speed of 1200 rpm (Note: Fast stirring is essential due to the heterogeneity of the reaction mixture.) and irradiated for 16 h while maintaining the temperature at approximately 35 °C through cooling with a fan. 2-fluorotoluene (22.0 mg, 22.0 µL, 0.200 mmol, 1.00 equiv.) was added as an internal standard. The reaction mixture was diluted with CD_3CN, and the ^{19}F NMR spectrum was measured. The yields of 4-fluorophenyl thiophene-2-carboxylate (**2b**, 53%, δ –118.39 (m) ppm) and 4-fluorophenyl 4-fluorobenzoate (**2c**, 40%, δ –118.57, 106.36 (m) ppm) 4-fluorobenzene (**2d**, 6%, δ –114.82 (m) ppm)were determined by ^{19}F NMR integration relative to the internal standard (δ –119.32 (m) ppm), respectively.

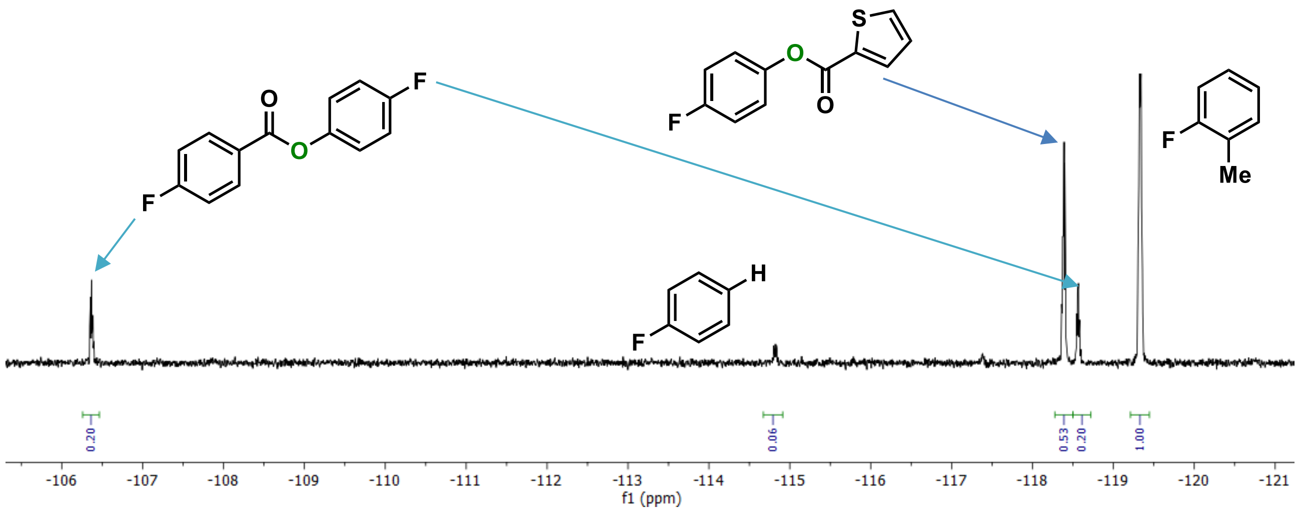

Figure S21. ^{19}F NMR of the resulting mixture of **2b**, **2c** and **2d** using Schlenk techniques

Failed substrates

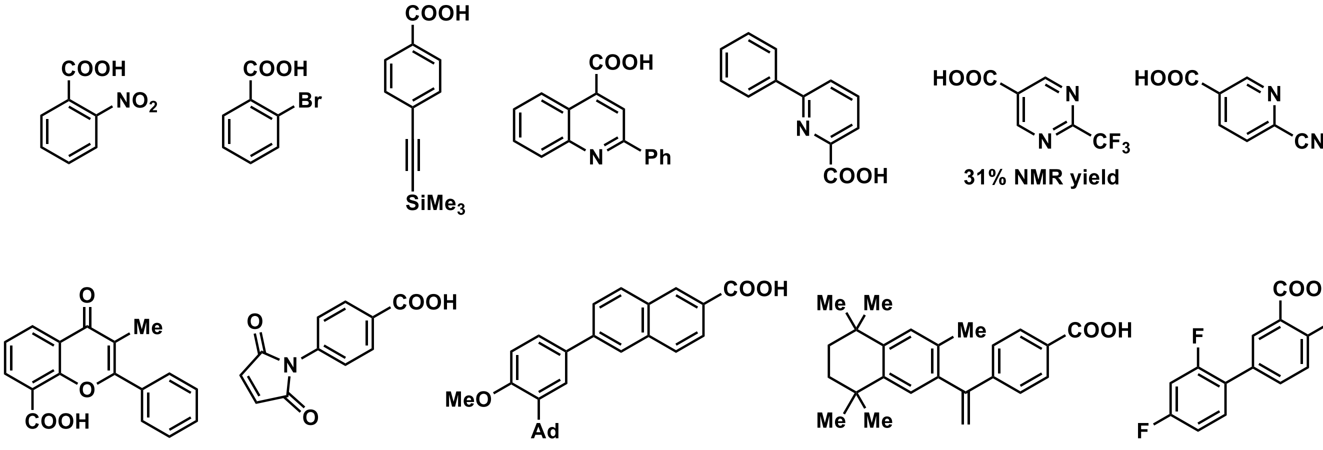

Adapalene Bexarotene Diflusinal

Preparation of starting material

Triclosan-derived benzoic acid S31

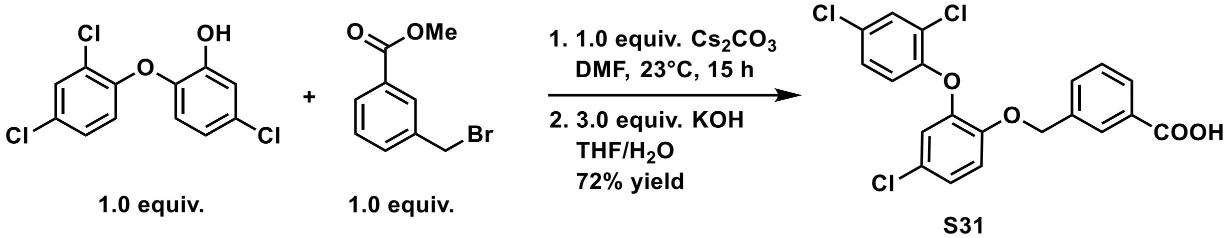

S31

A 50 mL round-bottom flask was charged with a stirring bar, triclosan (868 mg, 3.00 mmol, 1.00 equiv), methyl 3-(bromomethyl)benzoate (687 mg, 3.00 mmol, 1.00 equiv) and Cs_2CO_3 (977 mg, 3.00 mmol, 1.00 equiv). 10 mL DMF was added, and the reaction mixture was stirred at 23 °C for 12 h. The mixture was diluted with ethyl acetate (25 ml), washed with water (25 ml x 3), and dried over Na_2SO_4. The drying agent was removed by filtration and the filtrate was evaporated to dryness under reduced pressure. To the flask which contained the residue, a stirring bar, 6 mL THF and 4.5 mL 2 M KOH (aq) were added. The reaction mixture was stirred at 23 °C for 15 h. THF was removed under reduced pressure and the mixture was acidified with 1M HCl (aq) to pH = 2. The remaining aqueous suspension was extracted with EtOAc (50 mL x 3). The combined organic layers were dried over Na_2SO_4, filtered, and the filtrate was concentrated under reduced pressure to dryness. The residue was purified by chromatography on silica gel (pentane/EtOAc = 5/1, v/v) to afford triclosan-derived benzoic acid (**S31**) (918 mg, 2.16 mmol, 72%) as a white solid.

R*f* =0.3 (pentane/EA = 5/1, v/v).

NMR Spectroscopy:

^{1}H NMR (500 MHz, $(CD_3)_2SO$, 298 K, δ): 12.99 (brs, 1H), 7.89–7.82 (m, 2H), 7.61 (d, *J* = 2.6 Hz, 1H), 7.46–7.34 (m, 3H), 7.27 (dd, *J* = 8.8, 2.5 Hz, 1H), 7.14 (d, *J* = 8.6 Hz, 1H), 7.06 (dd, *J* = 8.5, 2.4 Hz, 1H), 6.78 (d, *J* = 8.8 Hz, 1H), 5.21 (s, 2H).ppm.

^{13}C NMR (126 MHz, $(CD_3)_2SO$, 298 K, δ): 167.0, 152.0, 150.1, 142.5, 136.7, 131.4, 131.0, 129.8, 129.7, 128.8, 128.6, 128.3, 128.0, 127.0, 123.3, 122.5, 121.4, 118.2, 115.4, 69.6. ppm.

HRMS-ESI (m/z) calculated for $C_{20}H_{12}O_4Cl_3^-$ $[M\text{-}H]^-$, 420.9807; found, 420.9810; deviation: –0.8 ppm.

Mechanistic Studies

UV-vis Absorption Spectrum

All UV-vis measurements were recorded on a Shimadzu UV-vis Spectrophotometer UV-2600 with temperature controller using a screw-top quartz cuvette (Hellma fluorescence quartz cuvette, 10 × 10 mm, 3.5 mL). Samples were prepared in a glovebox and then taken out of the glovebox.

Preparation of the stock solution/suspension of reaction components in a nitrogen-filled glovebox:

A. lithium 4-fluorobenzoate (**2a**) (8.8 mg, 60 μmol) in 20 mL MeCN;
B. $Cu(OTf)_2$ (54.2 mg,150 μmol) in 20 mL MeCN (7.5 mM);
C. CuTC (17.2 mg, 90.0 μmol) in 20 mL MeCN.

Note: stock A and C are suspensions.

UV-vis spectra of **2a** (1.0 mL of stock A + 2.0 mL MeCN; filtered through 0.22 μm syringe filter), $Cu(OTf)_2$ (1.0 mL of stock B + 2.0 mL MeCN, 2.5 mM), CuTC (1.0 mL of stock C + 2.0 mL MeCN, filtered through 0.22 μm syringe filter), the mixture of **2a** + $Cu(OTf)_2$ (1 mL of stock A + 1mL of stock B +1 mL MeCN, homogenous), the mixture of **2a** + CuTC (1.0 mL of stock A + 1.0 mL of stock C + 1.0 mL MeCN, filtered through 0.22 μm syringe filter), the mixture of $Cu(OTf)_2$ + CuTC (1.0 mL of stock B + 1.0 mL of stock C + 1.0 mL MeCN, filtered through 0.22 μm syringe filter), and the mixture of **2a** + $Cu(OTf)_2$ + CuTC (1.0 mL of stock A + 1.0 mL of stock B + 1.0 mL of stock C, filtered through 0.22 μm syringe filter) were recorded respectively. The mixture of **2a** + $Cu(OTf)_2$, **2a** + CuTC and the mixture of **2a** + $Cu(OTf)_2$ + CuTC have significant absorbance from 370 to 470 nm which indicates the ligand to metal charge transfer (LMCT) band. All samples containing $Cu(OTf)_2$ show a strong absorbance from 550 to 900 nm.

Note: lithium 4-fluorobenzoate and CuTC are poorly dissolved in MeCN in all samples that contain either of these two components (except for the mixture of lithium 4-fluorobenzoate + $Cu(OTf)_2$). The actual concentration of lithium 4-fluorobenzoate in these samples is less than 1.0 mM and the actual concentration of CuTC is less than 1.5 mM.

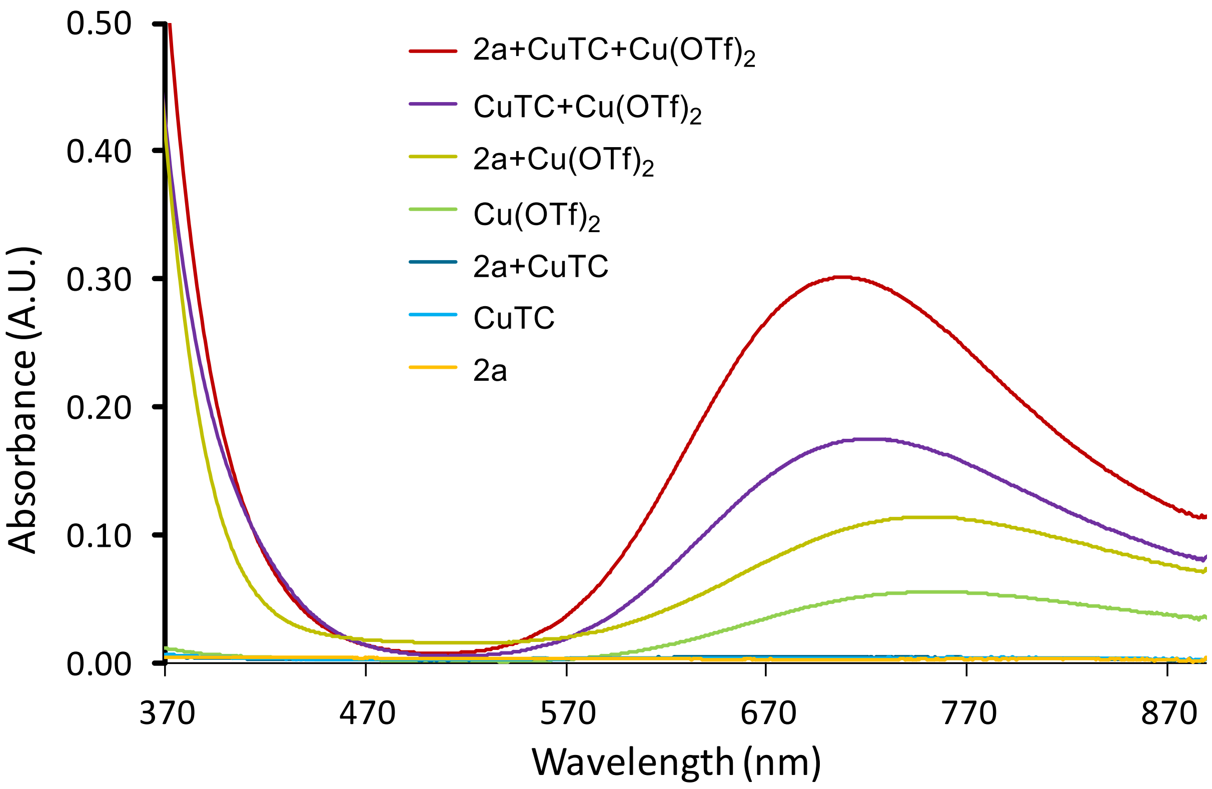

Figure S22. UV-vis spectra of the reaction components

Photolysis of the mixture of 2a, $Cu(OTf)_2$, and CuTC

Preparation of the stock solution/suspension of reaction components in a nitrogen-filled glove box:

A. lithium 4-fluorobenzoate (**2a**) (8.8 mg, 60 μmol) in 20 mL MeCN;
B. $Cu(OTf)_2$ (54.2 mg,150 μmol) in 20 mL MeCN (7.5 mM);
C. CuTC (17.2 mg, 90.0 μmol) in 20 mL MeCN.

Note: stock A and C are suspensions.

In a nitrogen-filled glovebox, a mixture of **2a,** $Cu(OTf)_2$, CuTC (1.0 mL of stock A + 1.0 mL of stock B + 1.0 mL of stock C, filtered through 0.22 μm syringe filter) was transferred to a screw-top quartz cuvette (Hellma fluorescence quartz cuvette, 10 × 10 mm, 3.5 mL). The quartz cuvette was sealed and taken out of the glovebox. The absorption spectra were recorded on a Shimadzu UV-vis Spectrophotometer UV-2600 after the cuvette was irradiated by two Kessil PR160-390 nm LEDs (5 cm away from two Kessil PR160-390 nm LEDs, and the temperature was maintained at approximately 35 °C through cooling with a fan) for various time (0 min, 1 min, 3 mins, 6 mins, 16 mins, and 46 mins).

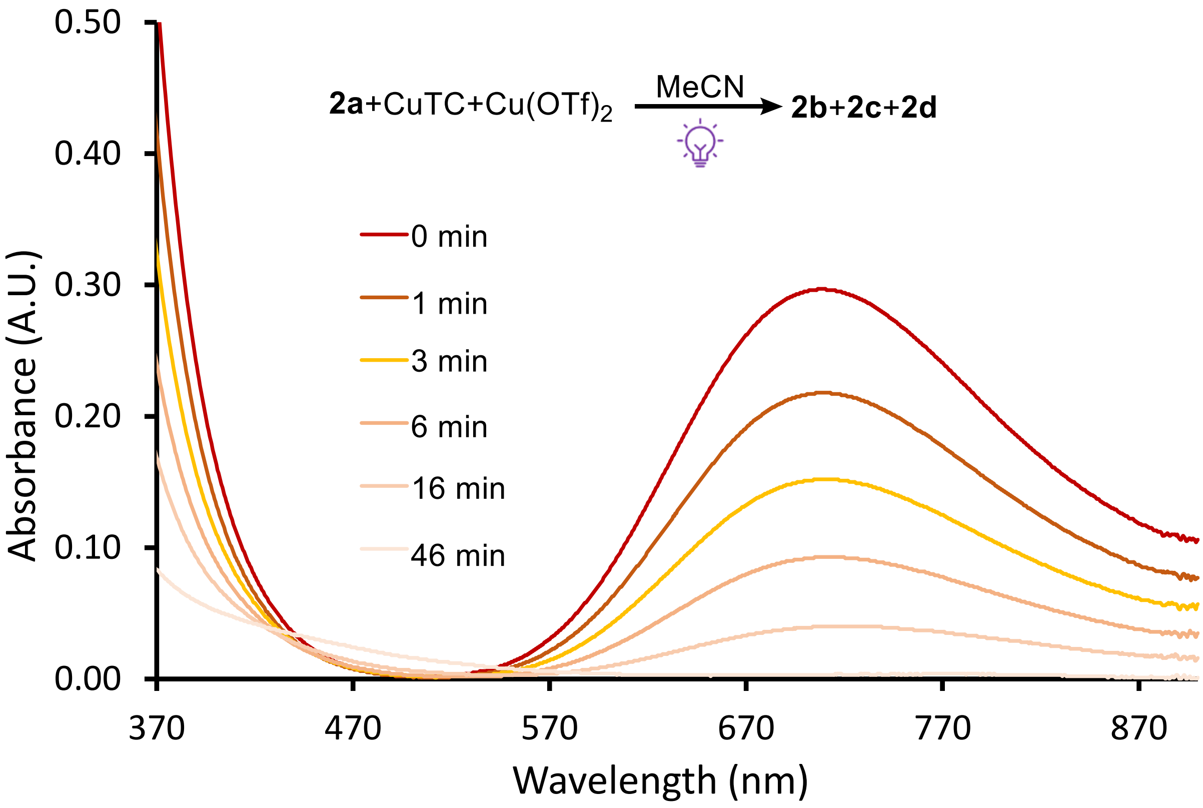

Figure S23. UV-vis spectra of the photolyzed mixture of **2a**, $Cu(OTf)_2$, and CuTC in CH_3CN

Radical cyclisation experiment

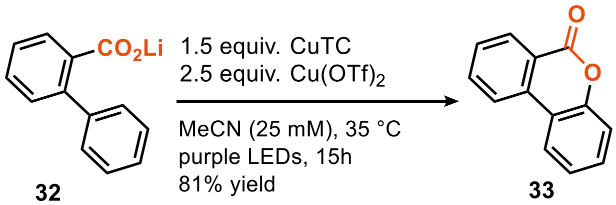

Under an ambient atmosphere, a 10 mL borosilicate vial equipped with a magnetic stir bar was charged with lithium [1,1'-biphenyl]-2-carboxylate (**32**) (40.8 mg, 0.200 mmol, 1.00 equiv.) and copper (I) thiophene-2-carboxylate (57.2 mg, 0.300 mmol, 1.50 equiv.). The vial was transferred into an anhydrous, N_2-filled glovebox. Under nitrogen atmosphere, $Cu(OTf)_2$ (181 mg, 0.500 mmol, 2.50 equiv.), and anhydrous MeCN (8.0 mL, 25 mM) and were then added into the vial, resulting in a green suspension. The vial was sealed with a Teflon cap and placed 5 cm away from two purple LEDs (Kessil PR160-390 nm LEDs). The reaction mixture was stirred at a speed of 1200 rpm (Note: Fast stirring is essential due to the heterogeneity of the reaction mixture.) and irradiated for 16 h while maintaining the temperature at approximately 35 °C through cooling with a fan. Subsequently, the reaction mixture was transferred into a 50 mL round-bottom flask and evaporated to dryness. The residue was purified by chromatography on silica gel (pentane/EtOAc = 9/1, v/v) to afford 6*H*-benzo[*c*]chromen-6-one (**33**) (32.0 mg, 163 μmol, 81%) as a colorless solid.

R*f* =0.30 (pentane/EtOAc = 9/1, v/v).

NMR Spectroscopy:

¹H NMR (500 MHz, CD_3CN, 298 K, δ): 8.24 (ddd, *J* = 7.9, 1.5, 0.6 Hz, 1H), 8.16 (ddd, *J* = 8.1, 1.1, 0.6 Hz, 1H), 8.10 (dd, *J* = 7.9, 1.5 Hz, 1H), 7.88–7.76 (m, 1H), 7.58 (ddd, *J* = 8.2, 7.2, 1.1 Hz, 1H), 7.49 (ddd, *J* = 8.2, 7.3, 1.5 Hz, 1H), 7.41–7.25 (m, 2H) ppm.

¹³C NMR (126 MHz, CD_3CN, 298 K, δ): 161.7, 152.2, 135.9, 135.6, 131.5, 130.8, 130.0, 125.5, 124.2, 123.1, 122.2, 119.0, 118.3 ppm.

HRMS-ESI (m/z) calculated for $C_{13}H_8O_2^+$ $[M]^+$, 196.0519; found, 196.0520; deviation: –0.6 ppm.

Radical trapping experiments

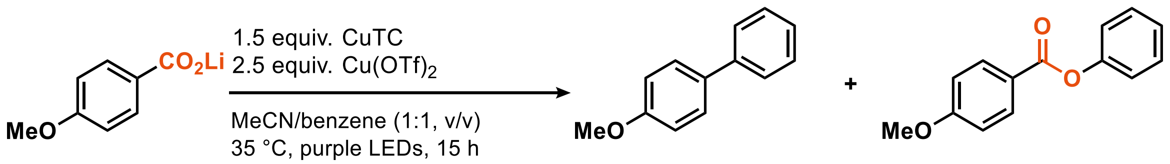

34 **35,** 6% **36,** 9%

Under an ambient atmosphere, a 10 mL borosilicate vial equipped with a magnetic stir bar was charged with lithium 4-methoxybenzoate (**34**) (31.6 mg, 0.200 mmol, 1.00 equiv.) and copper (I) thiophene-2-carboxylate (57.2 mg, 0.300 mmol, 1.50 equiv.). The vial was transferred into an anhydrous, N_2-filled glovebox. Under nitrogen atmosphere, $Cu(OTf)_2$ (181 mg, 0.500 mmol, 2.50 equiv.), and anhydrous MeCN (4.0 mL) and benzene (4.0 mL) were then added into the vial, resulting in a green suspension. The vial was sealed with a Teflon cap and placed 5 cm away from two purple LEDs (Kessil PR160-390 nm LEDs). The reaction mixture was stirred at a speed of 1200 rpm (Note: Fast stirring is essential due to the heterogeneity of the reaction mixture.) and irradiated for 16 h while maintaining the temperature at approximately 35 °C through cooling with a fan. Subsequently, the reaction mixture was transferred into a 50 mL round-bottom flask and evaporated to dryness. The residue was purified by chromatography on silica gel (pentane/DCM = 4/1 to 1/1, v/v) to afford 4-methoxy-1,1'-biphenyl (**35**) (2.1 mg, 11 μmol, 6%) as a colorless solid and phenyl 4-methoxybenzoate (**36**) (3.9 mg, 17 μmol, 9%) also as a pale yellow liquid.

4-methoxy-1,1'-biphenyl (**35**):

R*f* = 0.7 (Pentane/DCM = 1/1, v/v).

NMR Spectroscopy:

¹H NMR (500 MHz, CD_2Cl_2, 298 K, δ): 7.62–7.50 (m, 4H), 7.41 (t, *J* = 7.8 Hz, 2H), 7.34–7.25 (m, 1H), 6.98 (d, *J* = 8.8 Hz, 2H), 3.84 (s, 3H) ppm.

¹³C NMR (126 MHz, $CDCl_3$, 298 K, δ): 159.7, 141.1, 133.9, 129.1, 128.4, 127.0, 127.0, 114.6, 55.7 ppm.

HRMS-EI (m/z) calculated for $C_{13}H_{12}O^+$ $[M]^+$, 184.0883; found, 184.0881; deviation: 0.5 ppm.

phenyl 4-methoxybenzoate (**36**):

R*f* = 0.2 (Pentane/DCM= 1/1, v/v).

NMR Spectroscopy:

^{1}H NMR (500 MHz, CD_2Cl_2, 298 K, δ): 8.14 (d, *J* = 9.0 Hz, 1H), 7.50–7.41 (m, 1H), 7.31–7.25 (m, 1H), 7.23–7.17 (m, 1H), 7.01 (d, *J* = 9.0 Hz, 1H), 3.90 (s, 2H) ppm.

^{13}C NMR (126 MHz, CD_2Cl_2, 298 K, δ): 165.2, 164.4, 151.6, 132.5, 129.8, 128.3, 126.1, 122.3, 114.2, 56.0 ppm.

HRMS-CI (m/z) calculated for $C_{14}H_{13}O_3^+$ $[M+H]^+$, 229.0859; found, 229.0861; deviation: –1 ppm.

SPECTROSCOPIC DATA

^{1}H NMR of ataluren derivative 1

CD_3CN, 298 K

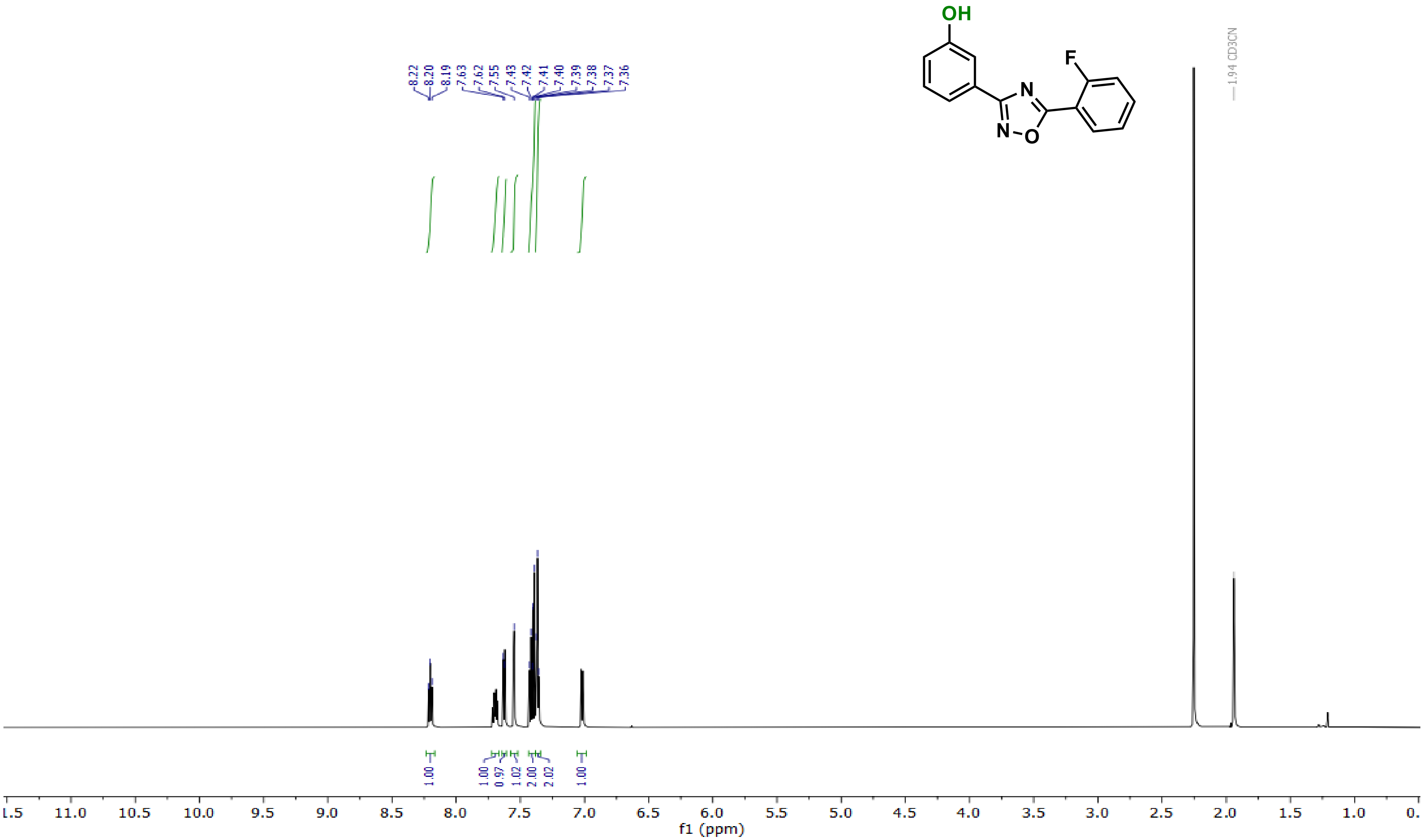

^{13}C NMR of ataluren derivative 1

CD$_3$CN, 298 K

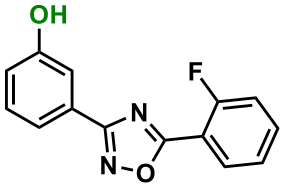

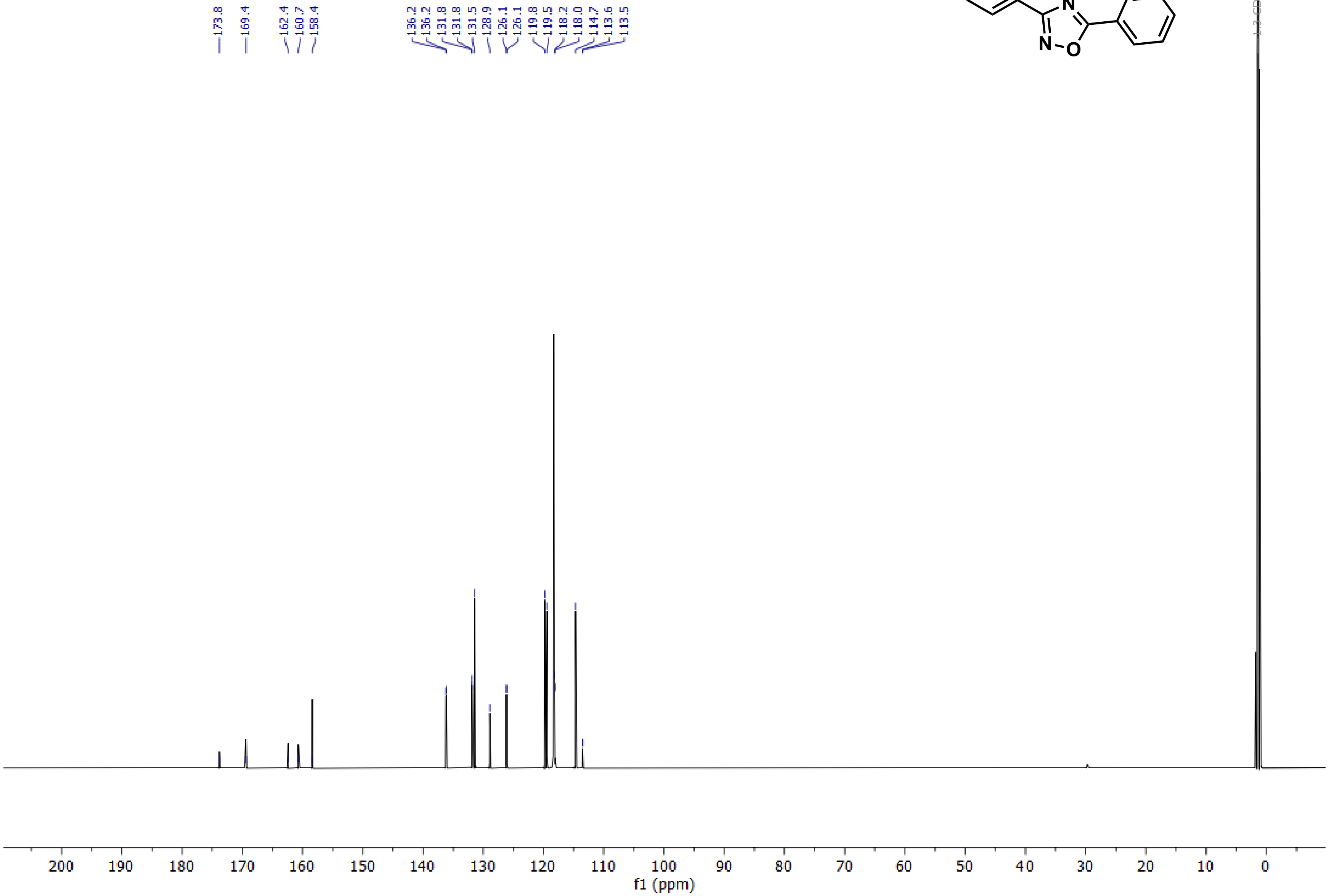

^{19}F NMR of ataluren derivative 1

CD_3CN, 298 K

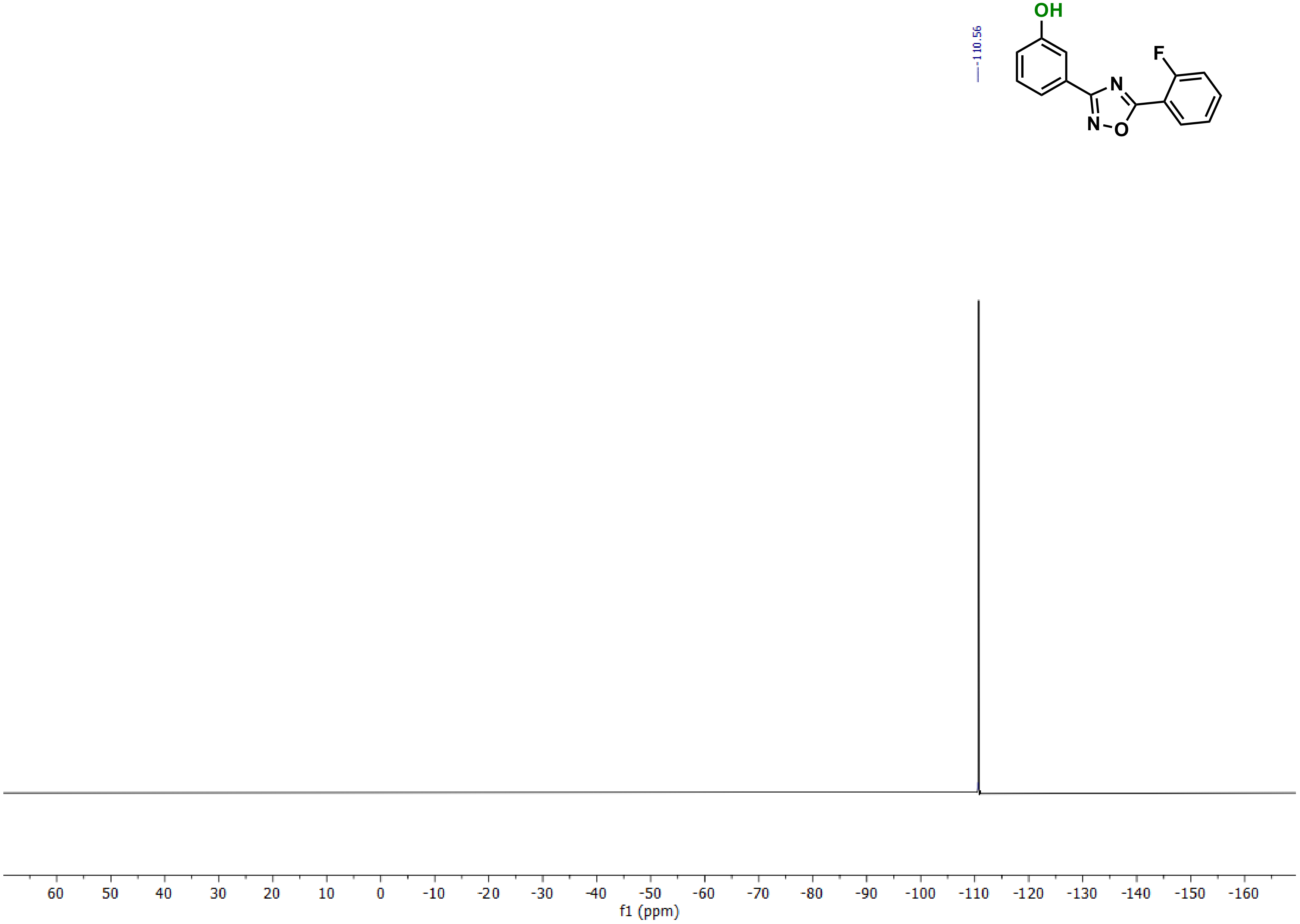

^{1}H NMR of 4-cyanophenol (3)

$CDCl_3$, 298 K

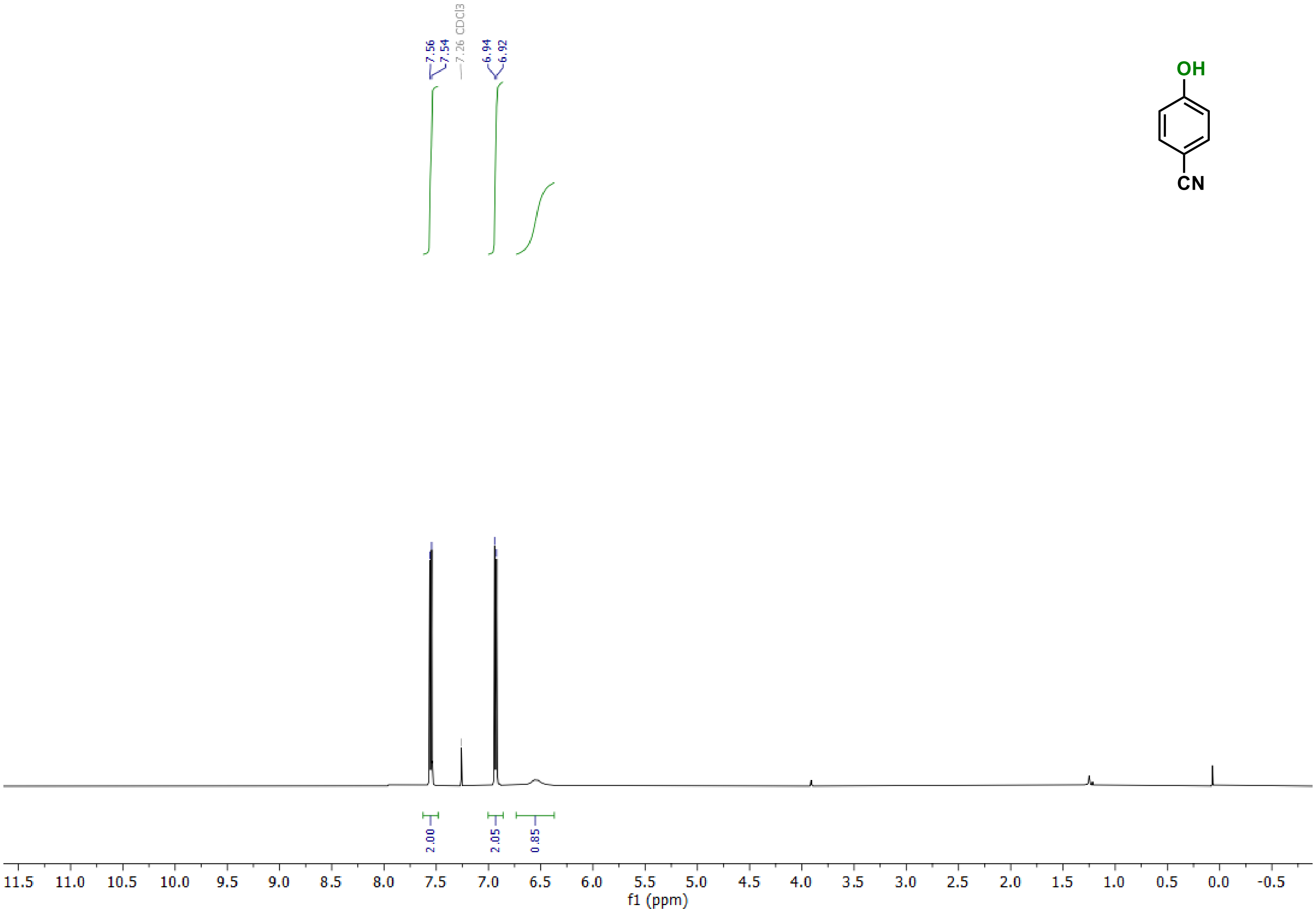

^{13}C NMR of 4-cyanophenol (3)

$CDCl_3$, 298 K

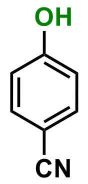

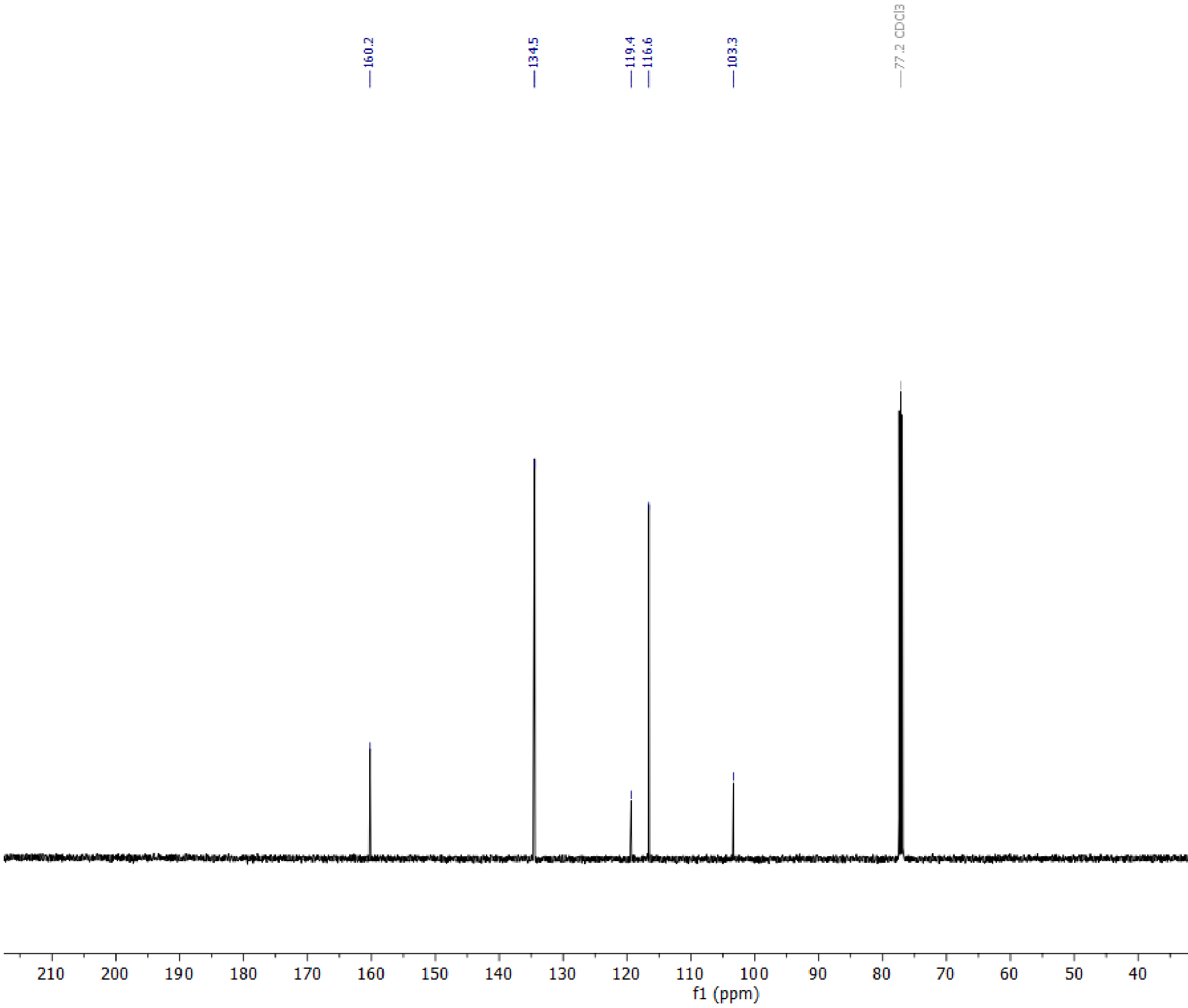

^{1}H NMR of 2-(trifluoromethyl)pyridin-4-ol (6)

$CDCl_3$, 298 K

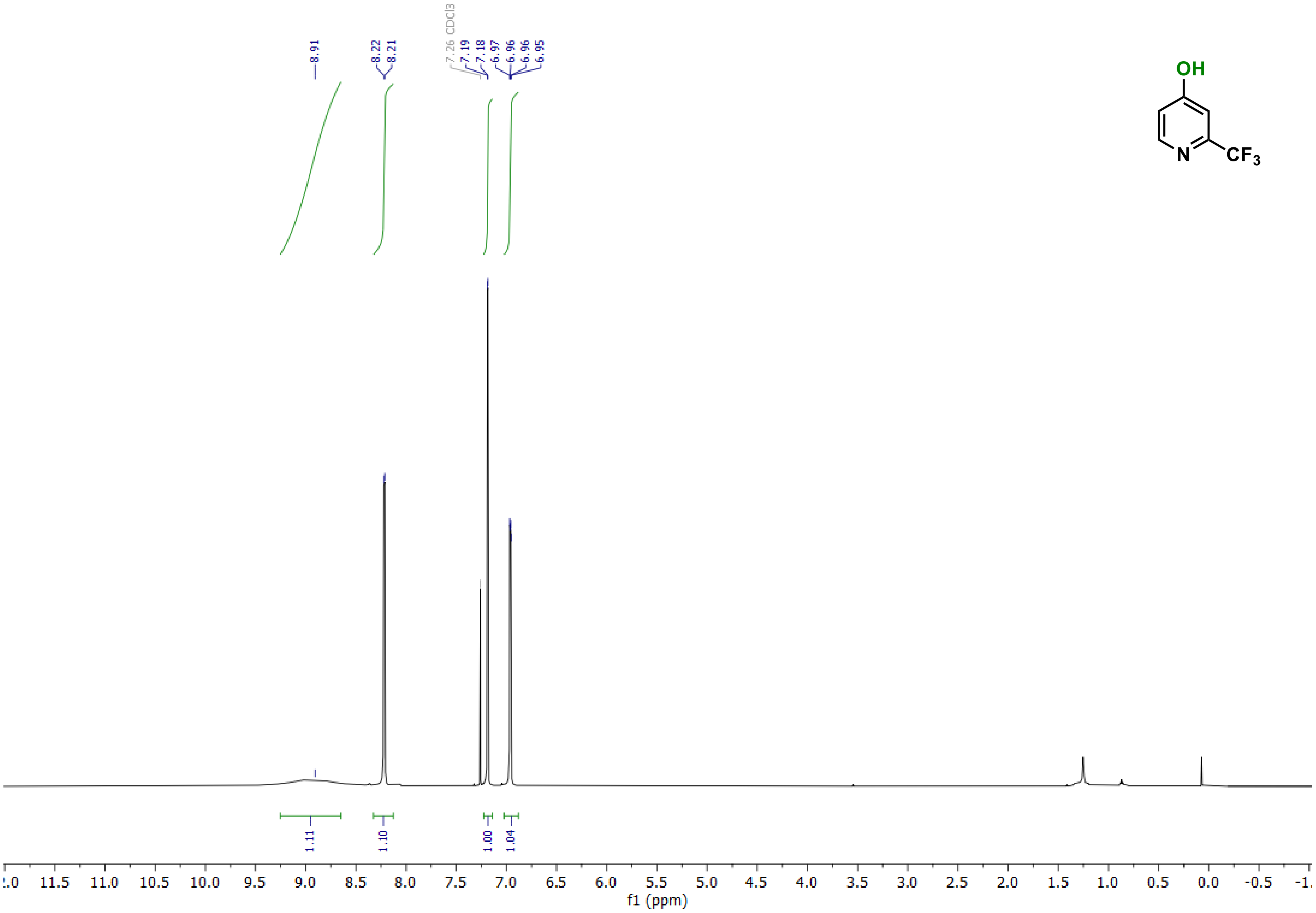

^{13}C NMR of 2-(trifluoromethyl)pyridin-4-ol (6)

$CDCl_3$, 298 K

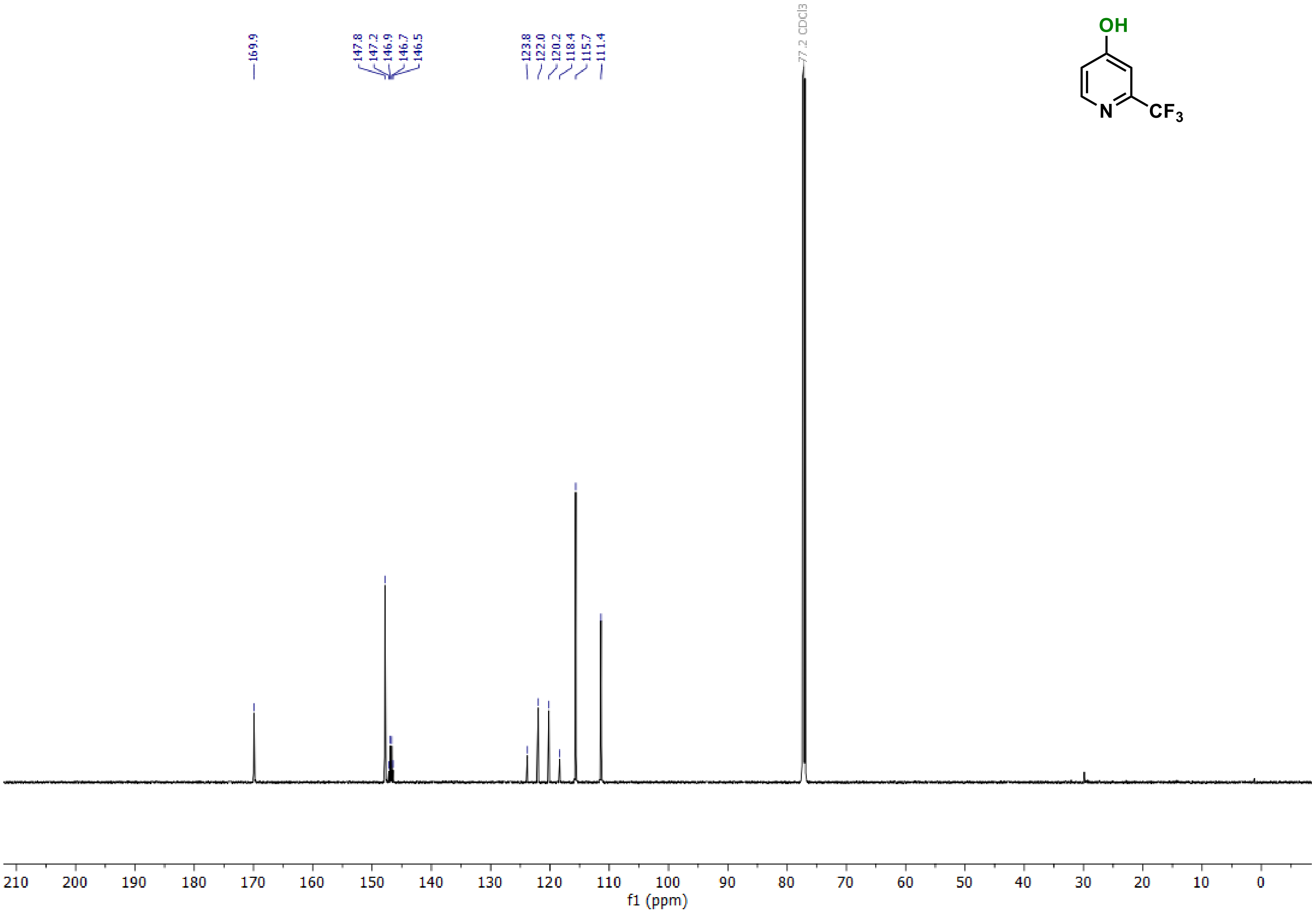

^{19}F NMR of 2-(trifluoromethyl)pyridin-4-ol (6)

$CDCl_3$, 298 K

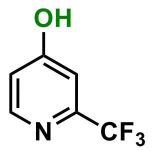

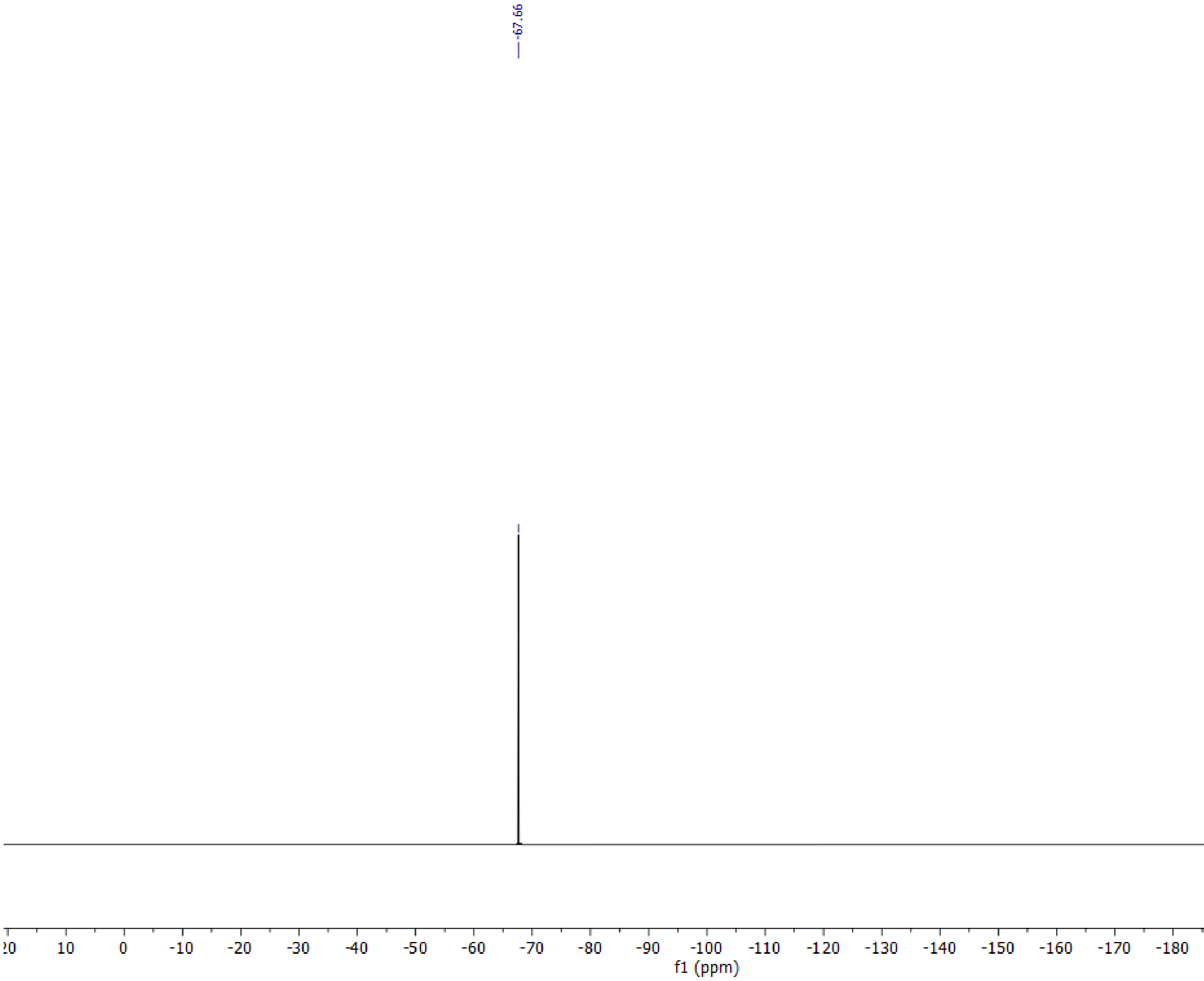

^{1}H NMR of 4-(5-methyl-1,2,4-oxadiazol-3-yl)phenol (7)

CD_3CN, 298 K

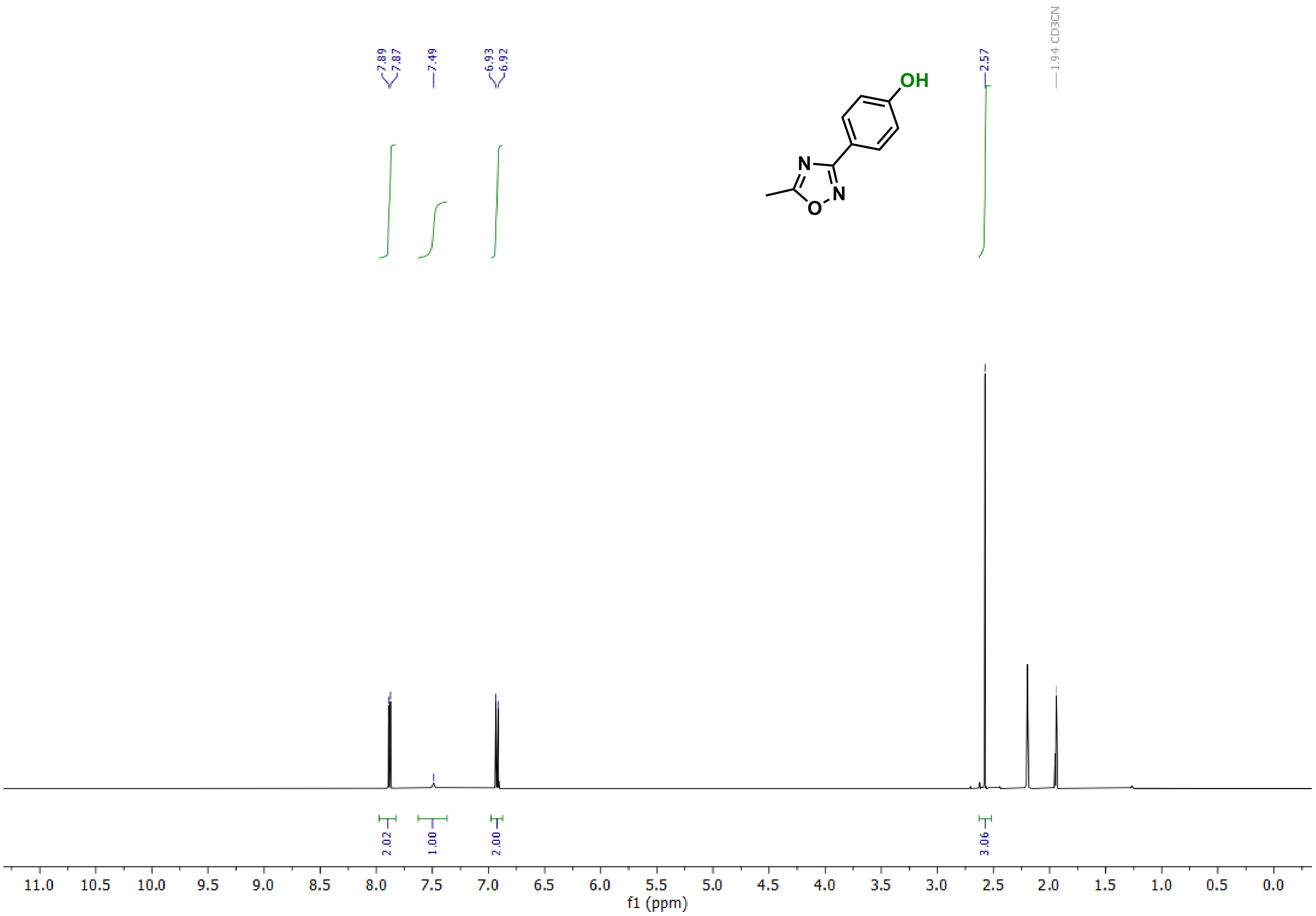

^{13}C NMR of 4-(5-methyl-1,2,4-oxadiazol-3-yl)phenol (7)

CD_3CN, 298 K

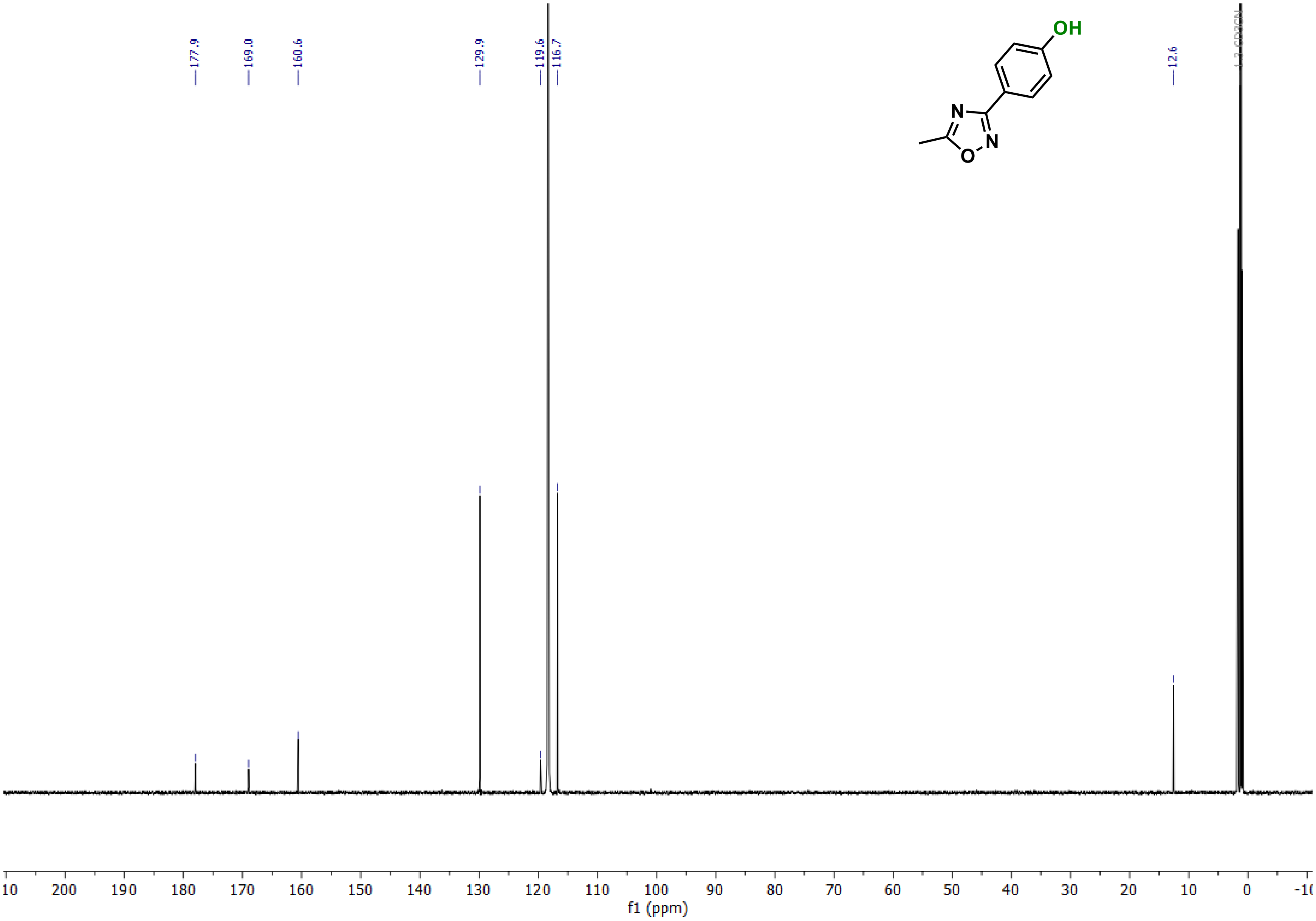

^{1}H NMR of 6-hydroxy-2-naphthonitrile (8)

CD_3CN, 25 ºC

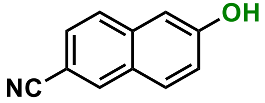

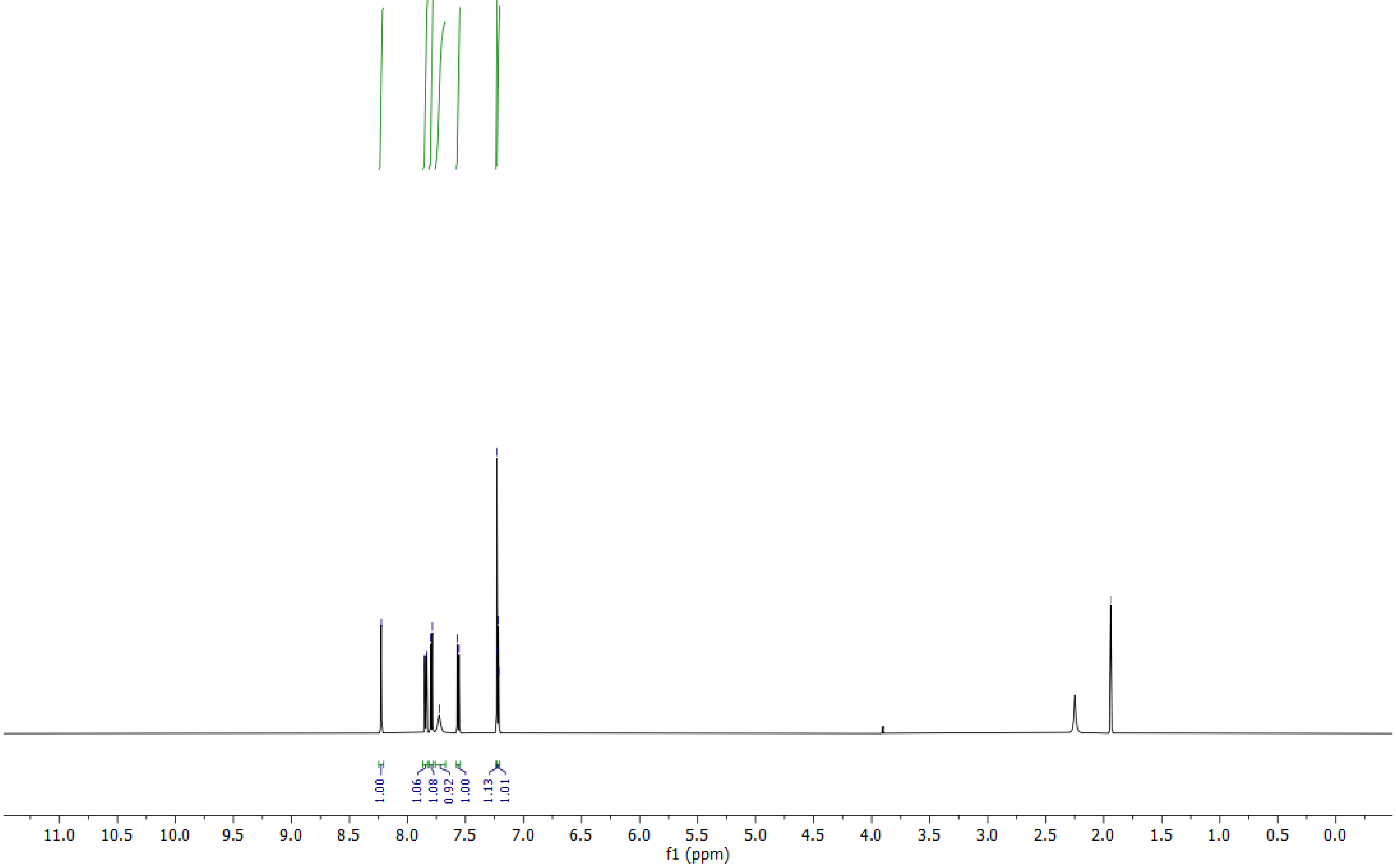

^{13}C NMR of 6-hydroxy-2-naphthonitrile (8)

CD_3CN, 25 ºC

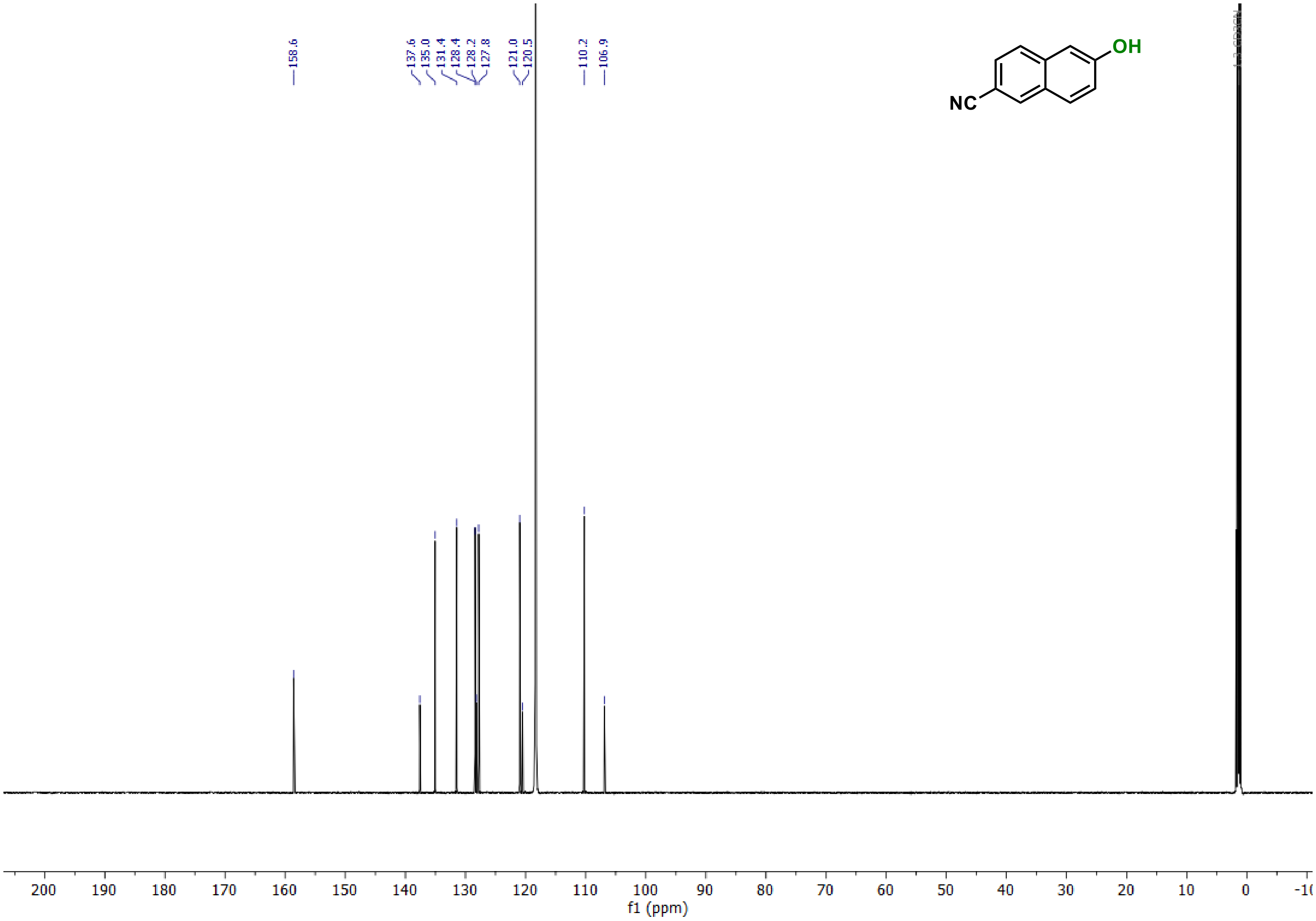

^{1}H NMR of (+)-menthol derivative 9

CD2Cl2, 25 ºC

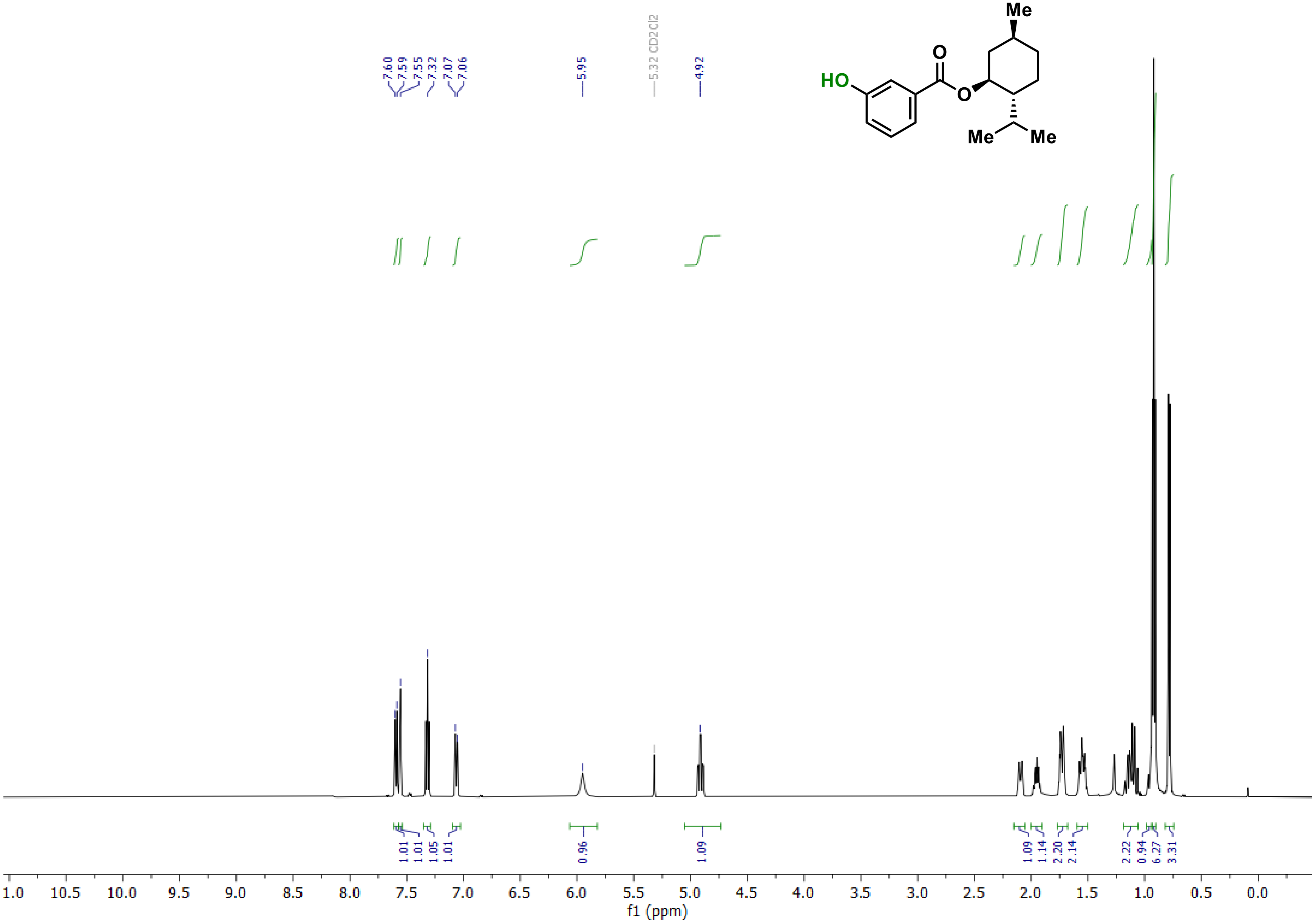

^{13}C NMR of (+)-menthol derivative 9

CD_2Cl_2, 25 ºC

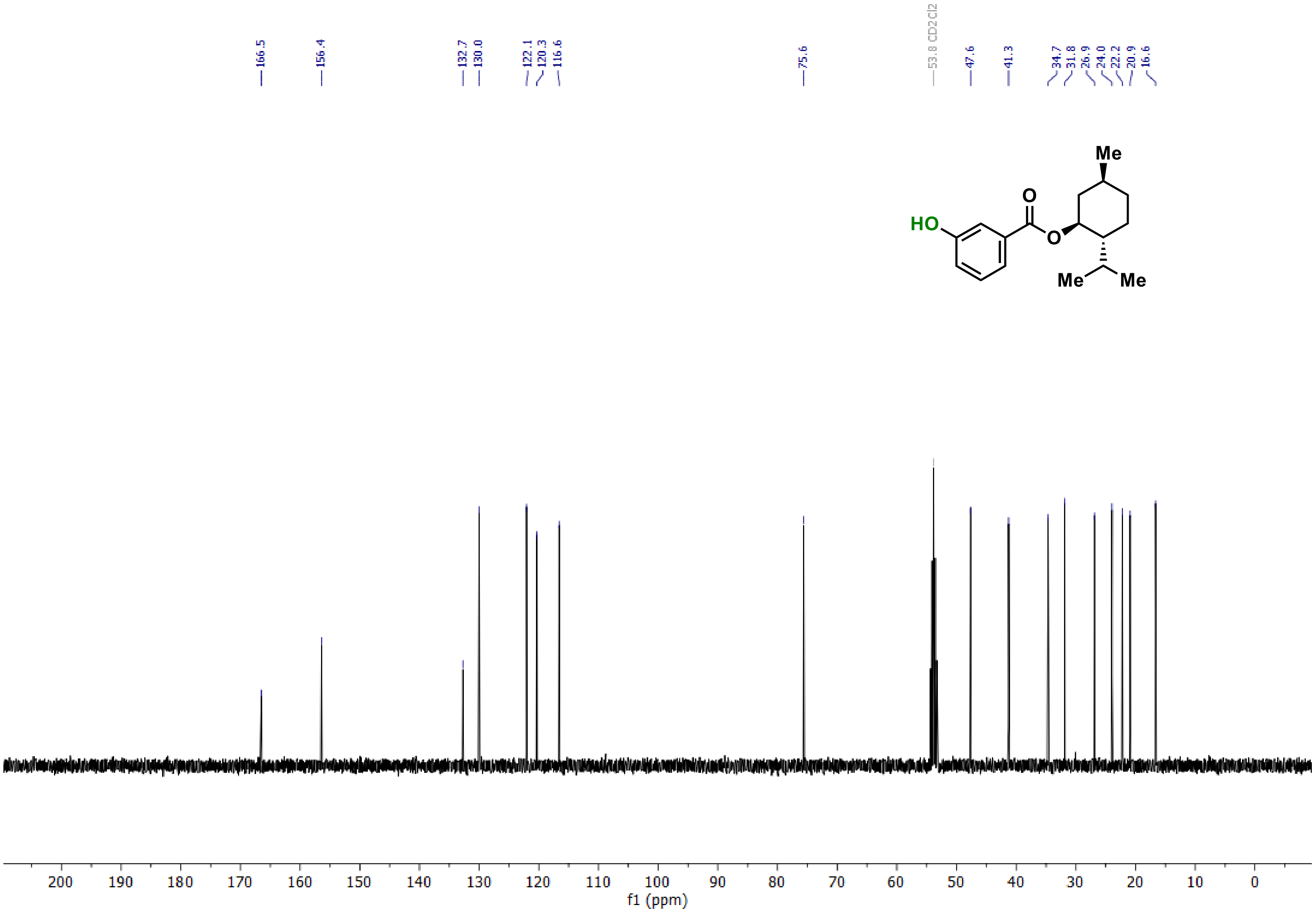

^{1}H NMR of 3-hydroxybenzaldehyde (10)

CD_3CN, 298 K

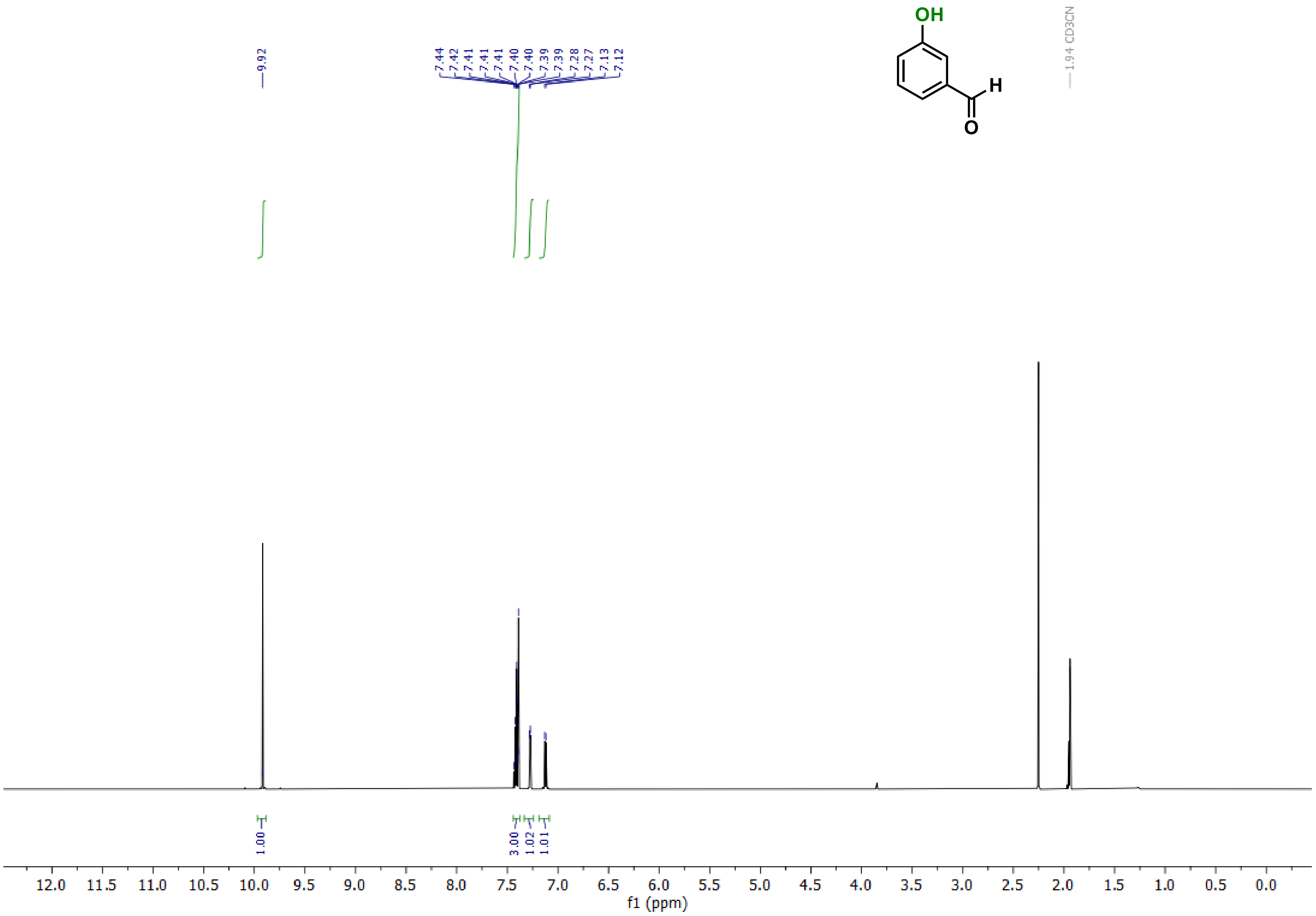

^{13}C NMR of 3-hydroxybenzaldehyde (10)

CD_3CN, 298 K

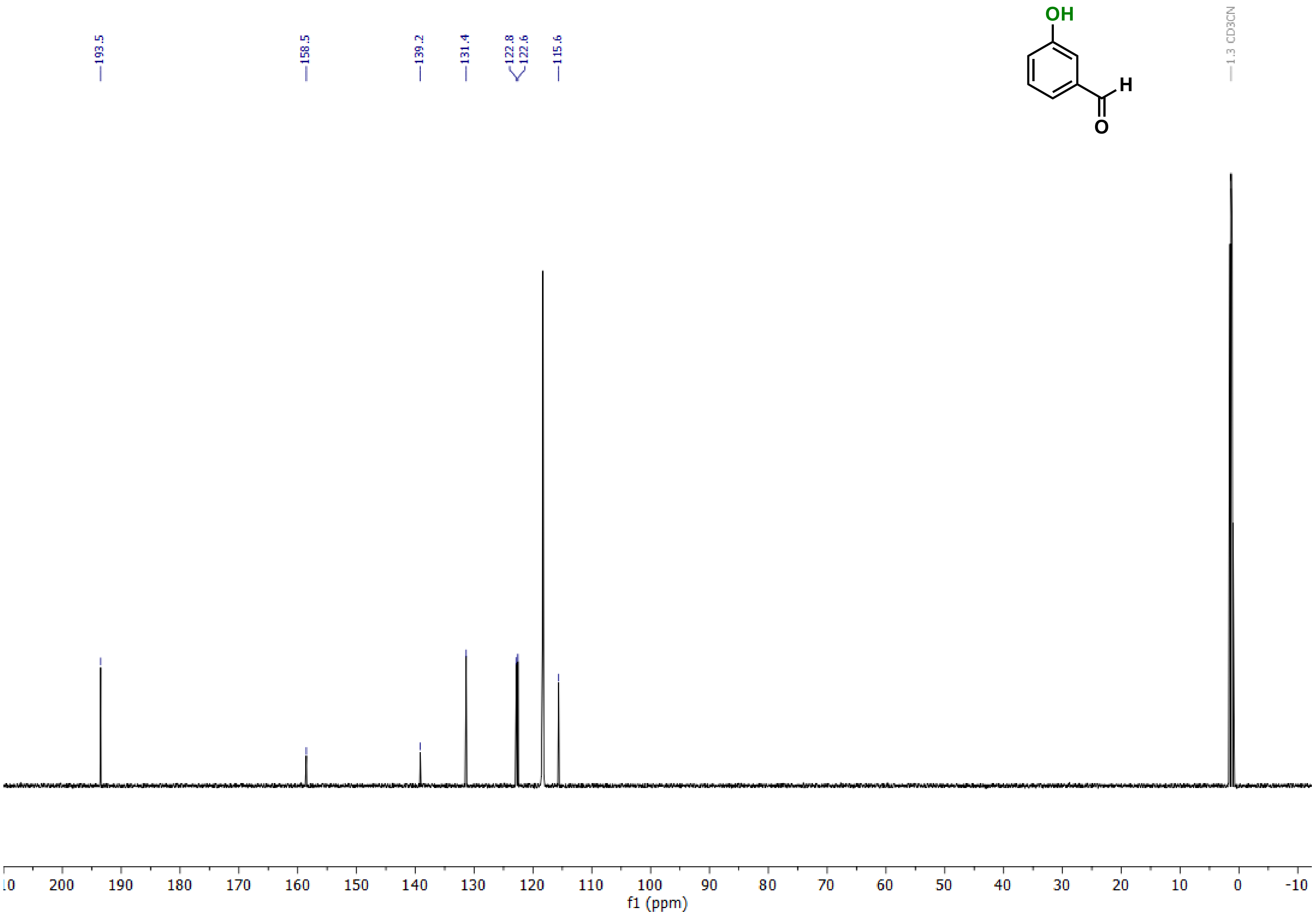

^{1}H NMR of 2,6-dichloropyridin-4-ol (14)

CD_3CN, 298 K

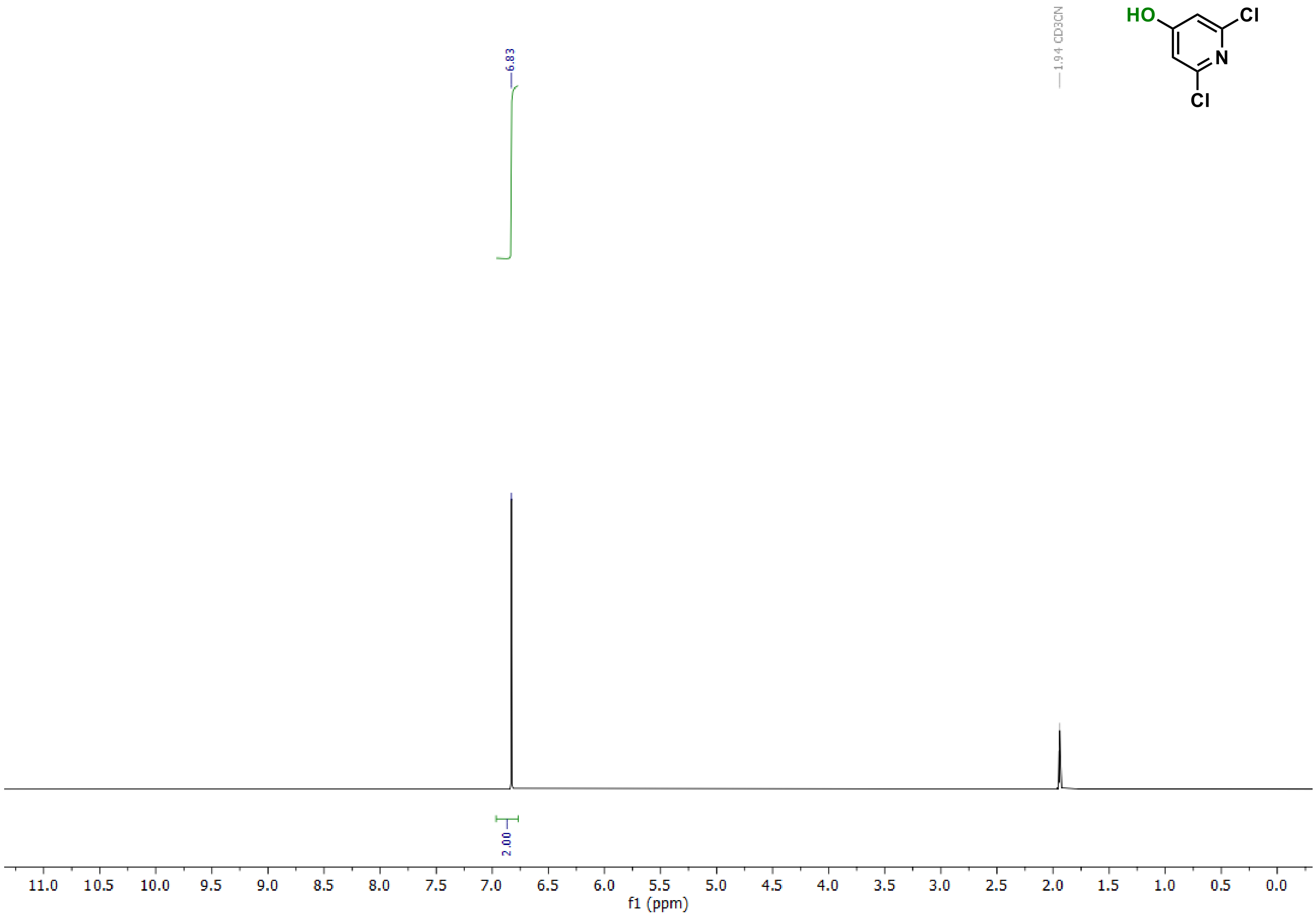

^{13}C NMR of 2,6-dichloropyridin-4-ol (14)

CD_3CN, 298 K

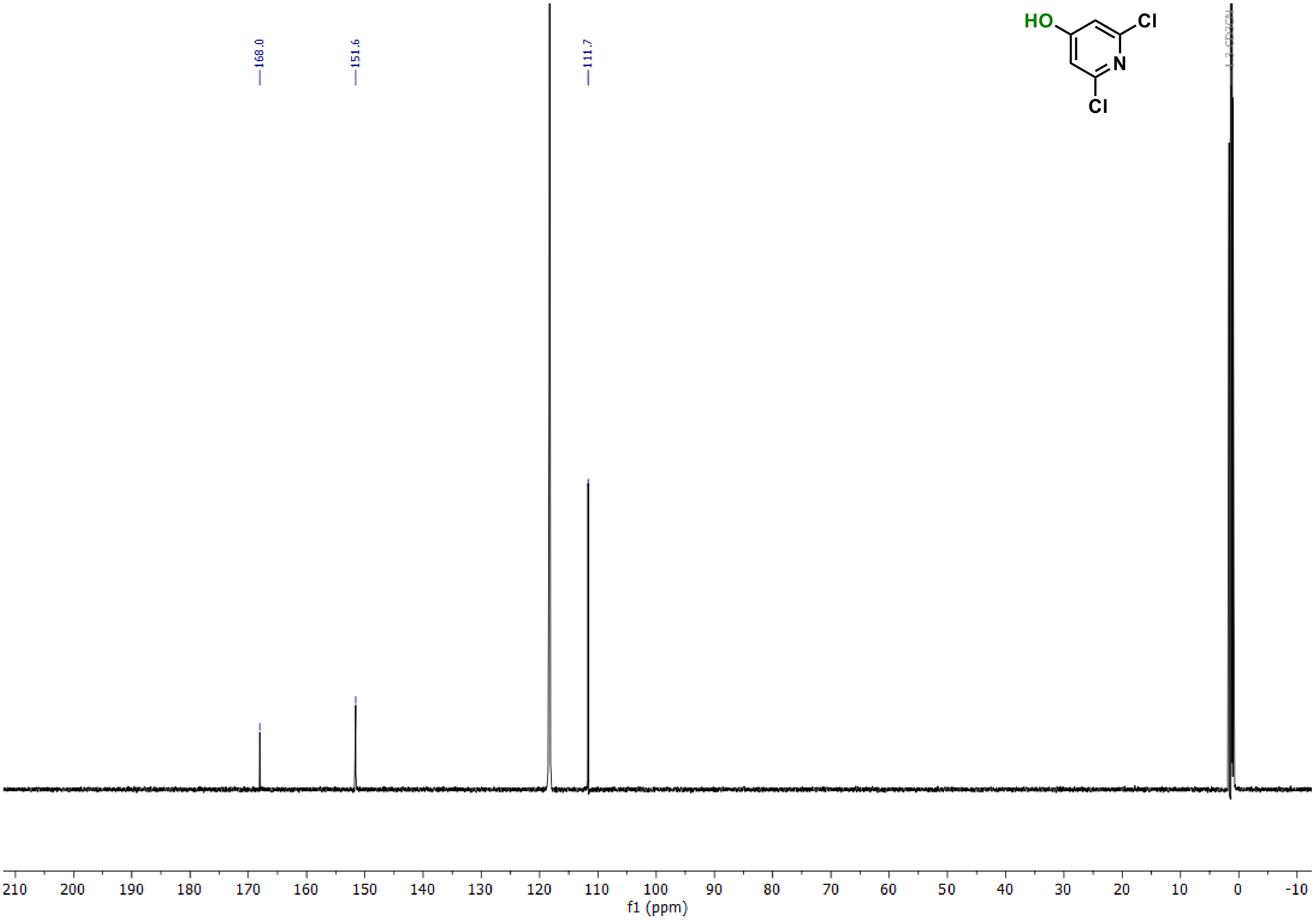

^{1}H NMR of 2-hydroxyquinoxaline (15)

$(CD_3)_2SO$, 298 K

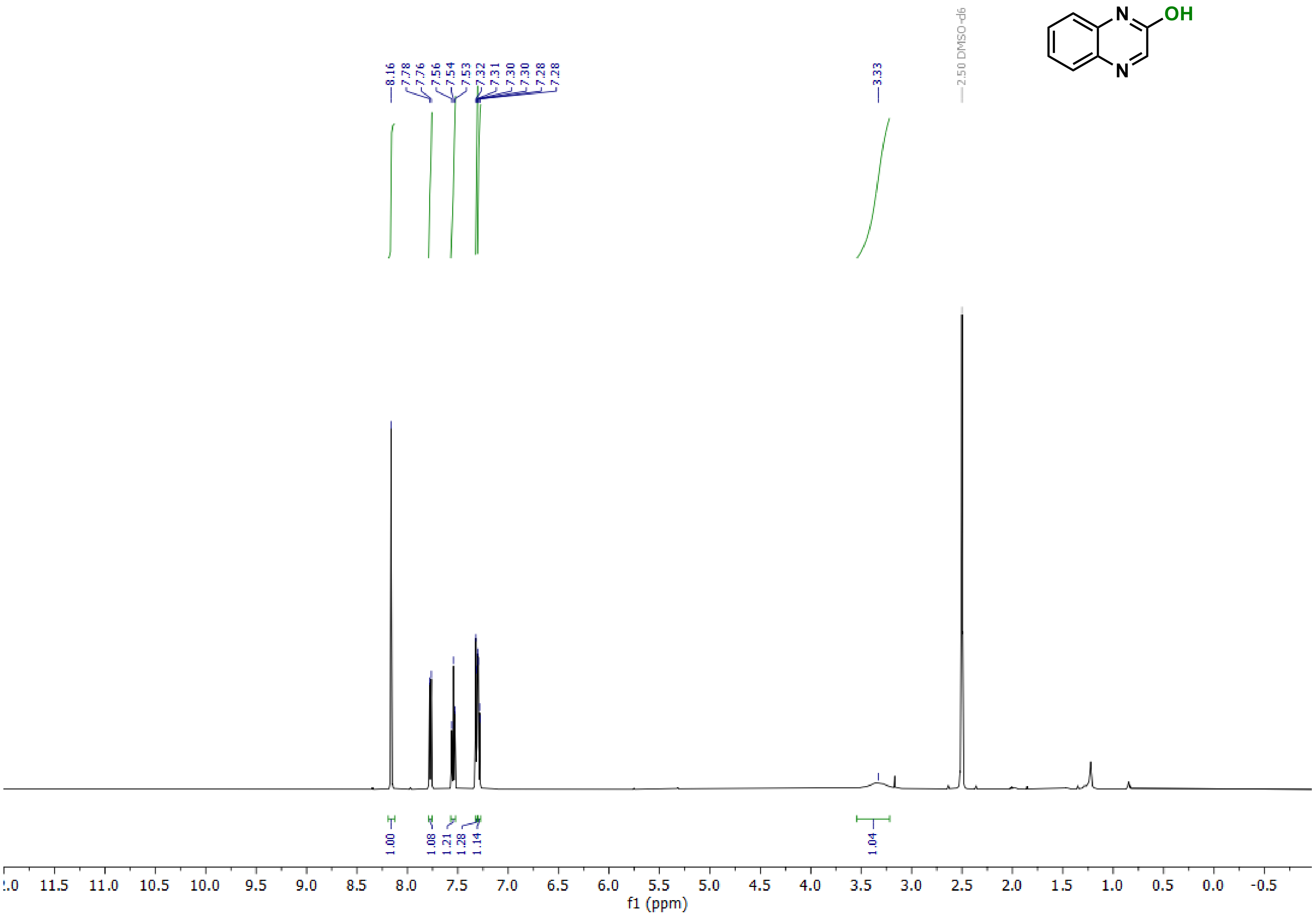

^{13}C NMR of 2-hydroxyquinoxaline (15)

$(CD_3)_2SO$, 298 K

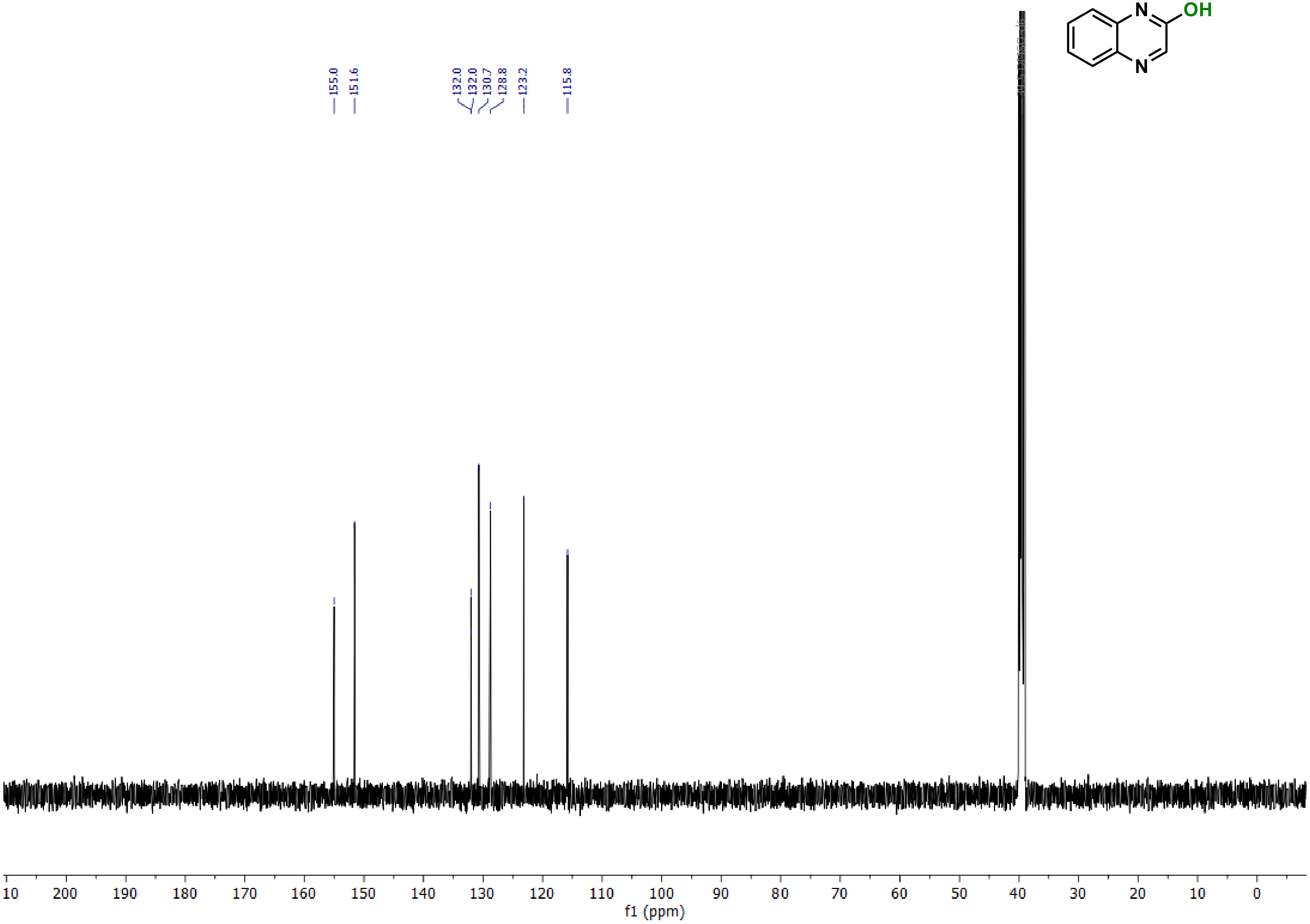

^{1}H NMR of 4-methyl-3-(morpholinosulfonyl)phenol (16)

$CDCl_3$, 298 K

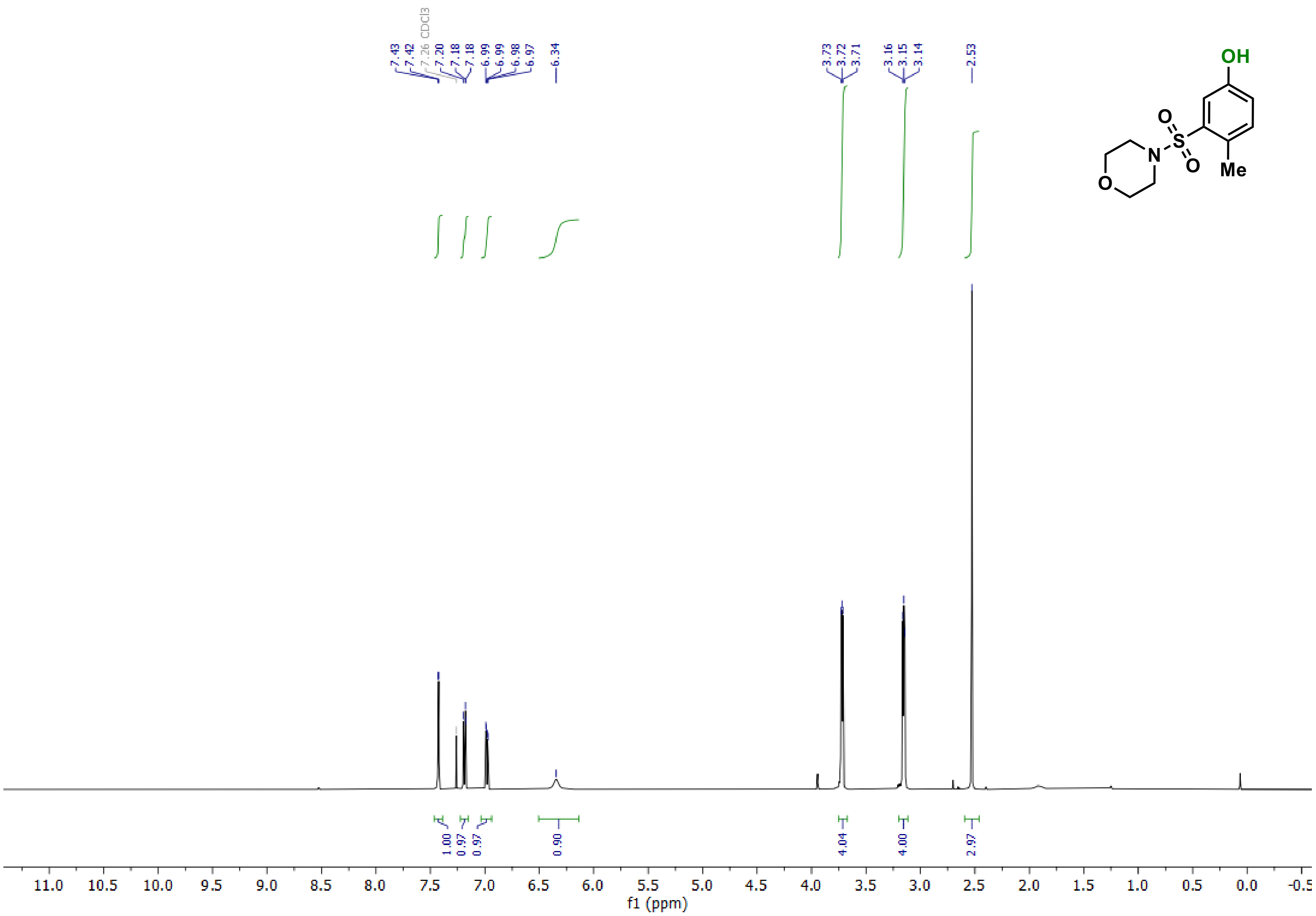

^{13}C NMR of 4-methyl-3-(morpholinosulfonyl)phenol (16)

$CDCl_3$, 298 K

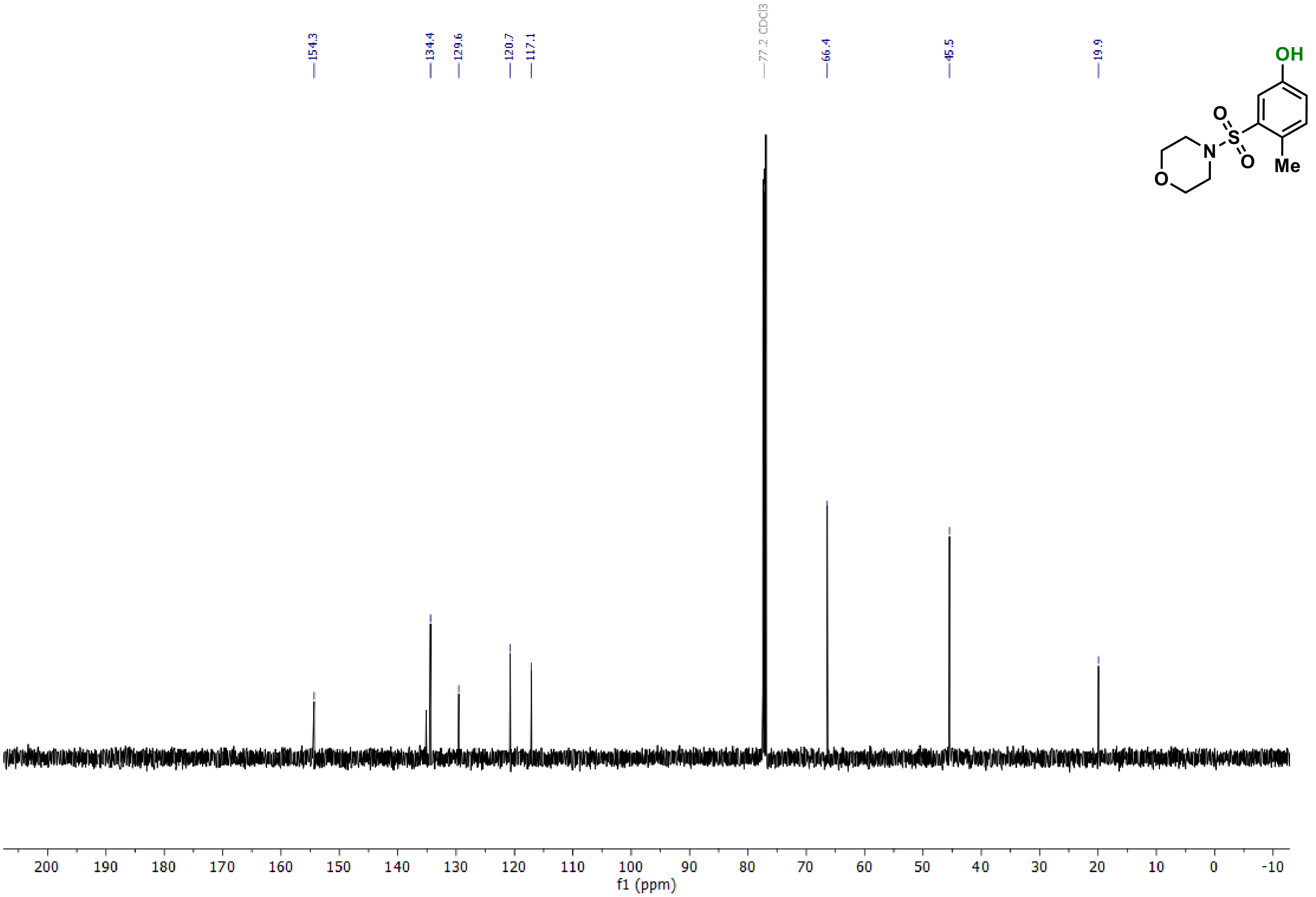

^{1}H NMR of probenicid derivative 17

$CDCl_3$, 298 K

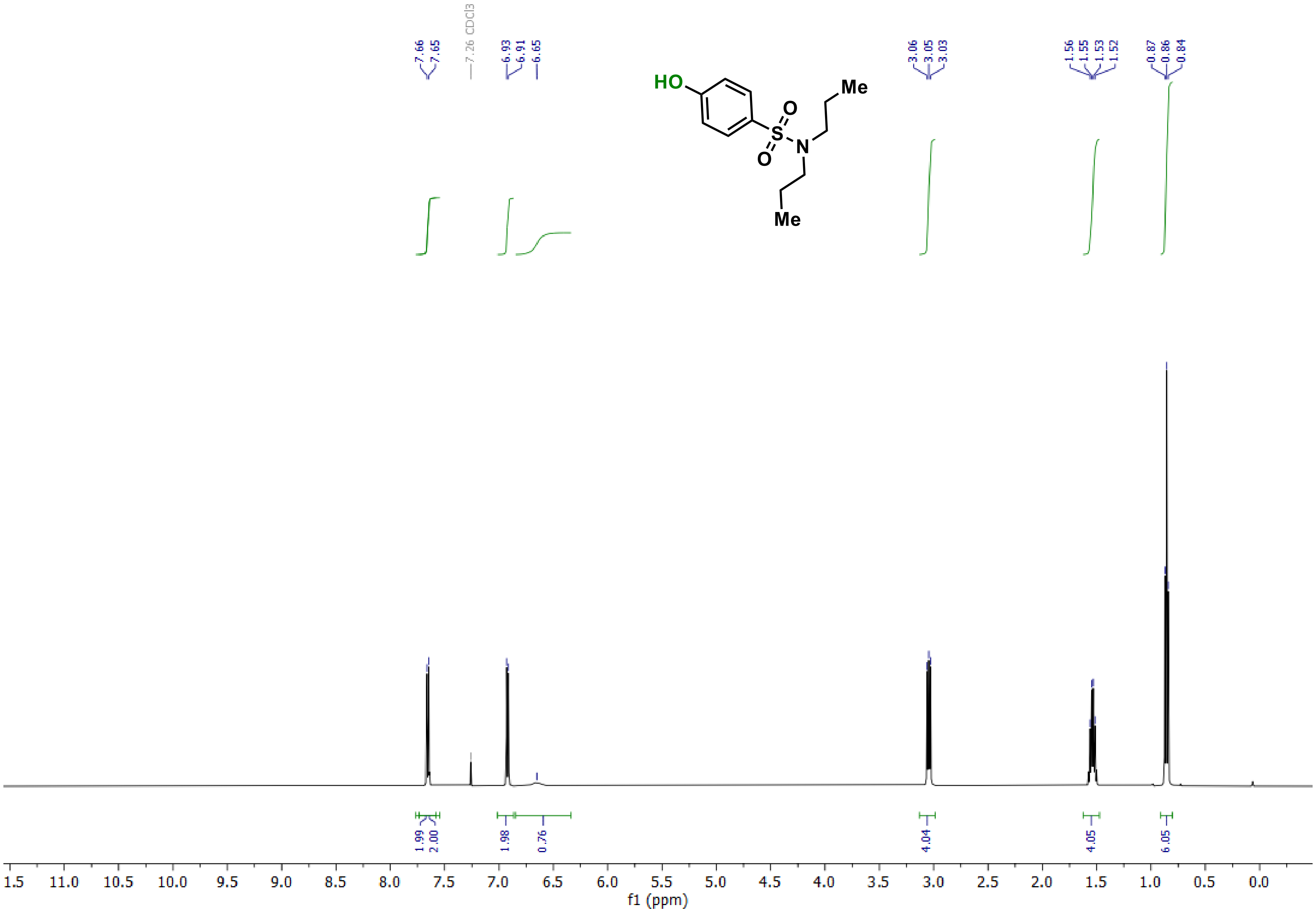

^{13}C NMR of probenicid derivative 17

$CDCl_3$, 298 K

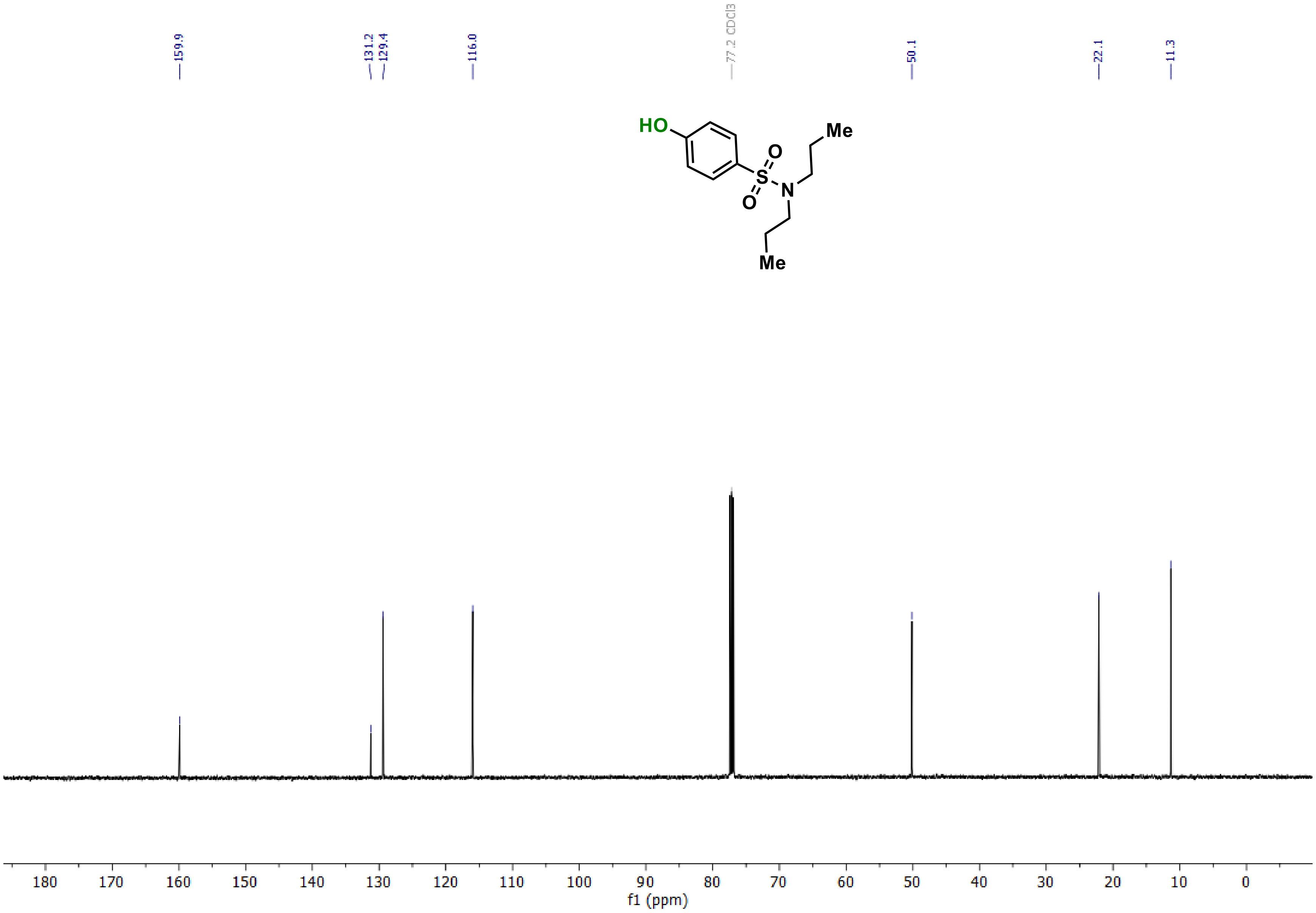

^{1}H NMR of 4-acetylphenol (18)

$CDCl_3$, 298 K

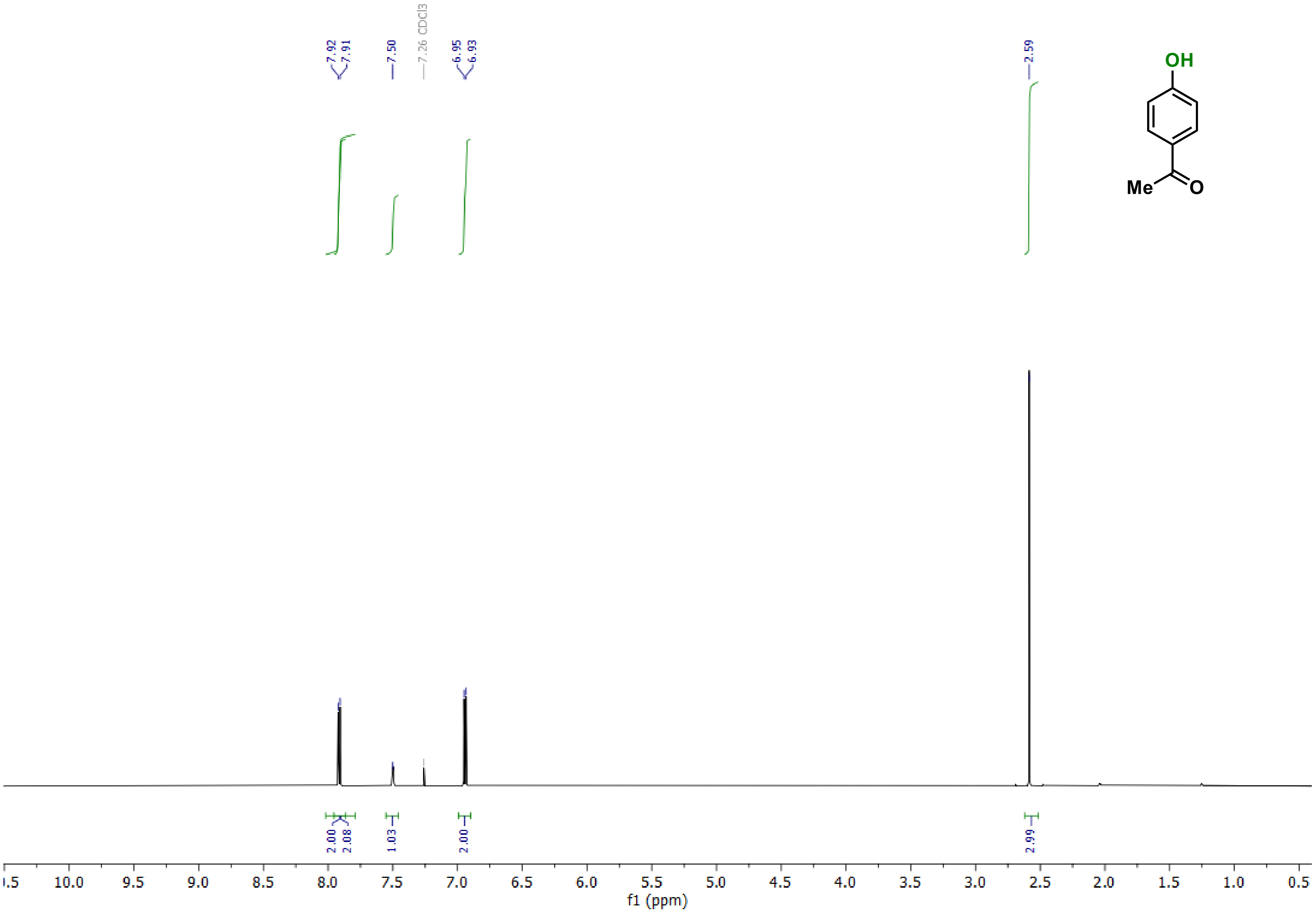

^{13}C NMR of 4-acetylphenol (18)

$CDCl_3$, 298 K

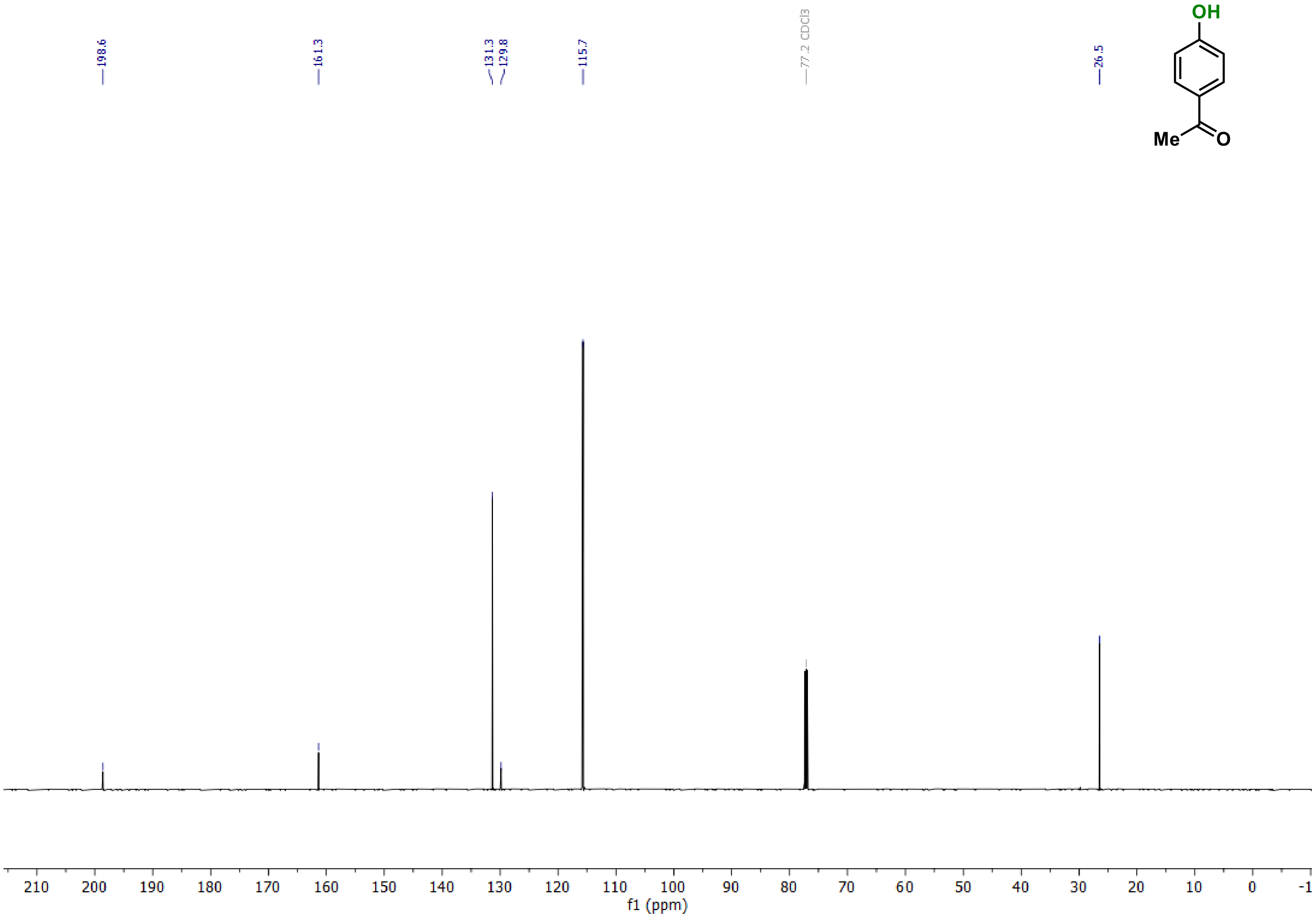

^{1}H NMR of 4-sulfonamidephenol (19)

CD_3CN, 298 K

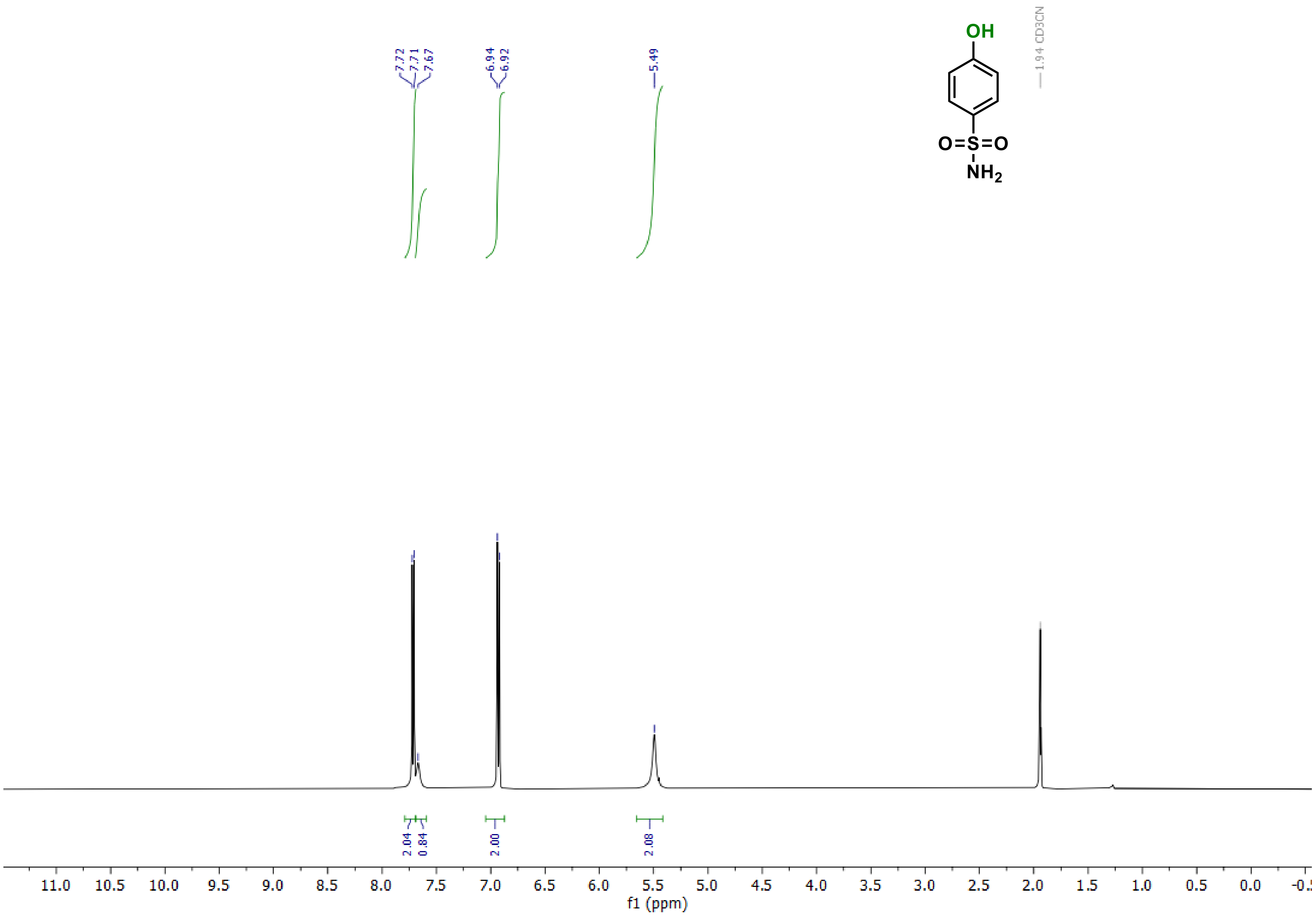

^{13}C NMR of 4-sulfonamidephenol (19)

CD_3CN, 298 K

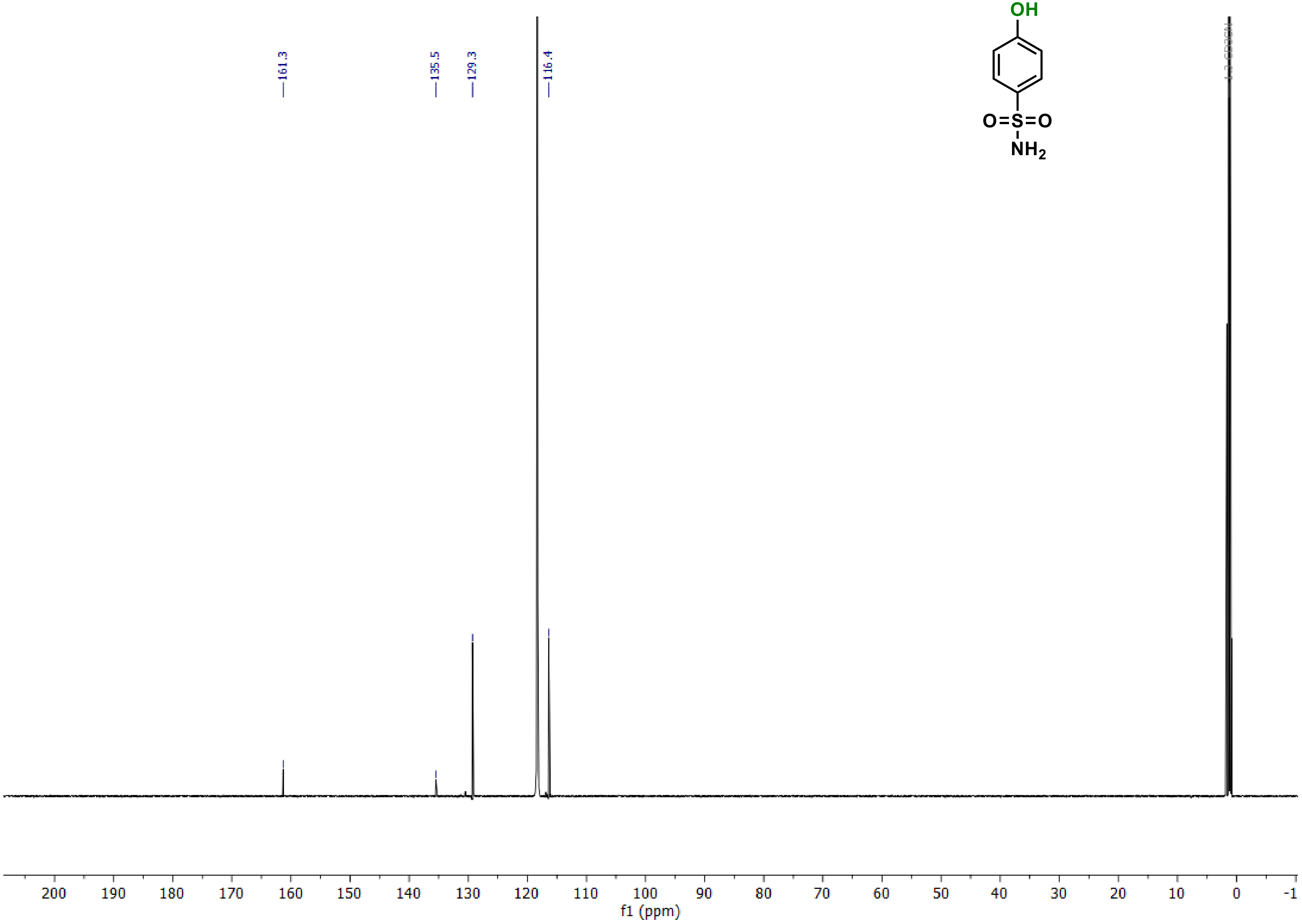

^{1}H NMR of 4-(methylsulfonyl)phenol (20)

$CDCl_3$, 298 K

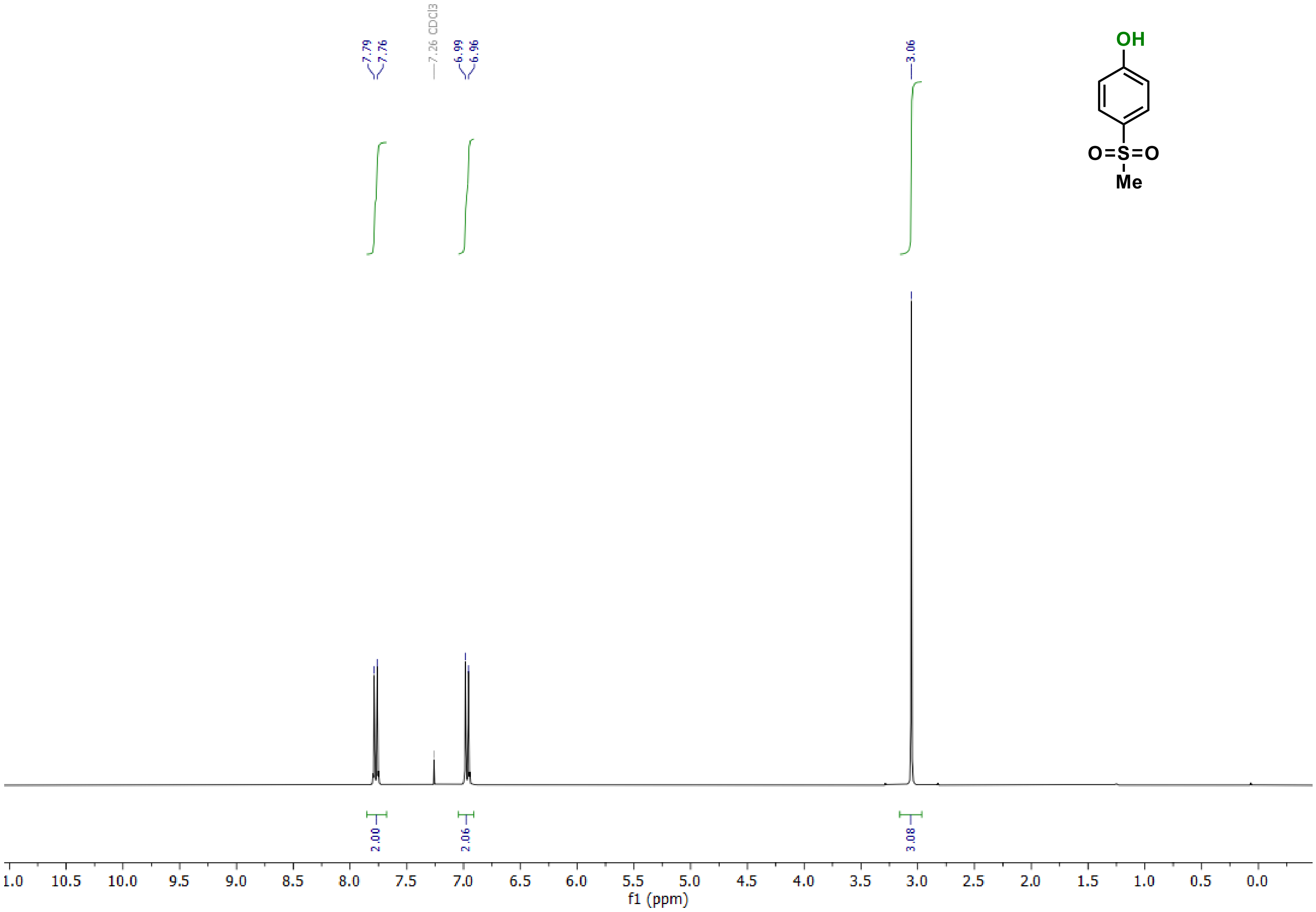

^{13}C NMR of 4-(methylsulfonyl)phenol (20)

$CDCl_3$, 298 K

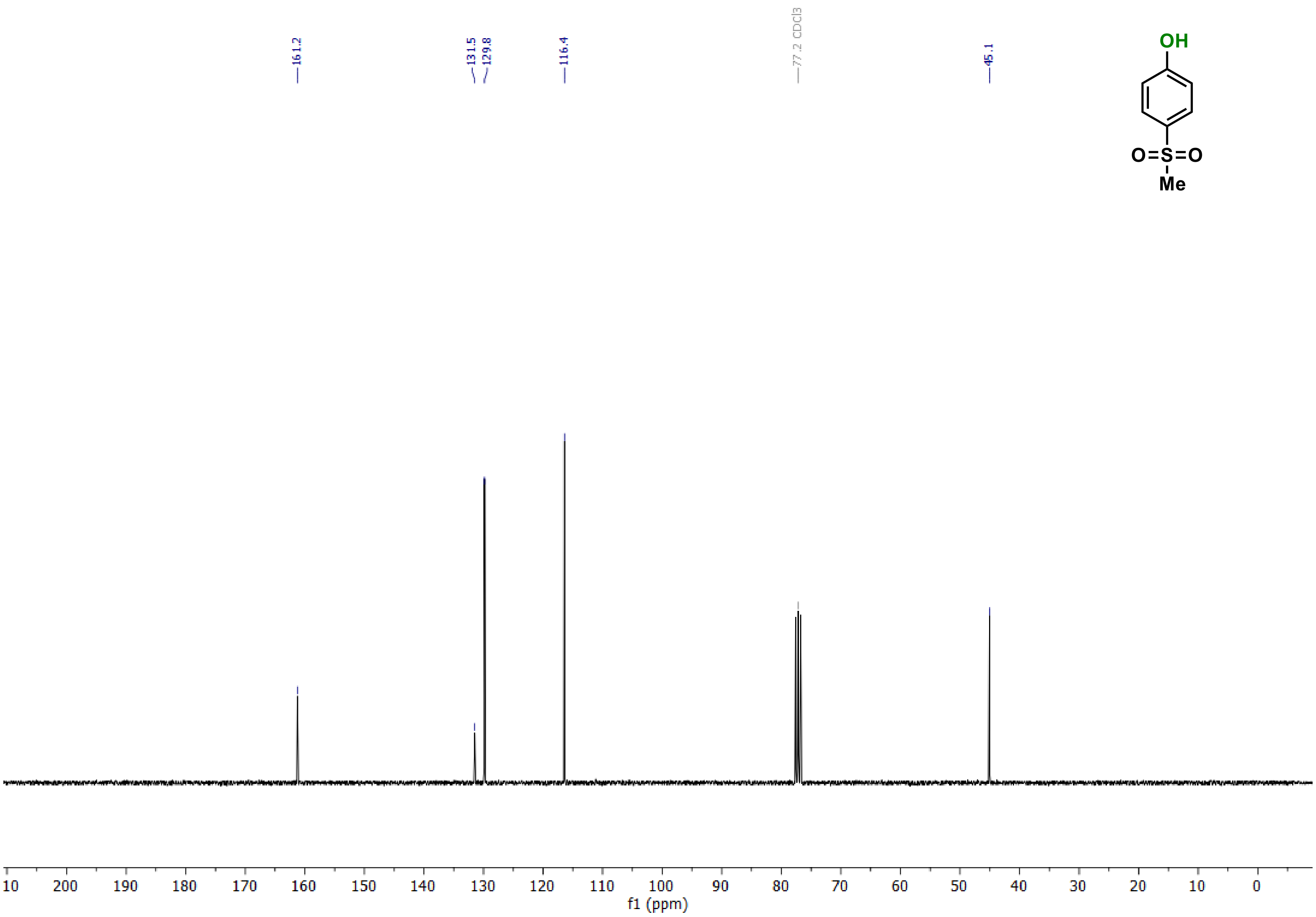

^{1}H NMR of 2-naphthol (21)

$CDCl_3$, 298 K

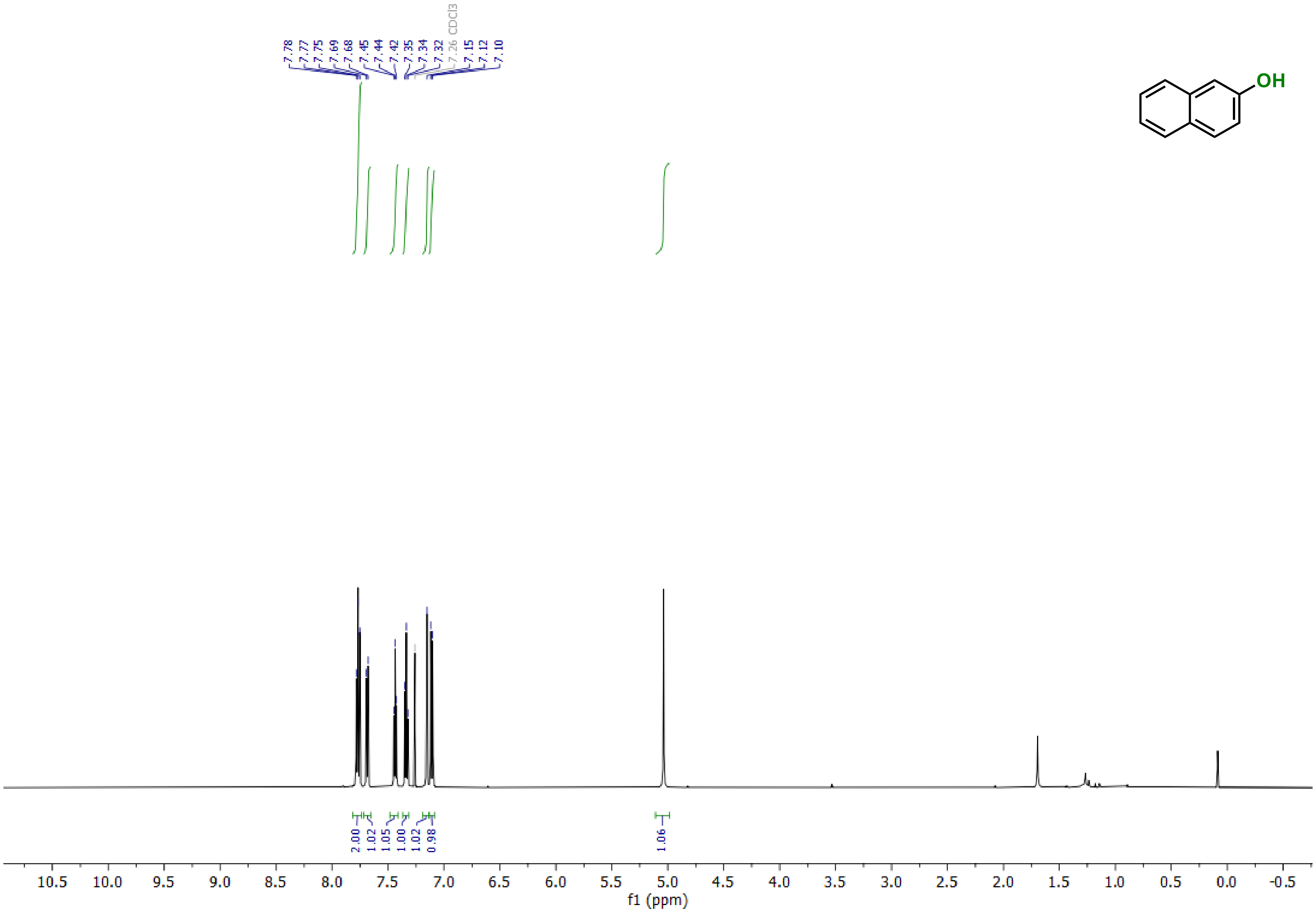

^{13}C NMR of 2-naphthol (21)

$CDCl_3$, 298 K

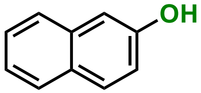

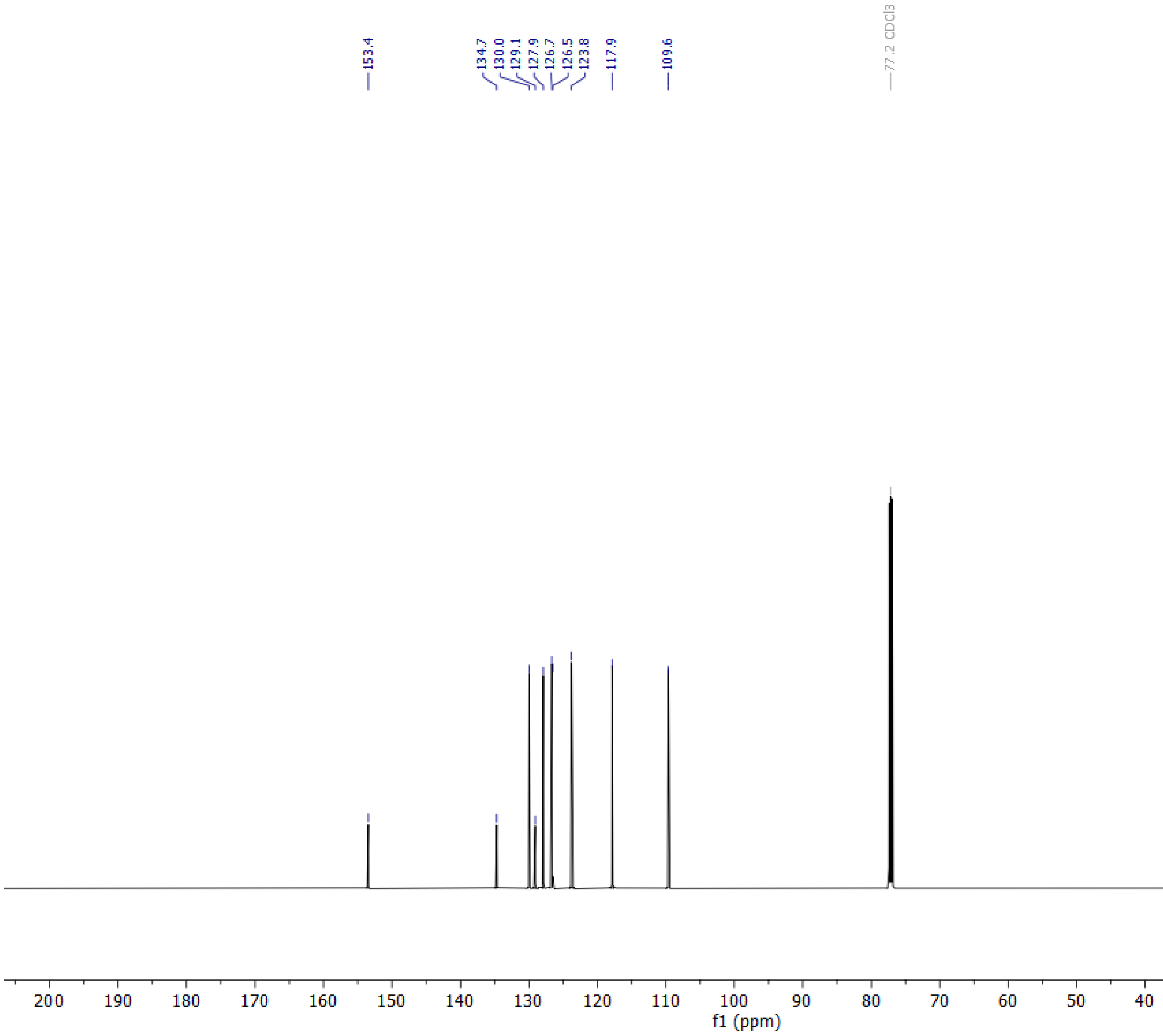

^{1}H NMR of (4-hydroxyphenyl)(morpholino)methanone (22)

$CDCl_3$, 298 K

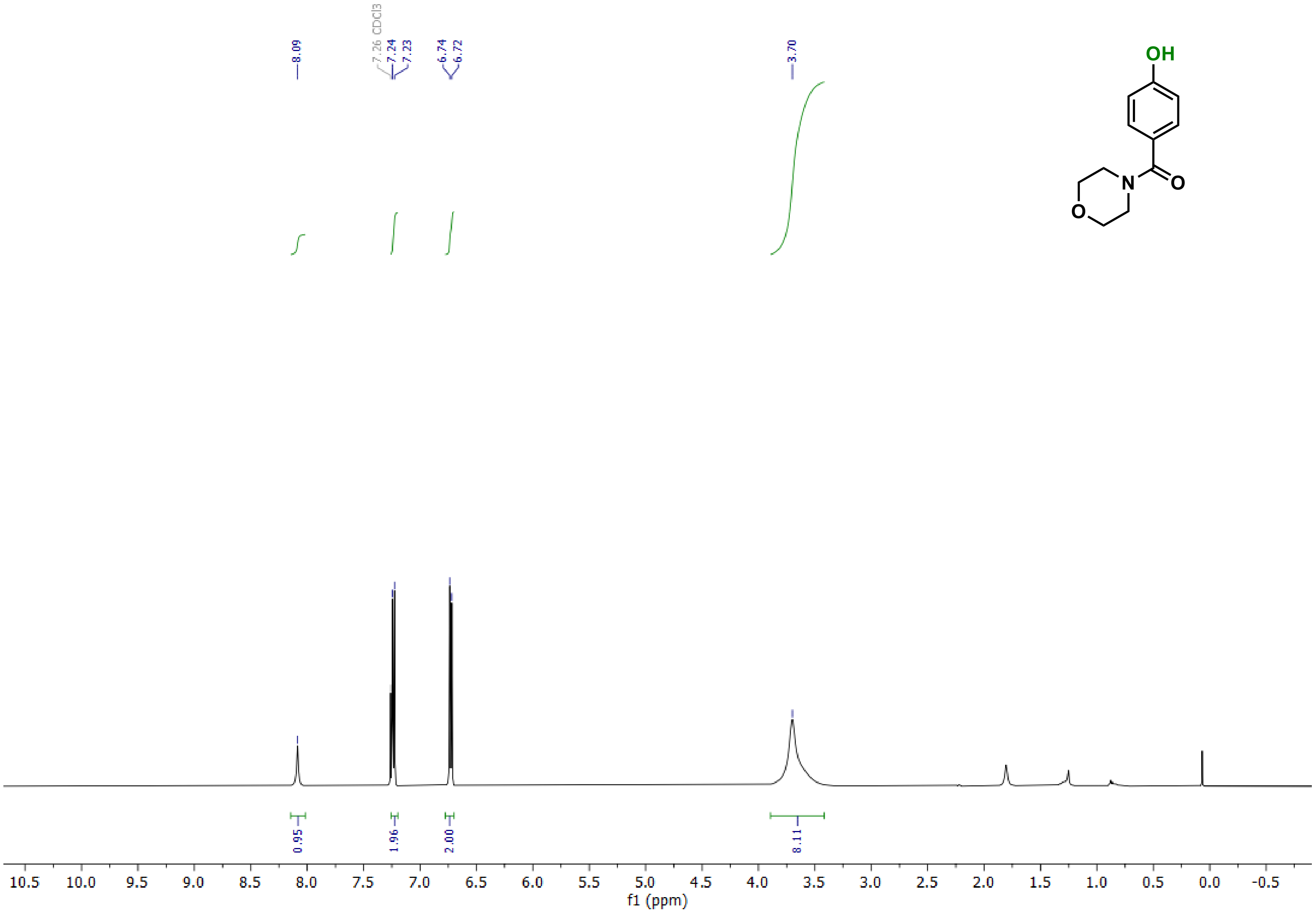

^{13}C NMR of 4-(morpholinocarbonyl)phenone (22)

$CDCl_3$, 298K

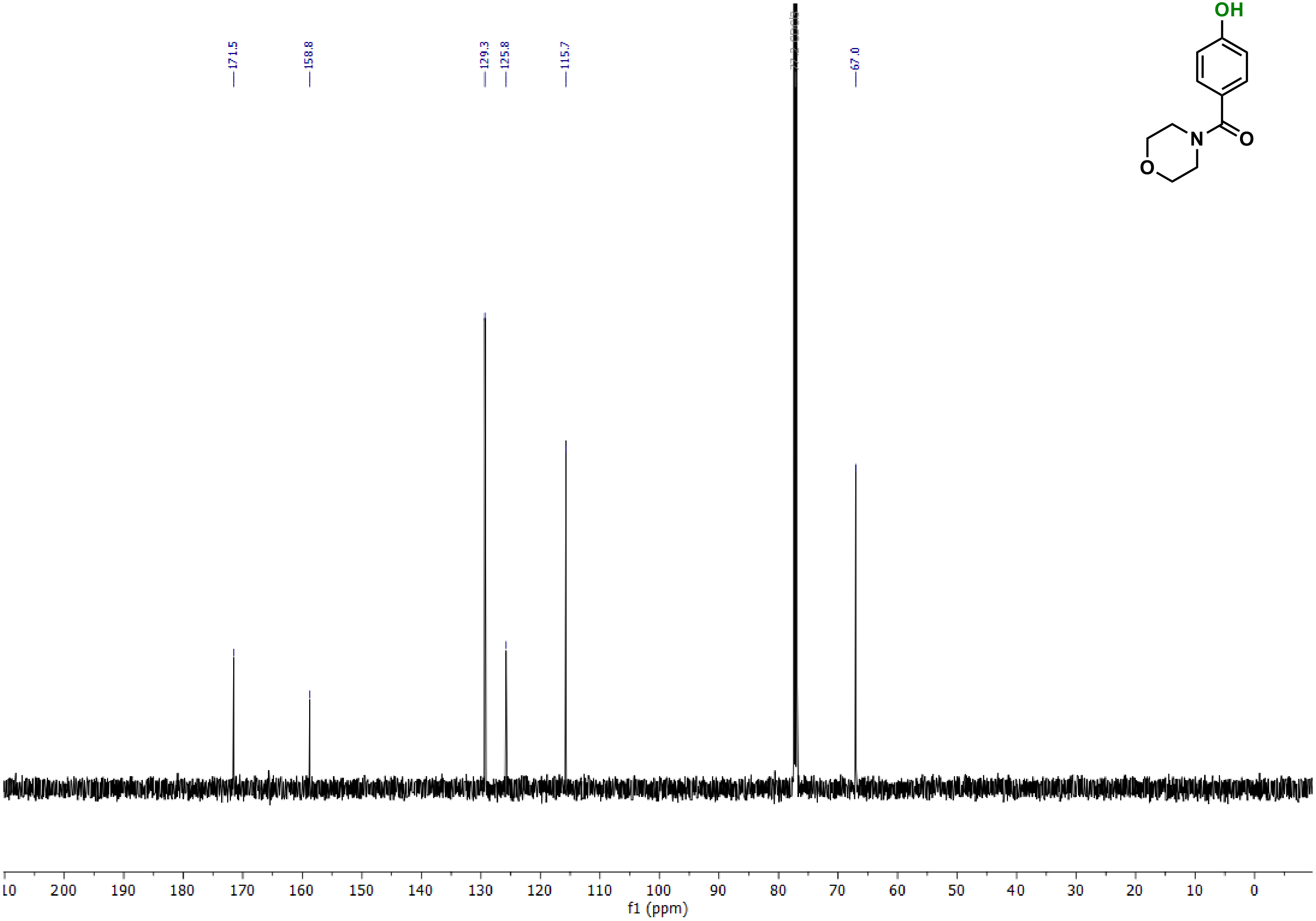

^{1}H NMR of 4-(*trans*-4-pentylcyclohexyl)phenol (23)

$CDCl_3$, 298 K

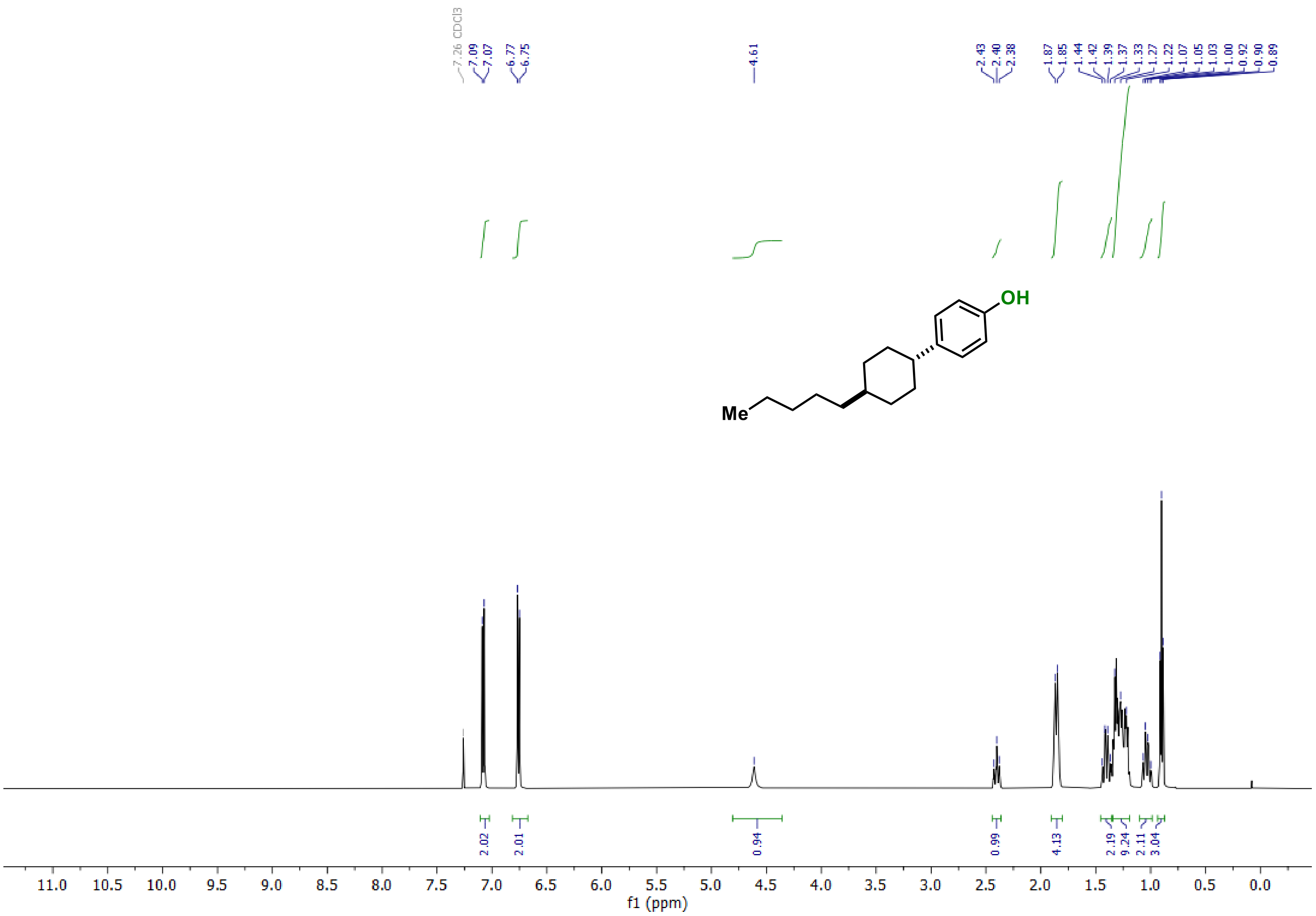

^{13}C NMR of 4-(*trans*-4-pentylcyclohexyl)phenol (23)

$CDCl_3$, 298 K

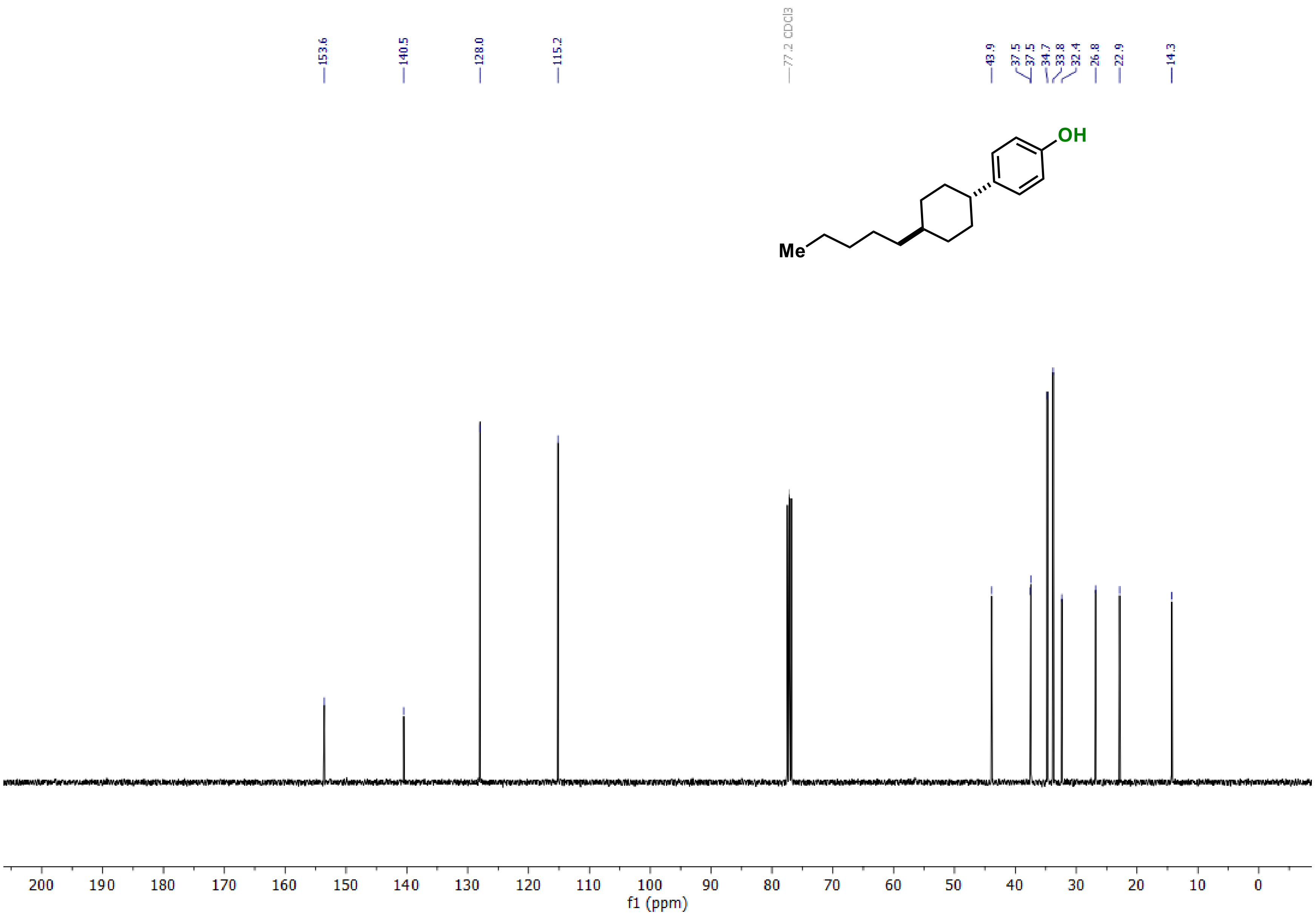

^{1}H NMR of celecoxib derivative 24

CD_3CN, 298 K

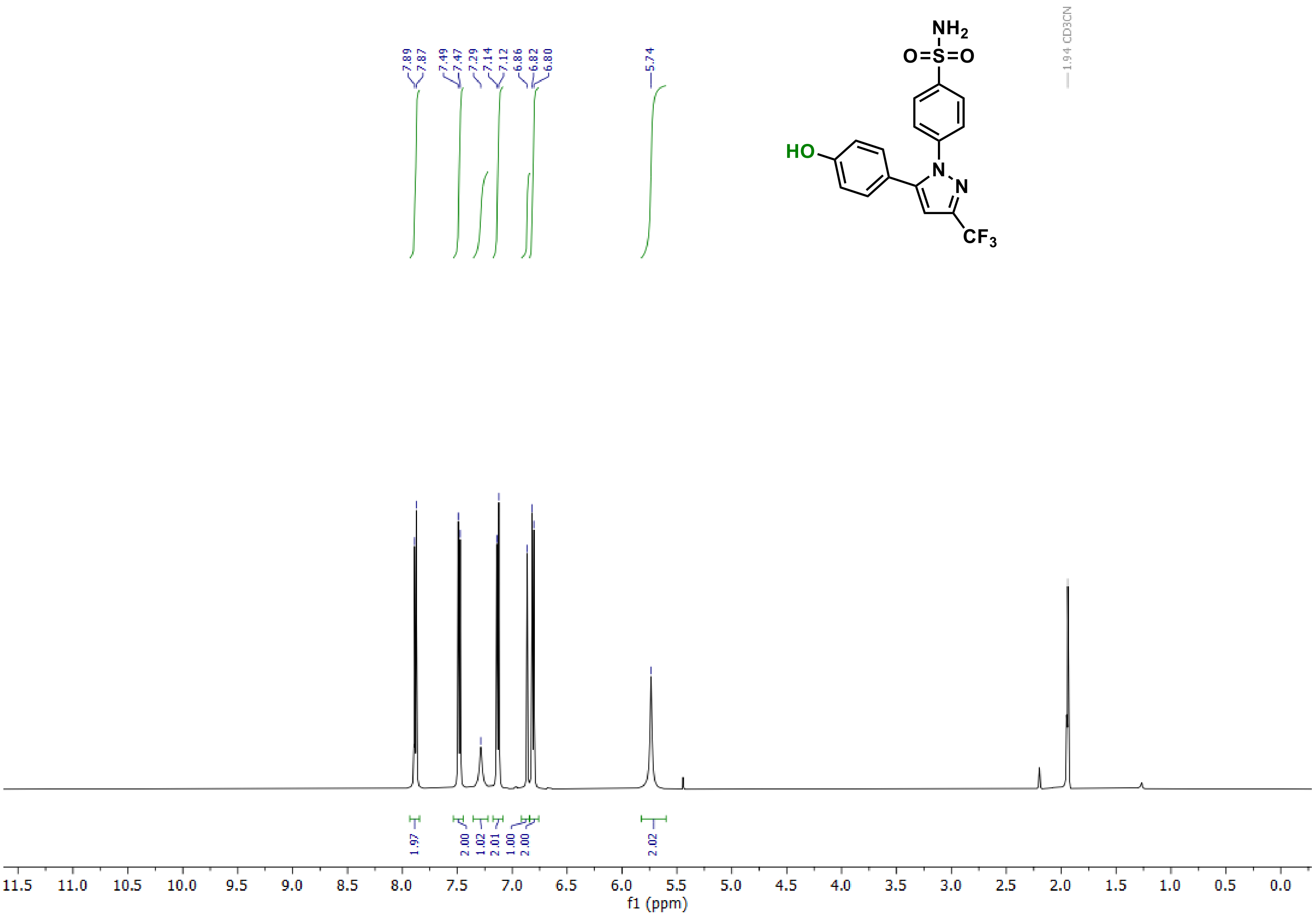

^{13}C NMR of celecoxib derivative 24

CD_3CN, 298 K

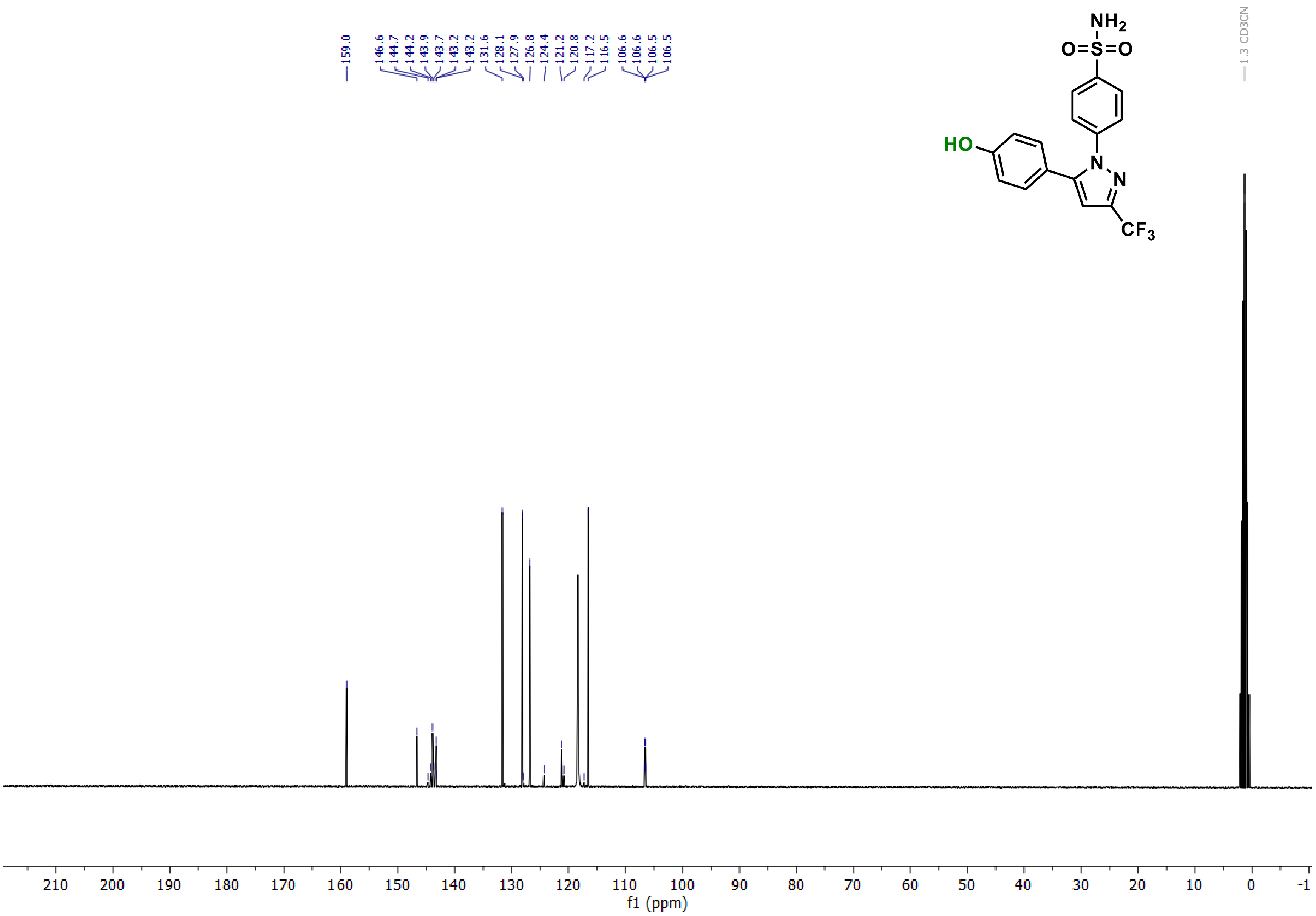

^{19}F NMR of celecoxib derivative 24

CD_3CN, 298 K

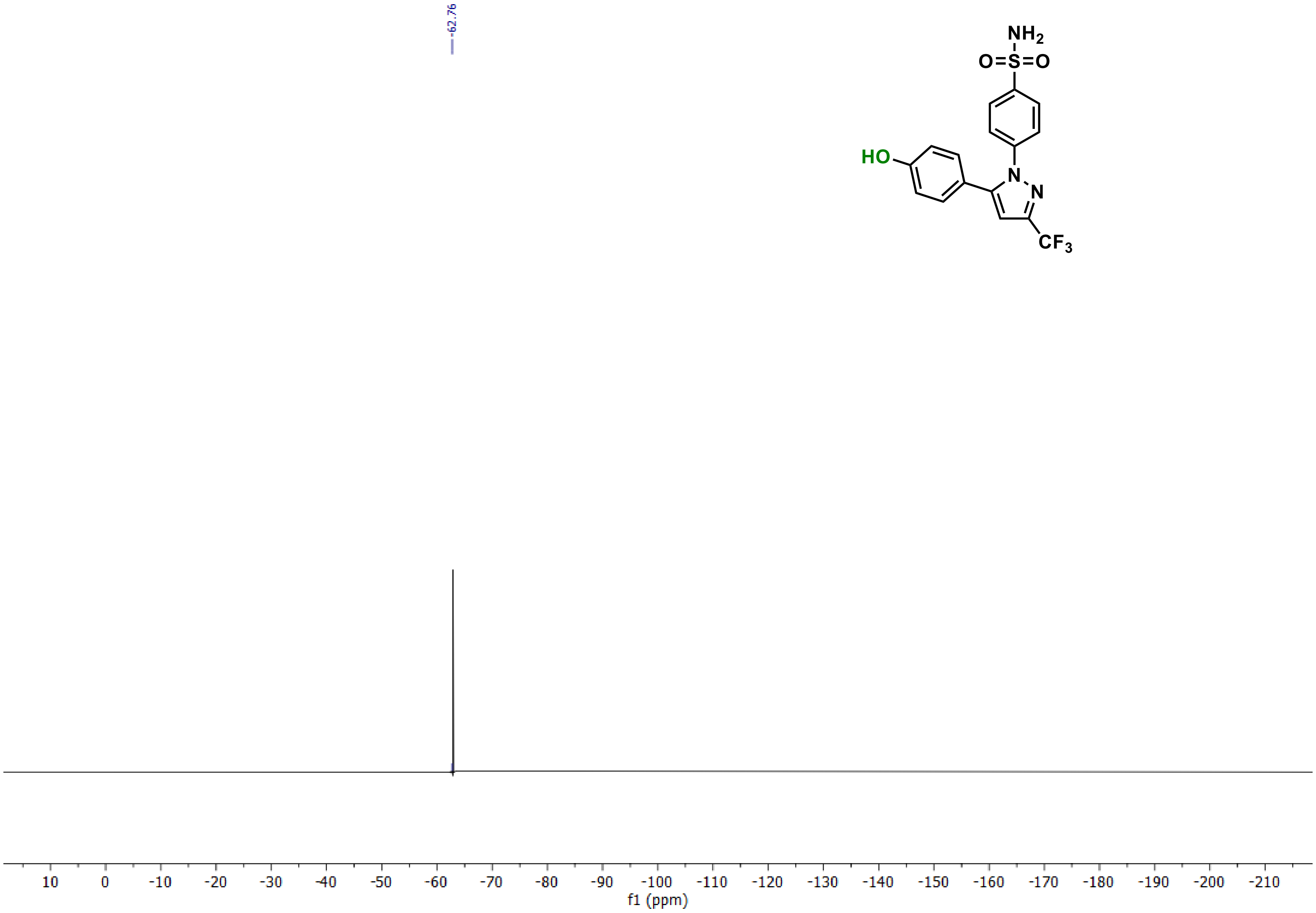

^{1}H NMR of 4-bromophenol (25)

CD_2Cl_2, 298 K

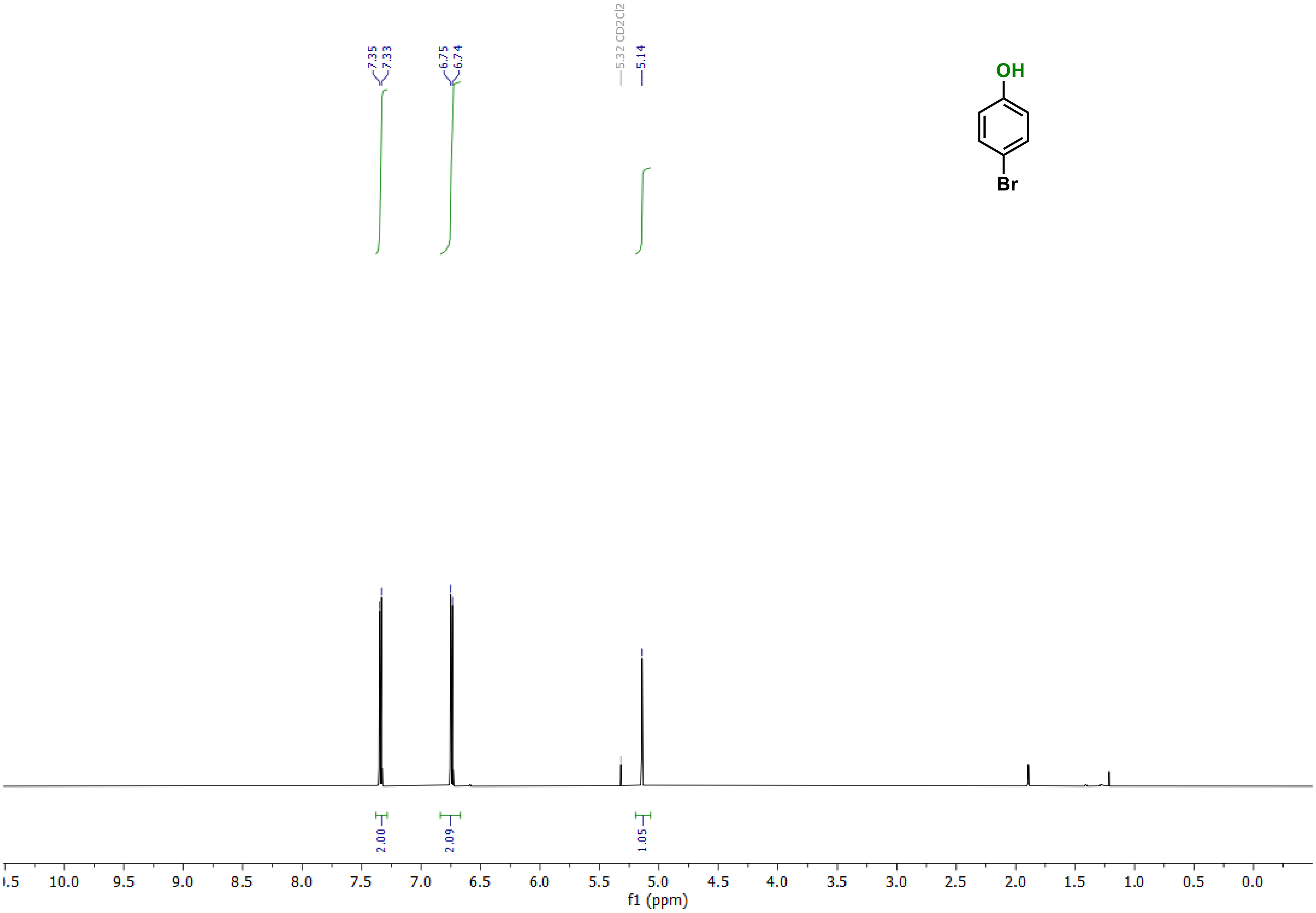

^{13}C NMR of 4-bromophenol (25)

CD_2Cl_2, 298 K

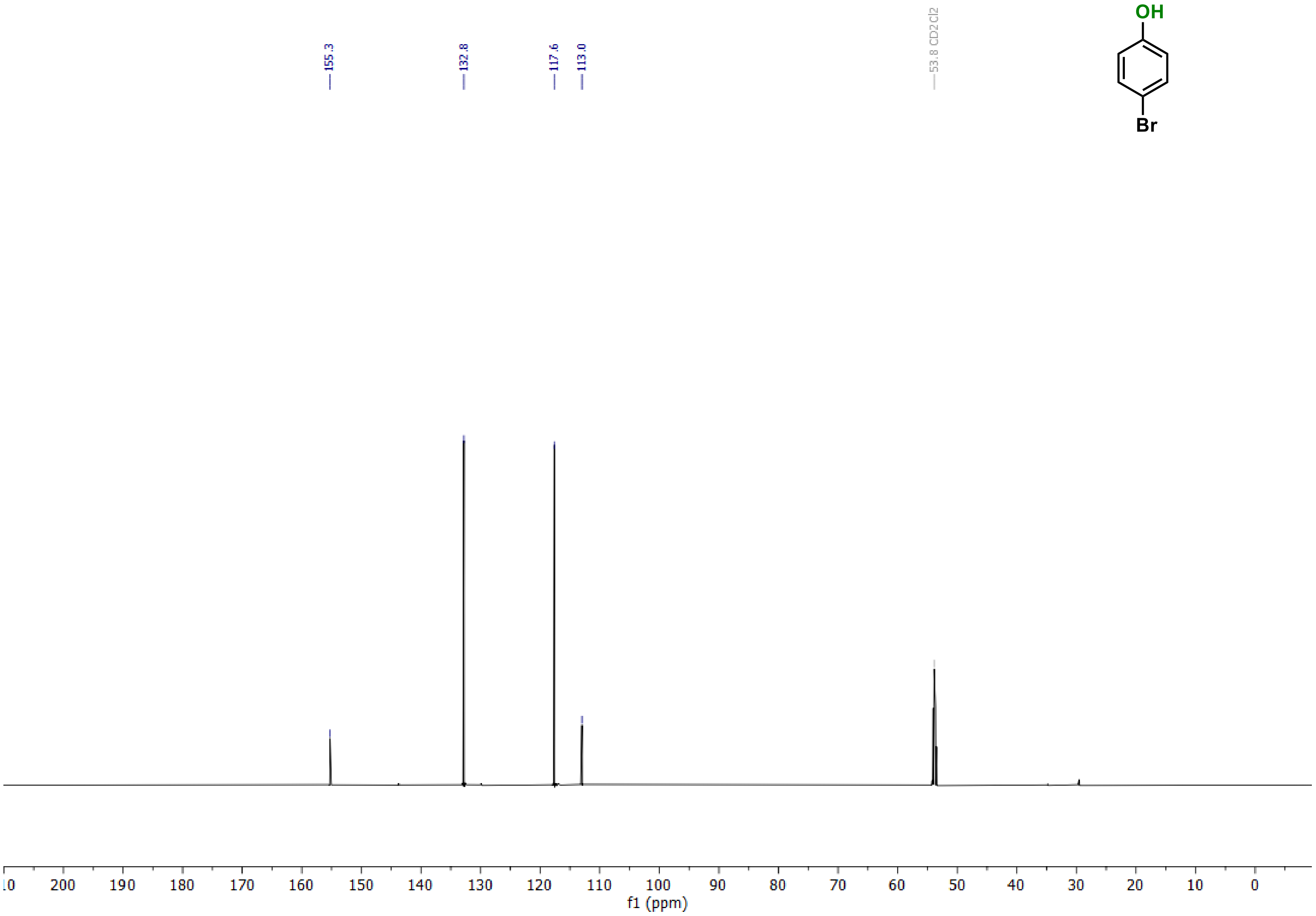

^{1}H NMR of 4-nitrophenol (26)

CD_3CN, 298 K

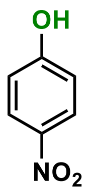

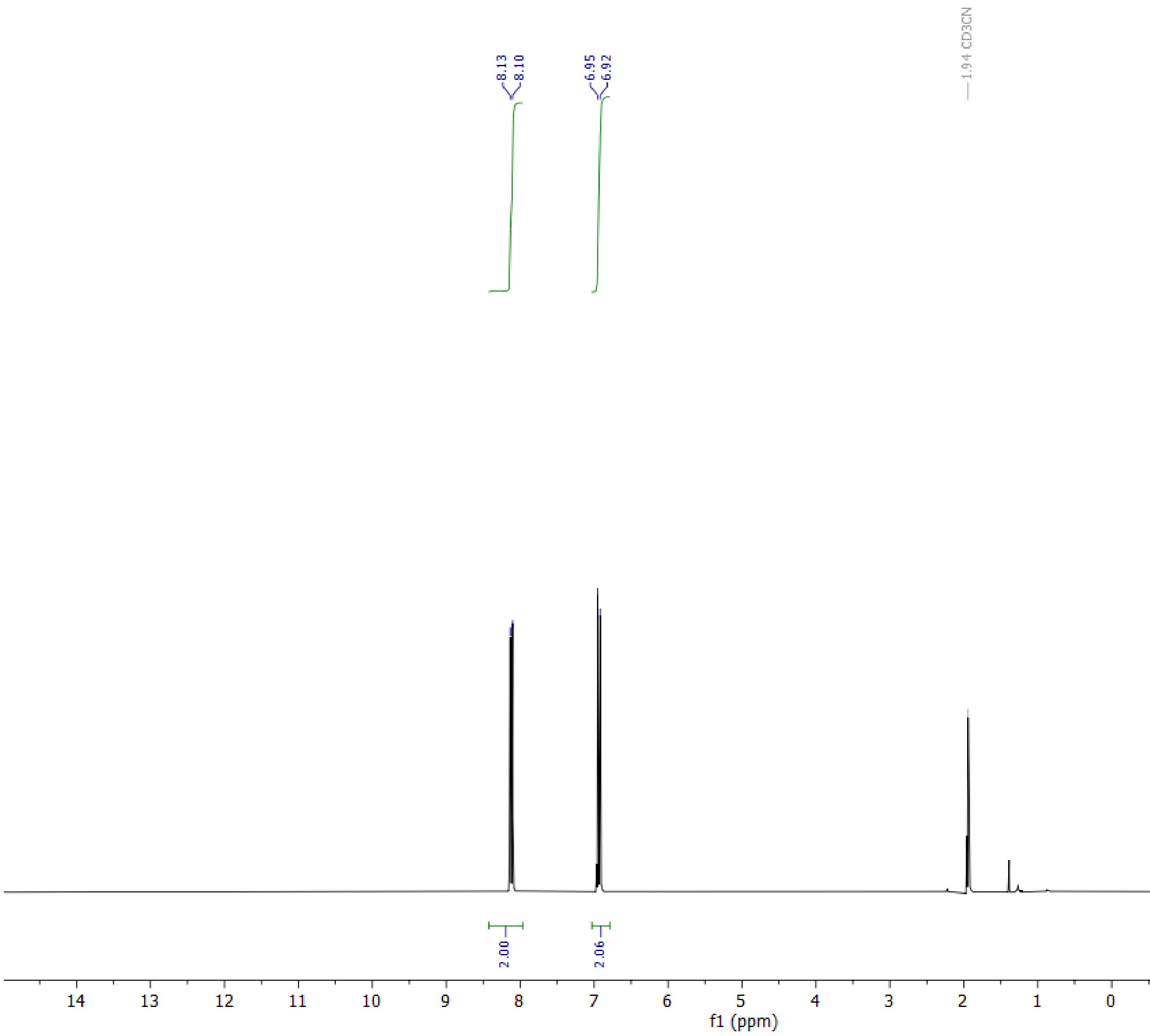

^{13}C NMR of 4-nitrophenol (26)

CD_3CN, 298 K

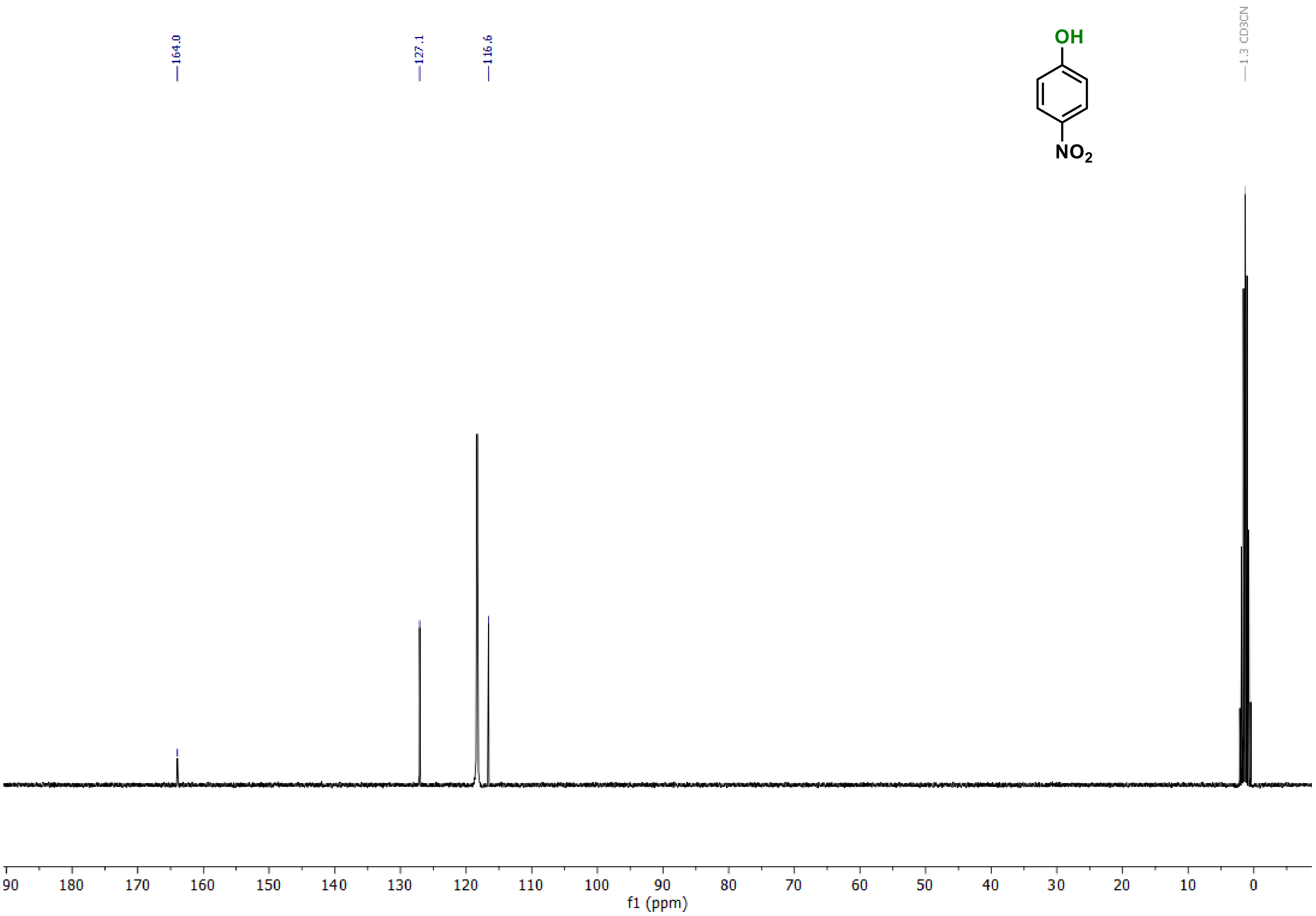

^{1}H NMR of 6-hydroxy-1-tetralone (28)

$CDCl_3$, 298 K

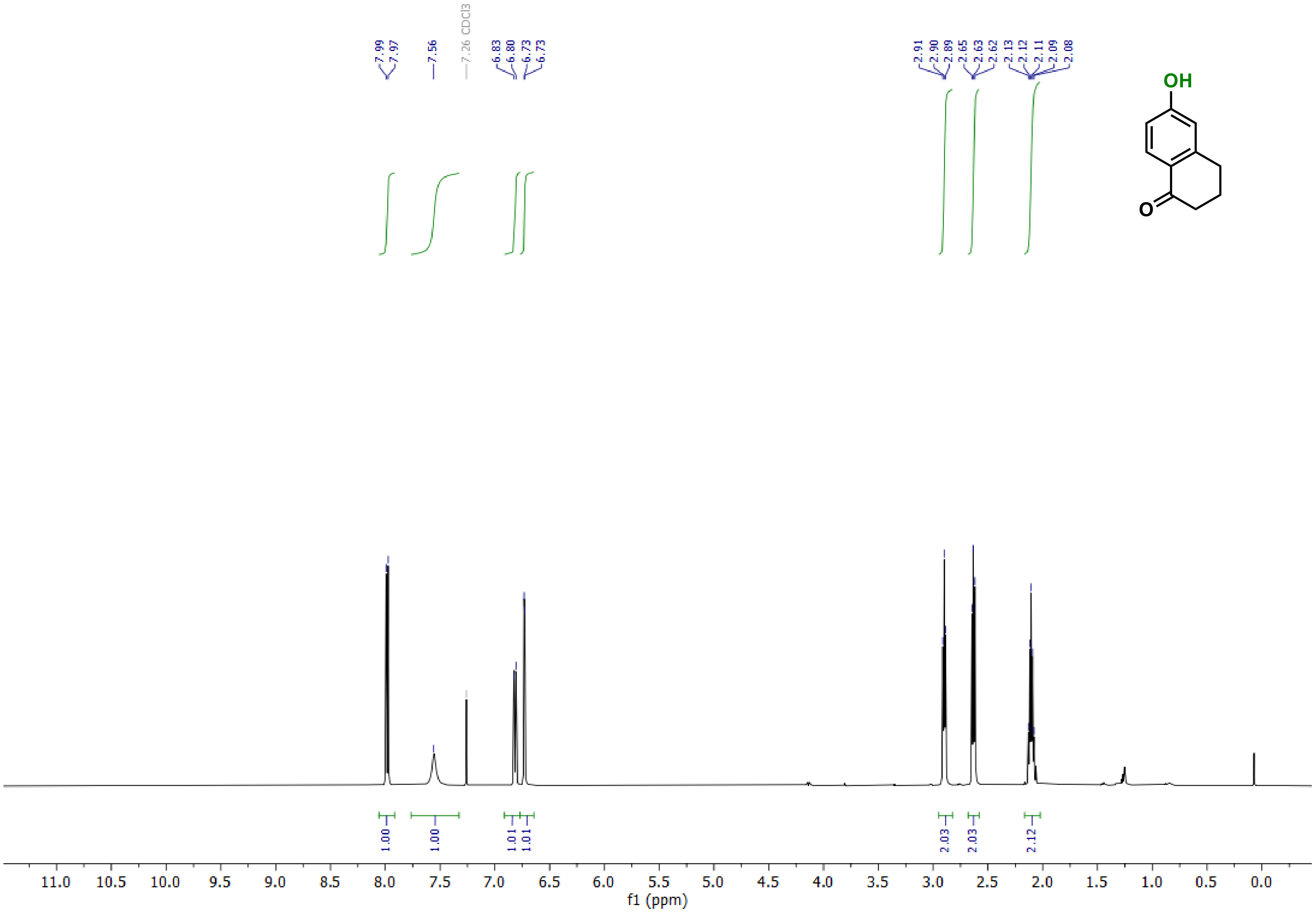

^{13}C NMR of 6-hydroxy-1-tetralone (28)

$CDCl_3$, 298 K

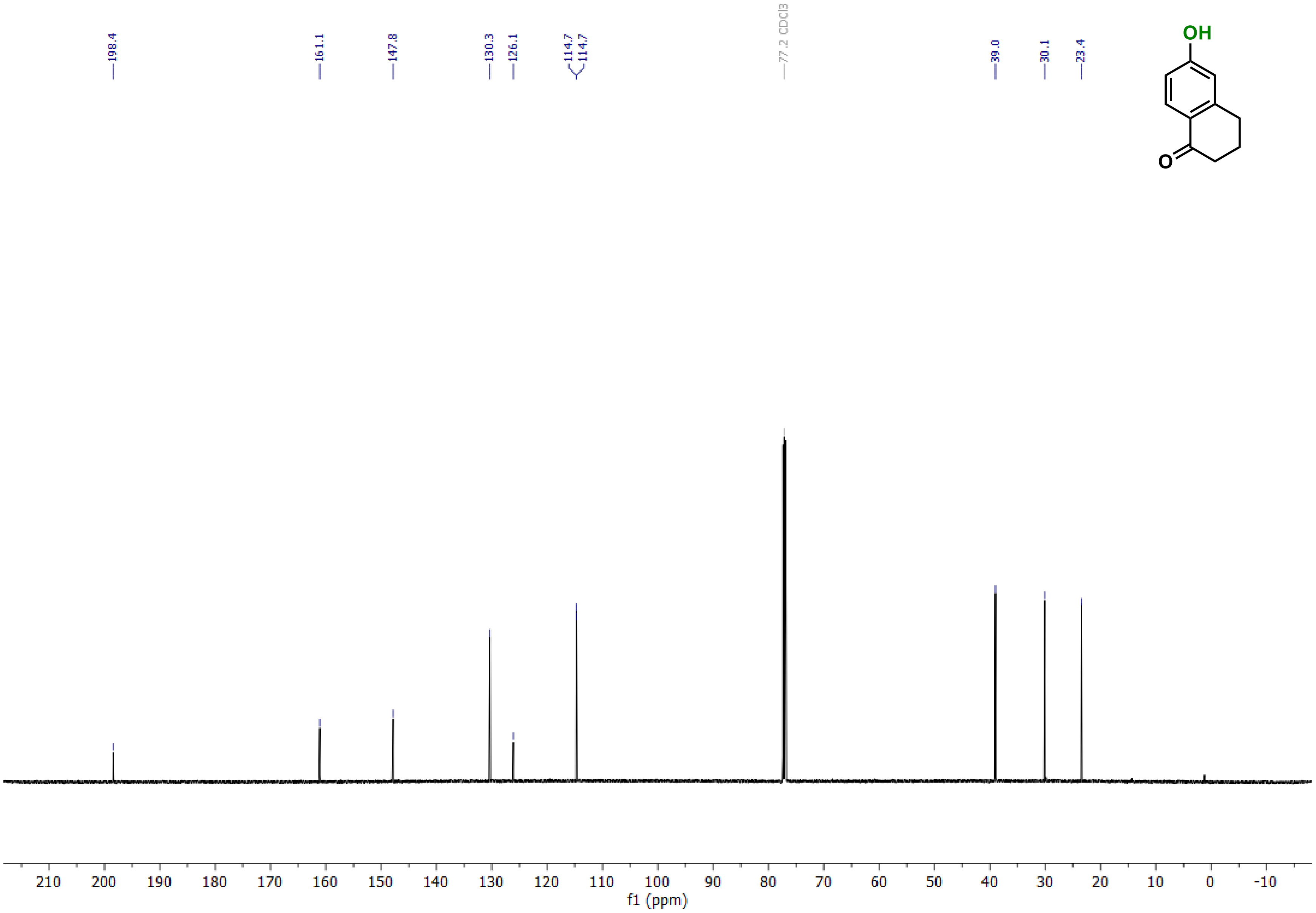

^{1}H NMR of 2,2'-(5-Hydroxy-1,3-phenylene)bis(2-methylpropanenitrile) (29)

$CDCl_3$, 298 K

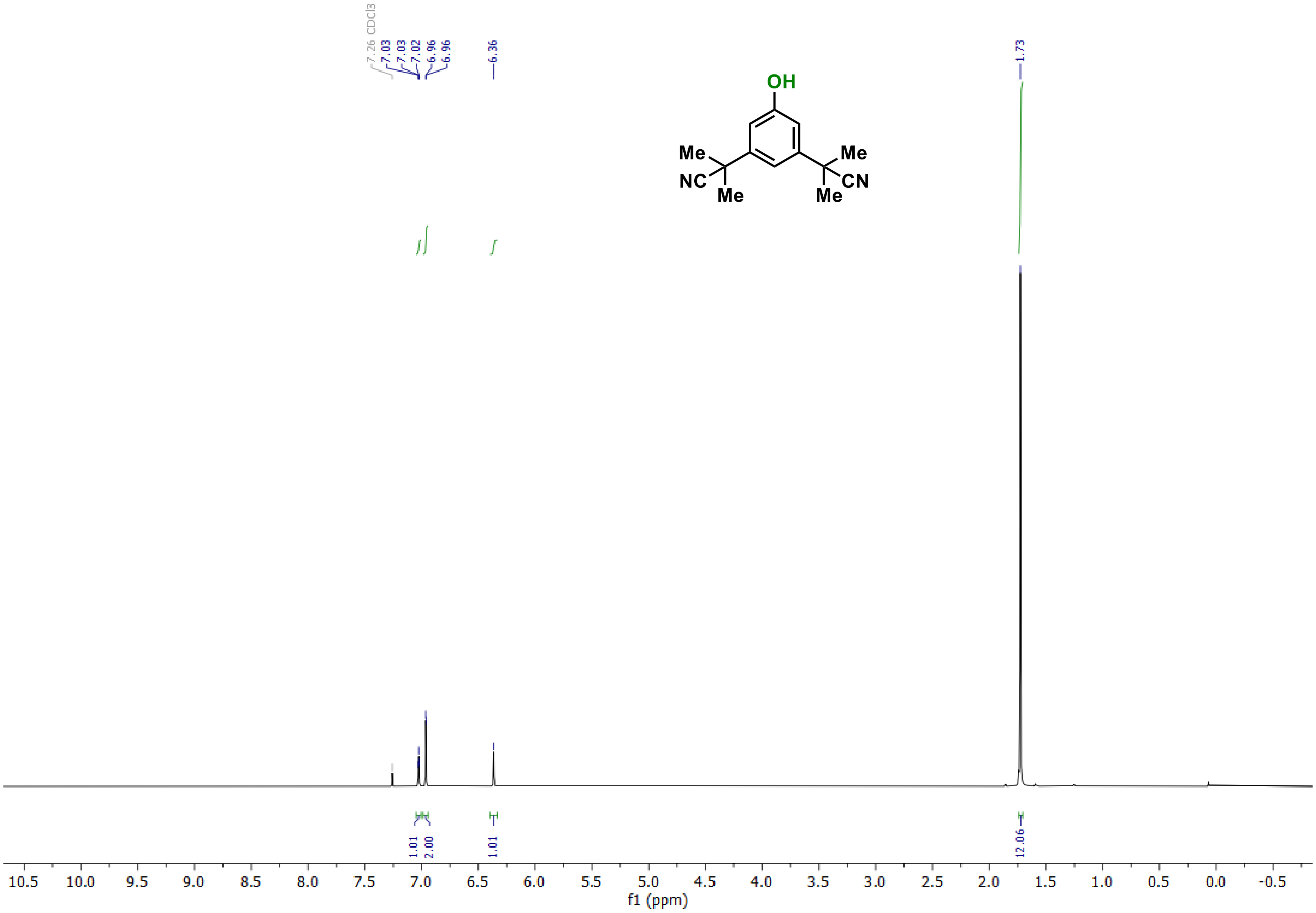

^{13}C NMR of 2,2'-(5-Hydroxy-1,3-phenylene)bis(2-methylpropanenitrile) (29)

$CDCl_3$, 298 K

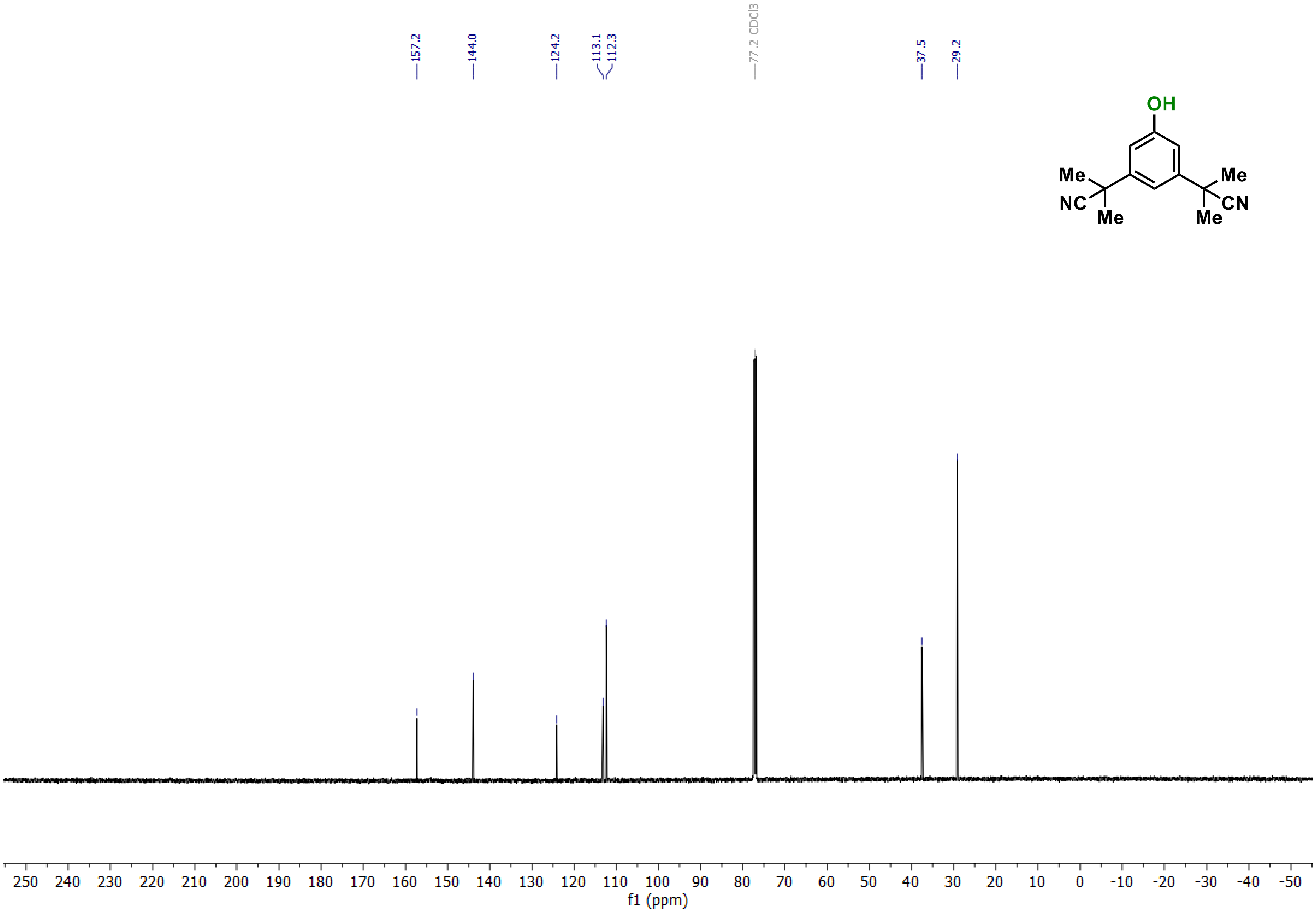

^{1}H NMR of 4-phenylphenol (30)

CD_3CN, 298 K

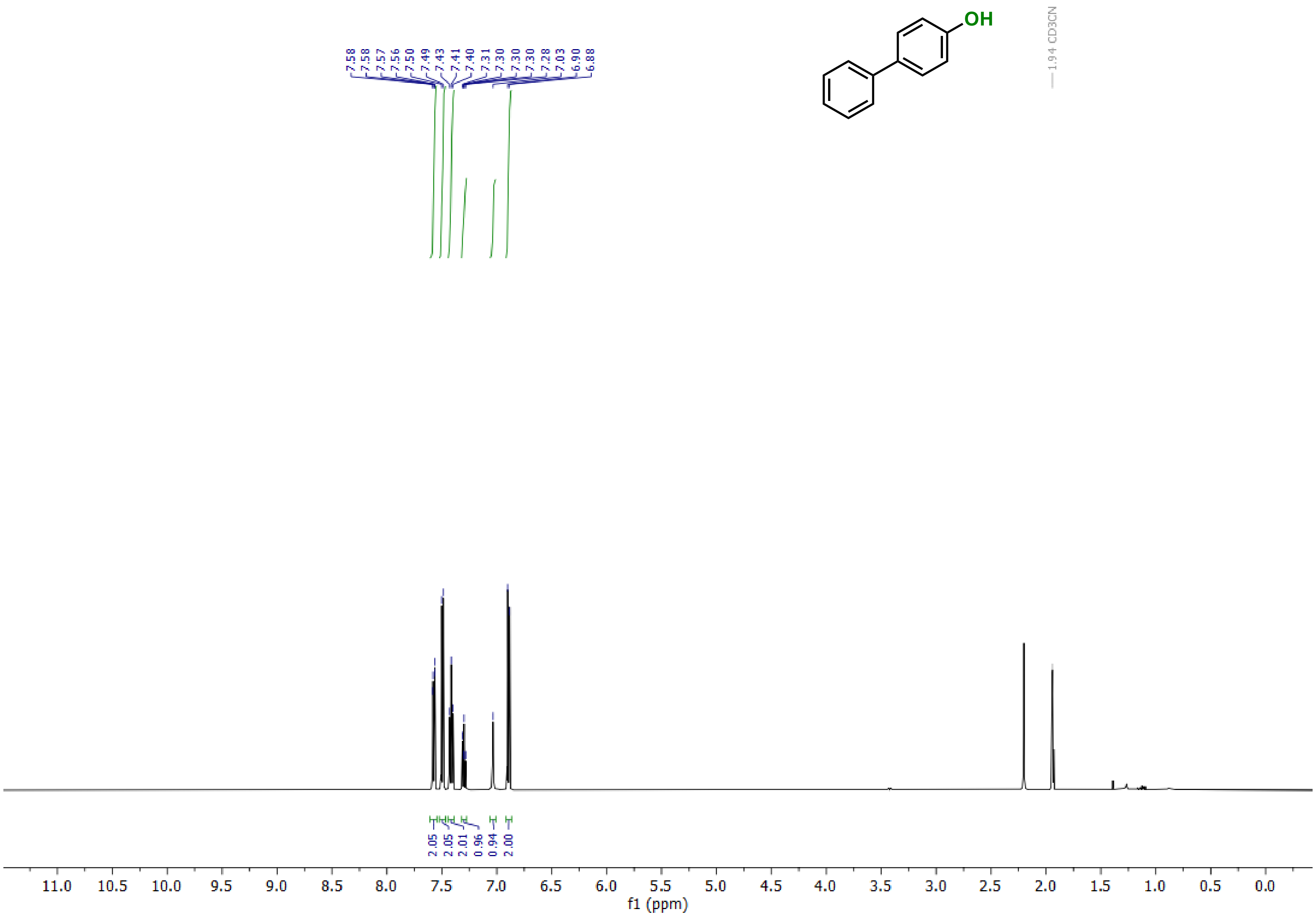

^{13}C NMR of 4-phenylphenol (30)

CD_3CN, 298 K

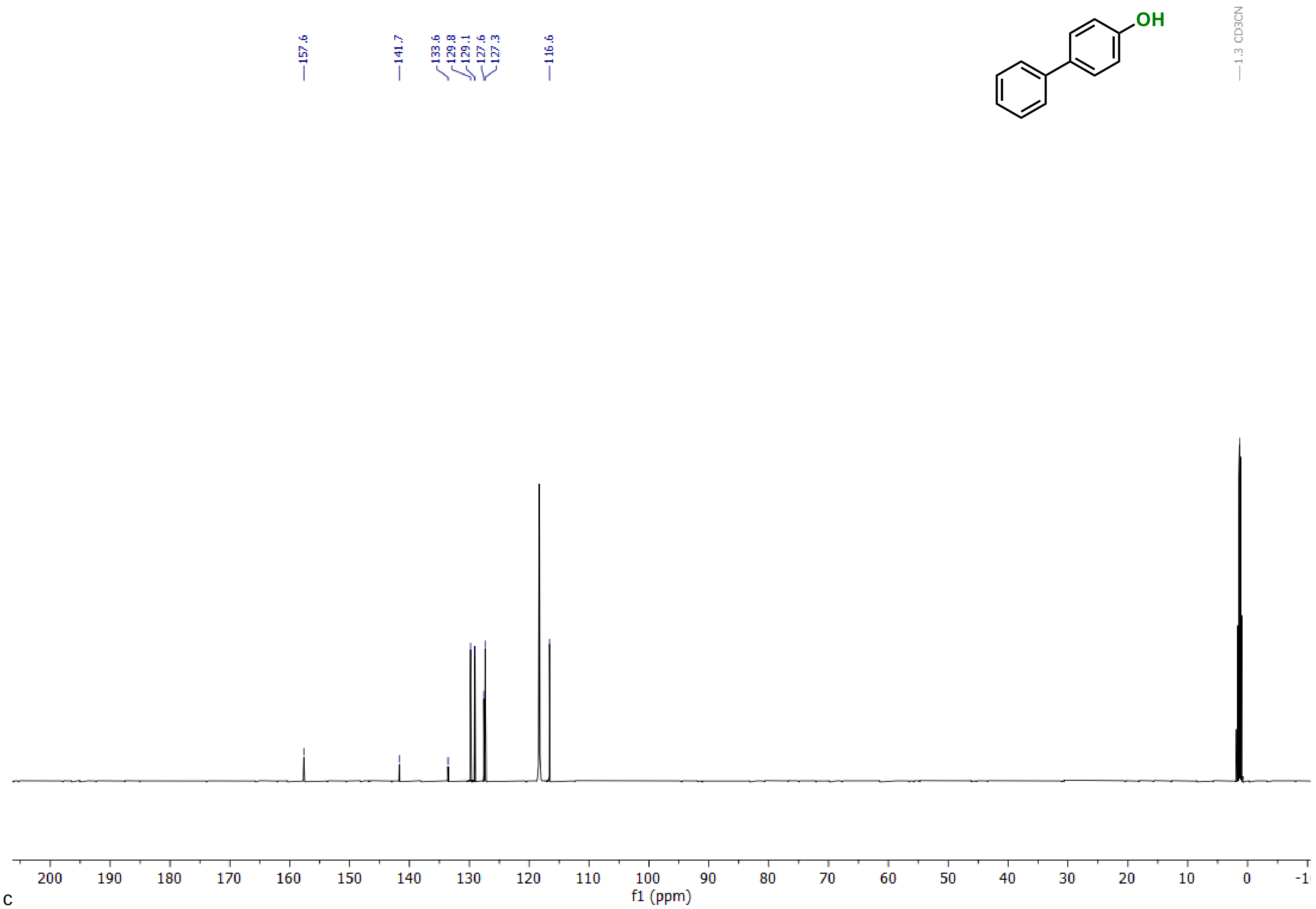

c

^{1}H NMR of triclosan derivative 31

CD$_3$CN, 298 K

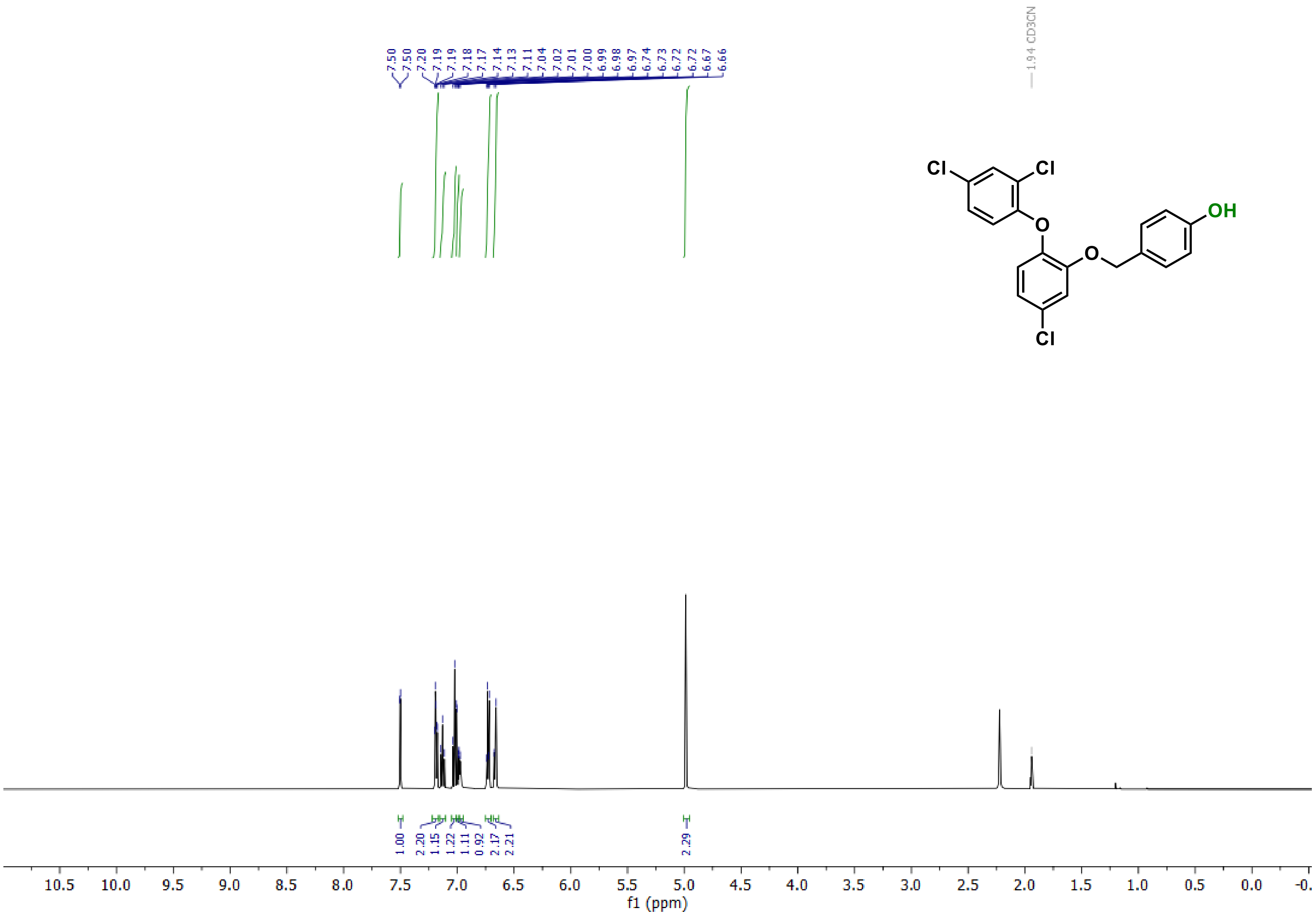

^{13}C NMR of triclosan derivative 31

CD_3CN, 298 K

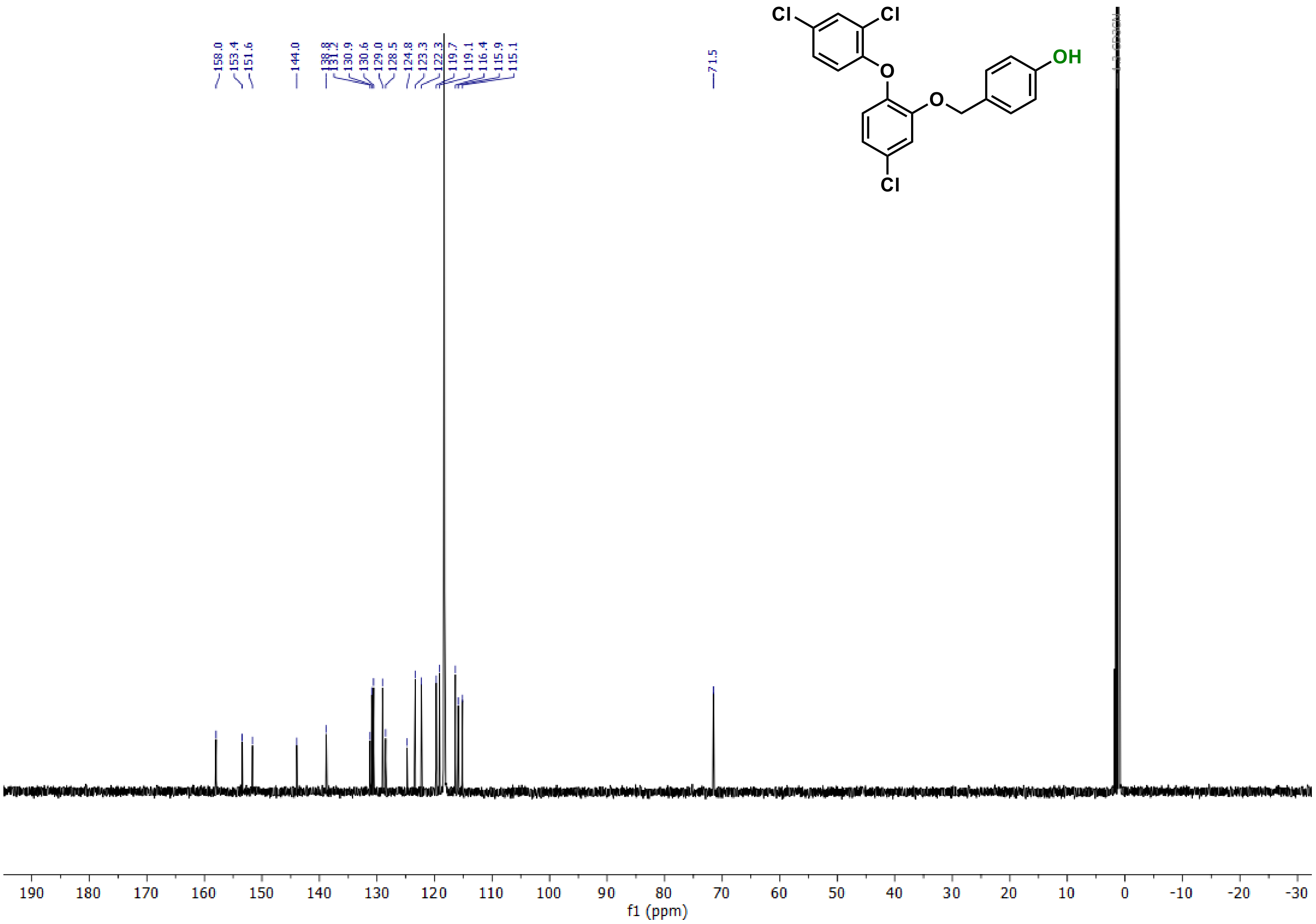

^{1}H NMR of triclosan-derived benzoic acid S31

$(CD_3)_2SO$, 298 K

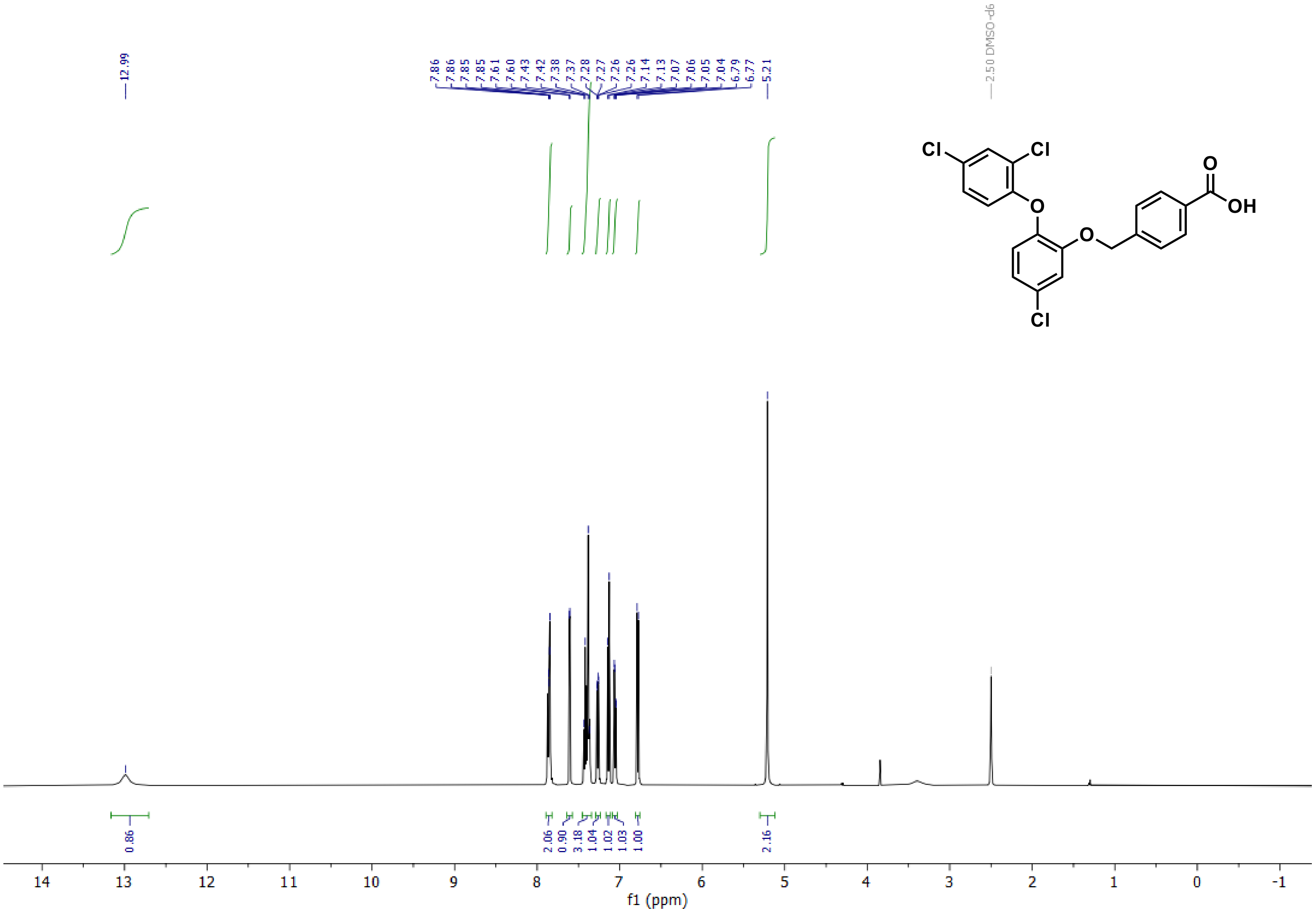

^{13}C NMR of triclosan-derived benzoic acid S31

$(CD_3)_2SO$, 298 K

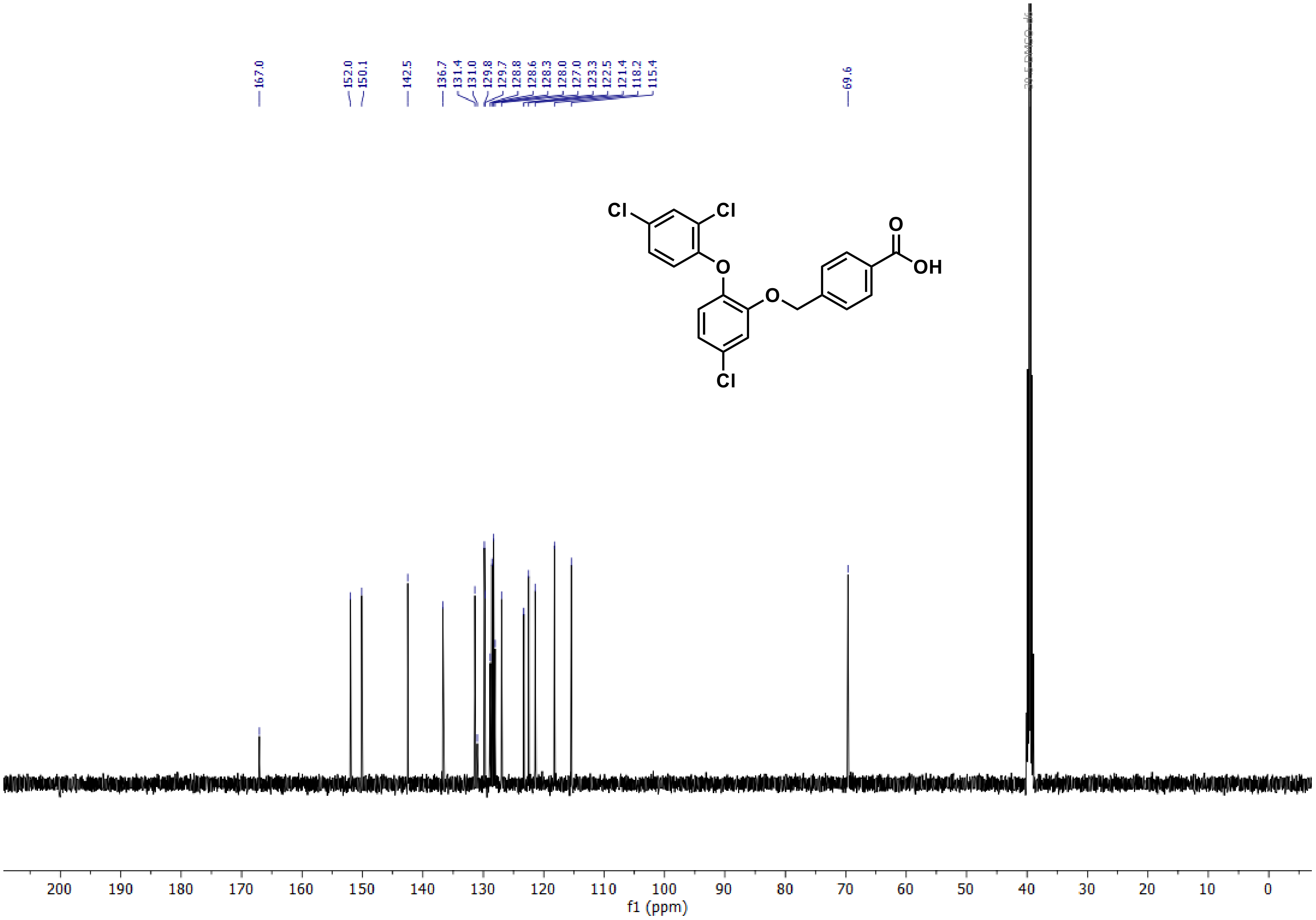

^{1}H NMR of 6H-benzo[c]chromen-6-one (33)

CD_3CN, 298 K

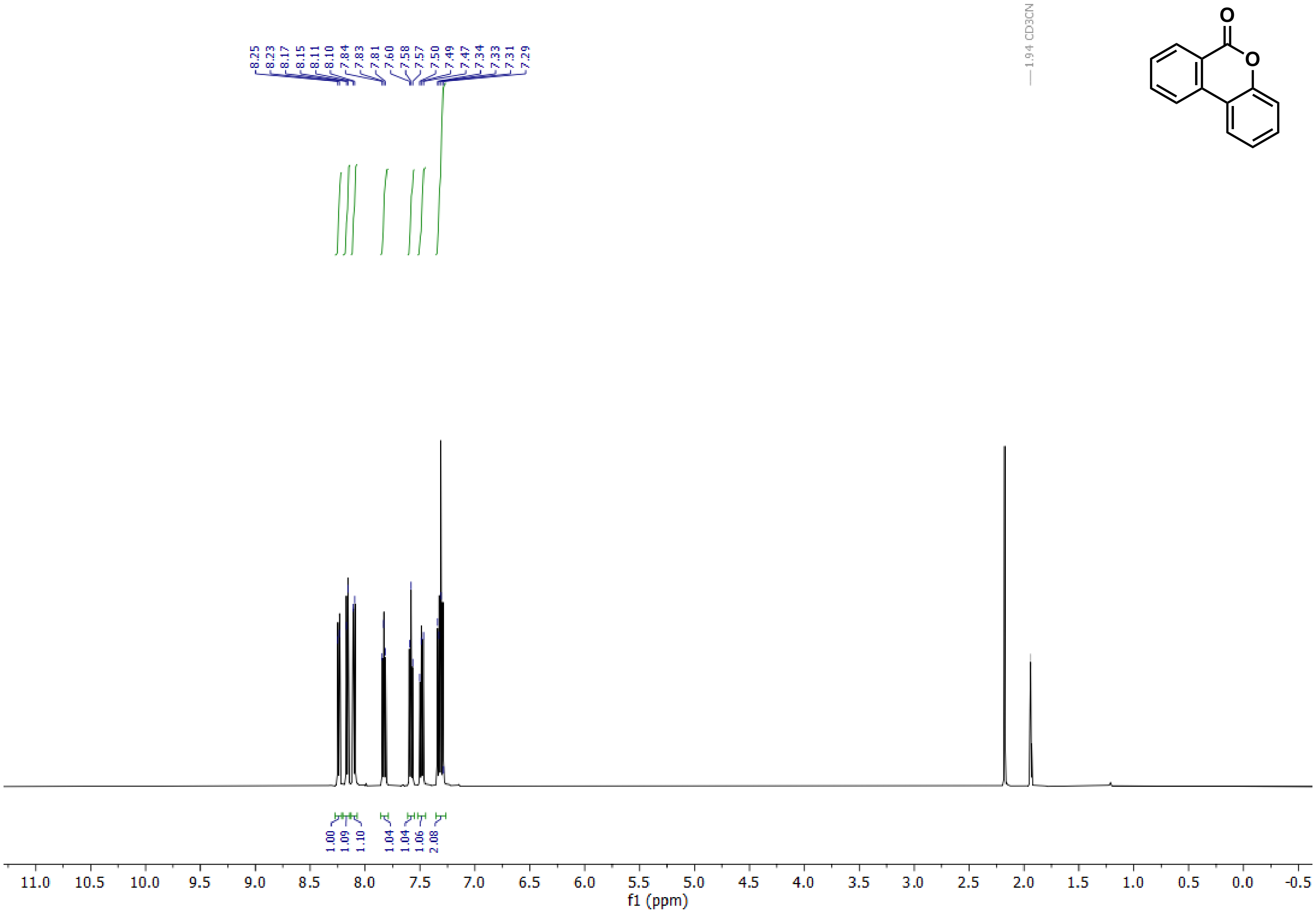

^{13}C NMR of 6H-benzo[c]chromen-6-one (33)

CD_3CN, 298 K

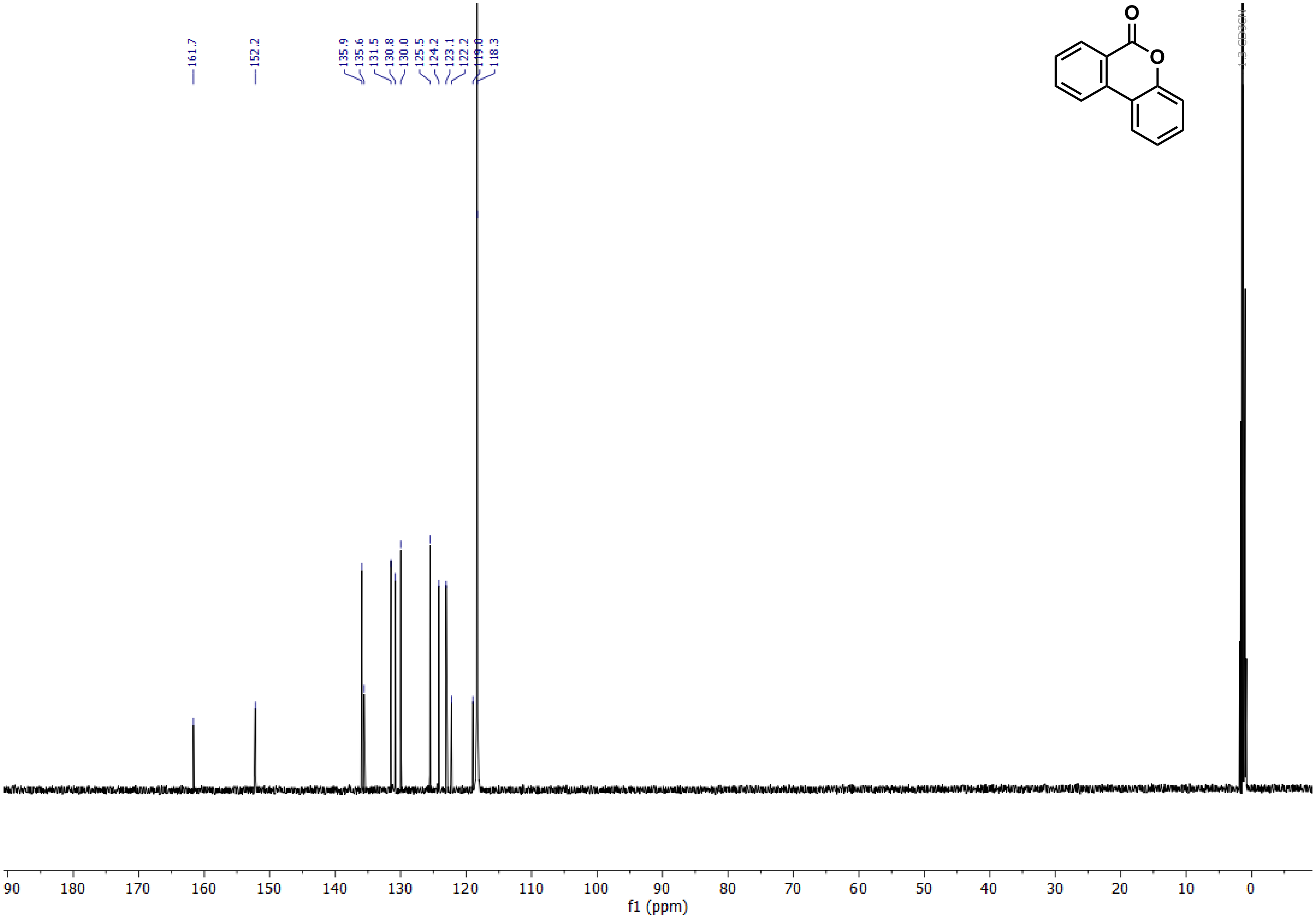

^{1}H NMR of 4-methoxy-1,1'-biphenyl (35)

CD_2Cl_2, 298 K

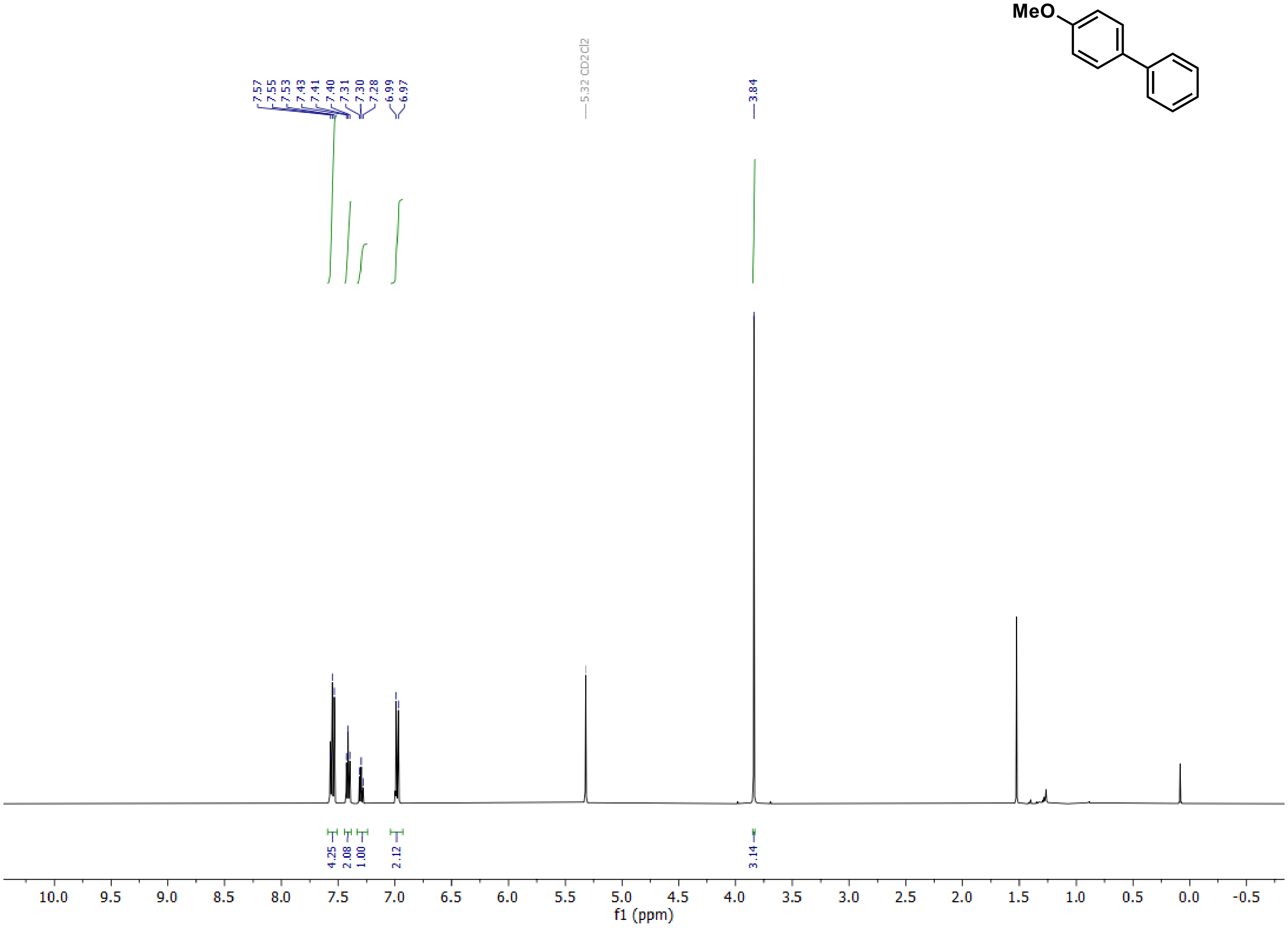

^{13}C NMR of 4-methoxy-1,1'-biphenyl (35)

CD_2Cl_2, 298 K

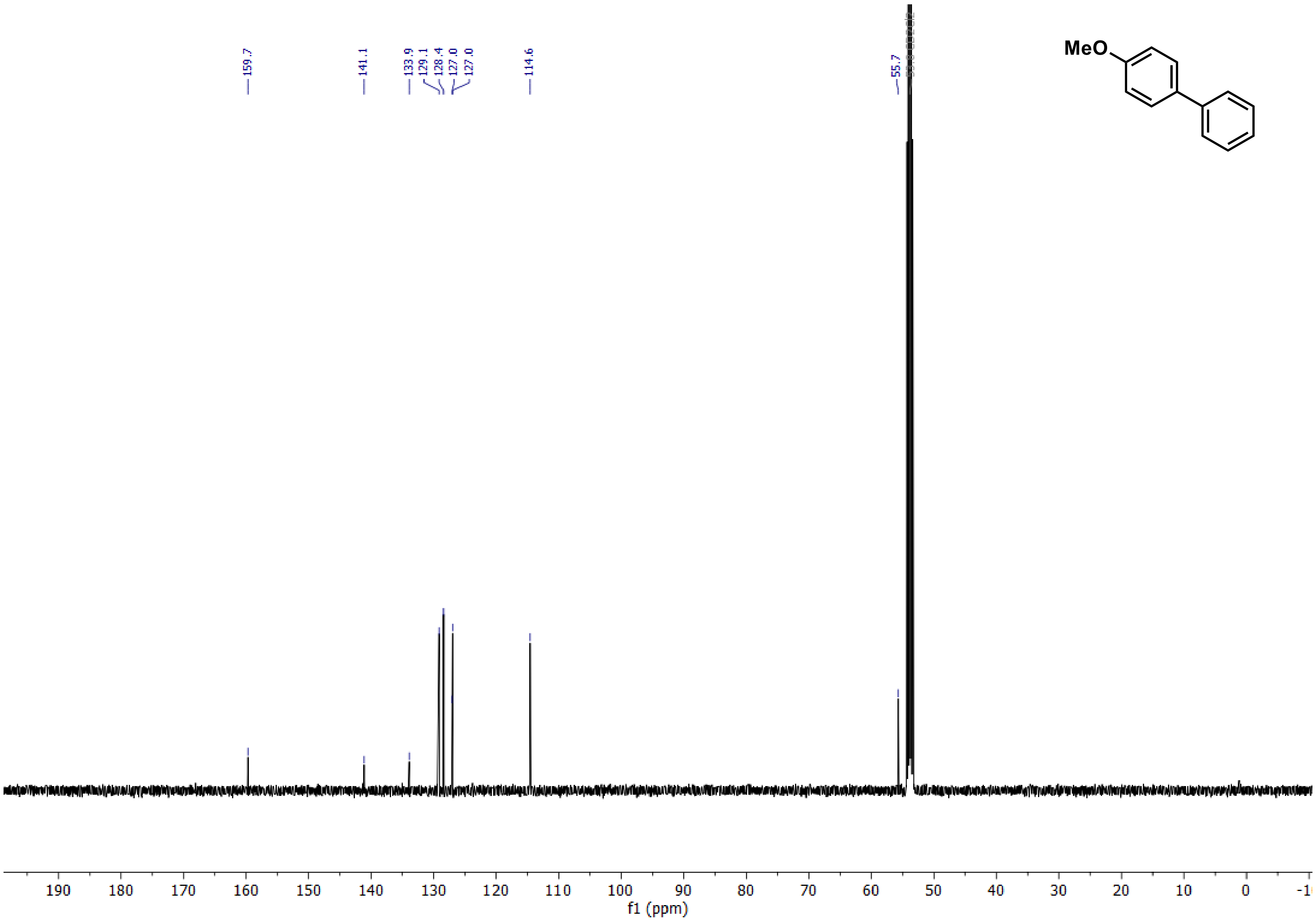

^{1}H NMR of phenyl 4-methoxybenzoate (36)

CD_2Cl_2, 298 K

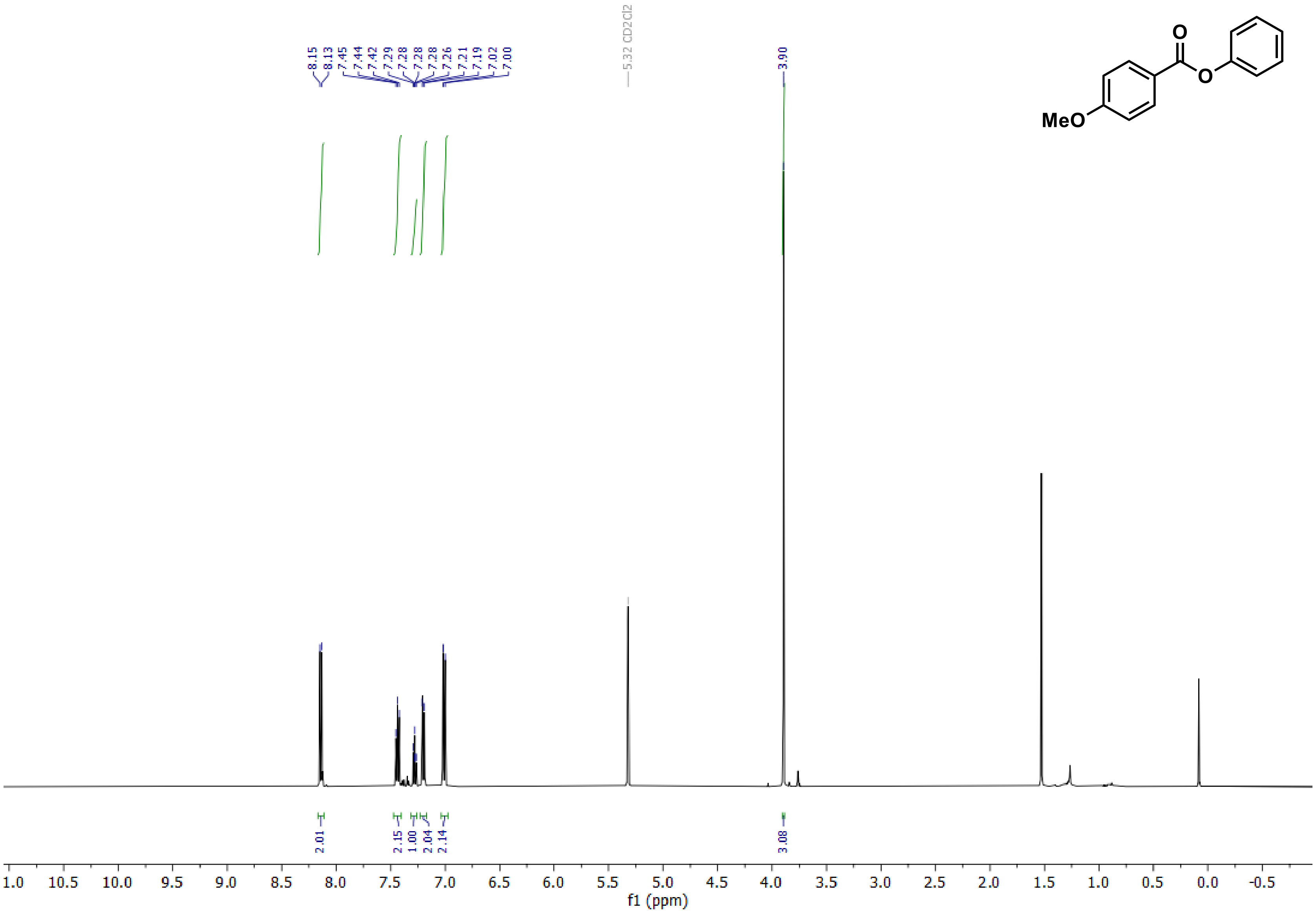

^{13}C NMR of phenyl 4-methoxybenzoate (36)

CD_2Cl_2, 298 K

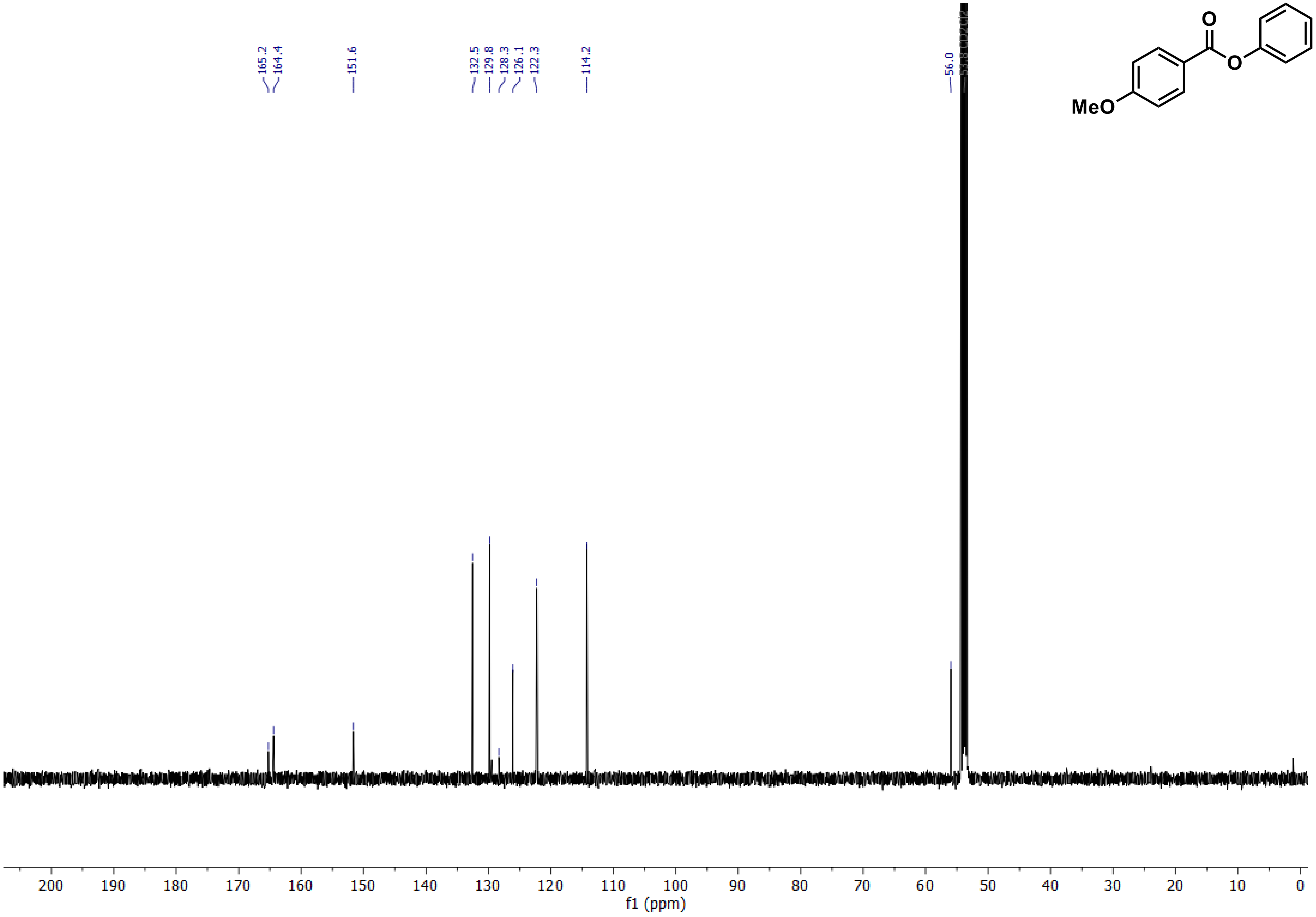

^{1}H NMR of 4-fluorophenyl thiophene-2-carboxylate (2b)

CD_3CN, 298 K

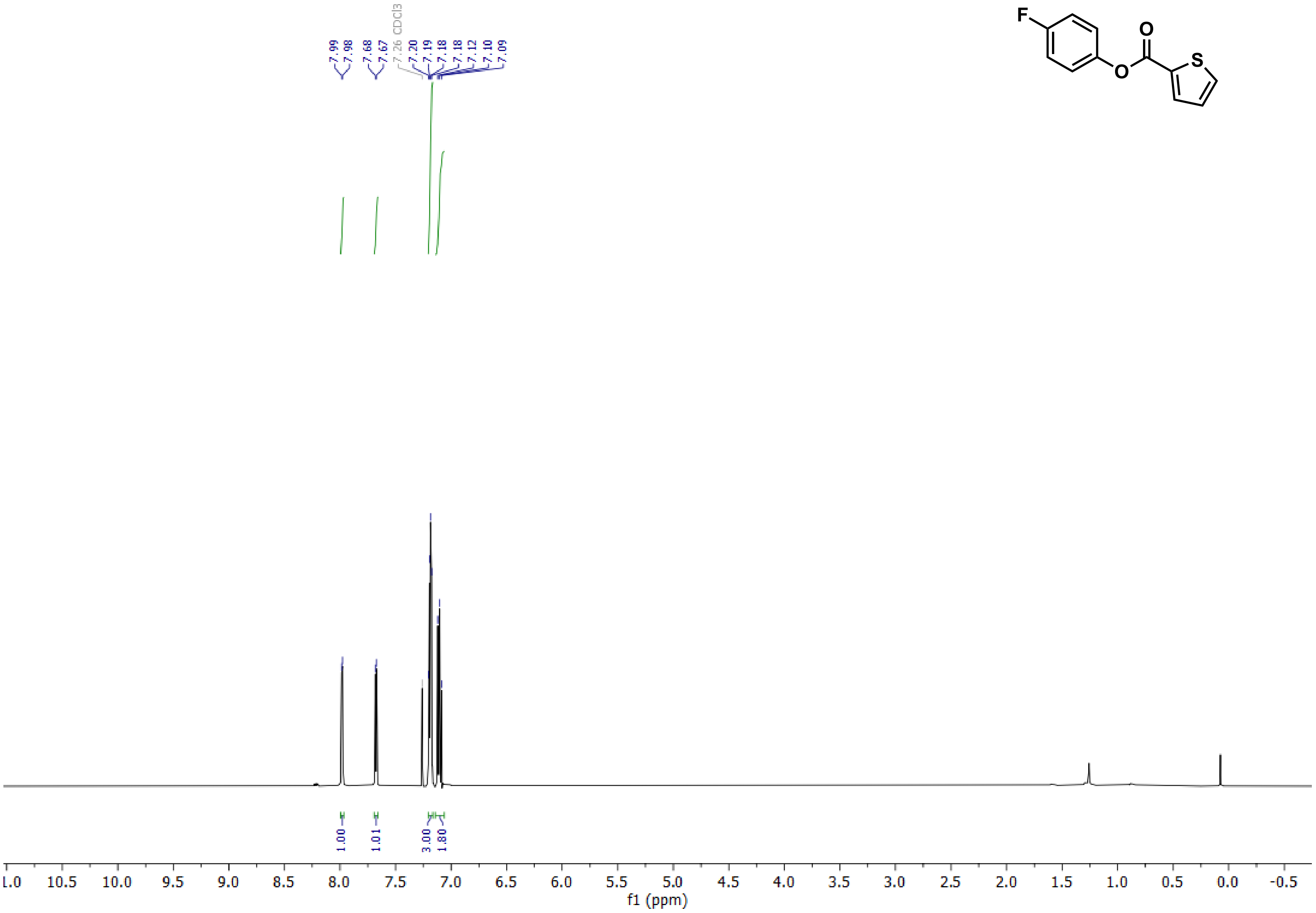

^{13}C NMR of 4-fluorophenyl thiophene-2-carboxylate (2b)

CD_3CN, 298 K

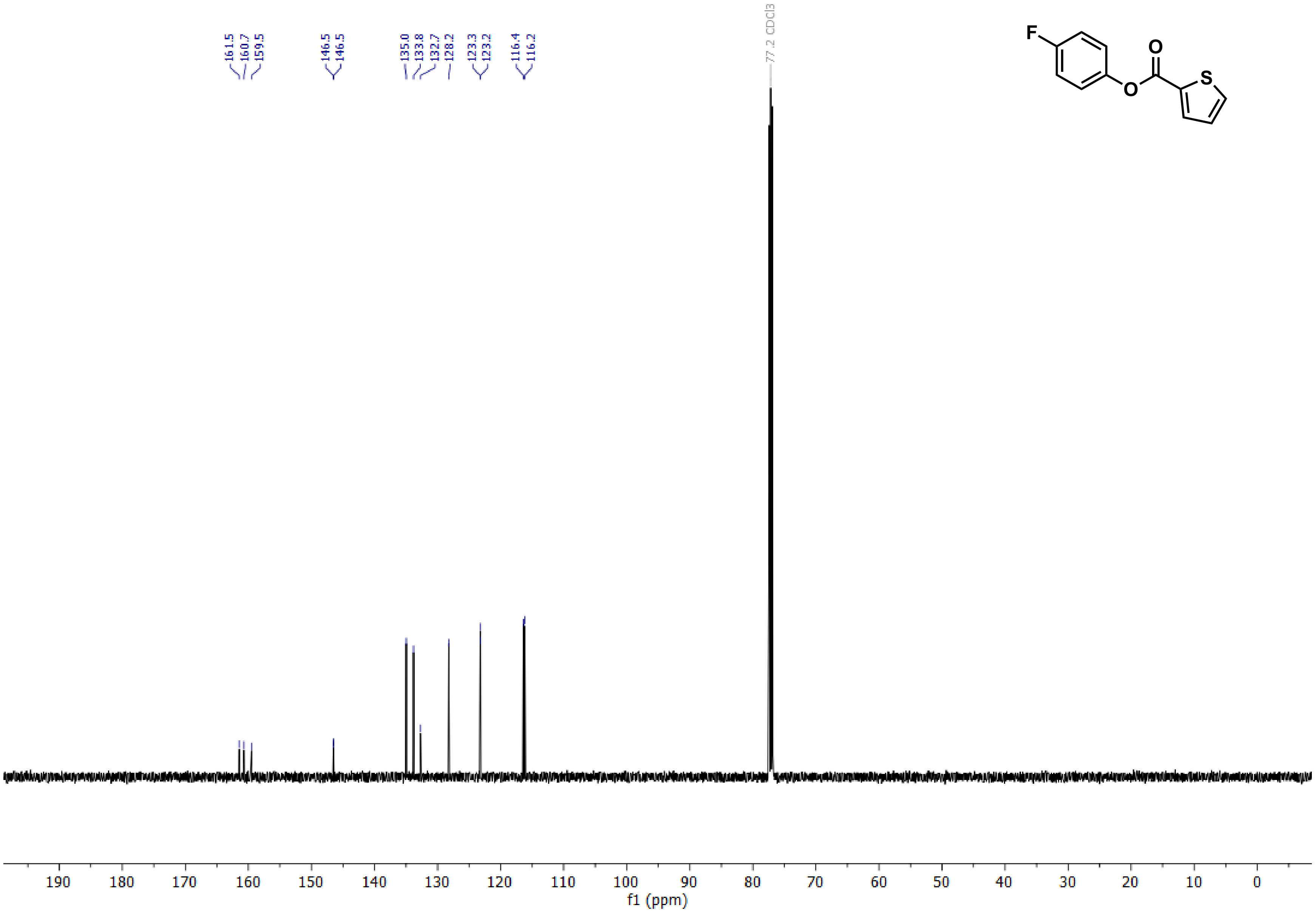

^{19}F NMR of 4-fluorophenyl thiophene-2-carboxylate (2b)

$CDCl_3$, 298 K

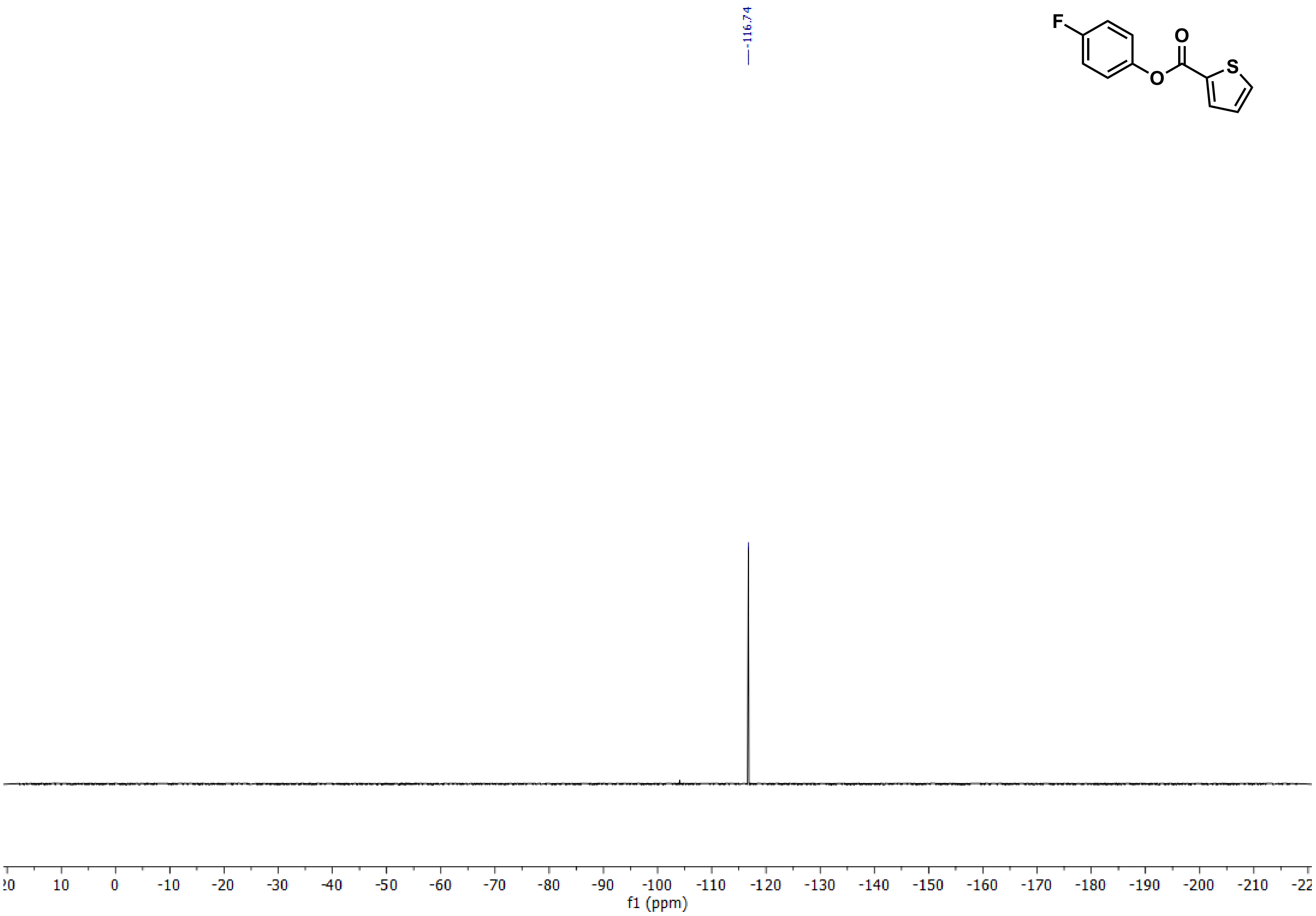

^{1}H NMR of 4-fluorophenyl 4-fluorobenzoate (2c)

$CDCl_3$, 298 K

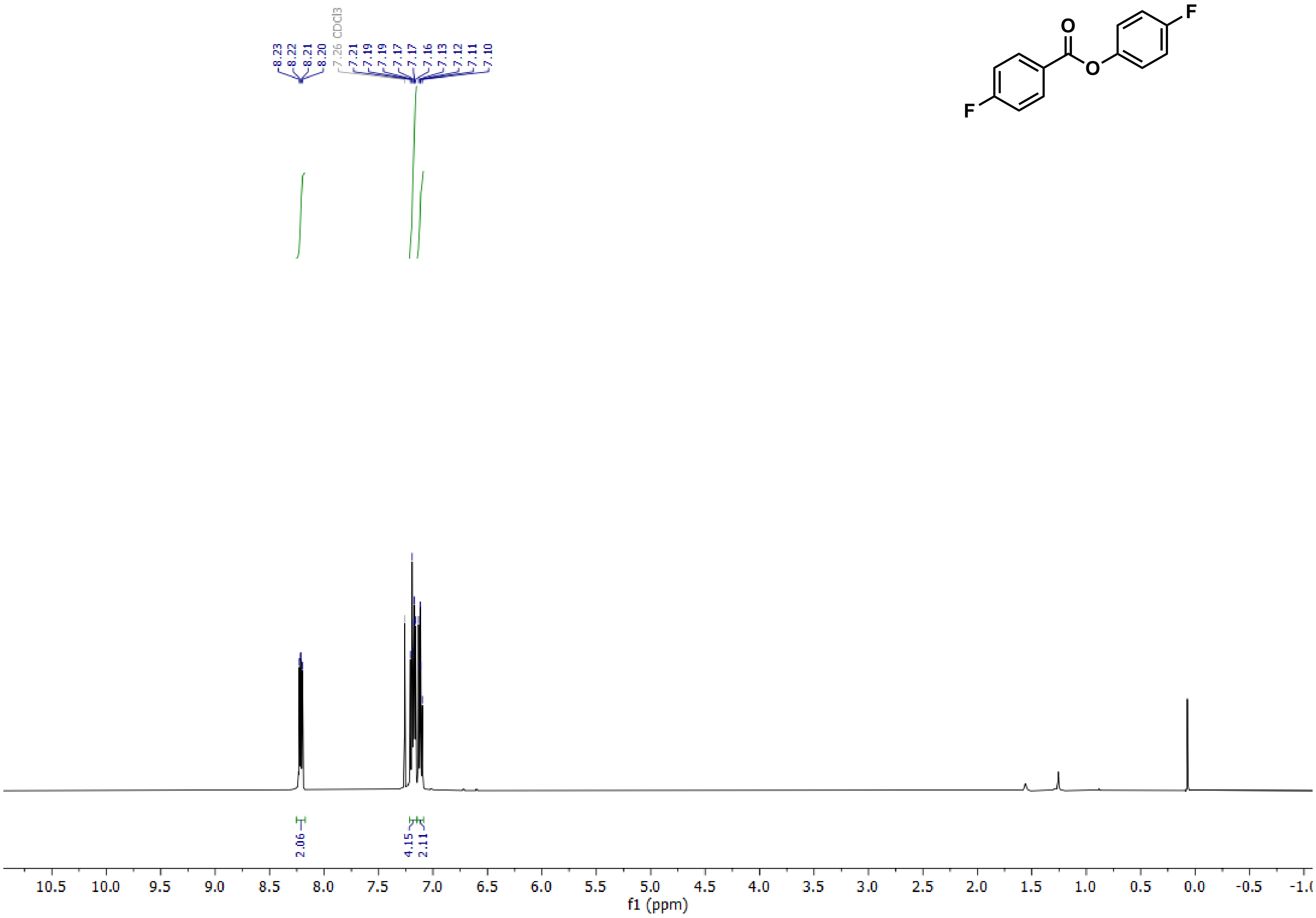

^{13}C NMR of 4-fluorophenyl 4-fluorobenzoate (2c)

$CDCl_3$, 298 K

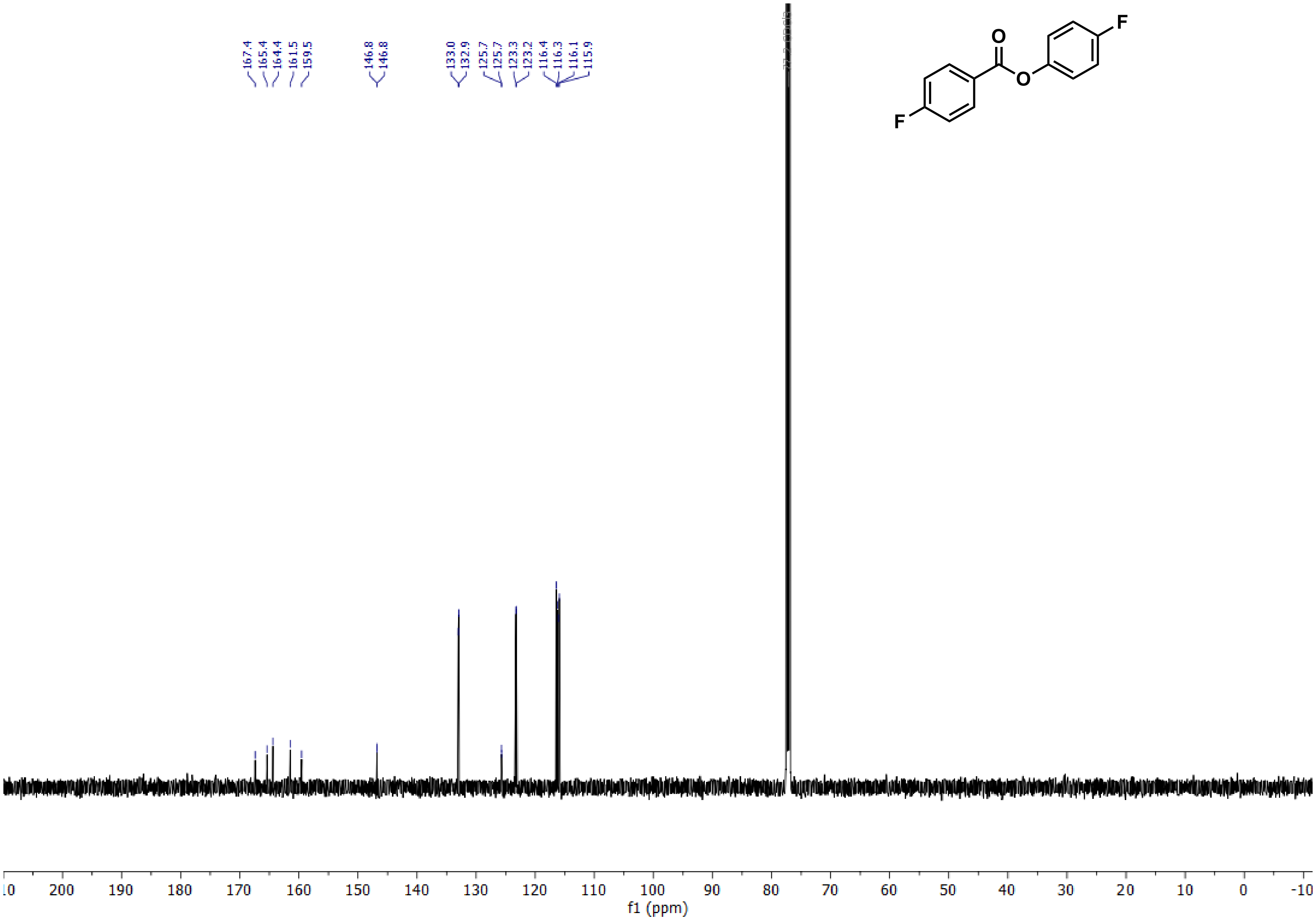

^{19}F NMR of 4-fluorophenyl 4-fluorobenzoate (2c)

CD3CN, 298 K

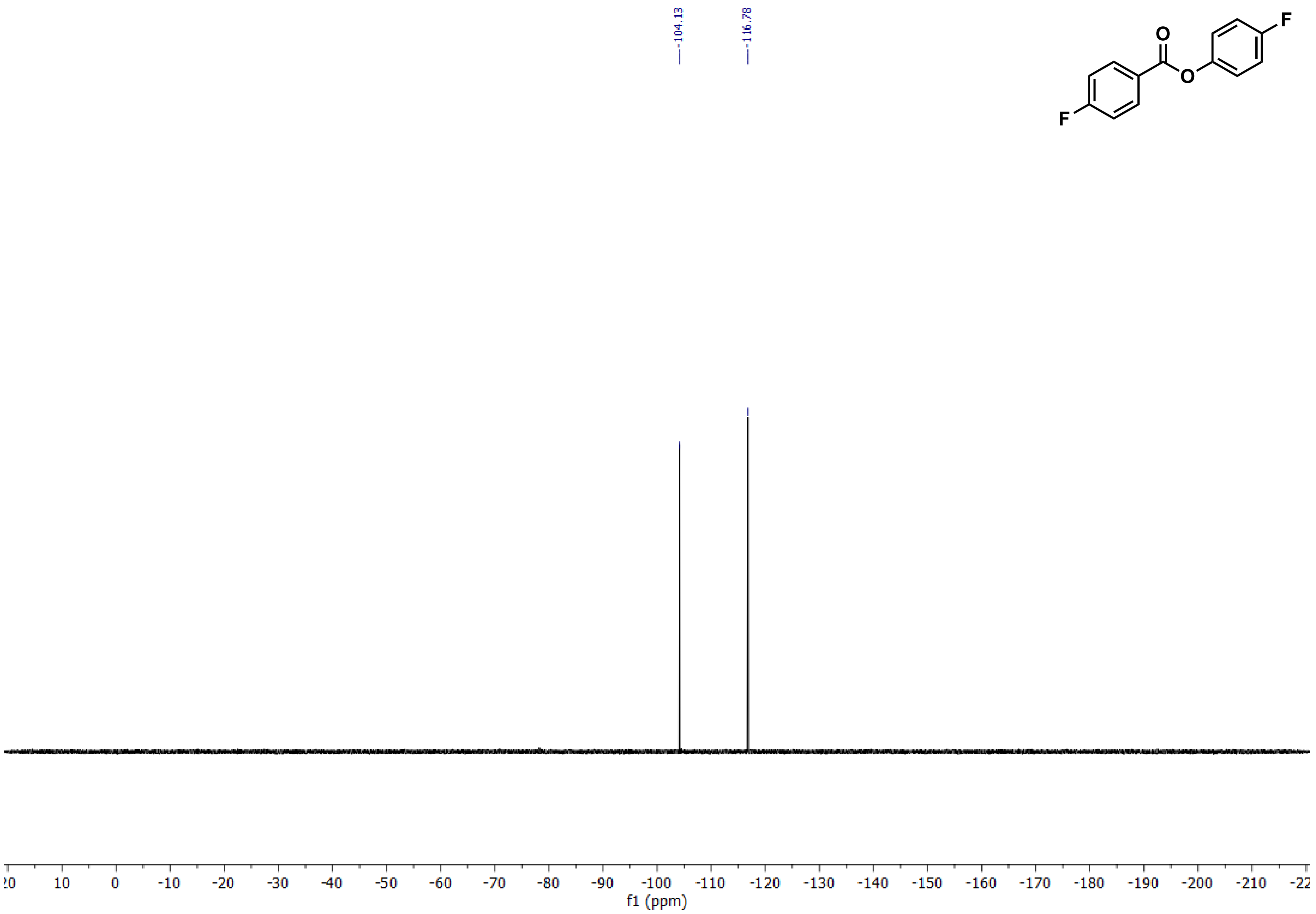

^{1}H NMR of 4-fluorophenyl 4-methoxybenzoate (2e)

$CDCl_3$, 298 K

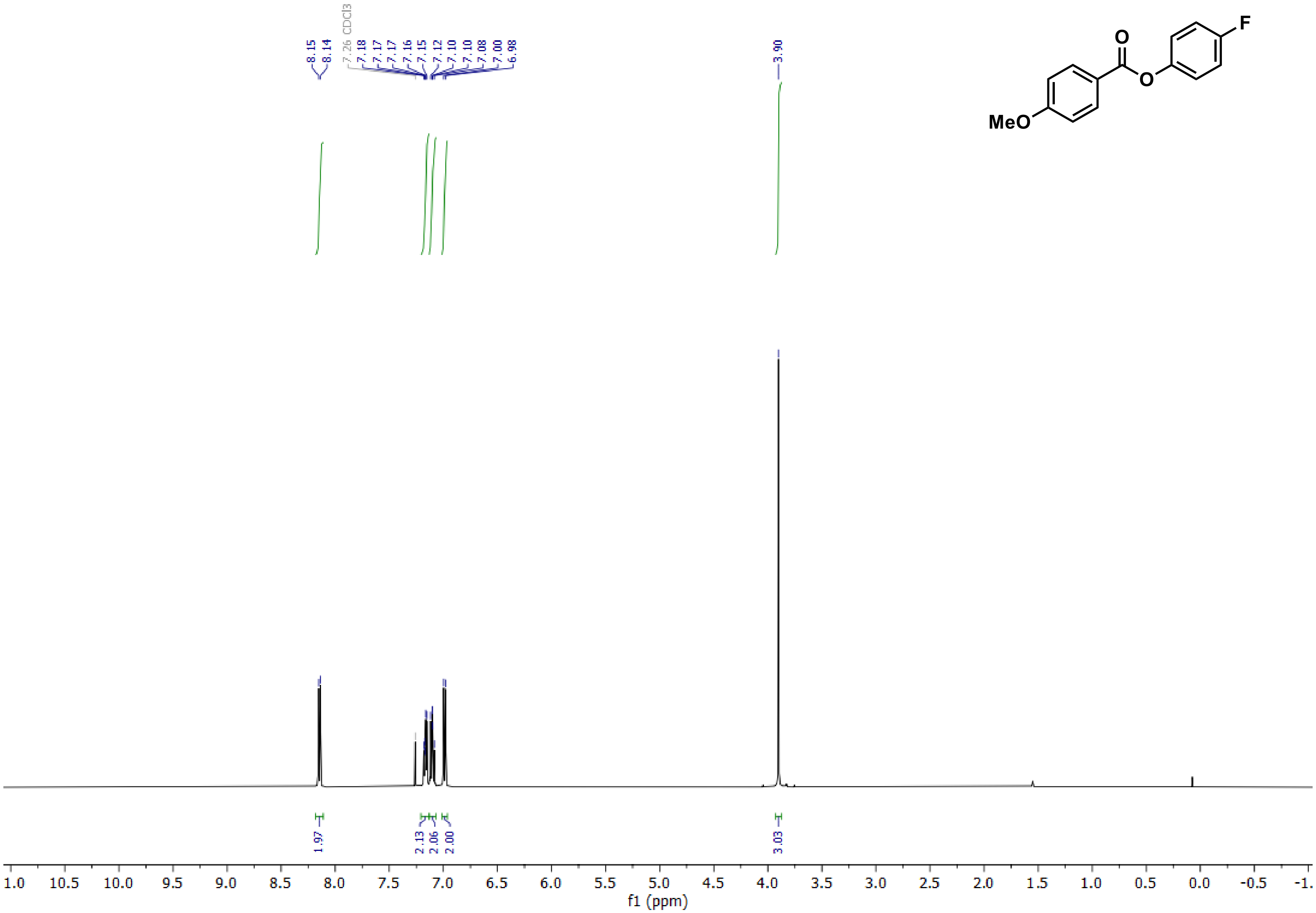

^{13}C NMR of 4-fluorophenyl 4-methoxybenzoate (2e)

$CDCl_3$, 298 K

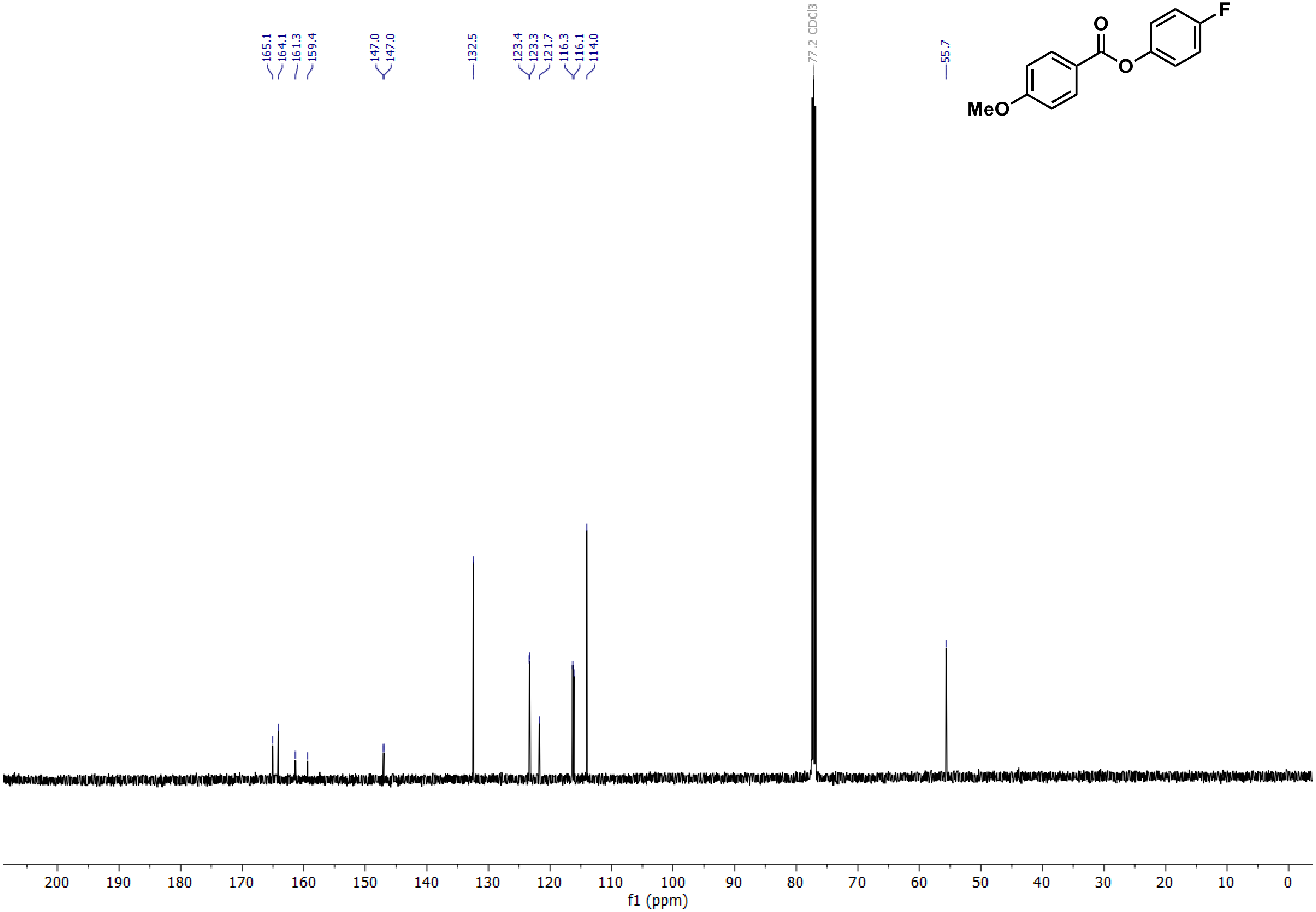

^{19}F NMR of 4-fluorophenyl 4-methoxybenzoate (2e)

$CDCl_3$, 298 K

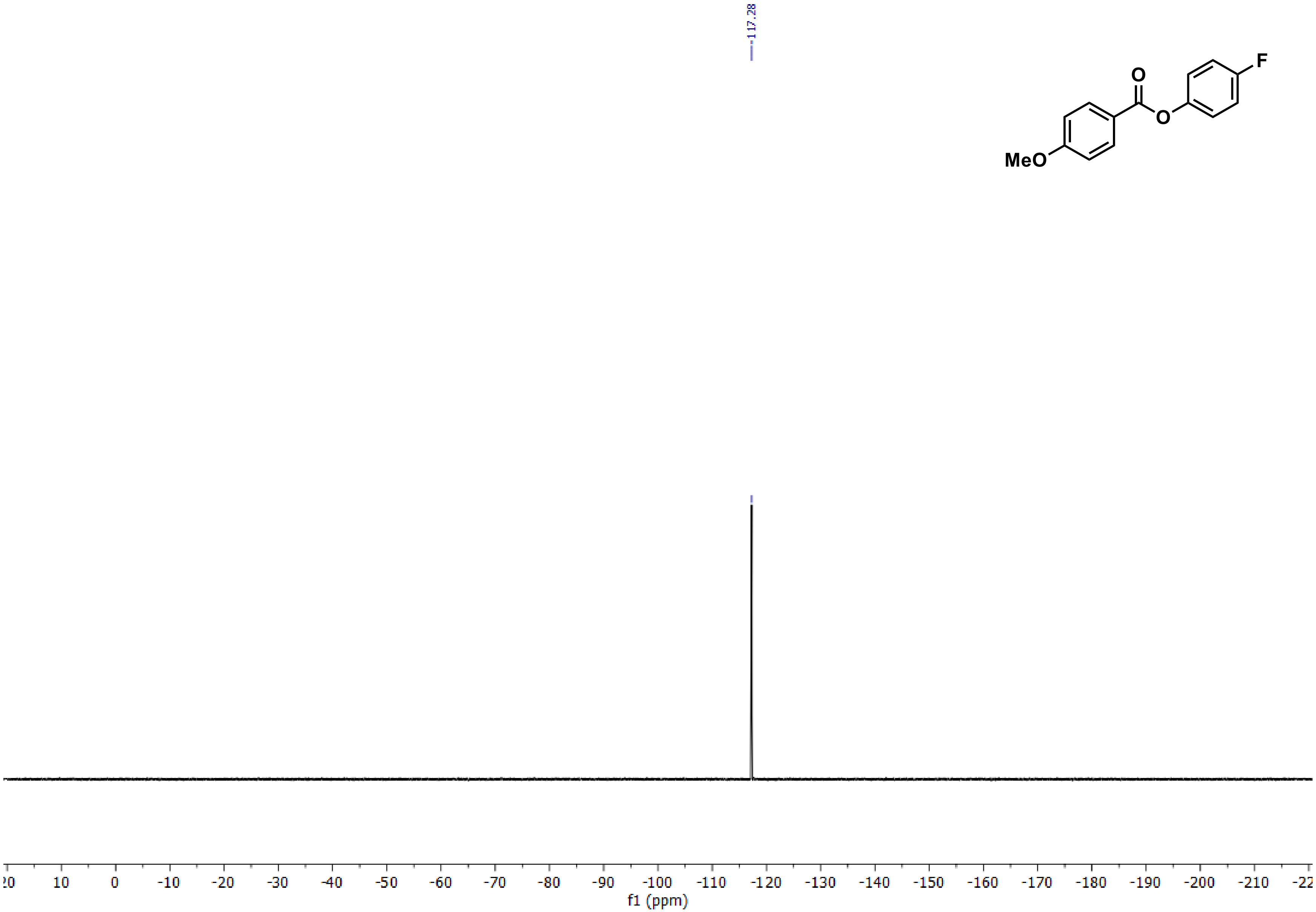

^{1}H NMR of 4-methoxyphenyl 4-fluorobenzoate (2f)

$CDCl_3$, 298 K

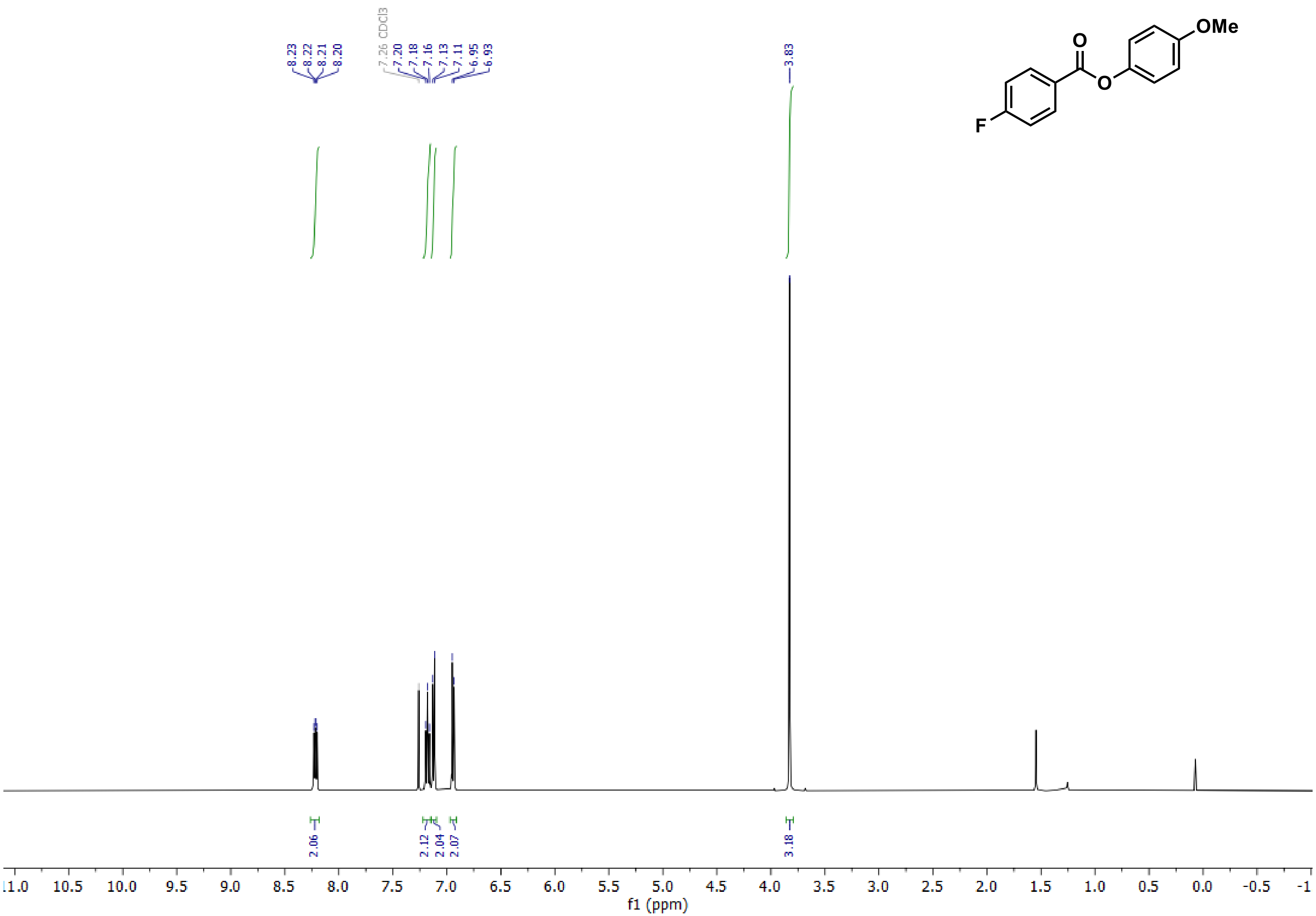

^{13}C NMR of4-methoxyphenyl 4-fluorobenzoate (2f)

$CDCl_3$, 298 K

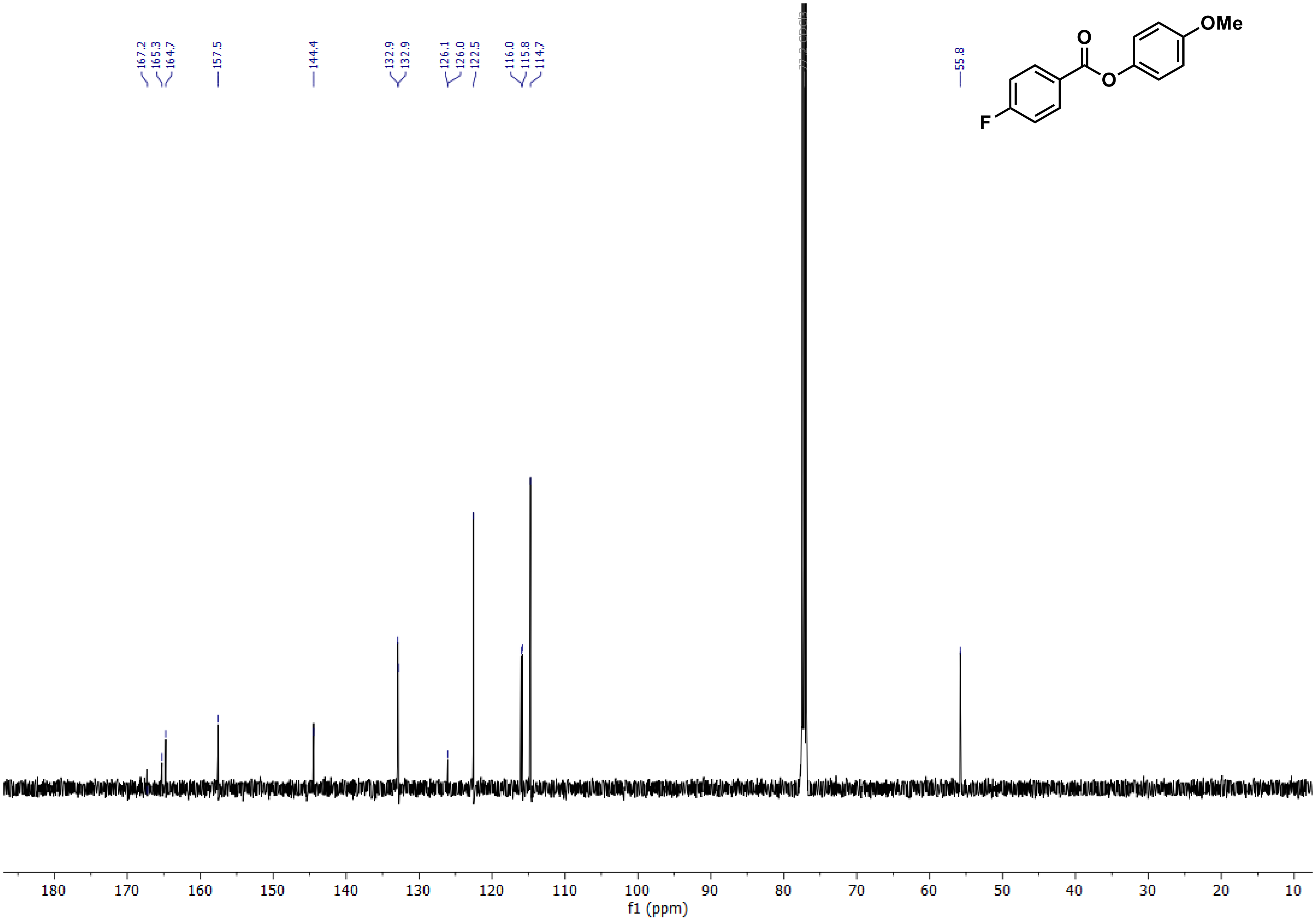

^{19}F NMR of 4-methoxyphenyl 4-fluorobenzoate (2f)

$CDCl_3$, 298 K

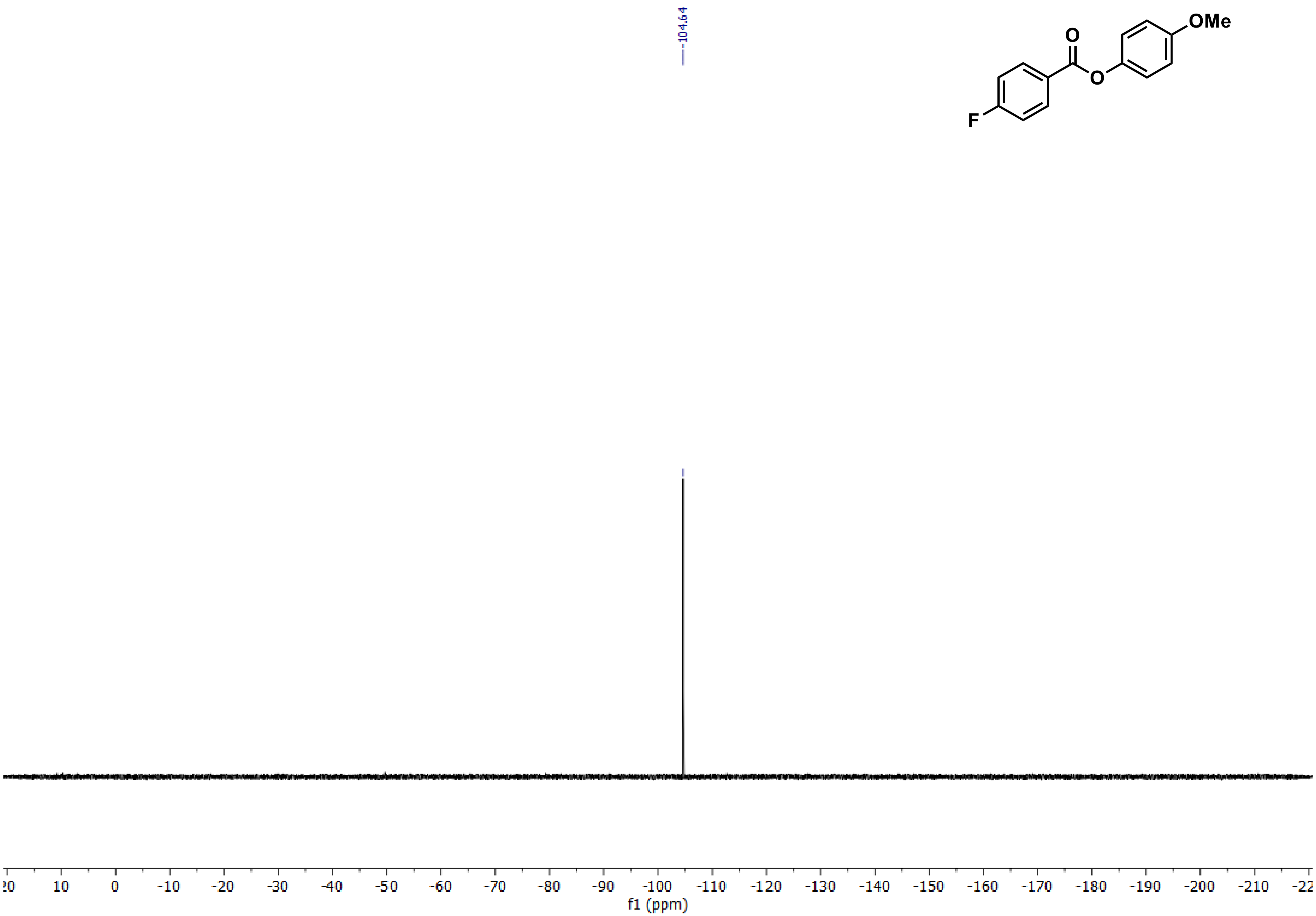

^{1}H NMR of 4-methoxyphenyl 4-methoxybenzoate (2g)

$CDCl_3$, 298 K

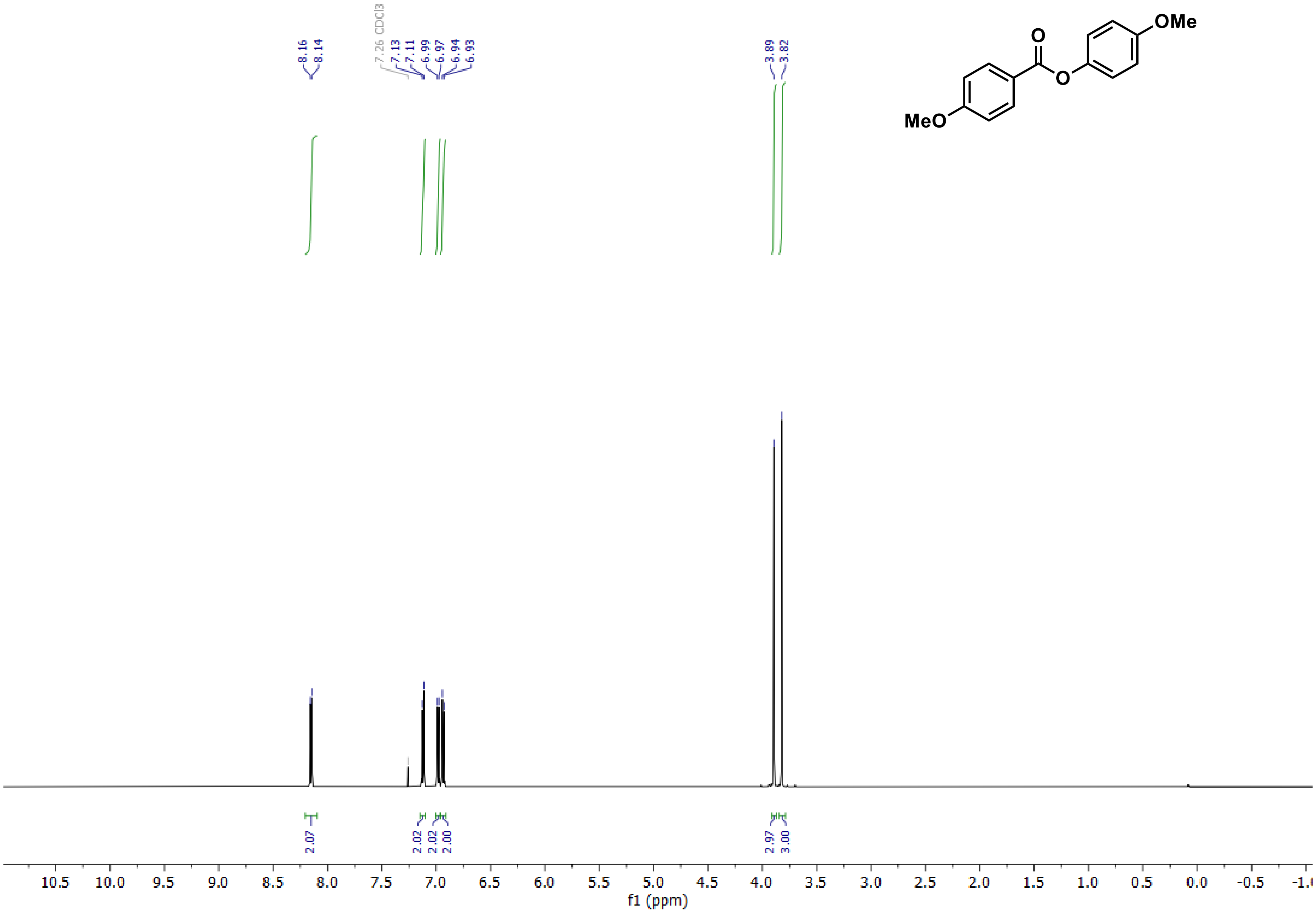

^{13}C NMR of 4-methoxyphenyl 4-methoxybenzoate (2g)

$CDCl_3$, 298 K

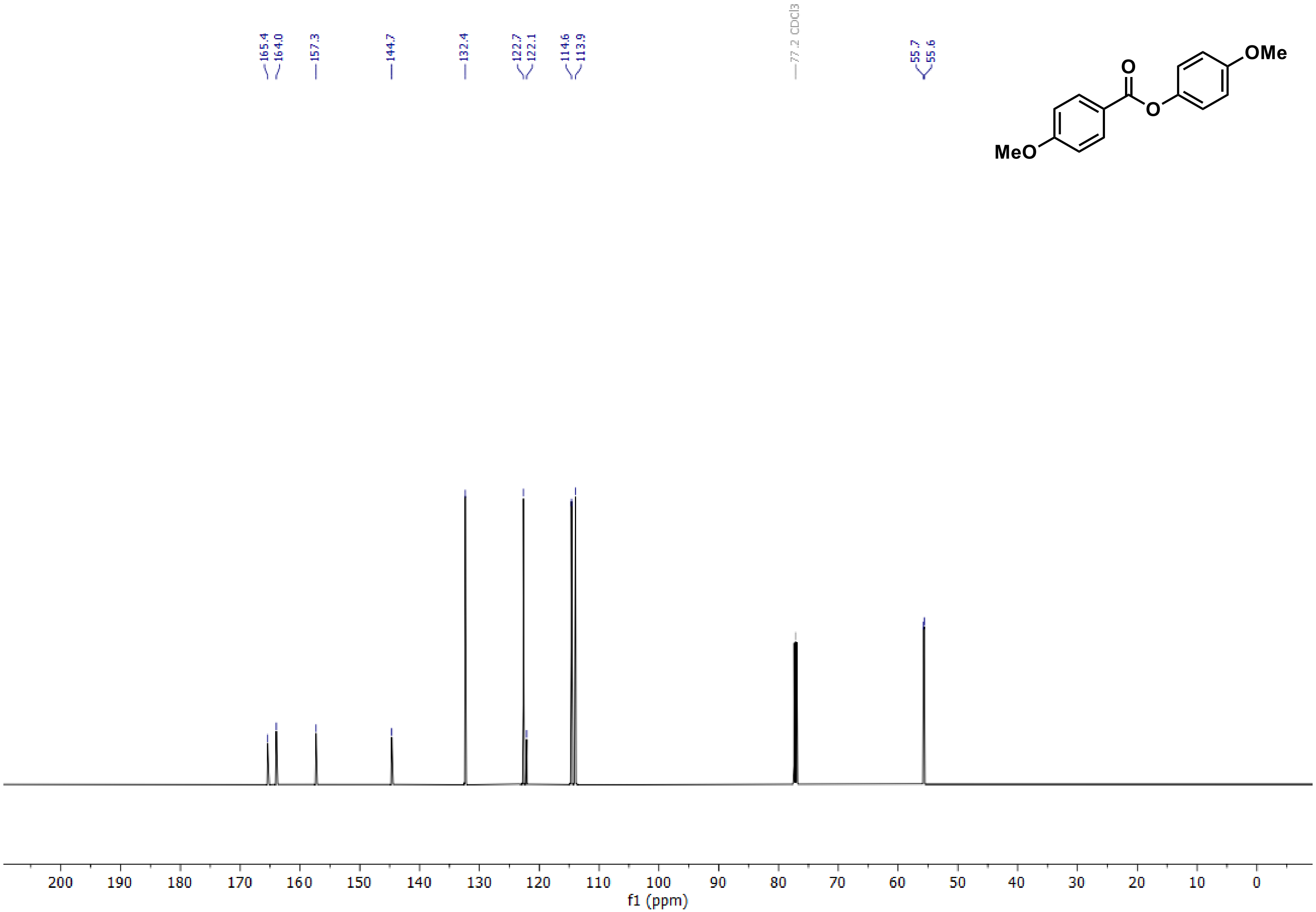

REFERENCES

1. Fulmer, G. R.; Miller, A. J. M.; Sherden, N. H.; Gottlieb, H. E.; Nudelman, A.; Stoltz, B. M.; Bercaw, J. E.; Goldberg, K. I. NMR Chemical Shifts of Trace Impurities: Common Laboratory Solvents, Organics, and Gases in Deuterated Solvents Relevant to the Organometallic Chemist. *Organometallics* **2010**, *29*, 2176–2179.
2. Xu, P.; López-Rojas, P.; Ritter, T. Radical Decarboxylative Carbometalation of Benzoic Acids: A Solution to Aromatic Decarboxylative Fluorination, *J. Am. Chem. Soc.* **2021**, *143*, 5349-5354.
3. Lill, A.; Scholich, K.; Stark, H. Synthesis of Novel Dansyl-Labeled Celecoxib Derivatives. *Tetrahedron Lett*. **2013**, *54*, 6682–6686.
4. Bell. K. H. Facile Selective Aminolysis of Phenolic Benzoates with 1-Butanamine in Benzene. *Tetrahedron Lett*. **1986**, *20*, 2263–2264.
